# Mammals on the EDGE: Conservation Priorities Based on Threat and Phylogeny

**DOI:** 10.1371/journal.pone.0000296

**Published:** 2007-03-14

**Authors:** Nick J.B. Isaac, Samuel T. Turvey, Ben Collen, Carly Waterman, Jonathan E.M. Baillie

**Affiliations:** Institute of Zoology, Zoological Society of London, London, United Kingdom; The David and Lucile Packard Foundation, Conservation and Science Program, United States of America

## Abstract

Conservation priority setting based on phylogenetic diversity has frequently been proposed but rarely implemented. Here, we define a simple index that measures the contribution made by different species to phylogenetic diversity and show how the index might contribute towards species-based conservation priorities. We describe procedures to control for missing species, incomplete phylogenetic resolution and uncertainty in node ages that make it possible to apply the method in poorly known clades. We also show that the index is independent of clade size in phylogenies of more than 100 species, indicating that scores from unrelated taxonomic groups are likely to be comparable. Similar scores are returned under two different species concepts, suggesting that the index is robust to taxonomic changes. The approach is applied to a near-complete species-level phylogeny of the Mammalia to generate a global priority list incorporating both phylogenetic diversity and extinction risk. The 100 highest-ranking species represent a high proportion of total mammalian diversity and include many species not usually recognised as conservation priorities. Many species that are both evolutionarily distinct and globally endangered (EDGE species) do not benefit from existing conservation projects or protected areas. The results suggest that global conservation priorities may have to be reassessed in order to prevent a disproportionately large amount of mammalian evolutionary history becoming extinct in the near future.

## Introduction

Our planet is currently experiencing a severe anthropogenically driven extinction event, comparable in magnitude to prehistoric mass extinctions. Global extinction rates are now elevated up to a thousand times higher than the background extinction rates shown by the fossil record, and may climb another order of magnitude in the near future [1]–[3]. The resources currently available for conservation are, unfortunately, insufficient to prevent the loss of much of the world's threatened biodiversity during this crisis, and conservation planners have been forced into the unenviable situation of having to prioritise which species should receive the most protection–this is ‘the agony of choice’ [4] or the ‘Noah’s Ark problem' [5].

A range of methods for setting species-based conservation priorities have been advocated by different researchers or organisations, focusing variously on threatened species, restricted-range endemics, ‘flagship’, ‘umbrella’, ‘keystone’, ‘landscape’ or ‘indicator’ species, or species with significant economic, ecological, scientific or cultural value [6]–[8]. To date, global priority-setting exercises have tended to focus on endemic (or restricted range) species [6], [9], [10], presumably because endemism is easier to measure than competing methods. However, recent data show that endemism is a poor predictor of total species richness or the number of threatened species [11].

It has also been argued that maximising Phylogenetic Diversity (PD) should be a key component of conservation priority setting [4], [12]–[14]. Species represent different amounts of evolutionary history, reflecting the tempo and mode of divergence across the Tree of Life. The extinction of a species in an old, monotypic or species-poor clade would therefore result in a greater loss of biodiversity than that of a young species with many close relatives [15], [16]. However, conserving such lineages may be difficult, since there is some evidence that they are more likely to be threatened with extinction than expected by chance [17]. This clumping of extinction risk in species-poor clades greatly increases the loss of PD compared with a null model of random extinction [18] and suggests that entire vertebrate orders may be lost within centuries [19]. Among mammals alone, at least 14 genera and three families have gone extinct since AD 1500 [20], and all members of a further 19 families and three orders are considered to be in imminent danger of extinction [2]. Many academic papers have suggested ways to maximise the conservation of PD [e.g. 12], [13], [21]–[23] and measure species' contributions to PD [e.g. 4], [23]–[25], but these have rarely been incorporated into conservation strategies. Therefore, it is possible that evolutionary history is being rapidly lost, yet the most distinct species are not being identified as high priorities in existing conservation frameworks.

There are several reasons why PD has not gained wider acceptance in the conservation community. First, although evolutionary history consists of two distinct components (the branching pattern of a phylogenetic tree and the length of its branches), complete dated species-level phylogenies for large taxonomic groups have only recently become available [26]. Early implementations of PD-based approaches were therefore unable to incorporate branch length data, and focused solely on measurements of branching pattern [4]. Second, PD removes the focus from species and so may lack wider tangible appeal to the public; conserving PD may be seen as less important than the protection of endemic or threatened species [16]. However, the current instability in species taxonomy [27] means that decisions based on PD might be more objective than those based on different species concepts [13], [16], [27]. Combining species' conservation status with a measure of their contribution to PD is therefore desirable, because species can be retained as units but weighted appropriately [5], [22]. This would generate a useful and transparent means for setting global priorities for species-based conservation [25].

This paper describes a new method for measuring species' relative contributions to phylogenetic diversity [the ‘originality’ of species: ref 24]. We explore the statistical properties of the resulting measure, which we call Evolutionary Distinctiveness (ED), and test its robustness to changing species concepts. ED scores are calculated for the Class Mammalia, and combined with values for species' extinction risk to generate a list of species that are both evolutionarily distinct and globally endangered (‘EDGE species’). The resultant list provides a set of priorities for mammalian conservation based not only on the likelihood that a species will be lost, but also on its irreplaceability.

### Evolutionary Distinctiveness and its use in priority-setting

In order to calculate ED scores for each species, we divide the total phylogenetic diversity of a clade amongst its members. This is achieved by applying a value to each branch equal to its length divided by the number of species subtending the branch. The ED of a species is simply the sum of these values for all branches from which the species is descended, to the root of the phylogeny. For the examples in this paper, we have measured ED in units of time, such that each million years of evolution receives equal weighting and the branches terminate at the same point (i.e. the phylogeny is ultrametric). The method could be applied to non-ultrametric phylogenies if the conservation of other units [e.g. character diversity 28], [29] was prioritised [although see ref 30].

**Figure 1:**
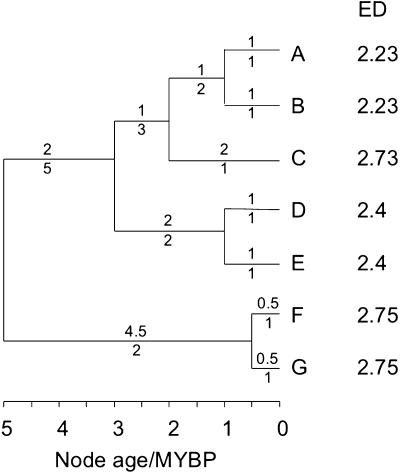
Hypothetical phylogeny of seven species (A–G) with Evolutionary Distinctiveness (ED) scores. Numbers above each branch indicate the length; numbers below show the number of descendent species. MYBP, millions of years before present.

The basic procedure for calculating ED scores is illustrated in figure 1, which describes a clade of seven species (A–G). The ED score of species A is given by the sum of the ED scores for each of the four branches between A and the root. The terminal branch contains just one species (A) and is 1 million years (MY) long, so receives a score of 1 MY. The next two branches are both 1 MY long and contain two and three species, so each daughter species (A, B and C) receives 1/2 and 1/3 MY respectively. The deepest branch that is ancestral to species A is 2 MY long and is shared among five species (A to E), so the total ED score for species A is given by (1/1+1/2+1/3+2/5) = 2.23 MY. Species B is the sister taxon of A, so receives the same score. By the same arithmetic, C has a score of (2/1+1/3+2/5) = 2.73 MY, both D and E receive (1/1+2/2+2/5) = 2.4 MY, and both F and G receive (0.5/1+4.5/2) = 2.75 MY. The example illustrates that ED is not solely determined by a species' unique PD (i.e. the length of the terminal branch). Species F and G are the top-ranked species based on their ED scores, even though each represents just a small amount of unique evolutionary history (0.5 MY). This suggests that the conservation of both F and G should be prioritised, because the extinction of either would leave a single descendant of the oldest and most unusual lineage in the phylogeny [c.f. 15], [24]. The ED calculation is similar to the Equal Splits measure [25], which apportions branch length equally among daughter clades, rather than among descendent species.

In order to represent a useful tool in priority setting, ED scores must be applicable in real phylogenies of large taxonomic groups. To do this, we modified the basic procedure described above to control for missing species, incomplete phylogenetic resolution and uncertainty in node ages (see Materials and Methods). The approach is implemented using a dated phylogeny of the Class Mammalia that is nearly complete (>99%) at the species level [31]. We then combined ED and extinction risk to identify species that are both evolutionarily distinct and globally endangered (‘EDGE species’). We measured extinction risk using the quantitative and objective framework provided by the World Conservation Union (IUCN) Red List Categories [2]. We follow previous researchers in treating the Red List categories as intervals of extinction risk and by assuming equivalence among criteria [32], [33], [but see 34]. The resulting list of conservation priorities (‘EDGE scores’) was calculated as follows:
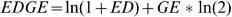
1where GE is the Red List category weight [Least Concern = 0, Near Threatened and Conservation Dependent = 1, Vulnerable = 2, Endangered = 3, Critically Endangered = 4, ref 32], here representing extinction risk on a log scale. EDGE scores are therefore equivalent to a log_e_-transformation of the species-specific expected loss of evolutionary history [5], [25] in which each increment of Red List category represents a doubling (*e* ^ln(2)^) of extinction risk. For the purposes of these analyses, we did not calculate EDGE scores for species listed as Extinct in the Wild (n = 4), domesticated populations of threatened species and 34 species (mostly of dubious taxonomic status) for which an evaluation has not been made.

## Results

### Statistical properties of ED

**Figure 2:**
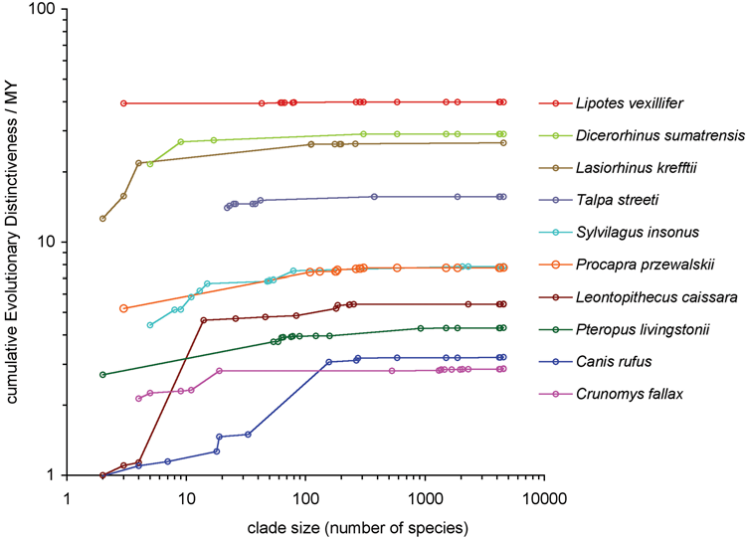
Scaling of ED scores with clade size for ten Critically Endangered mammal species. ED scores were calculated at each node between the tips and root for ten species in different orders. Species chosen are: the baiji (*Lipotes vexillifer*), sumatran rhino (*Dicerorhinus sumatrensis*), northern hairy-nosed wombat (*Lasiorhinus krefftii*), persian mole (*Talpa streeti*), Omiltemi rabbit (*Sylvilagus insonus*), Przewalski's gazelle (*Procapra przewalskii*), black-faced lion tamarin (*Leontopithecus caissara*), Livingstone's flying fox (*Pteropus livingstonii*), red wolf (*Canis rufus*) and northern Luzon shrew rat (*Crunomys fallax*). See Materials and Methods for further details.

We measured ED in clades of different sizes to test whether ED scores from different taxonomic groups are likely to be comparable. We found that most ED is derived from a few branches near the tips (i.e. those shared with few other species) and that virtually no ED is gained in clades above ∼180 species (figure 2). Median ED in clades of 60 species is 88% of the total accumulated using the whole tree (n = 10, figure 2). Moreover, the rank order of ED scores is unaffected by the size of the clade under consideration, except in very small clades and among species with low overall ED (i.e. few of the lines in figure 2 cross one another). These findings suggest that ED scores of different taxonomic groups measured on separate phylogenies (i.e. with no nodes in common) will be comparable, so long as each phylogeny is larger than a threshold size. Based on the scaling observed in figure 2, we suggest a minimum species richness of 100 as a useful rule of thumb to ensure comparability among taxa.

Although most species (90% in figure 2) derive at least two-thirds of their total ED from the terminal branch (which is not shared with others), this branch length is a poor predictor of total ED (r^2^ = 0.03 on a log-log scale). For species on short branches, there is an order of magnitude difference between the length of the terminal branch and ED. For example, the pale-throated and brown-throated three-toed sloths (*Bradypus tridactylus* and *B. variegatus*) share a common ancestor thought to be just over a million years old, but the total ED of both species is 20.4 MY (Table S1) since they have few close living relatives.

ED scores are also robust to taxonomic changes. For example, ED scores in primates under the biological species concept [35] are tightly correlated with ED scores under the phylogenetic species concept [36] (r^2^ = 0.65 on a log-log scale), in spite of the fact that there are substantial differences between the two: the number of primate species differs by 50%. Furthermore, the highest-ranking species do not change their identity: 45 of 58 biological species in the upper quartile of ED scores are also in the upper quartile as phylogenetic species. However, species that have been split into three or more species do tend to lose a large portion of their ED. For example, the fork-marked lemur (*Phaner furcifer*) is the second most distinct biological species of primate, with an ED score of 38.33. It was split into four phylogenetic species [36] with an ED score of 10.45 (Table S2), which is just inside the upper quartile.

### ED and EDGE scores in mammals

Mammal ED scores range from 0.0582 MY (19 murid rodents) to 97.6 MY (duck-billed platypus, *Ornithorhynchus anatinus*). Scores are approximately log-normally distributed, with a median of 7.86 MY and geometric mean of 6.28 MY.

Evolutionary Distinctiveness is not evenly distributed among the Red List categories. Least Concern species have significantly lower ED than the other categories (F_1,4180_ = 26.3, *p*<0.0001, using log_e_ transformed scores); there are no significant differences among the remaining categories. This suggests that species with low ED scores tend to suffer from low levels of extinction risk, although the explanatory power of this model is extremely low (r^2^ = 0.006).

**Figure 3:**
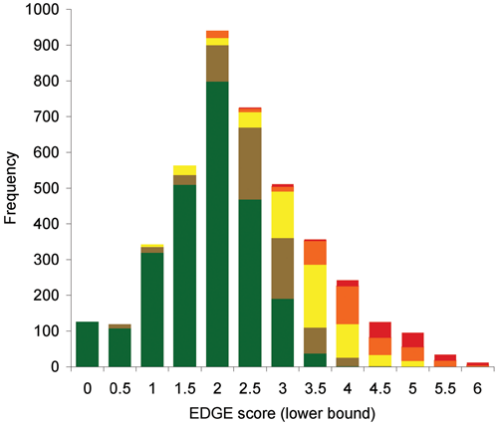
Histogram of EDGE scores for 4182 mammal species, by threat category. Colours indicate the Red List category: Least Concern (green), Near Threatened and Conservation Dependent (brown), Vulnerable (yellow), Endangered (orange) and Critically Endangered (red).

EDGE scores range from 0.0565 (10 murid rodents) to 6.48 (Yangtze River dolphin or baiji, *Lipotes vexillifer*) and are approximately normally distributed around a mean of 2.63 (±0.017; figure 3). The 100 highest priority (EDGE) species includes several large-bodied and charismatic mammals, including the giant and lesser pandas, the orang-utan, African and Asian elephants, four rhinoceroses, two tapirs, two baleen whales, a dugong and a manatee. However, many smaller and less appreciated species also receive high priority, including sixteen rodents, thirteen eulipotyphlans, twelve bats, four lagomorphs and an elephant shrew (Table S1). The top 100 also includes at least 37 species that would not qualify for most area-based definitions of endemism, since they are listed as threatened under Red List criterion A (reduction in population size) without qualifying for criteria B–D, which are based on population size or geographical range. Whilst the highest-ranked species, by definition, are all highly threatened (44 of the top 100 species are Critically Endangered, a further 47 are Endangered), threat status alone does not guarantee a high priority. For example, 10 Critically Endangered species (in the genera *Gerbillus, Peromyscus* and *Crocidura*), as well as 32 Endangered species, fail to make the top 1000, whilst 130 Near Threatened species do.

## Discussion

It is important that conservation priority-setting approaches are able to satisfy two conditions: they capture biodiversity and are robust to uncertainty. The method described herein satisfies the first condition because EDGE scores incorporate species value (in terms of originality, or irreplaceability) weighted by urgency of action (i.e. risk of extinction). Our approach satisfies the second condition because the scores are also robust to clade size, missing species and poor phylogenetic resolution. EDGE scores are also easy to calculate, as all that is required is a set of Red List assessments and a near-complete phylogeny containing at least 100 species.

In particular, EDGE priorities are much less sensitive to taxonomic uncertainty than alternate methods. The current trend towards the adoption of the phylogenetic species concept among biologists [27] is likely to produce a large number of ‘new’ threatened and endemic species [37], potentially altering the distribution of hotspots [38] and distorting other biodiversity patterns [27]. The EDGE approach is robust to such distortion because any increase in extinction risk due to splitting is balanced by a decrease in ED. A good example is that of the ruffed lemurs (*Varecia* spp.), which consist of one Endangered biological species (ED = 19.8; EDGE = 5.11) or two phylogenetic species (Endangered and Critically Endangered; ED = 10.3; EDGE = 4.50 and 5.20). Using the same approach, we estimate that the long-beaked echidna (*Zaglossus bruijni*) would fall from the second-ranked priority to the 20^th^ after the addition of two new congeners [suggested by 39]. Thus, EDGE scores for existing species are robust to the ongoing discovery of new species.

EDGE priorities are also robust to several other forms of uncertainty. Like all phylogenetic methods, the precise EDGE scores are dependent on the topology and branch lengths of the phylogeny. However, errors in the phylogeny are unlikely to alter the identity of high-ranking species, particularly for clades of several hundred species. Topological uncertainty is usually expressed in supertrees as polytomies, which are accounted for using simple correction factors. Likewise, branch length uncertainty has been incorporated into the scoring system to down-weight the priority of species descended from nodes with imprecisely estimated ages (see Materials and Methods). These developments make it possible to estimate robustly the contribution to phylogenetic diversity of species in poorly known clades. The other major source of uncertainty is in estimating extinction risk: most recent changes in Red List category have come about through improvements in knowledge, rather than genuine changes in status [32]. EDGE scores will inevitably be affected by future changes in extinction risk, although no more so than other approaches using the Red List categories.

A minority of mammal species could not be assigned EDGE scores. Around 300 species are classified as Data Deficient and could not be meaningfully included, although in reality they may have a high risk of extinction [17]. By far the most likely candidate for high EDGE status following future Red List re-assessment is the franciscana or La Plata River dolphin *Pontoporia blainvillei* (ED = 36.3 MY). In addition, fifty extant species are missing from the phylogeny. The highest ranked of these are probably a pair of Critically Endangered shrews (*Sorex cansulus* and *S. kizlovi*); median and maximum ED scores for the genus are 4.55 and 14.6 MY, giving potential respective EDGE scores of 4.49 and 5.52 for these species (cf. figure 3). A further 260 species have been described since the chosen taxonomy was published [40]. Of these, the recently described Annamite striped rabbit *Nesolagus timminsi* [41] is the sister species to the tenth-ranked Sumatran rabbit *N. netscheri*, so would be a high priority if similarly threatened.

It has been suggested that species with few close relatives (i.e. high ED) are ‘relicts’ or ‘living fossils’ that have limited ability to generate novel diversity. This view implies that conservation efforts should instead be focused on recent radiations containing species with low ED scores (e.g. murid rodents), which represent ‘cradles’ rather than ‘museums’ of diversity [e.g. 16], [42]. However, the assumption that we are able to predict future evolutionary potential is dubious and no general relationships between phylogeny and diversity over geological time have yet been established [43], [44]. Furthermore, phylogenetic diversity is clearly related to character diversity [30], and so ED may be a useful predictor of divergent properties and hence potential utilitarian value [14]. Moreover, because species with low ED scores tend to suffer from low levels of extinction risk, phylogenetic cradles of mammalian diversity are likely to survive the current extinction crisis even without specific interventions. Focusing on lower risk species, at the expense of EDGE priorities, would therefore result in a severe pruning of major branches of the Tree of Life comparable to that seen in previous mass extinction events [45], [46].

The top 100 EDGE species span all the major mammalian clades [being distributed among 18 orders and 52 families recognised by ref 35] and display a comparable range of morphological and ecological disparity, including the largest and smallest mammals, most of the world's freshwater cetaceans, an oviparous mammal and the only species capable of injecting venom using their teeth. However, around three-quarters of species-based mammal conservation projects are specifically aimed at charismatic megafauna [47], so conventional priority-setting tools may not be sufficient to protect high priority EDGE species. This concern is supported by two additional lines of evidence. First, we found that species not found in protected areas [‘gap species’ defined by ref 48] tended to have higher EDGE scores than those found inside protected areas (logistic regression: χ^2^_1,3994_ = 69.46, p<0.0001). Second, an assessment of published conservation strategies and recommendations (including IUCN Specialist Group Conservation Action Plans, captive breeding protocols and the wider scientific literature listed in the 1978–2005 Zoological Record database) reveals that no species-specific conservation actions have even been suggested for 42 of the top 100 EDGE species. Most of these species are from poorly known regions or taxonomic groups and until now have rarely been highlighted as conservation priorities. Little conservation action is actually being implemented for many other top EDGE species, despite frequent recommendations in the conservation literature. Indeed, the top-scoring EDGE species, the Yangtze River dolphin (*Lipotes vexillifer*), is now possibly the world's most threatened mammal despite two decades of debate over a potential *ex situ* breeding programme, and may number fewer than 13 surviving individuals [49]. The lack of conservation attention for priority EDGE species is a serious problem for mammalian biodiversity and suggests that large amounts of evolutionary history are likely to be lost in the near future. This phenomenon of diversity slipping quietly towards extinction is likely to be much more severe in less charismatic groups than mammals.

The approach described in this paper can be used for conservation in a number of ways. First, conservation managers with limited resources at their disposal typically need to conserve populations of several threatened species. If all other factors were equal, the management of the most evolutionarily distinct species should be prioritized. Second, a list of high-priority species requiring urgent conservation action can be generated easily. In this paper, we have selected the 100 highest-ranking species, but one might equally choose all threatened (Vulnerable and above) species with above average ED. This would result in a list of 521 (using median) or 630 (using geometric mean) ‘EDGE species’ that are both evolutionarily distinct and globally endangered. Third, EDGE scores could also be used to weight species' importance in selecting reserve networks, building on previous studies that have used phylogenetic diversity [50]–[52] or threatened species [11] to identify priority areas for conservation. The statistical properties of EDGE scores (they are both normally-distributed and bounded at zero) make them especially suitable for these kinds of analysis. In this way, the EDGE approach is not an alternative to existing conservation frameworks [e.g. 6] but complements them.

The EDGE approach identifies the species representing most evolutionary history from among those in imminent danger of extinction. Our methods extend the application of PD-based conservation to a wider range of taxa and situations than previous approaches [4], [5], [13], [22], [24], [25]. Future work might incorporate socioeconomic considerations [5], [14] and the fact that a species' value depends also on the extinction risk of its close relatives [53]. However, our results suggest that large numbers of evolutionarily distinct species are inadequately served by existing conservation measures, and that more work is carried out to prevent the imminent loss of large quantities of our evolutionary heritage. It is hoped that this approach will serve to highlight their importance to biodiversity and emphasize the need for urgent conservation action.

## Materials and Methods

### Implementing ED scores for mammals

We used a composite ‘supertree’ phylogeny [31] to calculate ED scores for mammals. The supertree presents several challenges to the estimation of ED when compared with the (unknown) true phylogeny: poor resolution, missing species and uncertainty in node ages. Accordingly, we modified the basic procedure to control for these problems.

Phylogenetic information is poor in many mammalian clades (especially bats and rodents, which together make up >60% of species) and the whole supertree contains only 47% of all possible nodes, many of which are polytomies (nodes with more than two daughter branches). Across the whole phylogeny, ∼40% of species are immediately descended from bifurcations, ∼20% from small polytomies (3–5 daughters), ∼15% from medium-sized polytomies (6–10 daughters) and the remainder from large polytomies with >10 daughters. Polytomies in supertrees result from poor or conflicting data rather than a true representation of the speciation process, so the distinctiveness of branches subtending them is overestimated [54], thus leading to biased ED scores. For example, the common ancestor of species X, Y and Z is believed to be 1 MY old, but the branching pattern within the clade is unknown. The polytomy appears to show that each species represents 1 MY of unique evolutionary history. In reality, the phylogeny is bifurcating, with one species aged 1 MY and the others sharing a more recent common ancestor. The bias induced by polytomies can be corrected by estimating the expected ED of descendant species under an appropriate null model of diversification. We achieved this by applying a scaling factor based on the empirical distribution of ED scores in a randomly generated phylogeny of 5000 species grown under constant rates of speciation (0.1) and extinction (0.08). The mean ED score of species in 819 clades of three species was 0.81 of the clade age; ED scores for nodes of 2–20 species scale according to (branch length) * (1.081–0.267 * ln{d}), where d is the number of descendent branches (n = 2873 clades, r^2^ = 0.69). Quantitatively similar values were obtained in bifurcating clades of primates [1.117–0.246 * ln{d}, n = 78, ref 55] and carnivores [1.139–0.269 * ln{d}, n = 101, ref 56].

The mammal supertree contains 4510 of the 4548 (>99%) extant species listed in Wilson & Reeder [35]. Although few in number, the missing species need to be taken into account because their absence will tend to inflate the ED scores of close relatives. For example, omitting species A from the phylogeny in figure 1 would elevate B from the joint lowest ranking species (with A) to the joint highest-ranking (with C), with an ED score of (2/1+1/2+2/4) = 3.5 MY. The problem is acute in real datasets since missing species tend not to be a random sample: 22 of the 38 missing mammals are from the genus *Sorex*. We account for this problem using a simple correction factor that allocates the missing species among their presumed closest relatives. For example, we correct for the omission of the bare-bellied hedgehog (*Hemiechinus nudiventris*) by treating the other five *Hemiechinus* spp. as 6/5 = 1.2 species, and we correct for the omission of both *Cryptochloris* species by spreading the two missing species evenly between other Chrysochloridae.

Variation among morphological and molecular estimates of divergence times (node ages) can lead to considerable uncertainty in ED scores. To reduce the effects of this uncertainty, we estimated ED using three sets of branch lengths. One set was based on the best (i.e. mean) estimates of node age; the others were derived from the upper and lower 95% confidence intervals around these dates. Species values of ED were calculated as the geometric mean of scores under the three sets of branch lengths. The geometric mean was preferred since it down-weights species whose scores are based on nodes with symmetrical but wide confidence intervals in estimate age, and is therefore more conservative than the arithmetic mean.

### Tests of robustness

To test whether ED scores are comparable among taxonomic groups, we examined how species' ED accumulates as progressively larger clades are considered. If ED scores are truly comparable, their rank order will be independent of the size of the clade considered. We randomly selected one Critically Endangered species from each of ten mammal orders and measured the cumulative ED score at each node between the species and the root of the mammal supertree, thus redefining and enlarging the clade (and so increasing the number of species it contained) at each step.

Taxonomic changes have the potential to dramatically alter the ED scores of individual species. Splitting a species in two reduces the distinctiveness of all branches ancestral to the split, particularly those near the tips. If ED scores are highly sensitive to taxonomic changes then it may be meaningless to apply them in setting conservation priorities. The effects of taxonomic changes on ED scores were therefore investigated in the primates, which have recently experienced considerable taxonomic inflation [27]. We compared primate ED scores under a biological species concept [35: 233 species] and a phylogenetic species concept [36: 358 species]. We employed a single phylogeny [31], but changed the number of species represented by each tip. We calculated the expected ED for multi-species tips by treating them as if they were descended from a polytomy of {n+r+1} descendent branches, where n is the actual number of descendent branches and r is the number of species represented by the tip.

## Supporting Information

Table S1 Evolutionary Distinctiveness and EDGE scores for mammals. This table shows Evolutionary Distinctiveness (ED) and EDGE scores for all species included in the mammal supertree [31] ranked by their EDGE score. Species that could not be assigned EDGE scores are appended to the bottom of the list, sorted by status and ED score. Species taxonomy follows Wilson & Reeder [35]. Red List categories follow the 2006 IUCN Red List [2]: CR = Critically Endangered, EN = Endangered, VU = Vulnerable, NT = Near Threatened, LC = Least Concern, CD = Conservation Dependent, DD = Data Deficient, NE = Not Evaluated. The NE category includes species in Wilson & Reeder [35] that could not be matched with any species or subspecies names in the Red List.
(0.42 MB PDF)

Table S2 Evolutionary Distinctiveness for primates under two species concepts. This table lists ED scores for primates under the biological species concept i[.e. the taxonomy of ref 35], the number of phylogenetic species into which the biological species was split [36] and the estimated ED score of each phylogenetic species. See Materials and Methods for further information. ED scores are lower for phylogenetic species than biological species, even for taxa whose taxonomic status is the same under both concepts (i.e. the number of phylogenetic species is one). This occurs because the total number of species in the phylogeny is greater, so each receives a smaller share of the distinctiveness of ancestral branches. ED scores were calculated using just one set of branch lengths (the ‘best’ set), so differ from those in table S1.
(0.05 MB PDF)
