# Supplementary material for: Mammals on the EDGE: Conservation Priorities Based on Threat and Phylogeny
Source: PLoS One. 2007 Mar 14;2(3):e296. doi: 10.1371/journal.pone.0000296 (PMC1808424; doi:10.1371/journal.pone.0000296)
Supplement: Table S1 — Evolutionary Distinctiveness and EDGE scores for mammals. This table shows Evolutionary Distinctiveness (ED) and EDGE scores for all species included in the mammal supertree [31] ranked by their EDGE score. Species that could not be assigned EDGE scores are appended to the bottom of the list, sorted by status and ED score. Species taxonomy follows Wilson & Reeder [35]. Red List categories follow the 2006 IUCN Red List [2]: CR = Critically Endangered, EN = Endangered, VU = Vulnerable, NT = Near Threatened, LC = Least Concern, CD = Conservation Dependent, DD = Data Deficient, NE = Not Evaluated. The NE category includes species in Wilson & Reeder [35] that could not be matched with any species or subspecies names in the Red List. (0.42 MB PDF) [file pone.0000296.s001.pdf]

Rank	Order	Family	Species	IUCN	ED'	EDGE
1	Cetacea	Platanistidae	Lipotes vexillifer	CR	39.801	6.4813
2	Monotremata	Tachyglossidae	Zaglossus bruijni	EN	71.245	6.3595
3	Lagomorpha	Leporidae	Bunolagus monticularis	CR	31.406	6.2509
4	Insectivora	Solenodontidae	Solenodon cubanus	EN	62.699	6.2336
4	Insectivora	Solenodontidae	Solenodon paradoxus	EN	62.699	6.2336
6	Perissodactyla	Rhinocerotidae	Dicerorhinus sumatrensis	CR	29.082	6.1765
7	Perissodactyla	Rhinocerotidae	Diceros bicornis	CR	27.656	6.1280
8	Artiodactyla	Camelidae	Camelus bactrianus	CR	27.491	6.1222
9	Diprotodontia	Vombatidae	Lasiorhinus krefftii	CR	26.528	6.0878
10	Lagomorpha	Leporidae	Nesolagus netscheri	CR	24.942	6.0284
11	Perissodactyla	Rhinocerotidae	Rhinoceros sondaicus	CR	24.915	6.0274
12	Proboscidea	Elephantidae	Elephas maximus	EN	49.684	6.0050
13	Perissodactyla	Equidae	Equus asinus	CR	24.078	5.9946
14	Perissodactyla	Equidae	Equus onager	CR	23.022	5.9516
15	Chiroptera	Rhinolophidae	Paracoelops megalotis	CR	21.788	5.8988
16	Primates	Daubentoniidae	Daubentonia madagascariensis	EN	43.157	5.8672
17	Rodentia	Myoxidae	Glirulus japonicus	EN	40.134	5.7963
18	Carnivora	Ursidae	Ailuropoda melanoleuca	EN	38.941	5.7669
19	Carnivora	Ursidae	Ailurus fulgens	EN	38.128	5.7463
20	Chiroptera	Molossidae	Otomops wroughtoni	CR	18.496	5.7428
21	Artiodactyla	Hippopotamidae	Hexaprotodon liberiensis	EN	37.155	5.7211
22	Primates	Loridae	Loris tardigradus	EN	36.520	5.7043
23	Primates	Lemuridae	Hapalemur aureus	CR	17.638	5.6978
23	Primates	Lemuridae	Hapalemur simus	CR	17.638	5.6978
25	Chiroptera	Emballonuridae	Coleura seychellensis	CR	17.634	5.6976
26	Didelphimorphia	Didelphidae	Marmosa andersoni	CR	17.618	5.6967
27	Carnivora	Phocidae	Monachus monachus	CR	17.379	5.6838
28	Diprotodontia	Burramyidae	Burramys parvus	EN	35.174	5.6678
29	Primates	Indridae	Propithecus tattersalli	CR	16.735	5.6481
30	Notoryctemorphia	Notoryctidae	Notoryctes caurinus	EN	34.142	5.6388
30	Notoryctemorphia	Notoryctidae	Notoryctes typhlops	EN	34.142	5.6388
32	Rodentia	Capromyidae	Isolobodon portoricensis	CR	16.423	5.6304
33	Chiroptera	Pteropodidae	Aproteles bulmerae	CR	16.392	5.6286
34	Perissodactyla	Tapiridae	Tapirus bairdii	EN	33.776	5.6284
35	Didelphimorphia	Didelphidae	Gracilinanus aceramarcae	CR	16.341	5.6257
36	Primates	Indridae	Indri indri	EN	33.383	5.6170
37	Artiodactyla	Bovidae	Damaliscus hunteri	CR	16.044	5.6084
38	Rodentia	Muridae	Macrotarsomys ingens	CR	16.031	5.6076
39	Chiroptera	Vespertilionidae	Pharotis imogene	CR	15.703	5.5882
40	Insectivora	Talpidae	Talpa streeti	CR	15.629	5.5837
41	Lagomorpha	Leporidae	Romerolagus diazi	EN	31.935	5.5740
42	Microbiotheria	Microbiotheriidae	Dromiciops gliroides	VU	64.351	5.5661
43	Carnivora	Viverridae	Cryptoprocta ferox	EN	31.143	5.5496
44	Lagomorpha	Leporidae	Pentalagus furnessi	EN	31.120	5.5489
45	Insectivora	Erinaceidae	Hylomys hainanensis	EN	30.644	5.5340
46	Macroscelidea	Macroscelididae	Rhynchocyon chrysopygus	EN	30.571	5.5317
47	Insectivora	Erinaceidae	Podogymnura aureospinula	EN	29.485	5.4967
47	Insectivora	Erinaceidae	Podogymnura truei	EN	29.485	5.4967
49	Chiroptera	Craseonycteridae	Craseonycteris thonglongyai	EN	29.372	5.4930
50	Primates	Cheirogaleidae	Allocebus trichotis	EN	29.365	5.4927
51	Rodentia	Muridae	Tokudaia muenninki	CR	14.044	5.4835
52	Insectivora	Talpidae	Euroscaptor parvidens	CR	14.019	5.4819
53	Sirenia	Dugongidae	Dugong dugon	VU	58.982	5.4803
54	Diprotodontia	Petauridae	Gymnobelideus leadbeateri	EN	28.592	5.4670
55	Insectivora	Tenrecidae	Micropotamogale lamottei	EN	28.424	5.4612

Rank	Order	Family	Species	IUCN	ED'	EDGE
56	Chiroptera	Mystacinidae	Mystacina tuberculata	VU	57.572	5.4565
57	Rodentia	Chinchillidae	Chinchilla brevicaudata	CR	13.638	5.4562
58	Insectivora	Soricidae	Chimarrogale hantu	CR	13.637	5.4562
58	Insectivora	Soricidae	Chimarrogale sumatrana	CR	13.637	5.4562
60	Rodentia	Myoxidae	Selevinia betpakdalaensis	EN	27.892	5.4430
61	Insectivora	Soricidae	Soriculus salenskii	CR	13.373	5.4379
62	Artiodactyla	Bovidae	Saiga tatarica	CR	13.247	5.4292
63	Xenarthra	Bradypodidae	Bradypus torquatus	EN	27.307	5.4226
64	Rodentia	Dipodidae	Allactaga firouzi	CR	12.964	5.4091
65	Cetacea	Platanistidae	Platanista gangetica	EN	26.554	5.3956
65	Cetacea	Platanistidae	Platanista minor	EN	26.554	5.3956
67	Artiodactyla	Tayassuidae	Catagonus wagneri	EN	26.533	5.3948
68	Insectivora	Talpidae	Nesoscaptor uchidai	EN	26.217	5.3833
69	Didelphimorphia	Didelphidae	Marmosops handleyi	CR	12.585	5.3816
70	Diprotodontia	Potoroidae	Potorous longipes	EN	25.895	5.3714
71	Dermoptera	Cynocephalidae	Cynocephalus volans	VU	52.120	5.3588
72	Insectivora	Talpidae	Uropsilus investigator	EN	25.261	5.3475
72	Insectivora	Talpidae	Uropsilus soricipes	EN	25.261	5.3475
74	Perissodactyla	Rhinocerotidae	Rhinoceros unicornis	EN	24.915	5.3343
75	Rodentia	Dipodidae	Sicista armenica	CR	11.769	5.3196
76	Rodentia	Muridae	Typhlomys chapensis	CR	11.764	5.3192
77	Proboscidea	Elephantidae	Loxodonta africana	VU	49.684	5.3119
78	Cetacea	Phocoenidae	Phocoena sinus	CR	11.641	5.3096
79	Primates	Cebidae	Lagothrix flavicauda	CR	11.452	5.2945
80	Perissodactyla	Tapiridae	Tapirus pinchaque	EN	23.833	5.2916
81	Rodentia	Dipodidae	Euchoreutes naso	EN	23.591	5.2818
82	Perissodactyla	Equidae	Equus grevyi	EN	23.439	5.2756
82	Perissodactyla	Equidae	Equus zebra	EN	23.439	5.2756
84	Sirenia	Trichechidae	Trichechus inunguis	VU	47.738	5.2728
85	Chiroptera	Vespertilionidae	Murina grisea	CR	11.176	5.2720
86	Rodentia	Myoxidae	Dryomys sichuanensis	EN	23.144	5.2635
87	Chiroptera	Vespertilionidae	Tomopeas ravus	VU	47.124	5.2601
88	Cetacea	Balaenopteridae	Balaenoptera musculus	EN	22.566	5.2392
88	Cetacea	Balaenopteridae	Balaenoptera physalus	EN	22.566	5.2392
90	Carnivora	Viverridae	Eupleres goudotii	EN	22.428	5.2334
91	Rodentia	Muridae	Dendromus kahuziensis	CR	10.676	5.2301
92	Didelphimorphia	Didelphidae	Glironia venusta	VU	45.620	5.2283
93	Chiroptera	Molossidae	Chaerephon gallagheri	CR	10.569	5.2209
94	Chiroptera	Myzopodidae	Myzopoda aurita	VU	45.098	5.2171
95	Rodentia	Muridae	Hypogeomys antimena	EN	22.043	5.2168
96	Chiroptera	Rhinolophidae	Rhinolophus imaizumii	EN	21.957	5.2131
97	Primates	Hominidae	Pongo pygmaeus	EN	21.655	5.1998
98	Rodentia	Muridae	Tylomys bullaris	CR	10.311	5.1984
98	Rodentia	Muridae	Tylomys tumbalensis	CR	10.311	5.1984
100	Rodentia	Myoxidae	Myomimus setzeri	EN	21.495	5.1927
101	Rodentia	Capromyidae	Mesocapromys angelcabrerai	CR	9.976	5.1683
101	Rodentia	Capromyidae	Mesocapromys auritus	CR	9.976	5.1683
101	Rodentia	Capromyidae	Mesocapromys nanus	CR	9.976	5.1683
101	Rodentia	Capromyidae	Mesocapromys sanfelipensis	CR	9.976	5.1683
105	Artiodactyla	Bovidae	Pantholops hodgsonii	EN	20.717	5.1575
106	Primates	Cebidae	Ateles fusciceps	CR	9.838	5.1557
107	Sirenia	Trichechidae	Trichechus manatus	VU	42.117	5.1502
107	Sirenia	Trichechidae	Trichechus senegalensis	VU	42.117	5.1502
109	Cetacea	Physeteridae	Physeter catodon	VU	41.881	5.1447

Rank	Order	Family	Species	IUCN	ED'	EDGE
110	Chiroptera	Pteropodidae	Pteralopex acrodonta	CR	9.521	5.1259
110	Chiroptera	Pteropodidae	Pteralopex anceps	CR	9.521	5.1259
110	Chiroptera	Pteropodidae	Pteralopex atrata	CR	9.521	5.1259
110	Chiroptera	Pteropodidae	Pteralopex pulchra	CR	9.521	5.1259
114	Rodentia	Muridae	Macruromys elegans	CR	9.465	5.1206
115	Primates	Lemuridae	Varecia variegata	EN	19.781	5.1135
116	Chiroptera	Emballonuridae	Balantiopteryx infusca	EN	19.570	5.1033
117	Insectivora	Tenrecidae	Limnogale mergulus	VU	40.058	5.1013
118	Insectivora	Chrysochloridae	Chrysospalax trevelyani	EN	19.396	5.0948
119	Artiodactyla	Bovidae	Addax nasomaculatus	CR	9.121	5.0872
120	Lagomorpha	Ochotonidae	Ochotona pusilla	VU	39.247	5.0813
121	Rodentia	Muridae	Zyzomys palatilis	CR	9.061	5.0813
121	Rodentia	Muridae	Zyzomys pedunculatus	CR	9.061	5.0813
123	Insectivora	Chrysochloridae	Amblysomus gunningi	EN	18.981	5.0742
123	Insectivora	Chrysochloridae	Amblysomus julianae	EN	18.981	5.0742
125	Cetacea	Balaenopteridae	Balaenoptera borealis	EN	18.918	5.0711
126	Perissodactyla	Tapiridae	Tapirus indicus	VU	38.821	5.0707
127	Chiroptera	Emballonuridae	Emballonura semicaudata	EN	18.754	5.0628
128	Rodentia	Muridae	Pogonomelomys bruijni	CR	8.794	5.0543
129	Rodentia	Muridae	Mallomys gunung	CR	8.778	5.0527
130	Peramelemorphia	Peramelidae	Macrotis lagotis	VU	38.070	5.0517
131	Rodentia	Capromyidae	Mysateles garridoi	CR	8.726	5.0473
132	Rodentia	Muridae	Pseudohydromys murinus	CR	8.702	5.0449
133	Carnivora	Felidae	Lynx pardinus	CR	8.696	5.0443
134	Artiodactyla	Suidae	Sus cebifrons	CR	8.642	5.0388
134	Artiodactyla	Suidae	Sus salvanius	CR	8.642	5.0388
136	Carnivora	Viverridae	Viverra civettina	CR	8.624	5.0368
137	Artiodactyla	Hippopotamidae	Hippopotamus amphibius	VU	37.155	5.0280
138	Carnivora	Viverridae	Cynogale bennettii	EN	17.731	5.0096
139	Artiodactyla	Bovidae	Cephalophus adersi	CR	8.326	5.0054
140	Cetacea	Platanistidae	Inia geoffrensis	VU	36.295	5.0052
141	Didelphimorphia	Didelphidae	Marmosa xerophila	EN	17.618	5.0036
142	Rodentia	Muridae	Crateromys paulus	CR	8.255	4.9978
143	Rodentia	Muridae	Uromys rex	CR	8.232	4.9953
144	Carnivora	Phocidae	Monachus schauinslandi	EN	17.379	4.9907
145	Chiroptera	Rhinolophidae	Anthops ornatus	VU	35.667	4.9882
146	Artiodactyla	Bovidae	Bos sauveli	CR	8.120	4.9831
147	Rodentia	Octodontidae	Tympanoctomys barrerae	VU	35.240	4.9764
148	Rodentia	Muridae	Delanymys brooksi	EN	17.068	4.9736
149	Primates	Cercopithecidae	Procolobus rufomitratus	CR	8.029	4.9730
150	Chiroptera	Molossidae	Mormopterus phrudus	EN	17.037	4.9719
151	Rodentia	Dinomyidae	Dinomys branickii	EN	16.945	4.9667
152	Rodentia	Muridae	Leptomys elegans	CR	7.958	4.9651
152	Rodentia	Muridae	Leptomys signatus	CR	7.958	4.9651
154	Insectivora	Soricidae	Myosorex eisentrauti	CR	7.879	4.9563
154	Insectivora	Soricidae	Myosorex rumpii	CR	7.879	4.9563
156	Primates	Indridae	Propithecus diadema	EN	16.735	4.9550
157	Rodentia	Heteromyidae	Heteromys nelsoni	CR	7.856	4.9537
158	Paucituberculata	Caenolestidae	Rhyncholestes raphanurus	VU	34.420	4.9536
159	Lagomorpha	Leporidae	Caprolagus hispidus	EN	16.587	4.9466
160	Diprotodontia	Petauridae	Dactylopsila tatei	EN	16.502	4.9418
161	Artiodactyla	Bovidae	Procapra przewalskii	CR	7.747	4.9414
162	Chiroptera	Vespertilionidae	Pipistrellus anthonyi	CR	7.744	4.9409
162	Chiroptera	Vespertilionidae	Pipistrellus joffrei	CR	7.744	4.9409
164	Didelphimorphia	Didelphidae	Lestodelphys halli	VU	33.749	4.9345
165	Primates	Hominidae	Gorilla gorilla	EN	16.374	4.9344

Rank	Order	Family	Species	IUCN	ED'	EDGE
166	Cetacea	Balaenidae	Eubalaena glacialis	EN	16.242	4.9268
167	Chiroptera	Furipteridae	Amorphochilus schnablii	VU	33.242	4.9197
168	Rodentia	Muridae	Eliurus penicillatus	CR	7.541	4.9175
169	Rodentia	Muridae	Pelomys isseli	EN	15.989	4.9120
170	Dasyuromorphia	Myrmecobiidae	Myrmecobius fasciatus	VU	32.857	4.9084
171	Carnivora	Herpestidae	Mungotictis decemlineata	EN	15.921	4.9080
172	Lagomorpha	Leporidae	Sylvilagus insonus	CR	7.443	4.9060
173	Rodentia	Sciuridae	Biswamoyopterus biswasi	CR	7.425	4.9038
174	Artiodactyla	Bovidae	Bubalus mindorensis	CR	7.373	4.8976
175	Diprotodontia	Phalangeridae	Spilocuscus rufoniger	EN	15.589	4.8882
176	Rodentia	Sciuridae	Paraxerus vincenti	CR	7.180	4.8743
177	Cetacea	Delphinidae	Orcaella brevirostris	CR	7.143	4.8698
178	Carnivora	Ursidae	Tremarctos ornatus	VU	31.003	4.8521
179	Carnivora	Herpestidae	Liberiictis kuhni	EN	14.991	4.8515
180	Chiroptera	Rhinolophidae	Hipposideros lamottei	CR	6.951	4.8459
181	Rodentia	Sciuridae	Hylopetes winstoni	CR	6.932	4.8435
182	Primates	Cercopithecidae	Pygathrix avunculus	CR	6.913	4.8410
183	Macroscelidea	Macroscelididae	Rhynchocyon petersi	VU	30.571	4.8385
184	Carnivora	Herpestidae	Galidictis grandidieri	EN	14.635	4.8290
185	Carnivora	Canidae	Urocyon littoralis	CR	6.779	4.8240
186	Artiodactyla	Suidae	Babyrousa babyrussa	VU	30.067	4.8224
187	Lagomorpha	Ochotonidae	Ochotona koslowi	EN	14.448	4.8169
188	Diprotodontia	Phalangeridae	Phalanger matanim	EN	14.416	4.8148
189	Rodentia	Geomyidae	Zygogeomys trichopus	EN	14.390	4.8131
190	Chiroptera	Vespertilionidae	Miniopterus robustior	EN	14.295	4.8070
191	Primates	Cebidae	Brachyteles arachnoides	EN	14.057	4.7913
192	Rodentia	Muridae	Tokudaia osimensis	EN	14.044	4.7904
193	Dasyuromorphia	Dasyuridae	Sminthopsis aitkeni	EN	14.037	4.7899
194	Rodentia	Aplodontidae	Aplodontia rufa	NT	58.497	4.7791
195	Primates	Cheirogaleidae	Microcebus coquereli	VU	28.710	4.7778
196	Rodentia	Geomyidae	Pappogeomys neglectus	CR	6.383	4.7717
197	Insectivora	Talpidae	Mogera etigo	EN	13.732	4.7695
197	Insectivora	Talpidae	Mogera tokudae	EN	13.732	4.7695
199	Insectivora	Soricidae	Chimarrogale phaeura	EN	13.637	4.7630
200	Primates	Loridae	Nycticebus pygmaeus	VU	27.985	4.7531
201	Artiodactyla	Bovidae	Tragelaphus buxtoni	EN	13.436	4.7492
202	Peramelemorphia	Peramelidae	Perameles bougainville	EN	13.316	4.7408
203	Xenarthra	Dasypodidae	Priodontes maximus	VU	27.615	4.7402
204	Primates	Hylobatidae	Hylobates moloch	CR	6.104	4.7333
205	Scandentia	Tupaiidae	Tupaia longipes	EN	13.202	4.7328
205	Scandentia	Tupaiidae	Tupaia nicobarica	EN	13.202	4.7328
207	Dasyuromorphia	Dasyuridae	Sminthopsis psammophila	EN	13.168	4.7304
208	Insectivora	Talpidae	Desmana moschata	VU	27.327	4.7301
208	Insectivora	Talpidae	Galemys pyrenaicus	VU	27.327	4.7301
210	Hyracoidea	Procaviidae	Heterohyrax antineae	VU	27.319	4.7298
211	Chiroptera	Pteropodidae	Nyctimene rabori	CR	6.064	4.7277
212	Didelphimorphia	Didelphidae	Caluromysiops irrupta	VU	27.139	4.7235
213	Rodentia	Heteromyidae	Dipodomys insularis	CR	6.020	4.7213
214	Chiroptera	Megadermatidae	Macroderma gigas	VU	26.864	4.7136
215	Rodentia	Muridae	Mystromys albicaudatus	EN	12.768	4.7018
216	Didelphimorphia	Didelphidae	Marmosops cracens	EN	12.585	4.6884
217	Hyracoidea	Procaviidae	Dendrohyrax validus	VU	26.004	4.6823
218	Chiroptera	Molossidae	Eumops maurus	VU	25.994	4.6819
219	Didelphimorphia	Didelphidae	Monodelphis kunsi	EN	12.337	4.6700
220	Rodentia	Muridae	Meriones chengi	CR	5.644	4.6663
221	Rodentia	Muridae	Mesocricetus auratus	EN	12.281	4.6658

Rank	Order	Family	Species	IUCN	ED'	EDGE
222	Chiroptera	Pteropodidae	Latidens salimalii	EN	12.228	4.6618
223	Chiroptera	Phyllostomidae	Phyllonycteris aphylla	EN	12.201	4.6597
224	Rodentia	Myoxidae	Eliomys quercinus	VU	25.014	4.6449
225	Carnivora	Procyonidae	Bassaricyon lasius	EN	11.965	4.6417
225	Carnivora	Procyonidae	Bassaricyon pauli	EN	11.965	4.6417
227	Rodentia	Muridae	Pseudomys fieldi	CR	5.469	4.6396
227	Rodentia	Muridae	Pseudomys glaucus	CR	5.469	4.6396
229	Rodentia	Heteromyidae	Dipodomys ingens	CR	5.446	4.6361
230	Primates	Callitrichidae	Leontopithecus caissara	CR	5.394	4.6279
230	Primates	Callitrichidae	Leontopithecus chrysopygus	CR	5.394	4.6279
232	Rodentia	Dipodidae	Sicista caudata	EN	11.769	4.6264
233	Chiroptera	Phyllostomidae	Leptonycteris nivalis	EN	11.644	4.6166
234	Perissodactyla	Equidae	Equus hemionus	VU	24.078	4.6083
235	Chiroptera	Vespertilionidae	Scotophilus borbonicus	CR	5.269	4.6081
236	Artiodactyla	Cervidae	Elaphurus davidianus	CR	5.259	4.6067
237	Primates	Hominidae	Pan paniscus	EN	11.451	4.6013
237	Primates	Hominidae	Pan troglodytes	EN	11.451	4.6013
239	Carnivora	Herpestidae	Herpestes palustris	EN	11.432	4.5997
240	Perissodactyla	Tapiridae	Tapirus terrestris	VU	23.833	4.5985
241	Insectivora	Soricidae	Cryptotis endersi	EN	11.355	4.5935
242	Monotremata	Ornithorhynchidae	Ornithorhynchus anatinus	LC	97.603	4.5911
243	Artiodactyla	Bovidae	Capra walie	CR	5.129	4.5856
244	Rodentia	Muridae	Anotomys leander	EN	11.258	4.5856
245	Diprotodontia	Pseudocheiridae	Pseudochirops corinnae	VU	22.811	4.5564
246	Didelphimorphia	Didelphidae	Gracilinanus dryas	VU	22.736	4.5533
247	Rodentia	Sciuridae	Hylopetes sipora	EN	10.856	4.5523
248	Rodentia	Geomyidae	Orthogeomys cuniculus	CR	4.917	4.5503
249	Tubulidentata	Orycteropodidae	Orycteropus afer	LC	93.318	4.5467
250	Cetacea	Balaenopteridae	Megaptera novaeangliae	VU	22.566	4.5461
251	Primates	Hylobatidae	Hylobates hoolock	EN	10.761	4.5442
252	Primates	Cercopithecidae	Pygathrix nemaeus	EN	10.729	4.5415
253	Chiroptera	Pteropodidae	Pteropus insularis	CR	4.863	4.5413
253	Chiroptera	Pteropodidae	Pteropus phaeocephalus	CR	4.863	4.5413
253	Chiroptera	Pteropodidae	Pteropus pselaphon	CR	4.863	4.5413
256	Chiroptera	Pteropodidae	Notopteris macdonaldi	VU	22.434	4.5405
257	Carnivora	Viverridae	Fossa fossana	VU	22.428	4.5402
258	Rodentia	Muridae	Melomys rubicola	CR	4.846	4.5383
259	Rodentia	Dipodidae	Cardiocranius paradoxus	VU	22.381	4.5382
260	Chiroptera	Pteropodidae	Pteropus aldabrensis	CR	4.813	4.5327
261	Chiroptera	Natalidae	Natalus tumidifrons	VU	22.069	4.5248
262	Diprotodontia	Macropodidae	Lagostrophus fasciatus	VU	22.019	4.5226
263	Rodentia	Muridae	Nectomys parvipes	CR	4.752	4.5221
264	Insectivora	Soricidae	Suncus ater	CR	4.733	4.5189
264	Insectivora	Soricidae	Suncus mertensi	CR	4.733	4.5189
266	Chiroptera	Rhinolophidae	Rhinonicteris aurantia	VU	21.869	4.5161
267	Rodentia	Myoxidae	Myomimus personatus	VU	21.495	4.4996
267	Rodentia	Myoxidae	Myomimus roachi	VU	21.495	4.4996
269	Rodentia	Muridae	Leporillus conditor	EN	10.196	4.4950
270	Carnivora	Felidae	Catopuma badia	EN	10.169	4.4926
271	Chiroptera	Emballonuridae	Diclidurus ingens	VU	21.217	4.4872
272	Cetacea	Monodontidae	Delphinapterus leucas	VU	21.041	4.4792
273	Rodentia	Sciuridae	Callosciurus quinquestriatus	VU	20.999	4.4773
274	Rodentia	Sciuridae	Eupetaurus cinereus	EN	9.998	4.4772
275	Dasyuromorphia	Dasyuridae	Parantechinus apicalis	EN	9.987	4.4761
276	Rodentia	Sciuridae	Sciurotamias forresti	VU	20.903	4.4729
277	Scandentia	Tupaiidae	Urogale everetti	VU	20.884	4.4720

Rank	Order	Family	Species	IUCN	ED'	EDGE
278	Chiroptera	Pteropodidae	Pteropus molossinus	CR	4.445	4.4673
278	Chiroptera	Pteropodidae	Pteropus rodricensis	CR	4.445	4.4673
280	Chiroptera	Vespertilionidae	Rhogeessa alleni	EN	9.788	4.4579
281	Diprotodontia	Petauridae	Petaurus gracilis	EN	9.767	4.4559
282	Rodentia	Muridae	Microtus evoronensis	CR	4.371	4.4535
282	Rodentia	Muridae	Microtus mujanensis	CR	4.371	4.4535
284	Rodentia	Castoridae	Castor fiber	NT	41.879	4.4515
285	Rodentia	Muridae	Habromys simulatus	EN	9.684	4.4482
286	Rodentia	Muridae	Lamottemys okuensis	EN	9.682	4.4480
287	Diprotodontia	Phascolarctidae	Phascolarctos cinereus	NT	41.655	4.4463
288	Rodentia	Muridae	Melasmothrix naso	EN	9.641	4.4441
289	Diprotodontia	Macropodidae	Dendrolagus goodfellowi	EN	9.630	4.4431
290	Artiodactyla	Bovidae	Pseudois schaeferi	EN	9.570	4.4374
291	Chiroptera	Vespertilionidae	Eptesicus floweri	VU	20.112	4.4361
292	Xenarthra	Dasypodidae	Tolypeutes tricinctus	VU	20.029	4.4322
293	Chiroptera	Pteropodidae	Pteropus livingstonii	CR	4.254	4.4316
294	Chiroptera	Emballonuridae	Saccolaimus mixtus	VU	19.942	4.4281
295	Rodentia	Muridae	Coccymys albidens	EN	9.465	4.4274
295	Rodentia	Muridae	Macruromys major	EN	9.465	4.4274
297	Rodentia	Myoxidae	Muscardinus avellanarius	NT	40.694	4.4235
298	Primates	Lemuridae	Lemur catta	VU	19.781	4.4203
299	Carnivora	Felidae	Acinonyx jubatus	VU	19.693	4.4161
300	Chiroptera	Rhinolophidae	Triaenops furculus	VU	19.640	4.4135
301	Rodentia	Myoxidae	Myoxus glis	NT	40.134	4.4100
302	Dasyuromorphia	Dasyuridae	Phascogale calura	EN	9.250	4.4068
303	Carnivora	Otariidae	Eumetopias jubatus	EN	9.230	4.4048
304	Chiroptera	Emballonuridae	Taphozous hildegardeae	VU	19.452	4.4044
305	Chiroptera	Rhinolophidae	Hipposideros nequam	CR	4.100	4.4018
306	Insectivora	Chrysochloridae	Chrysospalax villosus	VU	19.396	4.4016
307	Rodentia	Muridae	Sigmodontomys aphrastus	CR	4.084	4.3987
308	Scandentia	Tupaiidae	Dendrogale melanura	VU	19.299	4.3969
309	Artiodactyla	Bovidae	Bos javanicus	EN	9.115	4.3935
310	Rodentia	Muridae	Rattus enganus	CR	4.036	4.3893
310	Rodentia	Muridae	Rattus montanus	CR	4.036	4.3893
312	Chiroptera	Pteropodidae	Myonycteris brachycephala	EN	9.043	4.3863
313	Chiroptera	Emballonuridae	Saccopteryx gymnura	VU	19.001	4.3821
314	Dasyuromorphia	Dasyuridae	Sminthopsis douglasi	EN	8.942	4.3762
315	Chiroptera	Emballonuridae	Emballonura atrata	VU	18.754	4.3696
315	Chiroptera	Emballonuridae	Emballonura dianae	VU	18.754	4.3696
315	Chiroptera	Emballonuridae	Emballonura furax	VU	18.754	4.3696
318	Artiodactyla	Bovidae	Gazella dama	CR	3.925	4.3669
319	Chiroptera	Phyllostomidae	Platalina genovensium	VU	18.639	4.3638
320	Primates	Cheirogaleidae	Phaner furcifer	NT	38.277	4.3638
321	Chiroptera	Emballonuridae	Taphozous kapalgensis	VU	18.528	4.3582
322	Insectivora	Soricidae	Sylvisorex isabellae	EN	8.763	4.3580
323	Chiroptera	Molossidae	Otomops formosus	VU	18.496	4.3565
323	Chiroptera	Molossidae	Otomops papuensis	VU	18.496	4.3565
323	Chiroptera	Molossidae	Otomops secundus	VU	18.496	4.3565
326	Carnivora	Felidae	Uncia uncia	EN	8.734	4.3550
327	Rodentia	Muridae	Solomys ponceleti	EN	8.716	4.3532
328	Rodentia	Muridae	Gymnuromys roberti	VU	18.432	4.3532
329	Cetacea	Delphinidae	Cephalorhynchus hectori	EN	8.707	4.3523
330	Diprotodontia	Macropodidae	Dendrolagus scottae	EN	8.657	4.3472
331	Artiodactyla	Suidae	Sus verrucosus	EN	8.642	4.3456
332	Carnivora	Felidae	Panthera tigris	EN	8.633	4.3446
333	Chiroptera	Mormoopidae	Pteronotus macleayii	VU	18.243	4.3434

Rank	Order	Family	Species	IUCN	ED'	EDGE
334	Diprotodontia	Macropodidae	Dorcopsis atrata	EN	8.594	4.3406
335	Lagomorpha	Leporidae	Sylvilagus graysoni	EN	8.549	4.3359
336	Diprotodontia	Macropodidae	Petrogale persephone	EN	8.535	4.3344
337	Chiroptera	Vespertilionidae	Myotis planiceps	CR	3.751	4.3309
338	Didelphimorphia	Didelphidae	Caluromys derbianus	VU	17.932	4.3272
339	Carnivora	Ursidae	Melursus ursinus	VU	17.834	4.3219
340	Carnivora	Viverridae	Macrogalidia musschenbroekii	VU	17.797	4.3200
341	Rodentia	Muridae	Ellobius alaicus	EN	8.390	4.3191
342	Rodentia	Sciuridae	Trogopterus xanthipes	EN	8.385	4.3186
343	Carnivora	Ursidae	Ursus thibetanus	VU	17.767	4.3184
344	Rodentia	Muridae	Bunomys coelestis	EN	8.375	4.3175
344	Rodentia	Muridae	Bunomys prolatus	EN	8.375	4.3175
346	Diprotodontia	Macropodidae	Dendrolagus matschiei	EN	8.294	4.3088
347	Rodentia	Muridae	Crateromys australis	EN	8.255	4.3046
348	Rodentia	Muridae	Millardia kondana	EN	8.254	4.3045
349	Primates	Hylobatidae	Hylobates concolor	EN	8.253	4.3043
350	Artiodactyla	Cervidae	Axis calamianensis	EN	8.228	4.3017
350	Artiodactyla	Cervidae	Axis kuhlii	EN	8.228	4.3017
352	Rodentia	Muridae	Reithrodontomys spectabilis	EN	8.182	4.2966
353	Insectivora	Chrysochloridae	Chlorotalpa duthieae	VU	17.363	4.2966
354	Carnivora	Mustelidae	Gulo gulo	VU	17.348	4.2958
355	Chiroptera	Vespertilionidae	Myotis stalkeri	EN	8.147	4.2929
356	Rodentia	Muridae	Mus famulus	EN	8.064	4.2838
357	Rodentia	Muridae	Hybomys basilii	EN	8.046	4.2817
357	Rodentia	Muridae	Hybomys eisentrauti	EN	8.046	4.2817
359	Monotremata	Tachyglossidae	Tachyglossus aculeatus	LC	71.245	4.2801
360	Chiroptera	Molossidae	Mormopterus acetabulosus	VU	17.037	4.2787
360	Chiroptera	Molossidae	Mormopterus doriae	VU	17.037	4.2787
360	Chiroptera	Molossidae	Mormopterus jugularis	VU	17.037	4.2787
360	Chiroptera	Molossidae	Mormopterus kalinowskii	VU	17.037	4.2787
360	Chiroptera	Molossidae	Mormopterus minutus	VU	17.037	4.2787
365	Chiroptera	Phyllostomidae	Micronycteris pusilla	VU	17.033	4.2785
366	Rodentia	Muridae	Stenomys vandeuseni	EN	8.016	4.2784
367	Artiodactyla	Bovidae	Bison bonasus	EN	8.000	4.2767
368	Insectivora	Erinaceidae	Mesechinus hughi	VU	16.985	4.2758
369	Chiroptera	Vespertilionidae	Barbastella barbastellus	VU	16.951	4.2739
370	Rodentia	Abrocomidae	Abrocoma boliviensis	VU	16.945	4.2736
371	Insectivora	Soricidae	Feroculus feroculus	EN	7.957	4.2719
371	Insectivora	Soricidae	Solisorex pearsoni	EN	7.957	4.2719
373	Primates	Cebidae	Ateles marginatus	EN	7.948	4.2709
374	Rodentia	Sciuridae	Sundasciurus juvencus	EN	7.929	4.2687
375	Primates	Cercopithecidae	Nasalis concolor	EN	7.882	4.2635
375	Primates	Cercopithecidae	Nasalis larvatus	EN	7.882	4.2635
377	Insectivora	Soricidae	Myosorex okuensis	EN	7.879	4.2632
378	Primates	Indridae	Propithecus verreauxi	VU	16.735	4.2618
379	Diprotodontia	Macropodidae	Onychogalea fraenata	EN	7.866	4.2617
380	Rodentia	Muridae	Grammomys gigas	EN	7.819	4.2563
381	Rodentia	Muridae	Chiropodomys karlkoopmani	EN	7.813	4.2557
382	Artiodactyla	Moschidae	Moschus moschiferus	VU	16.580	4.2531
383	Xenarthra	Dasypodidae	Chlamyphorus retusus	NT	34.032	4.2494
383	Xenarthra	Dasypodidae	Chlamyphorus truncatus	NT	34.032	4.2494
385	Chiroptera	Phyllostomidae	Sturnira nana	VU	16.506	4.2488
386	Diprotodontia	Petauridae	Dactylopsila megalura	VU	16.502	4.2486
387	Carnivora	Felidae	Oreailurus jacobita	EN	7.750	4.2485
388	Chiroptera	Molossidae	Molossops aequatorianus	VU	16.477	4.2472

Rank	Order	Family	Species	IUCN	ED'	EDGE
389	Rodentia	Muridae	Neusticomys mussoi	EN	7.721	4.2452
389	Rodentia	Muridae	Neusticomys oyapocki	EN	7.721	4.2452
389	Rodentia	Muridae	Neusticomys peruviensis	EN	7.721	4.2452
389	Rodentia	Muridae	Neusticomys venezuelae	EN	7.721	4.2452
393	Rodentia	Capromyidae	Plagiodontia aedium	VU	16.423	4.2441
394	Chiroptera	Rhinolophidae	Hipposideros turpis	EN	7.672	4.2395
395	Didelphimorphia	Didelphidae	Gracilinanus emiliae	VU	16.341	4.2394
396	Lagomorpha	Leporidae	Sylvilagus dicei	EN	7.557	4.2262
397	Primates	Galagonidae	Galagoides zanzibaricus	NT	33.185	4.2249
398	Rodentia	Muridae	Eliurus majori	EN	7.541	4.2243
399	Rodentia	Sciuridae	Callosciurus pygerythrus	VU	16.078	4.2241
400	Rodentia	Muridae	Leopoldamys neilli	EN	7.537	4.2238
401	Chiroptera	Phyllostomidae	Macrotus californicus	VU	16.058	4.2229
402	Rodentia	Sciuridae	Hyosciurus heinrichi	VU	16.033	4.2214
402	Rodentia	Sciuridae	Hyosciurus ileile	VU	16.033	4.2214
404	Rodentia	Muridae	Pelomys hopkinsi	VU	15.989	4.2189
405	Chiroptera	Pteropodidae	Scotonycteris ophiodon	EN	7.483	4.2175
406	Xenarthra	Myrmecophagidae	Myrmecophaga tridactyla	NT	32.896	4.2164
407	Rodentia	Muridae	Praomys hartwigi	EN	7.473	4.2164
408	Carnivora	Herpestidae	Galidia elegans	VU	15.921	4.2149
408	Carnivora	Herpestidae	Salanoia concolor	VU	15.921	4.2149
410	Carnivora	Mustelidae	Mustela felipei	EN	7.419	4.2099
411	Carnivora	Canidae	Canis rufus	CR	3.194	4.2063
412	Artiodactyla	Bovidae	Tetracerus quadricornis	VU	15.767	4.2057
413	Artiodactyla	Bovidae	Bubalus bubalis	EN	7.373	4.2044
413	Artiodactyla	Bovidae	Bubalus depressicornis	EN	7.373	4.2044
413	Artiodactyla	Bovidae	Bubalus quarlesi	EN	7.373	4.2044
416	Insectivora	Soricidae	Sorex sadonis	EN	7.286	4.1940
417	Chiroptera	Nycteridae	Nycteris javanica	VU	15.554	4.1929
418	Rodentia	Muridae	Scolomys melanops	EN	7.250	4.1896
418	Rodentia	Muridae	Scolomys ucayalensis	EN	7.250	4.1896
420	Primates	Cercopithecidae	Procolobus badius	EN	7.238	4.1882
420	Primates	Cercopithecidae	Procolobus preussi	EN	7.238	4.1882
422	Chiroptera	Vespertilionidae	Myotis cobanensis	CR	3.083	4.1793
423	Rodentia	Muridae	Calomyscus hotsoni	EN	7.101	4.1714
424	Rodentia	Muridae	Maxomys alticola	EN	7.048	4.1649
424	Rodentia	Muridae	Maxomys baeodon	EN	7.048	4.1649
424	Rodentia	Muridae	Maxomys wattsi	EN	7.048	4.1649
427	Chiroptera	Phyllostomidae	Lonchorhina fernandezi	VU	15.063	4.1628
427	Chiroptera	Phyllostomidae	Lonchorhina marinkellei	VU	15.063	4.1628
429	Chiroptera	Vespertilionidae	Chalinolobus tuberculatus	VU	15.045	4.1617
430	Rodentia	Muridae	Dicrostonyx vinogradovi	CR	2.991	4.1567
431	Artiodactyla	Bovidae	Ammotragus lervia	VU	14.894	4.1523
432	Insectivora	Erinaceidae	Hylomys sinensis	NT	30.644	4.1477
433	Chiroptera	Vespertilionidae	Pipistrellus endoi	EN	6.905	4.1469
434	Chiroptera	Pteropodidae	Melonycteris aurantius	VU	14.800	4.1463
435	Macroscelidea	Macroscelididae	Rhynchocyon cirnei	NT	30.571	4.1454
436	Chiroptera	Mormoopidae	Mormoops blainvillii	NT	30.474	4.1423
437	Carnivora	Herpestidae	Galidictis fasciata	VU	14.635	4.1358
438	Diprotodontia	Macropodidae	Setonix brachyurus	VU	14.601	4.1336
439	Primates	Cercopithecidae	Cercopithecus diana	EN	6.783	4.1313
440	Chiroptera	Vespertilionidae	Rhogeessa mira	EN	6.776	4.1304
441	Artiodactyla	Bovidae	Oryx leucoryx	EN	6.773	4.1301
442	Primates	Indridae	Avahi laniger	NT	29.966	4.1260
443	Xenarthra	Dasypodidae	Chaetophractus nationi	VU	14.479	4.1258

Rank	Order	Family	Species	IUCN	ED'	EDGE
444	Carnivora	Viverridae	Chrotogale owstoni	VU	14.473	4.1254
444	Carnivora	Viverridae	Diplogale hosei	VU	14.473	4.1254
446	Carnivora	Mustelidae	Enhydra lutris	EN	6.722	4.1235
447	Chiroptera	Vespertilionidae	Glischropus javanus	EN	6.697	4.1202
448	Rodentia	Muridae	Acomys cilicicus	CR	2.843	4.1189
449	Carnivora	Felidae	Prionailurus planiceps	VU	14.311	4.1149
450	Rodentia	Muridae	Sundamys maxi	EN	6.639	4.1127
451	Chiroptera	Phyllostomidae	Diphylla ecaudata	NT	29.513	4.1113
452	Primates	Cercopithecidae	Macaca assamensis	EN	6.580	4.1049
453	Cetacea	Balaenopteridae	Balaenoptera acutorostrata	NT	29.255	4.1028
454	Chiroptera	Emballonuridae	Cyttarops alecto	NT	29.219	4.1016
455	Artiodactyla	Bovidae	Budorcas taxicolor	VU	14.095	4.1007
456	Primates	Cercopithecidae	Macaca silenus	EN	6.547	4.1005
457	Rodentia	Sciuridae	Ratufa macroura	VU	14.072	4.0991
458	Primates	Cercopithecidae	Macaca maura	EN	6.534	4.0989
459	Rodentia	Pedetidae	Pedetes capensis	LC	59.232	4.0982
460	Rodentia	Dasyproctidae	Dasyprocta coibae	EN	6.529	4.0982
460	Rodentia	Dasyproctidae	Dasyprocta ruatanica	EN	6.529	4.0982
462	Insectivora	Talpidae	Euroscaptor mizura	VU	14.019	4.0956
463	Rodentia	Muridae	Xeromys myoides	VU	13.886	4.0867
464	Rodentia	Caviidae	Dolichotis patagonum	NT	28.659	4.0829
464	Rodentia	Caviidae	Dolichotis salinicola	NT	28.659	4.0829
466	Chiroptera	Nycteridae	Nycteris major	VU	13.826	4.0827
467	Chiroptera	Pteropodidae	Neopteryx frosti	VU	13.771	4.0790
468	Insectivora	Tenrecidae	Micropotamogale ruwenzorii	NT	28.424	4.0749
469	Xenarthra	Dasypodidae	Dasypus pilosus	VU	13.700	4.0742
470	Rodentia	Dipodidae	Salpingotus crassicauda	VU	13.662	4.0715
471	Chiroptera	Pteropodidae	Acerodon jubatus	EN	6.329	4.0712
472	Rodentia	Chinchillidae	Chinchilla lanigera	VU	13.638	4.0699
473	Chiroptera	Phyllostomidae	Micronycteris behnii	VU	13.602	4.0675
474	Rodentia	Muridae	Mus kasaicus	CR	2.648	4.0667
475	Rodentia	Sciuridae	Hylopetes alboniger	EN	6.294	4.0665
476	Rodentia	Muridae	Nannospalax leucodon	VU	13.585	4.0663
477	Primates	Cercopithecidae	Mandrillus leucophaeus	EN	6.242	4.0594
478	Carnivora	Mustelidae	Pteronura brasiliensis	EN	6.220	4.0563
479	Peramelemorphia	Peramelidae	Perameles gunnii	VU	13.425	4.0553
480	Chiroptera	Vespertilionidae	Myotis schaubi	EN	6.201	4.0536
481	Primates	Cebidae	Alouatta pigra	EN	6.186	4.0515
482	Primates	Galagonidae	Galago alleni	NT	27.736	4.0513
483	Perissodactyla	Rhinocerotidae	Ceratotherium simum	NT	27.656	4.0485
484	Lagomorpha	Ochotonidae	Ochotona iliensis	VU	13.328	4.0485
485	Primates	Cercopithecidae	Semnopithecus entellus	EN	6.164	4.0484
485	Primates	Cercopithecidae	Trachypithecus vetulus	EN	6.164	4.0484
487	Rodentia	Muridae	Crunomys fallax	CR	2.566	4.0441
488	Rodentia	Sciuridae	Prosciurillus abstrusus	VU	13.259	4.0437
489	Primates	Lemuridae	Eulemur coronatus	VU	13.214	4.0405
490	Didelphimorphia	Didelphidae	Thylamys macrura	NT	27.407	4.0398
491	Scandentia	Tupaiidae	Tupaia chrysogaster	VU	13.202	4.0397
491	Scandentia	Tupaiidae	Tupaia palawanensis	VU	13.202	4.0397
493	Carnivora	Ursidae	Ursus maritimus	VU	13.199	4.0395
494	Artiodactyla	Bovidae	Hemitragus hylocrius	EN	6.063	4.0343
494	Artiodactyla	Bovidae	Hemitragus jayakari	EN	6.063	4.0343
496	Xenarthra	Myrmecophagidae	Cyclopes didactylus	LC	55.297	4.0306
497	Chiroptera	Pteropodidae	Syconycteris carolinae	VU	13.034	4.0278
497	Chiroptera	Pteropodidae	Syconycteris hobbit	VU	13.034	4.0278
499	Chiroptera	Emballonuridae	Diclidurus isabellus	NT	26.976	4.0245

Rank	Order	Family	Species	IUCN	ED'	EDGE
500	Primates	Cebidae	Saimiri oerstedii	EN	5.950	4.0181
501	Chiroptera	Vespertilionidae	Antrozous dubiaquercus	VU	12.823	4.0126
502	Rodentia	Sciuridae	Ratufa indica	VU	12.804	4.0112
503	Primates	Lemuridae	Eulemur mongoz	VU	12.755	4.0077
503	Primates	Lemuridae	Eulemur rubriventer	VU	12.755	4.0077
505	Primates	Cercopithecidae	Cercopithecus erythrogaster	EN	5.853	4.0041
505	Primates	Cercopithecidae	Cercopithecus sclateri	EN	5.853	4.0041
507	Lagomorpha	Leporidae	Lepus flavigularis	EN	5.845	4.0030
508	Primates	Lemuridae	Eulemur macaco	VU	12.682	4.0024
509	Didelphimorphia	Didelphidae	Marmosops dorothea	VU	12.585	3.9953
510	Carnivora	Mustelidae	Mydaus marchei	VU	12.522	3.9906
511	Rodentia	Dipodidae	Eozapus setchuanus	VU	12.477	3.9872
512	Carnivora	Felidae	Prionailurus rubiginosus	VU	12.437	3.9843
513	Artiodactyla	Cervidae	Hippocamelus bisulcus	EN	5.679	3.9785
514	Rodentia	Anomaluridae	Anomalurus pelii	NT	25.688	3.9774
515	Didelphimorphia	Didelphidae	Monodelphis emiliae	VU	12.337	3.9768
515	Didelphimorphia	Didelphidae	Monodelphis maraxina	VU	12.337	3.9768
515	Didelphimorphia	Didelphidae	Monodelphis osgoodi	VU	12.337	3.9768
515	Didelphimorphia	Didelphidae	Monodelphis rubida	VU	12.337	3.9768
515	Didelphimorphia	Didelphidae	Monodelphis scalops	VU	12.337	3.9768
515	Didelphimorphia	Didelphidae	Monodelphis sorex	VU	12.337	3.9768
515	Didelphimorphia	Didelphidae	Monodelphis theresa	VU	12.337	3.9768
515	Didelphimorphia	Didelphidae	Monodelphis unistriata	VU	12.337	3.9768
523	Chiroptera	Emballonuridae	Saccolaimus peli	NT	25.650	3.9759
524	Rodentia	Muridae	Meriones arimalius	EN	5.644	3.9732
524	Rodentia	Muridae	Meriones dahli	EN	5.644	3.9732
524	Rodentia	Muridae	Meriones sacramenti	EN	5.644	3.9732
524	Rodentia	Muridae	Meriones zarudnyi	EN	5.644	3.9732
528	Rodentia	Muridae	Mesocricetus newtoni	VU	12.281	3.9727
529	Dermoptera	Cynocephalidae	Cynocephalus variegatus	LC	52.120	3.9725
530	Rodentia	Muridae	Podomys floridanus	VU	12.266	3.9715
531	Chiroptera	Pteropodidae	Alionycteris paucidentata	VU	12.228	3.9686
532	Carnivora	Felidae	Prionailurus viverrinus	VU	12.222	3.9682
533	Primates	Callitrichidae	Leontopithecus chrysomelas	EN	5.588	3.9646
534	Lagomorpha	Leporidae	Sylvilagus transitionalis	VU	12.163	3.9637
535	Chiroptera	Vespertilionidae	Murina tenebrosa	CR	2.286	3.9622
536	Chiroptera	Phyllostomidae	Sturnira thomasi	EN	5.570	3.9619
537	Pholidota	Manidae	Manis javanica	NT	25.182	3.9582
538	Chiroptera	Vespertilionidae	Plecotus rafinesquii	VU	12.011	3.9521
539	Chiroptera	Molossidae	Mops petersoni	VU	11.998	3.9511
540	Lagomorpha	Leporidae	Brachylagus idahoensis	NT	24.942	3.9490
541	Diprotodontia	Potoroidae	Bettongia lesueur	VU	11.960	3.9482
542	Lagomorpha	Leporidae	Lepus hainanus	VU	11.960	3.9481
543	Primates	Callitrichidae	Leontopithecus rosalia	EN	5.475	3.9474
544	Rodentia	Muridae	Pseudomys occidentalis	EN	5.469	3.9464
544	Rodentia	Muridae	Pseudomys oralis	EN	5.469	3.9464
546	Carnivora	Procyonidae	Procyon insularis	EN	5.466	3.9460
546	Carnivora	Procyonidae	Procyon maynardi	EN	5.466	3.9460
546	Carnivora	Procyonidae	Procyon minor	EN	5.466	3.9460
546	Carnivora	Procyonidae	Procyon pygmaeus	EN	5.466	3.9460
550	Rodentia	Heteromyidae	Dipodomys gravipes	EN	5.446	3.9429
551	Primates	Cercopithecidae	Pygathrix bieti	EN	5.429	3.9403
551	Primates	Cercopithecidae	Pygathrix brelichi	EN	5.429	3.9403
553	Chiroptera	Vespertilionidae	Plecotus taivanus	VU	11.846	3.9393
554	Chiroptera	Vespertilionidae	Myotis milleri	EN	5.407	3.9368

Rank	Order	Family	Species	IUCN	ED'	EDGE
555	Rodentia	Muridae	Spalax arenarius	VU	11.776	3.9338
555	Rodentia	Muridae	Spalax giganteus	VU	11.776	3.9338
555	Rodentia	Muridae	Spalax graecus	VU	11.776	3.9338
555	Rodentia	Muridae	Spalax microphthalmus	VU	11.776	3.9338
559	Primates	Loridae	Arctocebus aureus	NT	24.546	3.9336
559	Primates	Loridae	Arctocebus calabarensis	NT	24.546	3.9336
561	Rodentia	Octodontidae	Octodontomys gliroides	LC	49.951	3.9309
561	Rodentia	Octodontidae	Spalacopus cyanus	LC	49.951	3.9309
563	Artiodactyla	Bovidae	Ovis vignei	EN	5.347	3.9274
564	Chiroptera	Phyllostomidae	Leptonycteris curasoae	VU	11.644	3.9235
565	Rodentia	Muridae	Eropeplus canus	EN	5.317	3.9227
565	Rodentia	Muridae	Paruromys ursinus	EN	5.317	3.9227
567	Chiroptera	Molossidae	Tadarida brasiliensis	NT	24.147	3.9179
568	Artiodactyla	Bovidae	Cephalophus rubidus	EN	5.286	3.9178
569	Chiroptera	Vespertilionidae	Myotis findleyi	EN	5.264	3.9143
569	Chiroptera	Vespertilionidae	Myotis sodalis	EN	5.264	3.9143
571	Artiodactyla	Cervidae	Cervus alfredi	EN	5.259	3.9135
572	Chiroptera	Molossidae	Mops trevori	VU	11.496	3.9117
573	Rodentia	Sciuridae	Spermophilus brunneus	EN	5.222	3.9075
574	Chiroptera	Vespertilionidae	Myotis pruinosus	EN	5.220	3.9071
575	Chiroptera	Phyllostomidae	Chiroderma improvisum	EN	5.203	3.9044
576	Rodentia	Sciuridae	Lariscus hosei	VU	11.393	3.9034
577	Rodentia	Muridae	Steatomys jacksoni	VU	11.388	3.9030
578	Artiodactyla	Camelidae	Vicugna vicugna	CD	23.740	3.9016
579	Insectivora	Soricidae	Cryptotis gracilis	VU	11.355	3.9003
579	Insectivora	Soricidae	Cryptotis hondurensis	VU	11.355	3.9003
581	Artiodactyla	Bovidae	Capra caucasica	EN	5.129	3.8925
581	Artiodactyla	Bovidae	Capra falconeri	EN	5.129	3.8925
581	Artiodactyla	Bovidae	Capra nubiana	EN	5.129	3.8925
584	Primates	Callitrichidae	Saguinus oedipus	EN	5.122	3.8913
585	Didelphimorphia	Didelphidae	Chironectes minimus	NT	23.443	3.8895
586	Carnivora	Viverridae	Paradoxurus jerdoni	VU	11.115	3.8807
587	Rodentia	Myoxidae	Dryomys laniger	NT	23.144	3.8772
587	Rodentia	Myoxidae	Dryomys nitedula	NT	23.144	3.8772
589	Pholidota	Manidae	Manis crassicaudata	NT	23.124	3.8763
589	Pholidota	Manidae	Manis pentadactyla	NT	23.124	3.8763
591	Chiroptera	Emballonuridae	Taphozous hamiltoni	NT	23.119	3.8761
592	Insectivora	Tenrecidae	Microgale dryas	VU	11.052	3.8755
592	Insectivora	Tenrecidae	Microgale pulla	VU	11.052	3.8755
594	Cetacea	Eschrichtiidae	Eschrichtius robustus	CD	22.929	3.8682
595	Rodentia	Muridae	Chrotomys gonzalesi	CR	1.974	3.8624
596	Carnivora	Felidae	Profelis aurata	VU	10.855	3.8590
597	Chiroptera	Vespertilionidae	Nyctophilus heran	EN	4.925	3.8587
598	Rodentia	Sciuridae	Iomys sipora	VU	10.760	3.8510
599	Chiroptera	Pteropodidae	Pteropus leucopterus	EN	4.863	3.8482
600	Diprotodontia	Pseudocheiridae	Pseudochirops archeri	NT	22.379	3.8450
601	Rodentia	Muridae	Dendromus lovati	VU	10.676	3.8438
601	Rodentia	Muridae	Dendromus oreas	VU	10.676	3.8438
603	Carnivora	Mustelidae	Melogale everetti	VU	10.668	3.8431
604	Carnivora	Mustelidae	Mustela lutreola	EN	4.769	3.8319
604	Carnivora	Mustelidae	Mustela lutreolina	EN	4.769	3.8319
606	Carnivora	Felidae	Pardofelis marmorata	VU	10.517	3.8301
607	Primates	Hylobatidae	Hylobates gabriellae	VU	10.497	3.8284
608	Insectivora	Soricidae	Sorex arizonae	VU	10.493	3.8280
609	Chiroptera	Pteropodidae	Haplonycteris fischeri	VU	10.478	3.8268
609	Chiroptera	Pteropodidae	Otopteropus cartilagonodus	VU	10.478	3.8268

Rank	Order	Family	Species	IUCN	ED'	EDGE
611	Dasyuromorphia	Dasyuridae	Planigale novaeguineae	VU	10.474	3.8263
612	Insectivora	Soricidae	Suncus fellowesgordoni	EN	4.733	3.8258
612	Insectivora	Soricidae	Suncus zeylanicus	EN	4.733	3.8258
614	Carnivora	Mustelidae	Martes gwatkinsii	VU	10.457	3.8249
615	Pholidota	Manidae	Manis temminckii	NT	21.837	3.8215
616	Carnivora	Felidae	Neofelis nebulosa	VU	10.317	3.8126
617	Primates	Cercopithecidae	Cercopithecus preussi	EN	4.657	3.8123
618	Rodentia	Muridae	Tylomys panamensis	VU	10.311	3.8121
619	Carnivora	Hyaenidae	Crocuta crocuta	CD	21.593	3.8108
619	Carnivora	Hyaenidae	Hyaena hyaena	NT	21.593	3.8108
619	Carnivora	Hyaenidae	Parahyaena brunnea	NT	21.593	3.8108
622	Rodentia	Sciuridae	Sciurillus pusillus	LC	44.015	3.8070
623	Primates	Megaladapidae	Lepilemur dorsalis	VU	10.249	3.8066
623	Primates	Megaladapidae	Lepilemur septentrionalis	VU	10.249	3.8066
625	Artiodactyla	Bovidae	Dorcatragus megalotis	VU	10.237	3.8055
626	Primates	Cercopithecidae	Presbytis comata	EN	4.618	3.8053
627	Insectivora	Talpidae	Scaptonyx fusicaudus	LC	43.710	3.8002
628	Chiroptera	Natalidae	Natalus lepidus	NT	21.345	3.7997
629	Carnivora	Felidae	Catopuma temminckii	VU	10.169	3.7995
630	Cetacea	Phocoenidae	Phocoena phocoena	VU	10.167	3.7992
631	Insectivora	Soricidae	Blarinella wardi	NT	21.266	3.7962
632	Primates	Tarsiidae	Tarsius dianae	CD	21.234	3.7948
632	Primates	Tarsiidae	Tarsius spectrum	NT	21.234	3.7948
634	Rodentia	Muridae	Notomys fuscus	VU	10.035	3.7873
635	Diprotodontia	Phalangeridae	Phalanger vestitus	VU	10.034	3.7872
636	Peramelemorphia	Peramelidae	Isoodon auratus	VU	10.011	3.7852
637	Scandentia	Tupaiidae	Anathana ellioti	NT	20.884	3.7789
638	Carnivora	Otariidae	Callorhinus ursinus	VU	9.919	3.7768
639	Primates	Cercopithecidae	Colobus satanas	VU	9.869	3.7722
640	Insectivora	Talpidae	Condylura cristata	LC	42.445	3.7715
641	Dasyuromorphia	Dasyuridae	Dasycercus byrnei	VU	9.848	3.7703
641	Dasyuromorphia	Dasyuridae	Dasycercus cristicauda	VU	9.848	3.7703
643	Carnivora	Herpestidae	Bdeogale jacksoni	VU	9.839	3.7695
644	Chiroptera	Rhinolophidae	Rhinolophus cognatus	VU	9.833	3.7689
645	Chiroptera	Phyllostomidae	Vampyrum spectrum	NT	20.647	3.7680
646	Diprotodontia	Petauridae	Petaurus abidi	VU	9.767	3.7628
647	Carnivora	Mustelidae	Lontra felina	EN	4.362	3.7587
647	Carnivora	Mustelidae	Lontra provocax	EN	4.362	3.7587
649	Rodentia	Castoridae	Castor canadensis	LC	41.879	3.7584
650	Rodentia	Muridae	Tryphomys adustus	VU	9.706	3.7571
651	Chiroptera	Molossidae	Molossus sinaloae	LC	41.803	3.7566
652	Rodentia	Muridae	Dinaromys bogdanovi	NT	20.374	3.7553
653	Rodentia	Muridae	Aethomys silindensis	VU	9.675	3.7542
654	Chiroptera	Rhinolophidae	Aselliscus tricuspidatus	LC	41.579	3.7514
655	Rodentia	Muridae	Haeromys margarettae	VU	9.636	3.7505
655	Rodentia	Muridae	Haeromys pusillus	VU	9.636	3.7505
657	Cetacea	Balaenidae	Balaena mysticetus	CD	20.256	3.7498
658	Chiroptera	Pteropodidae	Paranyctimene raptor	NT	20.088	3.7418
659	Chiroptera	Rhinolophidae	Asellia patrizii	VU	9.523	3.7398
660	Xenarthra	Dasypodidae	Tolypeutes matacus	NT	20.029	3.7390
661	Chiroptera	Pteropodidae	Pteropus mariannus	EN	4.254	3.7384
662	Chiroptera	Emballonuridae	Saccolaimus flaviventris	NT	19.942	3.7349
663	Chiroptera	Pteropodidae	Pteropus gilliardi	VU	9.468	3.7346
663	Chiroptera	Pteropodidae	Pteropus mahaganus	VU	9.468	3.7346
665	Primates	Callitrichidae	Callithrix aurita	EN	4.233	3.7344
665	Primates	Callitrichidae	Callithrix flaviceps	EN	4.233	3.7344

Rank	Order	Family	Species	IUCN	ED'	EDGE
667	Diprotodontia	Macropodidae	Dorcopsulus macleayi	VU	9.465	3.7343
668	Chiroptera	Molossidae	Eumops underwoodi	NT	19.913	3.7335
669	Chiroptera	Vespertilionidae	Plecotus townsendii	VU	9.456	3.7335
670	Diprotodontia	Macropodidae	Lagorchestes hirsutus	VU	9.370	3.7252
671	Rodentia	Sciuridae	Cynomys mexicanus	EN	4.177	3.7237
672	Chiroptera	Phyllostomidae	Tonatia carrikeri	VU	9.348	3.7231
672	Chiroptera	Phyllostomidae	Tonatia schulzi	VU	9.348	3.7231
674	Insectivora	Talpidae	Neurotrichus gibbsii	LC	40.247	3.7196
675	Chiroptera	Emballonuridae	Balantiopteryx io	NT	19.570	3.7170
676	Primates	Cercopithecidae	Macaca arctoides	VU	9.270	3.7155
677	Insectivora	Tenrecidae	Geogale aurita	LC	40.058	3.7150
678	Artiodactyla	Bovidae	Madoqua piacentinii	VU	9.232	3.7119
679	Insectivora	Soricidae	Sorex jacksoni	EN	4.113	3.7113
680	Artiodactyla	Cervidae	Dama mesopotamica	VU	9.217	3.7103
681	Chiroptera	Vespertilionidae	Chalinolobus dwyeri	VU	9.216	3.7103
682	Chiroptera	Rhinolophidae	Hipposideros marisae	EN	4.100	3.7087
683	Chiroptera	Vespertilionidae	Myotis bechsteini	VU	9.141	3.7029
684	Artiodactyla	Bovidae	Bos frontalis	VU	9.115	3.7003
685	Rodentia	Sciuridae	Ammospermophilus nelsoni	EN	4.050	3.6988
686	Primates	Cebidae	Ateles belzebuth	VU	9.070	3.6958
687	Chiroptera	Pteropodidae	Myonycteris relicta	VU	9.043	3.6932
688	Rodentia	Muridae	Mesembriomys macrurus	VU	9.014	3.6902
689	Insectivora	Soricidae	Surdisorex norae	VU	8.977	3.6866
689	Insectivora	Soricidae	Surdisorex polulus	VU	8.977	3.6866
691	Chiroptera	Molossidae	Tadarida australis	NT	18.897	3.6837
692	Rodentia	Muridae	Beamys hindei	NT	18.897	3.6837
692	Rodentia	Muridae	Beamys major	NT	18.897	3.6837
694	Dasyuromorphia	Dasyuridae	Sminthopsis butleri	VU	8.942	3.6830
695	Rodentia	Myoxidae	Graphiurus hueti	LC	38.761	3.6829
696	Primates	Cebidae	Aotus brumbacki	VU	8.925	3.6814
696	Primates	Cebidae	Aotus lemurinus	VU	8.925	3.6814
696	Primates	Cebidae	Aotus miconax	VU	8.925	3.6814
699	Primates	Cercopithecidae	Macaca sinica	VU	8.910	3.6798
700	Chiroptera	Emballonuridae	Emballonura raffrayana	NT	18.754	3.6765
701	Rodentia	Capromyidae	Geocapromys brownii	VU	8.820	3.6707
701	Rodentia	Capromyidae	Geocapromys ingrahami	VU	8.820	3.6707
703	Chiroptera	Emballonuridae	Taphozous australis	NT	18.528	3.6650
704	Insectivora	Soricidae	Sylvisorex howelli	VU	8.763	3.6649
704	Insectivora	Soricidae	Sylvisorex morio	VU	8.763	3.6649
706	Rodentia	Erethizontidae	Sphiggurus vestitus	VU	8.757	3.6643
707	Chiroptera	Molossidae	Otomops martiensseni	NT	18.496	3.6633
708	Rodentia	Muridae	Rhagomys rufescens	CR	1.435	3.6627
709	Xenarthra	Dasypodidae	Cabassous chacoensis	NT	18.467	3.6618
710	Rodentia	Capromyidae	Mysateles gundlachi	VU	8.726	3.6611
711	Didelphimorphia	Didelphidae	Metachirus nudicaudatus	LC	37.890	3.6607
712	Rodentia	Muridae	Solomys salamonis	VU	8.716	3.6601
712	Rodentia	Muridae	Solomys sapientis	VU	8.716	3.6601
714	Rodentia	Muridae	Mayermys ellermani	VU	8.702	3.6586
714	Rodentia	Muridae	Neohydromys fuscus	VU	8.702	3.6586
714	Rodentia	Muridae	Paraleptomys wilhelmina	VU	8.702	3.6586
714	Rodentia	Muridae	Pseudohydromys occidentalis	VU	8.702	3.6586
718	Chiroptera	Rhinolophidae	Hipposideros corynophyllus	VU	8.687	3.6570
719	Diprotodontia	Macropodidae	Dendrolagus dorianus	VU	8.657	3.6540
720	Chiroptera	Vespertilionidae	Myotis ozensis	EN	3.824	3.6530
721	Artiodactyla	Suidae	Sus philippensis	VU	8.642	3.6525
722	Chiroptera	Emballonuridae	Rhynchonycteris naso	LC	37.544	3.6518

Rank	Order	Family	Species	IUCN	ED'	EDGE
723	Chiroptera	Mormoopidae	Pteronotus quadridens	NT	18.243	3.6503
724	Chiroptera	Phyllostomidae	Scleronycteris ega	VU	8.600	3.6481
725	Chiroptera	Vespertilionidae	Hesperoptenus gaskelli	VU	8.567	3.6446
725	Chiroptera	Vespertilionidae	Histiotus alienus	VU	8.567	3.6446
727	Rodentia	Caviidae	Kerodon rupestris	LC	37.261	3.6444
728	Rodentia	Hystricidae	Hystrix brachyura	VU	8.515	3.6391
729	Rodentia	Muridae	Cremnomys elvira	VU	8.504	3.6380
730	Chiroptera	Vespertilionidae	Pipistrellus aero	VU	8.496	3.6372
730	Chiroptera	Vespertilionidae	Pipistrellus maderensis	VU	8.496	3.6372
732	Chiroptera	Vespertilionidae	Eptesicus guadeloupensis	EN	3.744	3.6364
733	Didelphimorphia	Didelphidae	Caluromys lanatus	NT	17.932	3.6340
733	Didelphimorphia	Didelphidae	Caluromys philander	NT	17.932	3.6340
735	Artiodactyla	Giraffidae	Giraffa camelopardalis	CD	17.911	3.6329
735	Artiodactyla	Giraffidae	Okapia johnstoni	NT	17.911	3.6329
737	Primates	Cercopithecidae	Trachypithecus auratus	EN	3.726	3.6325
738	Primates	Cercopithecidae	Macaca sylvanus	VU	8.438	3.6310
739	Rodentia	Muridae	Neotoma anthonyi	EN	3.717	3.6306
739	Rodentia	Muridae	Neotoma bryanti	EN	3.717	3.6306
739	Rodentia	Muridae	Neotoma bunkeri	EN	3.717	3.6306
739	Rodentia	Muridae	Neotoma martinensis	EN	3.717	3.6306
739	Rodentia	Muridae	Neotoma nelsoni	EN	3.717	3.6306
739	Rodentia	Muridae	Neotoma varia	EN	3.717	3.6306
745	Artiodactyla	Bovidae	Bos grunniens	VU	8.415	3.6286
746	Artiodactyla	Bovidae	Kobus ellipsiprymnus	CD	17.827	3.6284
747	Insectivora	Talpidae	Parascaptor leucura	LC	36.592	3.6268
748	Carnivora	Felidae	Panthera leo	VU	8.381	3.6250
749	Rodentia	Muridae	Hydromys neobrittanicus	VU	8.369	3.6237
750	Diprotodontia	Potoroidae	Hypsiprymnodon moschatus	LC	36.472	3.6236
751	Carnivora	Canidae	Lycaon pictus	EN	3.683	3.6234
752	Scandentia	Tupaiidae	Ptilocercus lowii	LC	36.307	3.6192
753	Didelphimorphia	Didelphidae	Marmosa lepida	NT	17.618	3.6173
754	Rodentia	Muridae	Archboldomys luzonensis	EN	3.640	3.6142
755	Carnivora	Felidae	Oncifelis guigna	VU	8.279	3.6140
756	Rodentia	Muridae	Taeromys arcuatus	VU	8.263	3.6123
756	Rodentia	Muridae	Taeromys hamatus	VU	8.263	3.6123
758	Rodentia	Muridae	Kunsia fronto	VU	8.261	3.6121
758	Rodentia	Muridae	Kunsia tomentosus	VU	8.261	3.6121
760	Rodentia	Muridae	Crateromys schadenbergi	VU	8.255	3.6115
761	Rodentia	Muridae	Hodomys alleni	NT	17.357	3.6031
761	Rodentia	Muridae	Xenomys nelsoni	NT	17.357	3.6031
763	Rodentia	Muridae	Tarsomys echinatus	VU	8.158	3.6010
764	Chiroptera	Vespertilionidae	Myotis vivesi	VU	8.147	3.5998
765	Lagomorpha	Leporidae	Lepus yarkandensis	NT	17.191	3.5941
766	Chiroptera	Molossidae	Cheiromeles torquatus	NT	17.186	3.5938
767	Chiroptera	Rhinolophidae	Coelops robinsoni	NT	17.151	3.5919
768	Rodentia	Octodontidae	Octomys mimax	LC	35.240	3.5902
769	Rodentia	Muridae	Volemys kikuchii	VU	8.054	3.5895
770	Rodentia	Muridae	Oryzomys gorgasi	CR	1.263	3.5891
771	Rodentia	Muridae	Hybomys lunaris	VU	8.046	3.5886
772	Primates	Hylobatidae	Hylobates klossii	VU	8.043	3.5883
773	Diprotodontia	Macropodidae	Thylogale brunii	VU	8.034	3.5873
774	Artiodactyla	Bovidae	Ammodorcas clarkei	VU	8.032	3.5871
775	Primates	Cercopithecidae	Procolobus pennantii	VU	8.029	3.5867
776	Chiroptera	Vespertilionidae	Lasiurus castaneus	VU	8.011	3.5848
777	Rodentia	Muridae	Margaretamys elegans	VU	8.002	3.5837
777	Rodentia	Muridae	Margaretamys parvus	VU	8.002	3.5837

Rank	Order	Family	Species	IUCN	ED'	EDGE
779	Chiroptera	Thyropteridae	Thyroptera discifera	LC	35.005	3.5837
779	Chiroptera	Thyropteridae	Thyroptera tricolor	LC	35.005	3.5837
781	Rodentia	Bathyergidae	Heterocephalus glaber	LC	34.914	3.5811
782	Rodentia	Muridae	Reithrodontomys rodriguezi	VU	7.966	3.5797
783	Insectivora	Soricidae	Ruwenzorisorex suncoides	VU	7.957	3.5787
784	Rodentia	Muridae	Tateomys macrocercus	VU	7.943	3.5771
784	Rodentia	Muridae	Tateomys rhinogradoides	VU	7.943	3.5771
786	Rodentia	Sciuridae	Sundasciurus jentinki	VU	7.929	3.5756
786	Rodentia	Sciuridae	Sundasciurus rabori	VU	7.929	3.5756
786	Rodentia	Sciuridae	Sundasciurus samarensis	VU	7.929	3.5756
789	Macroscelidea	Macroscelididae	Petrodromus tetradactylus	LC	34.662	3.5741
790	Diprotodontia	Pseudocheiridae	Hemibelideus lemuroides	NT	16.815	3.5732
791	Artiodactyla	Bovidae	Kobus leche	CD	16.769	3.5706
791	Artiodactyla	Bovidae	Kobus megaceros	NT	16.769	3.5706
793	Insectivora	Soricidae	Myosorex babaulti	VU	7.879	3.5700
793	Insectivora	Soricidae	Myosorex blarina	VU	7.879	3.5700
793	Insectivora	Soricidae	Myosorex longicaudatus	VU	7.879	3.5700
793	Insectivora	Soricidae	Myosorex sclateri	VU	7.879	3.5700
797	Chiroptera	Vespertilionidae	Pipistrellus arabicus	VU	7.860	3.5678
798	Paucituberculata	Caenolestidae	Lestoros inca	LC	34.420	3.5673
799	Carnivora	Felidae	Felis nigripes	VU	7.852	3.5669
800	Insectivora	Erinaceidae	Hylomys suillus	LC	34.394	3.5665
801	Artiodactyla	Tayassuidae	Pecari tajacu	LC	34.379	3.5661
802	Primates	Cercopithecidae	Macaca cyclopis	VU	7.835	3.5650
803	Rodentia	Dipodidae	Pygeretmus shitkovi	NT	16.654	3.5641
804	Chiroptera	Emballonuridae	Taphozous theobaldi	LC	34.287	3.5635
805	Chiroptera	Emballonuridae	Mosia nigrescens	LC	34.278	3.5633
806	Rodentia	Muridae	Grammomys minnae	VU	7.819	3.5632
807	Rodentia	Muridae	Thamnomys kempi	VU	7.815	3.5628
808	Insectivora	Soricidae	Sorex hosonoi	VU	7.790	3.5599
809	Artiodactyla	Bovidae	Syncerus caffer	CD	16.578	3.5598
810	Insectivora	Talpidae	Scapanulus oweni	LC	34.081	3.5577
811	Rodentia	Muridae	Ichthyomys pittieri	VU	7.765	3.5570
812	Chiroptera	Phyllostomidae	Micronycteris daviesi	NT	16.527	3.5569
813	Chiroptera	Pteropodidae	Dobsonia beauforti	EN	3.381	3.5567
814	Chiroptera	Phyllostomidae	Sturnira bidens	NT	16.506	3.5557
815	Rodentia	Geomyidae	Geomys tropicalis	VU	7.748	3.5551
816	Chiroptera	Vespertilionidae	Pipistrellus cuprosus	VU	7.744	3.5546
817	Carnivora	Otariidae	Phocarctos hookeri	VU	7.744	3.5546
818	Chiroptera	Molossidae	Molossops mattogrossensis	NT	16.477	3.5540
819	Diprotodontia	Phalangeridae	Wyulda squamicaudata	NT	16.431	3.5514
820	Cetacea	Neobalaenidae	Caperea marginata	LC	33.849	3.5510
821	Insectivora	Talpidae	Parascalops breweri	LC	33.799	3.5496
822	Chiroptera	Rhinolophidae	Hipposideros inexpectatus	VU	7.672	3.5463
823	Didelphimorphia	Didelphidae	Gracilinanus marica	NT	16.341	3.5462
824	Chiroptera	Pteropodidae	Megaerops kusnotoi	VU	7.648	3.5436
825	Cetacea	Physeteridae	Kogia breviceps	LC	33.559	3.5427
825	Cetacea	Physeteridae	Kogia simus	LC	33.559	3.5427
827	Primates	Galagonidae	Galago gallarum	NT	16.277	3.5425
827	Primates	Galagonidae	Galago matschiei	NT	16.277	3.5425
829	Cetacea	Balaenidae	Eubalaena australis	CD	16.242	3.5405
830	Primates	Cercopithecidae	Allenopithecus nigroviridis	NT	16.220	3.5392
831	Primates	Cebidae	Callicebus modestus	VU	7.578	3.5355
831	Primates	Cebidae	Callicebus oenanthe	VU	7.578	3.5355
831	Primates	Cebidae	Callicebus olallae	VU	7.578	3.5355
834	Chiroptera	Furipteridae	Furipterus horrens	LC	33.242	3.5334

Rank	Order	Family	Species	IUCN	ED'	EDGE
835	Carnivora	Otariidae	Arctocephalus townsendi	VU	7.537	3.5307
836	Rodentia	Muridae	Leopoldamys siporanus	VU	7.537	3.5306
836	Rodentia	Muridae	Pogonomys championi	VU	7.537	3.5306
838	Chiroptera	Vespertilionidae	Miniopterus schreibersi	NT	16.056	3.5296
839	Chiroptera	Rhinolophidae	Rhinolophus monoceros	NT	16.053	3.5295
840	Carnivora	Felidae	Puma concolor	NT	16.044	3.5290
841	Chiroptera	Rhinolophidae	Hipposideros muscinus	VU	7.513	3.5278
842	Chiroptera	Phyllostomidae	Lonchophylla bokermanni	VU	7.509	3.5274
842	Chiroptera	Phyllostomidae	Lonchophylla dekeyseri	VU	7.509	3.5274
842	Chiroptera	Phyllostomidae	Lonchophylla handleyi	VU	7.509	3.5274
842	Chiroptera	Phyllostomidae	Lonchophylla hesperia	VU	7.509	3.5274
846	Chiroptera	Molossidae	Molossus bondae	LC	33.024	3.5271
846	Chiroptera	Molossidae	Molossus molossus	LC	33.024	3.5271
848	Rodentia	Muridae	Apodemus hermonensis	EN	3.249	3.5260
849	Rodentia	Muridae	Microtus kermanensis	EN	3.244	3.5250
850	Chiroptera	Pteropodidae	Pteropus dasymallus	EN	3.242	3.5245
850	Chiroptera	Pteropodidae	Pteropus faunulus	EN	3.242	3.5245
852	Rodentia	Sciuridae	Spermophilopsis leptodactylus	LC	32.928	3.5242
853	Rodentia	Muridae	Phaulomys andersoni	VU	7.477	3.5236
854	Insectivora	Tenrecidae	Potamogale velox	LC	32.904	3.5235
855	Rodentia	Muridae	Praomys morio	VU	7.473	3.5232
856	Artiodactyla	Bovidae	Cephalophus jentinki	VU	7.463	3.5220
857	Rodentia	Sciuridae	Spermophilus mohavensis	VU	7.411	3.5159
858	Carnivora	Canidae	Canis simensis	EN	3.188	3.5117
859	Primates	Cercopithecidae	Macaca nigra	VU	7.366	3.5105
860	Chiroptera	Rhinolophidae	Rhinolophus cornutus	NT	15.665	3.5065
861	Lagomorpha	Leporidae	Lepus castroviejoi	VU	7.311	3.5038
862	Artiodactyla	Bovidae	Damaliscus lunatus	CD	15.580	3.5014
862	Artiodactyla	Bovidae	Damaliscus pygargus	CD	15.580	3.5014
864	Dasyuromorphia	Dasyuridae	Dasyurus maculatus	VU	7.287	3.5010
865	Rodentia	Muridae	Reithrodontomys raviventris	VU	7.270	3.4989
866	Rodentia	Echimyidae	Diplomys rufodorsalis	VU	7.260	3.4977
867	Chiroptera	Pteropodidae	Nyctimene celaeno	VU	7.253	3.4968
868	Primates	Galagonidae	Euoticus elegantulus	NT	15.489	3.4958
868	Primates	Galagonidae	Euoticus pallidus	NT	15.489	3.4958
870	Xenarthra	Myrmecophagidae	Tamandua mexicana	LC	31.951	3.4950
870	Xenarthra	Myrmecophagidae	Tamandua tetradactyla	LC	31.951	3.4950
872	Xenarthra	Dasypodidae	Zaedyus pichiy	NT	15.413	3.4912
873	Insectivora	Erinaceidae	Echinosorex gymnura	LC	31.823	3.4911
874	Primates	Cebidae	Saimiri vanzolinii	VU	7.204	3.4909
875	Rodentia	Muridae	Tachyoryctes macrocephalus	VU	7.184	3.4885
876	Rodentia	Sciuridae	Paraxerus vexillarius	VU	7.180	3.4880
877	Primates	Galagonidae	Galagoides demidoff	LC	31.680	3.4868
878	Chiroptera	Vespertilionidae	Myotis grisescens	EN	3.083	3.4862
879	Rodentia	Muridae	Myospalax fontanierii	VU	7.133	3.4823
880	Chiroptera	Vespertilionidae	Myotis scotti	VU	7.110	3.4794
881	Chiroptera	Molossidae	Tadarida ventralis	NT	15.189	3.4775
882	Diprotodontia	Tarsipedidae	Tarsipes rostratus	LC	31.349	3.4766
883	Rodentia	Muridae	Stenocephalemys albocaudata	NT	15.158	3.4756
883	Rodentia	Muridae	Stenocephalemys griseicauda	NT	15.158	3.4756
885	Chiroptera	Molossidae	Myopterus daubentonii	NT	15.144	3.4747
886	Rodentia	Muridae	Hylomyscus baeri	EN	3.034	3.4742
887	Rodentia	Muridae	Maxomys dollmani	VU	7.048	3.4717
888	Chiroptera	Phyllostomidae	Lonchorhina orinocensis	NT	15.063	3.4697
889	Didelphimorphia	Didelphidae	Gracilinanus agilis	NT	15.020	3.4670
889	Didelphimorphia	Didelphidae	Gracilinanus microtarsus	NT	15.020	3.4670

Rank	Order	Family	Species	IUCN	ED'	EDGE
891	Rodentia	Muridae	Brachyuromys betsileoensis	NT	15.015	3.4667
891	Rodentia	Muridae	Brachyuromys ramirohitra	NT	15.015	3.4667
893	Didelphimorphia	Didelphidae	Micoureus alstoni	NT	14.999	3.4657
893	Didelphimorphia	Didelphidae	Micoureus constantiae	NT	14.999	3.4657
895	Chiroptera	Pteropodidae	Aethalops alecto	NT	14.969	3.4638
896	Insectivora	Talpidae	Scalopus aquaticus	LC	30.821	3.4601
897	Chiroptera	Rhinolophidae	Cloeotis percivali	VU	6.951	3.4596
898	Rodentia	Dipodidae	Allactodipus bobrinskii	LC	30.769	3.4585
899	Carnivora	Felidae	Felis bieti	VU	6.906	3.4539
900	Insectivora	Tenrecidae	Hemicentetes semispinosus	LC	30.590	3.4528
900	Insectivora	Tenrecidae	Tenrec ecaudatus	LC	30.590	3.4528
902	Chiroptera	Mormoopidae	Mormoops megalophylla	LC	30.474	3.4492
903	Chiroptera	Vespertilionidae	Eptesicus nasutus	VU	6.867	3.4490
904	Primates	Cercopithecidae	Trachypithecus geei	EN	2.907	3.4421
904	Primates	Cercopithecidae	Trachypithecus pileatus	EN	2.907	3.4421
906	Hyracoidea	Procaviidae	Procavia capensis	LC	30.242	3.4418
907	Chiroptera	Pteropodidae	Nyctimene draconilla	VU	6.729	3.4313
907	Chiroptera	Pteropodidae	Nyctimene minutus	VU	6.729	3.4313
909	Lagomorpha	Leporidae	Sylvilagus mansuetus	NT	14.458	3.4312
910	Artiodactyla	Bovidae	Kobus kob	CD	14.434	3.4297
910	Artiodactyla	Bovidae	Kobus vardonii	CD	14.434	3.4297
912	Chiroptera	Vespertilionidae	Pipistrellus anchietai	VU	6.697	3.4271
913	Diprotodontia	Vombatidae	Vombatus ursinus	LC	29.684	3.4237
914	Chiroptera	Phyllostomidae	Brachyphylla nana	NT	14.323	3.4225
915	Chiroptera	Phyllostomidae	Rhinophylla alethina	NT	14.323	3.4225
915	Chiroptera	Phyllostomidae	Rhinophylla fischerae	NT	14.323	3.4225
917	Rodentia	Sciuridae	Funambulus tristriatus	NT	14.321	3.4224
918	Artiodactyla	Cervidae	Muntiacus crinifrons	VU	6.646	3.4204
919	Primates	Callitrichidae	Callimico goeldii	NT	14.237	3.4169
920	Primates	Callitrichidae	Saguinus leucopus	VU	6.589	3.4130
921	Primates	Cercopithecidae	Macaca nemestrina	VU	6.547	3.4074
922	Rodentia	Dasyproctidae	Dasyprocta azarae	VU	6.529	3.4050
923	Chiroptera	Vespertilionidae	Miniopterus minor	NT	14.045	3.4042
924	Chiroptera	Molossidae	Molossops neglectus	NT	14.019	3.4025
925	Chiroptera	Molossidae	Eumops dabbenei	LC	29.010	3.4015
926	Insectivora	Talpidae	Urotrichus pilirostris	LC	28.983	3.4006
926	Insectivora	Talpidae	Urotrichus talpoides	LC	28.983	3.4006
928	Chiroptera	Phyllostomidae	Stenoderma rufum	VU	6.474	3.3977
929	Rodentia	Sciuridae	Marmota vancouverensis	EN	2.734	3.3968
930	Carnivora	Phocidae	Phoca caspica	VU	6.456	3.3954
931	Macroscelidea	Macroscelididae	Macroscelides proboscideus	LC	28.798	3.3945
932	Diprotodontia	Macropodidae	Macropus eugenii	NT	13.855	3.3915
933	Primates	Cheirogaleidae	Cheirogaleus major	LC	28.703	3.3912
933	Primates	Cheirogaleidae	Cheirogaleus medius	LC	28.703	3.3912
935	Chiroptera	Nycteridae	Nycteris intermedia	NT	13.826	3.3896
936	Chiroptera	Phyllostomidae	Choeroniscus periosus	VU	6.411	3.3893
937	Chiroptera	Pteropodidae	Styloctenium wallacei	NT	13.771	3.3858
938	Rodentia	Muridae	Leggadina forresti	NT	13.771	3.3858
938	Rodentia	Muridae	Leggadina lakedownensis	NT	13.771	3.3858
940	Rodentia	Geomyidae	Pappogeomys alcorni	VU	6.383	3.3854
941	Insectivora	Tenrecidae	Oryzorictes hova	LC	28.524	3.3852
941	Insectivora	Tenrecidae	Oryzorictes talpoides	LC	28.524	3.3852
943	Xenarthra	Dasypodidae	Dasypus hybridus	NT	13.700	3.3810
944	Rodentia	Dipodidae	Salpingotus heptneri	NT	13.662	3.3784
944	Rodentia	Dipodidae	Salpingotus kozlovi	NT	13.662	3.3784

Rank	Order	Family	Species	IUCN	ED'	EDGE
946	Chiroptera	Pteropodidae	Acerodon humilis	VU	6.329	3.3781
946	Chiroptera	Pteropodidae	Acerodon leucotis	VU	6.329	3.3781
948	Chiroptera	Phyllostomidae	Micronycteris sylvestris	NT	13.602	3.3743
949	Insectivora	Soricidae	Anourosorex squamipes	LC	28.199	3.3741
950	Artiodactyla	Moschidae	Moschus berezovskii	NT	13.561	3.3715
951	Primates	Loridae	Perodicticus potto	LC	28.088	3.3703
952	Primates	Loridae	Nycticebus coucang	LC	27.985	3.3668
953	Primates	Cercopithecidae	Mandrillus sphinx	VU	6.242	3.3662
954	Chiroptera	Phyllostomidae	Macrophyllum macrophyllum	LC	27.888	3.3634
955	Carnivora	Mustelidae	Lutrogale perspicillata	VU	6.220	3.3632
956	Artiodactyla	Bovidae	Tragelaphus angasii	CD	13.436	3.3629
956	Artiodactyla	Bovidae	Tragelaphus imberbis	CD	13.436	3.3629
958	Chiroptera	Noctilionidae	Noctilio albiventris	LC	27.722	3.3577
958	Chiroptera	Noctilionidae	Noctilio leporinus	LC	27.722	3.3577
960	Chiroptera	Molossidae	Eumops bonariensis	LC	27.694	3.3567
961	Primates	Cercopithecidae	Trachypithecus johnii	VU	6.164	3.3553
962	Chiroptera	Vespertilionidae	Scotomanes ornatus	NT	13.306	3.3538
963	Chiroptera	Molossidae	Molossops abrasus	NT	13.301	3.3534
964	Chiroptera	Molossidae	Molossus ater	LC	27.590	3.3530
964	Chiroptera	Molossidae	Molossus pretiosus	LC	27.590	3.3530
966	Macroscelidea	Macroscelididae	Elephantulus rozeti	LC	27.577	3.3526
967	Rodentia	Muridae	Crunomys celebensis	EN	2.566	3.3509
968	Rodentia	Sciuridae	Prosciurillus weberi	NT	13.259	3.3505
969	Artiodactyla	Bovidae	Gazella cuvieri	EN	2.562	3.3499
969	Artiodactyla	Bovidae	Gazella leptoceros	EN	2.562	3.3499
971	Insectivora	Tenrecidae	Echinops telfairi	LC	27.498	3.3498
971	Insectivora	Tenrecidae	Setifer setosus	LC	27.498	3.3498
973	Primates	Hylobatidae	Hylobates pileatus	VU	6.104	3.3470
974	Didelphimorphia	Didelphidae	Thylamys pusilla	LC	27.407	3.3466
975	Chiroptera	Molossidae	Promops centralis	LC	27.397	3.3463
975	Chiroptera	Molossidae	Promops nasutus	LC	27.397	3.3463
977	Didelphimorphia	Didelphidae	Marmosops fuscatus	NT	13.192	3.3458
977	Didelphimorphia	Didelphidae	Marmosops parvidens	NT	13.192	3.3458
979	Rodentia	Muridae	Isthmomys pirrensis	NT	13.167	3.3441
980	Hyracoidea	Procaviidae	Heterohyrax brucei	LC	27.319	3.3435
981	Chiroptera	Pteropodidae	Nyctimene malaitensis	VU	6.064	3.3414
981	Chiroptera	Pteropodidae	Nyctimene masalai	VU	6.064	3.3414
983	Artiodactyla	Bovidae	Hemitragus jemlahicus	VU	6.063	3.3411
984	Rodentia	Sciuridae	Pteromys volans	NT	13.124	3.3410
985	Chiroptera	Pteropodidae	Pteropus temmincki	NT	13.120	3.3408
986	Rodentia	Muridae	Rattus bontanus	VU	6.060	3.3408
986	Rodentia	Muridae	Rattus pelurus	VU	6.060	3.3408
988	Lagomorpha	Ochotonidae	Ochotona thomasi	NT	13.093	3.3388
989	Chiroptera	Emballonuridae	Centronycteris maximiliani	LC	27.057	3.3342
989	Chiroptera	Emballonuridae	Cormura brevirostris	LC	27.057	3.3342
991	Paucituberculata	Caenolestidae	Caenolestes caniventer	LC	27.024	3.3331
991	Paucituberculata	Caenolestidae	Caenolestes convelatus	LC	27.024	3.3331
991	Paucituberculata	Caenolestidae	Caenolestes fuliginosus	LC	27.024	3.3331
994	Rodentia	Anomaluridae	Idiurus macrotis	LC	26.977	3.3314
995	Artiodactyla	Cervidae	Hydropotes inermis	NT	12.972	3.3302
996	Rodentia	Dipodidae	Allactaga bullata	NT	12.964	3.3296
996	Rodentia	Dipodidae	Allactaga euphratica	NT	12.964	3.3296
998	Chiroptera	Megadermatidae	Cardioderma cor	LC	26.864	3.3274
998	Chiroptera	Megadermatidae	Lavia frons	LC	26.864	3.3274
1000	Primates	Cercopithecidae	Theropithecus gelada	NT	12.881	3.3237

Rank	Order	Family	Species	IUCN	ED'	EDGE
1001	Rodentia	Muridae	Peromyscus pseudocrinitus	CR	0.732	3.3219
1001	Rodentia	Muridae	Peromyscus slevini	CR	0.732	3.3219
1003	Chiroptera	Nycteridae	Nycteris woodi	NT	12.799	3.3177
1004	Artiodactyla	Tayassuidae	Tayassu pecari	LC	26.533	3.3154
1005	Diprotodontia	Vombatidae	Lasiorhinus latifrons	LC	26.528	3.3152
1006	Rodentia	Sciuridae	Tamias palmeri	VU	5.868	3.3132
1007	Carnivora	Procyonidae	Bassariscus sumichrasti	NT	12.732	3.3129
1008	Chiroptera	Molossidae	Mormopterus petrophilus	LC	26.449	3.3123
1009	Rodentia	Agoutidae	Agouti taczanowskii	NT	12.721	3.3121
1010	Carnivora	Odobenidae	Odobenus rosmarus	LC	26.435	3.3118
1011	Primates	Cercopithecidae	Cercopithecus erythrotis	VU	5.853	3.3110
1012	Carnivora	Mustelidae	Mellivora capensis	LC	26.400	3.3105
1013	Diprotodontia	Macropodidae	Dendrolagus bennettianus	NT	12.651	3.3070
1014	Rodentia	Muridae	Andalgalomys olrogi	VU	5.824	3.3067
1015	Insectivora	Talpidae	Scaptochirus moschatus	LC	26.217	3.3038
1016	Chiroptera	Phyllostomidae	Monophyllus plethodon	NT	12.606	3.3037
1017	Didelphimorphia	Didelphidae	Marmosops impavidus	NT	12.585	3.3021
1017	Didelphimorphia	Didelphidae	Marmosops incanus	NT	12.585	3.3021
1017	Didelphimorphia	Didelphidae	Marmosops invictus	NT	12.585	3.3021
1020	Carnivora	Hyaenidae	Proteles cristatus	LC	26.141	3.3011
1021	Rodentia	Sciuridae	Menetes berdmorei	LC	26.134	3.3008
1021	Rodentia	Sciuridae	Rhinosciurus laticaudatus	LC	26.134	3.3008
1023	Hyracoidea	Procaviidae	Dendrohyrax arboreus	LC	26.004	3.2960
1023	Hyracoidea	Procaviidae	Dendrohyrax dorsalis	LC	26.004	3.2960
1025	Diprotodontia	Burramyidae	Cercartetus caudatus	LC	25.999	3.2958
1026	Rodentia	Chinchillidae	Lagidium wolffsohni	NT	12.499	3.2957
1027	Artiodactyla	Cervidae	Pudu puda	VU	5.746	3.2953
1028	Lagomorpha	Leporidae	Oryctolagus cuniculus	LC	25.966	3.2946
1029	Artiodactyla	Bovidae	Redunca arundinum	CD	12.474	3.2939
1029	Artiodactyla	Bovidae	Redunca redunca	CD	12.474	3.2939
1031	Artiodactyla	Bovidae	Cephalophus maxwellii	NT	12.461	3.2930
1032	Diprotodontia	Potoroidae	Potorous tridactylus	LC	25.895	3.2919
1033	Rodentia	Octodontidae	Aconaemys fuscus	LC	25.889	3.2917
1033	Rodentia	Octodontidae	Aconaemys sagei	LC	25.889	3.2917
1035	Artiodactyla	Bovidae	Tragelaphus strepsiceros	CD	12.429	3.2906
1036	Chiroptera	Phyllostomidae	Musonycteris harrisoni	VU	5.707	3.2894
1037	Artiodactyla	Bovidae	Ovis ammon	VU	5.704	3.2890
1038	Rodentia	Muridae	Mylomys dybowskii	LC	25.727	3.2857
1039	Diprotodontia	Pseudocheiridae	Pseudocheirus herbertensis	NT	12.354	3.2849
1040	Rodentia	Anomaluridae	Anomalurus beecrofti	LC	25.688	3.2842
1040	Rodentia	Anomaluridae	Anomalurus derbianus	LC	25.688	3.2842
1040	Rodentia	Anomaluridae	Anomalurus pusillus	LC	25.688	3.2842
1043	Didelphimorphia	Didelphidae	Monodelphis americana	NT	12.337	3.2837
1043	Didelphimorphia	Didelphidae	Monodelphis dimidiata	NT	12.337	3.2837
1043	Didelphimorphia	Didelphidae	Monodelphis iheringi	NT	12.337	3.2837
1046	Artiodactyla	Bovidae	Tragelaphus eurycerus	NT	12.260	3.2779
1046	Artiodactyla	Bovidae	Tragelaphus spekii	NT	12.260	3.2779
1048	Rodentia	Muridae	Otomys occidentalis	VU	5.625	3.2771
1049	Insectivora	Talpidae	Scapanus latimanus	LC	25.494	3.2769
1050	Primates	Cercopithecidae	Cercopithecus hamlyni	NT	12.223	3.2751
1051	Artiodactyla	Cervidae	Blastocerus dichotomus	VU	5.601	3.2736
1052	Chiroptera	Phyllostomidae	Phyllonycteris poeyi	NT	12.201	3.2734
1053	Chiroptera	Vespertilionidae	Murina ussuriensis	EN	2.286	3.2690
1054	Insectivora	Talpidae	Uropsilus andersoni	LC	25.261	3.2681
1054	Insectivora	Talpidae	Uropsilus gracilis	LC	25.261	3.2681
1056	Chiroptera	Vespertilionidae	Hesperoptenus doriae	EN	2.280	3.2673

Rank	Order	Family	Species	IUCN	ED'	EDGE
1057	Primates	Cercopithecidae	Presbytis potenziani	VU	5.540	3.2642
1058	Rodentia	Myoxidae	Eliomys melanurus	LC	25.014	3.2586
1059	Xenarthra	Megalonychidae	Choloepus didactylus	LC	24.951	3.2562
1059	Xenarthra	Megalonychidae	Choloepus hoffmanni	LC	24.951	3.2562
1061	Chiroptera	Phyllostomidae	Phyllostomus latifolius	NT	11.974	3.2561
1062	Carnivora	Procyonidae	Bassaricyon beddardi	NT	11.965	3.2554
1062	Carnivora	Procyonidae	Bassaricyon gabbii	NT	11.965	3.2554
1064	Diprotodontia	Potoroidae	Bettongia penicillata	CD	11.960	3.2550
1064	Diprotodontia	Potoroidae	Bettongia gaimardi	NT	11.960	3.2550
1066	Rodentia	Muridae	Cricetulus migratorius	NT	11.955	3.2547
1067	Rodentia	Muridae	Pseudomys australis	VU	5.469	3.2533
1067	Rodentia	Muridae	Pseudomys fumeus	VU	5.469	3.2533
1067	Rodentia	Muridae	Pseudomys patrius	VU	5.469	3.2533
1067	Rodentia	Muridae	Pseudomys pilligaensis	VU	5.469	3.2533
1067	Rodentia	Muridae	Pseudomys praeconis	VU	5.469	3.2533
1072	Artiodactyla	Bovidae	Cephalophus spadix	VU	5.432	3.2476
1073	Primates	Cercopithecidae	Pygathrix roxellana	VU	5.429	3.2471
1074	Rodentia	Muridae	Microdillus peeli	LC	24.575	3.2416
1075	Chiroptera	Emballonuridae	Taphozous hilli	LC	24.553	3.2408
1076	Rodentia	Dipodidae	Sicista betulina	NT	11.769	3.2401
1076	Rodentia	Dipodidae	Sicista subtilis	NT	11.769	3.2401
1078	Rodentia	Muridae	Myopus schisticolor	NT	11.745	3.2383
1079	Chiroptera	Vespertilionidae	Myotis frater	NT	11.735	3.2375
1080	Artiodactyla	Bovidae	Ovis aries	VU	5.347	3.2342
1081	Rodentia	Bathyergidae	Heliophobius argenteocinereus	LC	24.376	3.2338
1082	Carnivora	Otariidae	Arctocephalus galapagoensis	VU	5.329	3.2315
1082	Carnivora	Otariidae	Arctocephalus philippii	VU	5.329	3.2315
1084	Chiroptera	Emballonuridae	Diclidurus scutatus	LC	24.278	3.2300
1085	Rodentia	Muridae	Komodomys rintjanus	VU	5.317	3.2296
1085	Rodentia	Muridae	Papagomys armandvillei	VU	5.317	3.2296
1085	Rodentia	Muridae	Phloeomys cumingi	VU	5.317	3.2296
1085	Rodentia	Muridae	Vandeleuria nolthenii	VU	5.317	3.2296
1089	Chiroptera	Vespertilionidae	Myotis ruber	VU	5.316	3.2294
1090	Rodentia	Sciuridae	Tamias canipes	NT	11.589	3.2260
1091	Chiroptera	Rhinopomatidae	Rhinopoma hardwickei	LC	24.177	3.2259
1091	Chiroptera	Rhinopomatidae	Rhinopoma microphyllum	LC	24.177	3.2259
1091	Chiroptera	Rhinopomatidae	Rhinopoma muscatellum	LC	24.177	3.2259
1094	Artiodactyla	Bovidae	Cephalophus zebra	VU	5.286	3.2246
1095	Chiroptera	Vespertilionidae	Myotis atacamensis	VU	5.264	3.2212
1095	Chiroptera	Vespertilionidae	Myotis dominicensis	VU	5.264	3.2212
1097	Artiodactyla	Antilocapridae	Antilocapra americana	LC	24.038	3.2204
1098	Artiodactyla	Cervidae	Cervus albirostris	VU	5.259	3.2204
1098	Artiodactyla	Cervidae	Cervus duvaucelii	VU	5.259	3.2204
1098	Artiodactyla	Cervidae	Cervus eldii	VU	5.259	3.2204
1101	Primates	Hylobatidae	Hylobates syndactylus	NT	11.504	3.2192
1102	Chiroptera	Molossidae	Mops congicus	NT	11.496	3.2185
1102	Chiroptera	Molossidae	Mops sarasinorum	NT	11.496	3.2185
1104	Rodentia	Muridae	Oligoryzomys victus	EN	2.123	3.2184
1105	Diprotodontia	Macropodidae	Macropus bernardus	NT	11.451	3.2150
1106	Chiroptera	Vespertilionidae	Myotis capaccinii	VU	5.220	3.2140
1106	Chiroptera	Vespertilionidae	Myotis longipes	VU	5.220	3.2140
1108	Chiroptera	Phyllostomidae	Ariteus flavescens	VU	5.213	3.2129
1109	Diprotodontia	Macropodidae	Dendrolagus lumholtzi	NT	11.423	3.2127
1110	Chiroptera	Phyllostomidae	Chiroderma doriae	VU	5.203	3.2113
1111	Rodentia	Sciuridae	Lariscus niobe	NT	11.393	3.2103
1111	Rodentia	Sciuridae	Lariscus obscurus	NT	11.393	3.2103

Rank	Order	Family	Species	IUCN	ED'	EDGE
1113	Insectivora	Soricidae	Nectogale elegans	LC	23.762	3.2093
1114	Artiodactyla	Camelidae	Lama guanicoe	LC	23.754	3.2090
1115	Chiroptera	Megadermatidae	Megaderma lyra	LC	23.748	3.2087
1115	Chiroptera	Megadermatidae	Megaderma spasma	LC	23.748	3.2087
1117	Chiroptera	Phyllostomidae	Platyrrhinus chocoensis	VU	5.181	3.2078
1117	Chiroptera	Phyllostomidae	Platyrrhinus recifinus	VU	5.181	3.2078
1119	Rodentia	Muridae	Oecomys cleberi	EN	2.086	3.2063
1120	Dasyuromorphia	Dasyuridae	Dasyurus albopunctatus	VU	5.169	3.2058
1121	Rodentia	Dipodidae	Paradipus ctenodactylus	LC	23.591	3.2024
1122	Rodentia	Sciuridae	Spermophilus citellus	VU	5.136	3.2005
1123	Primates	Cercopithecidae	Cercopithecus solatus	VU	5.130	3.1996
1124	Artiodactyla	Bovidae	Capra cylindricornis	VU	5.129	3.1993
1124	Artiodactyla	Bovidae	Capra hircus	VU	5.129	3.1993
1126	Rodentia	Muridae	Chibchanomys trichotis	NT	11.258	3.1993
1127	Lagomorpha	Ochotonidae	Ochotona forresti	NT	11.255	3.1991
1128	Primates	Cebidae	Alouatta coibensis	VU	5.123	3.1983
1129	Carnivora	Canidae	Vulpes cana	VU	5.120	3.1978
1130	Chiroptera	Emballonuridae	Peropteryx leucoptera	LC	23.468	3.1974
1131	Perissodactyla	Equidae	Equus burchellii	LC	23.439	3.1962
1132	Artiodactyla	Bovidae	Rupicapra pyrenaica	CD	11.186	3.1934
1133	Chiroptera	Molossidae	Eumops glaucinus	LC	23.265	3.1890
1134	Chiroptera	Phyllostomidae	Artibeus fraterculus	VU	5.058	3.1876
1135	Rodentia	Heteromyidae	Chaetodipus formosus	LC	23.219	3.1872
1136	Rodentia	Echimyidae	Echimys chrysurus	VU	5.053	3.1868
1136	Rodentia	Echimyidae	Echimys thomasi	VU	5.053	3.1868
1138	Rodentia	Dipodidae	Zapus trinotatus	NT	11.098	3.1862
1139	Rodentia	Geomyidae	Thomomys mazama	NT	11.090	3.1856
1140	Rodentia	Muridae	Xenuromys barbatus	NT	11.078	3.1845
1141	Chiroptera	Emballonuridae	Taphozous nudiventris	LC	23.119	3.1830
1142	Didelphimorphia	Didelphidae	Lutreolina crassicaudata	LC	23.113	3.1827
1143	Primates	Cercopithecidae	Procolobus verus	NT	11.045	3.1818
1144	Carnivora	Viverridae	Nandinia binotata	LC	23.070	3.1810
1145	Perissodactyla	Equidae	Equus kiang	LC	23.022	3.1790
1146	Chiroptera	Phyllostomidae	Desmodus rotundus	LC	22.966	3.1766
1146	Chiroptera	Phyllostomidae	Diaemus youngi	LC	22.966	3.1766
1148	Artiodactyla	Bovidae	Pelea capreolus	LC	22.955	3.1762
1149	Rodentia	Heteromyidae	Dipodomys elator	VU	4.986	3.1757
1150	Artiodactyla	Tragulidae	Moschiola meminna	LC	22.942	3.1756
1151	Dasyuromorphia	Dasyuridae	Antechinus godmani	NT	10.967	3.1753
1152	Rodentia	Muridae	Ochrotomys nuttalli	LC	22.918	3.1746
1153	Diprotodontia	Pseudocheiridae	Petropseudes dahli	LC	22.887	3.1733
1154	Carnivora	Mustelidae	Lyncodon patagonicus	LC	22.813	3.1702
1155	Artiodactyla	Suidae	Phacochoerus aethiopicus	LC	22.746	3.1674
1155	Artiodactyla	Suidae	Phacochoerus africanus	LC	22.746	3.1674
1157	Chiroptera	Molossidae	Mormopterus setiger	LC	22.714	3.1661
1158	Chiroptera	Vespertilionidae	Nyctophilus microdon	VU	4.925	3.1655
1158	Chiroptera	Vespertilionidae	Nyctophilus timoriensis	VU	4.925	3.1655
1160	Diprotodontia	Pseudocheiridae	Pseudochirops albertisii	LC	22.675	3.1644
1161	Rodentia	Echimyidae	Clyomys bishopi	VU	4.904	3.1619
1162	Artiodactyla	Bovidae	Taurotragus oryx	CD	10.806	3.1618
1162	Artiodactyla	Bovidae	Taurotragus derbianus	NT	10.806	3.1618
1164	Chiroptera	Vespertilionidae	Pipistrellus cadornae	NT	10.792	3.1605
1164	Chiroptera	Vespertilionidae	Pipistrellus macrotis	NT	10.792	3.1605
1166	Rodentia	Sciuridae	Glyphotes simus	LC	22.543	3.1588
1167	Chiroptera	Molossidae	Mops demonstrator	NT	10.742	3.1563

Rank	Order	Family	Species	IUCN	ED'	EDGE
1168	Chiroptera	Pteropodidae	Pteropus nitendiensis	VU	4.863	3.1550
1168	Chiroptera	Pteropodidae	Pteropus tuberculatus	VU	4.863	3.1550
1170	Diprotodontia	Potoroidae	Aepyprymnus rufescens	LC	22.415	3.1534
1171	Diprotodontia	Acrobatidae	Acrobates pygmaeus	LC	22.389	3.1523
1171	Diprotodontia	Acrobatidae	Distoechurus pennatus	LC	22.389	3.1523
1173	Rodentia	Muridae	Melomys fellowsi	VU	4.846	3.1520
1174	Diprotodontia	Pseudocheiridae	Pseudochirops cupreus	LC	22.379	3.1519
1175	Artiodactyla	Tragulidae	Tragulus javanicus	LC	22.374	3.1516
1175	Artiodactyla	Tragulidae	Tragulus napu	LC	22.374	3.1516
1177	Carnivora	Mustelidae	Melogale orientalis	NT	10.668	3.1500
1178	Rodentia	Muridae	Saccostomus campestris	LC	22.316	3.1491
1178	Rodentia	Muridae	Saccostomus mearnsi	LC	22.316	3.1491
1180	Primates	Cheirogaleidae	Microcebus murinus	LC	22.299	3.1484
1180	Primates	Cheirogaleidae	Microcebus rufus	LC	22.299	3.1484
1182	Chiroptera	Pteropodidae	Pteropus niger	VU	4.813	3.1464
1182	Chiroptera	Pteropodidae	Pteropus rufus	VU	4.813	3.1464
1182	Chiroptera	Pteropodidae	Pteropus voeltzkowi	VU	4.813	3.1464
1185	Rodentia	Muridae	Reithrodontomys hirsutus	NT	10.621	3.1459
1186	Chiroptera	Phyllostomidae	Artibeus hirsutus	VU	4.808	3.1455
1186	Chiroptera	Phyllostomidae	Artibeus inopinatus	VU	4.808	3.1455
1188	Chiroptera	Rhinolophidae	Rhinolophus lepidus	LC	22.193	3.1439
1189	Cetacea	Ziphiidae	Berardius arnuxii	CD	10.572	3.1417
1189	Cetacea	Ziphiidae	Berardius bairdii	CD	10.572	3.1417
1189	Cetacea	Ziphiidae	Hyperoodon ampullatus	CD	10.572	3.1417
1189	Cetacea	Ziphiidae	Hyperoodon planifrons	CD	10.572	3.1417
1193	Chiroptera	Molossidae	Chaerephon aloysiisabaudiae	NT	10.569	3.1415
1193	Chiroptera	Molossidae	Chaerephon johorensis	NT	10.569	3.1415
1193	Chiroptera	Molossidae	Chaerephon russata	NT	10.569	3.1415
1196	Carnivora	Mustelidae	Mustela strigidorsa	VU	4.769	3.1388
1197	Chiroptera	Natalidae	Natalus micropus	LC	22.069	3.1385
1197	Chiroptera	Natalidae	Natalus stramineus	LC	22.069	3.1385
1197	Chiroptera	Natalidae	Natalus tumidirostris	LC	22.069	3.1385
1200	Rodentia	Sciuridae	Spermophilus franklinii	VU	4.757	3.1368
1201	Chiroptera	Molossidae	Eumops hansae	LC	22.028	3.1367
1201	Chiroptera	Molossidae	Eumops perotis	LC	22.028	3.1367
1203	Chiroptera	Vespertilionidae	Murina puta	VU	4.752	3.1358
1204	Chiroptera	Pteropodidae	Thoopterus nigrescens	NT	10.478	3.1336
1205	Dasyuromorphia	Dasyuridae	Dasyurus geoffroii	VU	4.738	3.1335
1205	Dasyuromorphia	Dasyuridae	Dasyurus spartacus	VU	4.738	3.1335
1207	Insectivora	Soricidae	Suncus dayi	VU	4.733	3.1326
1207	Insectivora	Soricidae	Suncus hosei	VU	4.733	3.1326
1207	Insectivora	Soricidae	Suncus montanus	VU	4.733	3.1326
1210	Rodentia	Muridae	Rattus baluensis	EN	1.865	3.1319
1211	Pholidota	Manidae	Manis gigantea	LC	21.837	3.1284
1211	Pholidota	Manidae	Manis tetradactyla	LC	21.837	3.1284
1211	Pholidota	Manidae	Manis tricuspis	LC	21.837	3.1284
1214	Diprotodontia	Macropodidae	Macropus irma	NT	10.411	3.1277
1215	Artiodactyla	Bovidae	Hippotragus equinus	CD	10.408	3.1275
1215	Artiodactyla	Bovidae	Hippotragus niger	CD	10.408	3.1275
1217	Carnivora	Phocidae	Erignathus barbatus	LC	21.741	3.1242
1218	Artiodactyla	Bovidae	Naemorhedus baileyi	VU	4.685	3.1242
1218	Artiodactyla	Bovidae	Naemorhedus caudatus	VU	4.685	3.1242
1218	Artiodactyla	Bovidae	Naemorhedus sumatraensis	VU	4.685	3.1242
1218	Artiodactyla	Bovidae	Naemorhedus swinhoei	VU	4.685	3.1242
1222	Rodentia	Sciuridae	Rubrisciurus rubriventer	LC	21.733	3.1238

Rank	Order	Family	Species	IUCN	ED'	EDGE
1223	Insectivora	Talpidae	Scapanus orarius	LC	21.691	3.1220
1223	Insectivora	Talpidae	Scapanus townsendii	LC	21.691	3.1220
1225	Chiroptera	Mormoopidae	Pteronotus personatus	LC	21.659	3.1206
1226	Rodentia	Muridae	Tylomys fulviventer	NT	10.311	3.1189
1227	Primates	Megaladapidae	Lepilemur edwardsi	NT	10.249	3.1134
1227	Primates	Megaladapidae	Lepilemur leucopus	NT	10.249	3.1134
1227	Primates	Megaladapidae	Lepilemur microdon	NT	10.249	3.1134
1227	Primates	Megaladapidae	Lepilemur mustelinus	NT	10.249	3.1134
1227	Primates	Megaladapidae	Lepilemur ruficaudatus	NT	10.249	3.1134
1232	Rodentia	Muridae	Brachytarsomys albicauda	LC	21.493	3.1132
1233	Rodentia	Sciuridae	Petaurista magnificus	NT	10.202	3.1093
1234	Cetacea	Phocoenidae	Phocoenoides dalli	CD	10.167	3.1061
1235	Chiroptera	Pteropodidae	Boneia bidens	NT	10.138	3.1035
1236	Insectivora	Soricidae	Blarinella quadraticauda	LC	21.266	3.1031
1237	Didelphimorphia	Didelphidae	Didelphis virginiana	LC	21.254	3.1025
1238	Primates	Tarsiidae	Tarsius bancanus	LC	21.234	3.1016
1239	Chiroptera	Emballonuridae	Diclidurus albus	LC	21.217	3.1009
1240	Didelphimorphia	Didelphidae	Didelphis albiventris	LC	21.163	3.0984
1241	Chiroptera	Phyllostomidae	Micronycteris brachyotis	LC	21.095	3.0953
1242	Didelphimorphia	Didelphidae	Didelphis aurita	LC	21.088	3.0950
1242	Didelphimorphia	Didelphidae	Didelphis marsupialis	LC	21.088	3.0950
1244	Rodentia	Sciuridae	Atlantoxerus getulus	LC	21.087	3.0950
1245	Rodentia	Muridae	Notomys aquilo	NT	10.035	3.0942
1245	Rodentia	Muridae	Notomys cervinus	NT	10.035	3.0942
1247	Primates	Galagonidae	Galago moholi	LC	21.062	3.0938
1247	Primates	Galagonidae	Galago senegalensis	LC	21.062	3.0938
1249	Artiodactyla	Moschidae	Moschus chrysogaster	NT	10.018	3.0927
1249	Artiodactyla	Moschidae	Moschus fuscus	NT	10.018	3.0927
1251	Rodentia	Sciuridae	Aeretes melanopterus	NT	9.998	3.0909
1252	Rodentia	Octodontidae	Octodon bridgesi	LC	20.951	3.0888
1252	Rodentia	Octodontidae	Octodon degus	LC	20.951	3.0888
1252	Rodentia	Octodontidae	Octodon lunatus	LC	20.951	3.0888
1255	Rodentia	Muridae	Hapalomys delacouri	NT	9.975	3.0887
1255	Rodentia	Muridae	Hapalomys longicaudatus	NT	9.975	3.0887
1257	Rodentia	Muridae	Dasymys foxi	VU	4.487	3.0887
1257	Rodentia	Muridae	Dasymys montanus	VU	4.487	3.0887
1259	Chiroptera	Phyllostomidae	Sturnira aratathomasi	NT	9.961	3.0874
1260	Rodentia	Sciuridae	Sciurotamias davidianus	LC	20.903	3.0866
1261	Chiroptera	Vespertilionidae	Myotis emarginatus	VU	4.475	3.0864
1261	Chiroptera	Vespertilionidae	Myotis morrisi	VU	4.475	3.0864
1263	Chiroptera	Vespertilionidae	Eptesicus innoxius	VU	4.472	3.0860
1264	Rodentia	Muridae	Megadontomys cryophilus	NT	9.942	3.0858
1265	Chiroptera	Emballonuridae	Peropteryx kappleri	LC	20.869	3.0851
1265	Chiroptera	Emballonuridae	Peropteryx macrotis	LC	20.869	3.0851
1267	Rodentia	Sciuridae	Callosciurus prevostii	LC	20.831	3.0833
1268	Chiroptera	Pteropodidae	Pteropus fundatus	VU	4.445	3.0810
1268	Chiroptera	Pteropodidae	Pteropus pohlei	VU	4.445	3.0810
1270	Rodentia	Sciuridae	Spermophilus washingtoni	VU	4.444	3.0809
1271	Chiroptera	Molossidae	Nyctinomops aurispinosus	LC	20.757	3.0799
1271	Chiroptera	Molossidae	Nyctinomops femorosaccus	LC	20.757	3.0799
1271	Chiroptera	Molossidae	Nyctinomops laticaudatus	LC	20.757	3.0799
1271	Chiroptera	Molossidae	Nyctinomops macrotis	LC	20.757	3.0799
1275	Rodentia	Sciuridae	Tamias striatus	LC	20.747	3.0795
1276	Chiroptera	Phyllostomidae	Chrotopterus auritus	LC	20.647	3.0749
1277	Rodentia	Sciuridae	Callosciurus melanogaster	LC	20.617	3.0735

Rank	Order	Family	Species	IUCN	ED'	EDGE
1278	Rodentia	Muridae	Rattus elaphinus	VU	4.402	3.0731
1278	Rodentia	Muridae	Rattus feliceus	VU	4.402	3.0731
1280	Rodentia	Sciuridae	Hylopetes bartelsi	NT	9.795	3.0722
1281	Diprotodontia	Petauridae	Petaurus australis	NT	9.767	3.0696
1281	Diprotodontia	Petauridae	Petaurus norfolcensis	NT	9.767	3.0696
1283	Artiodactyla	Bovidae	Connochaetes taurinus	CD	9.737	3.0668
1284	Xenarthra	Bradypodidae	Bradypus tridactylus	LC	20.446	3.0655
1284	Xenarthra	Bradypodidae	Bradypus variegatus	LC	20.446	3.0655
1286	Rodentia	Sciuridae	Callosciurus phayrei	LC	20.430	3.0648
1287	Chiroptera	Vespertilionidae	Rhogeessa genowaysi	VU	4.349	3.0632
1288	Artiodactyla	Suidae	Hylochoerus meinertzhageni	LC	20.389	3.0629
1289	Rodentia	Muridae	Habromys lepturus	NT	9.684	3.0619
1290	Rodentia	Muridae	Aethomys stannarius	NT	9.675	3.0610
1291	Cetacea	Delphinidae	Globicephala macrorhynchus	CD	9.665	3.0601
1292	Macroscelidea	Macroscelididae	Elephantulus edwardii	LC	20.322	3.0597
1292	Macroscelidea	Macroscelididae	Elephantulus myurus	LC	20.322	3.0597
1294	Chiroptera	Vespertilionidae	Pipistrellus pulveratus	NT	9.658	3.0595
1294	Chiroptera	Vespertilionidae	Scotoecus pallidus	NT	9.658	3.0595
1296	Rodentia	Sciuridae	Pteromyscus pulverulentus	NT	9.650	3.0587
1297	Rodentia	Muridae	Akodon siberiae	VU	4.325	3.0586
1298	Rodentia	Muridae	Myomys albipes	NT	9.606	3.0545
1299	Artiodactyla	Bovidae	Alcelaphus buselaphus	CD	9.600	3.0540
1299	Artiodactyla	Bovidae	Sigmoceros lichtensteinii	CD	9.600	3.0540
1301	Insectivora	Soricidae	Blarina hylophaga	LC	20.178	3.0530
1302	Rodentia	Sciuridae	Callosciurus baluensis	LC	20.142	3.0513
1303	Insectivora	Soricidae	Crocidura dhofarensis	CR	0.320	3.0502
1303	Insectivora	Soricidae	Crocidura harenna	CR	0.320	3.0502
1303	Insectivora	Soricidae	Crocidura negrina	CR	0.320	3.0502
1303	Insectivora	Soricidae	Crocidura thomensis	CR	0.320	3.0502
1303	Insectivora	Soricidae	Crocidura wimmeri	CR	0.320	3.0502
1308	Rodentia	Myocastoridae	Myocastor coypus	LC	20.112	3.0499
1309	Chiroptera	Pteropodidae	Pteropus ocularis	VU	4.254	3.0453
1310	Rodentia	Sciuridae	Callosciurus inornatus	LC	20.014	3.0452
1311	Rodentia	Heteromyidae	Perognathus alticola	NT	9.494	3.0440
1311	Rodentia	Heteromyidae	Perognathus amplus	NT	9.494	3.0440
1313	Chiroptera	Phyllostomidae	Ectophylla alba	NT	9.482	3.0428
1314	Rodentia	Muridae	Microhydromys richardsoni	NT	9.465	3.0411
1315	Chiroptera	Molossidae	Eumops auripendulus	LC	19.913	3.0404
1316	Rodentia	Sciuridae	Callosciurus notatus	LC	19.890	3.0393
1317	Rodentia	Chinchillidae	Lagostomus maximus	LC	19.851	3.0374
1318	Rodentia	Sciuridae	Callosciurus adamsi	LC	19.820	3.0359
1319	Carnivora	Felidae	Otocolobus manul	NT	9.391	3.0341
1320	Diprotodontia	Macropodidae	Lagorchestes conspicillatus	NT	9.370	3.0321
1321	Rodentia	Sciuridae	Callosciurus orestes	LC	19.736	3.0319
1322	Chiroptera	Phyllostomidae	Tonatia evotis	NT	9.348	3.0300
1323	Chiroptera	Rhinolophidae	Triaenops persicus	LC	19.640	3.0272
1324	Rodentia	Muridae	Chionomys gud	NT	9.311	3.0263
1324	Rodentia	Muridae	Chionomys nivalis	NT	9.311	3.0263
1324	Rodentia	Muridae	Chionomys roberti	NT	9.311	3.0263
1327	Rodentia	Sciuridae	Callosciurus caniceps	LC	19.597	3.0252
1327	Rodentia	Sciuridae	Callosciurus finlaysonii	LC	19.597	3.0252
1329	Chiroptera	Emballonuridae	Balantiopteryx plicata	LC	19.570	3.0238
1330	Chiroptera	Vespertilionidae	Lasiurus egregius	NT	9.282	3.0235
1331	Chiroptera	Phyllostomidae	Trachops cirrhosus	LC	19.530	3.0219
1332	Artiodactyla	Bovidae	Gazella spekei	VU	4.130	3.0214
1333	Dasyuromorphia	Dasyuridae	Phascogale tapoatafa	NT	9.250	3.0205

Rank	Order	Family	Species	IUCN	ED'	EDGE
1334	Rodentia	Muridae	Osgoodomys banderanus	LC	19.486	3.0197
1335	Artiodactyla	Bovidae	Aepyceros melampus	CD	9.232	3.0187
1335	Artiodactyla	Bovidae	Neotragus moschatus	CD	9.232	3.0187
1335	Artiodactyla	Bovidae	Oreotragus oreotragus	CD	9.232	3.0187
1335	Artiodactyla	Bovidae	Ourebia ourebi	CD	9.232	3.0187
1335	Artiodactyla	Bovidae	Neotragus batesi	NT	9.232	3.0187
1335	Artiodactyla	Bovidae	Neotragus pygmaeus	NT	9.232	3.0187
1341	Insectivora	Soricidae	Sorex leucogaster	VU	4.113	3.0181
1342	Chiroptera	Emballonuridae	Taphozous longimanus	LC	19.452	3.0181
1342	Chiroptera	Emballonuridae	Taphozous mauritianus	LC	19.452	3.0181
1342	Chiroptera	Emballonuridae	Taphozous perforatus	LC	19.452	3.0181
1345	Lagomorpha	Leporidae	Pronolagus randensis	LC	19.437	3.0173
1346	Chiroptera	Rhinolophidae	Coelops frithi	LC	19.435	3.0172
1347	Chiroptera	Vespertilionidae	Chalinolobus picatus	NT	9.216	3.0172
1348	Rodentia	Ctenodactylidae	Massoutiera mzabi	LC	19.431	3.0170
1349	Chiroptera	Mormoopidae	Pteronotus parnellii	LC	19.409	3.0160
1350	Chiroptera	Rhinolophidae	Hipposideros breviceps	VU	4.100	3.0155
1350	Chiroptera	Rhinolophidae	Hipposideros coxi	VU	4.100	3.0155
1350	Chiroptera	Rhinolophidae	Hipposideros curtus	VU	4.100	3.0155
1350	Chiroptera	Rhinolophidae	Hipposideros papua	VU	4.100	3.0155
1350	Chiroptera	Rhinolophidae	Hipposideros ridleyi	VU	4.100	3.0155
1355	Rodentia	Echimyidae	Isothrix bistriata	NT	9.192	3.0148
1355	Rodentia	Echimyidae	Isothrix pagurus	NT	9.192	3.0148
1357	Rodentia	Sciuridae	Tamias sibiricus	LC	19.371	3.0141
1358	Rodentia	Muridae	Juscelinomys talpinus	VU	4.084	3.0124
1359	Insectivora	Soricidae	Blarina brevicauda	LC	19.328	3.0120
1359	Insectivora	Soricidae	Blarina carolinensis	LC	19.328	3.0120
1361	Scandentia	Tupaiidae	Dendrogale murina	LC	19.299	3.0106
1362	Lagomorpha	Leporidae	Pronolagus crassicaudatus	LC	19.287	3.0100
1362	Lagomorpha	Leporidae	Pronolagus rupestris	LC	19.287	3.0100
1364	Rodentia	Sciuridae	Exilisciurus concinnus	LC	19.255	3.0084
1365	Chiroptera	Emballonuridae	Saccopteryx bilineata	LC	19.193	3.0053
1365	Chiroptera	Emballonuridae	Saccopteryx leptura	LC	19.193	3.0053
1367	Rodentia	Muridae	Rattus hoogerwerfi	VU	4.036	3.0030
1367	Rodentia	Muridae	Rattus ranjiniae	VU	4.036	3.0030
1367	Rodentia	Muridae	Rattus stoicus	VU	4.036	3.0030
1370	Chiroptera	Phyllostomidae	Anoura latidens	NT	9.070	3.0027
1371	Artiodactyla	Bovidae	Gazella dorcas	VU	4.030	3.0017
1371	Artiodactyla	Bovidae	Gazella gazella	VU	4.030	3.0017
1373	Rodentia	Geomyidae	Geomys arenarius	NT	9.058	3.0016
1374	Diprotodontia	Pseudocheiridae	Pseudocheirus peregrinus	LC	19.108	3.0011
1375	Primates	Cercopithecidae	Macaca fascicularis	NT	9.053	3.0010
1376	Rodentia	Muridae	Mesembriomys gouldii	NT	9.014	2.9971
1377	Chiroptera	Emballonuridae	Saccopteryx canescens	LC	19.001	2.9958
1378	Rodentia	Muridae	Rhynchomys isarogensis	VU	3.999	2.9956
1379	Insectivora	Chrysochloridae	Amblysomus hottentotus	LC	18.981	2.9948
1379	Insectivora	Chrysochloridae	Amblysomus iris	LC	18.981	2.9948
1381	Rodentia	Muridae	Nesomys rufus	LC	18.972	2.9943
1382	Carnivora	Viverridae	Arctogalidia trivirgata	LC	18.929	2.9922
1383	Rodentia	Muridae	Microtus cabrerae	NT	8.962	2.9919
1384	Insectivora	Soricidae	Megasorex gigas	LC	18.902	2.9908
1384	Insectivora	Soricidae	Notiosorex crawfordi	LC	18.902	2.9908
1386	Chiroptera	Molossidae	Tadarida aegyptiaca	LC	18.897	2.9906
1387	Rodentia	Muridae	Cricetomys emini	LC	18.897	2.9906
1387	Rodentia	Muridae	Cricetomys gambianus	LC	18.897	2.9906

Rank	Order	Family	Species	IUCN	ED'	EDGE
1389	Didelphimorphia	Didelphidae	Thylamys elegans	LC	18.881	2.9898
1389	Didelphimorphia	Didelphidae	Thylamys pallidior	LC	18.881	2.9898
1391	Artiodactyla	Bovidae	Antilope cervicapra	NT	8.932	2.9890
1392	Diprotodontia	Burramyidae	Cercartetus concinnus	LC	18.865	2.9889
1392	Diprotodontia	Burramyidae	Cercartetus lepidus	LC	18.865	2.9889
1392	Diprotodontia	Burramyidae	Cercartetus nanus	LC	18.865	2.9889
1395	Primates	Cebidae	Callicebus personatus	NT	8.925	2.9882
1396	Carnivora	Mustelidae	Eira barbara	LC	18.804	2.9859
1397	Rodentia	Sciuridae	Petaurista nobilis	NT	8.887	2.9844
1398	Chiroptera	Emballonuridae	Emballonura beccarii	LC	18.754	2.9833
1399	Chiroptera	Pteropodidae	Cynopterus nusatenggara	NT	8.852	2.9808
1400	Artiodactyla	Bovidae	Gazella soemmerringii	VU	3.925	2.9806
1401	Chiroptera	Phyllostomidae	Micronycteris nicefori	LC	18.592	2.9751
1402	Rodentia	Muridae	Mallomys istapantap	NT	8.778	2.9732
1403	Chiroptera	Emballonuridae	Taphozous georgianus	LC	18.528	2.9719
1404	Chiroptera	Vespertilionidae	Euderma maculatum	LC	18.524	2.9716
1404	Chiroptera	Vespertilionidae	Idionycteris phyllotis	LC	18.524	2.9716
1406	Chiroptera	Vespertilionidae	Eptesicus brunneus	NT	8.759	2.9714
1406	Chiroptera	Vespertilionidae	Eptesicus guineensis	NT	8.759	2.9714
1408	Rodentia	Muridae	Abrawayaomys ruschii	EN	1.435	2.9696
1408	Rodentia	Muridae	Phaenomys ferrugineus	EN	1.435	2.9696
1410	Rodentia	Muridae	Cansumys canus	LC	18.468	2.9688
1410	Rodentia	Muridae	Tscherskia triton	LC	18.468	2.9688
1412	Xenarthra	Dasypodidae	Cabassous tatouay	LC	18.467	2.9687
1412	Xenarthra	Dasypodidae	Cabassous unicinctus	LC	18.467	2.9687
1414	Chiroptera	Emballonuridae	Saccolaimus saccolaimus	LC	18.455	2.9681
1415	Rodentia	Capromyidae	Mysateles melanurus	NT	8.726	2.9679
1415	Rodentia	Capromyidae	Mysateles meridionalis	NT	8.726	2.9679
1417	Rodentia	Muridae	Solomys salebrosus	NT	8.716	2.9669
1418	Chiroptera	Vespertilionidae	Myotis pequinius	NT	8.715	2.9668
1419	Primates	Callitrichidae	Callithrix geoffroyi	VU	3.853	2.9658
1420	Carnivora	Procyonidae	Potos flavus	LC	18.389	2.9647
1421	Insectivora	Erinaceidae	Atelerix frontalis	LC	18.387	2.9646
1422	Rodentia	Muridae	Nyctomys sumichrasti	LC	18.337	2.9620
1422	Rodentia	Muridae	Otonyctomys hatti	LC	18.337	2.9620
1424	Rodentia	Ctenodactylidae	Ctenodactylus gundi	LC	18.301	2.9602
1425	Chiroptera	Vespertilionidae	Myotis hosonoi	VU	3.824	2.9599
1425	Chiroptera	Vespertilionidae	Myotis yesoensis	VU	3.824	2.9599
1427	Insectivora	Chrysochloridae	Calcochloris obtusirostris	LC	18.253	2.9577
1428	Rodentia	Hystricidae	Atherurus africanus	LC	18.251	2.9576
1428	Rodentia	Hystricidae	Atherurus macrourus	LC	18.251	2.9576
1430	Rodentia	Sciuridae	Hylopetes fimbriatus	NT	8.597	2.9546
1431	Artiodactyla	Cervidae	Alces alces	LC	18.152	2.9524
1432	Chiroptera	Vespertilionidae	Histiotus macrotus	NT	8.567	2.9515
1432	Chiroptera	Vespertilionidae	Laephotis angolensis	NT	8.567	2.9515
1434	Dasyuromorphia	Dasyuridae	Antechinus leo	NT	8.559	2.9507
1435	Carnivora	Herpestidae	Atilax paludinosus	LC	18.117	2.9506
1435	Carnivora	Herpestidae	Dologale dybowskii	LC	18.117	2.9506
1435	Carnivora	Herpestidae	Rhynchogale melleri	LC	18.117	2.9506
1438	Lagomorpha	Leporidae	Sylvilagus cunicularius	NT	8.549	2.9496
1439	Rodentia	Muridae	Rhombomys opimus	LC	18.076	2.9484
1440	Diprotodontia	Macropodidae	Petrogale xanthopus	NT	8.535	2.9481
1441	Chiroptera	Pteropodidae	Casinycteris argynnis	NT	8.521	2.9466
1442	Rodentia	Hystricidae	Hystrix crassispinis	NT	8.515	2.9460
1443	Artiodactyla	Bovidae	Gazella rufifrons	VU	3.757	2.9460
1444	Chiroptera	Phyllostomidae	Artibeus concolor	NT	8.510	2.9455

Rank	Order	Family	Species	IUCN	ED'	EDGE
1445	Rodentia	Muridae	Alticola montosa	VU	3.752	2.9449
1446	Insectivora	Chrysochloridae	Chrysochloris asiatica	LC	17.992	2.9440
1446	Insectivora	Chrysochloridae	Chrysochloris stuhlmanni	LC	17.992	2.9440
1448	Chiroptera	Pteropodidae	Dyacopterus spadiceus	NT	8.492	2.9435
1449	Chiroptera	Vespertilionidae	Eptesicus demissus	VU	3.744	2.9433
1450	Cetacea	Delphinidae	Stenella attenuata	CD	8.478	2.9422
1451	Primates	Cercopithecidae	Trachypithecus francoisi	VU	3.726	2.9394
1452	Carnivora	Felidae	Panthera onca	NT	8.446	2.9388
1453	Lagomorpha	Ochotonidae	Ochotona rufescens	LC	17.859	2.9370
1454	Chiroptera	Phyllostomidae	Vampyressa melissa	NT	8.427	2.9367
1455	Chiroptera	Vespertilionidae	Chalinolobus alboguttatus	VU	3.711	2.9363
1455	Chiroptera	Vespertilionidae	Chalinolobus superbus	VU	3.711	2.9363
1457	Chiroptera	Phyllostomidae	Artibeus fimbriatus	NT	8.421	2.9361
1458	Chiroptera	Emballonuridae	Taphozous melanopogon	LC	17.821	2.9350
1459	Rodentia	Sciuridae	Marmota menzbieri	VU	3.703	2.9345
1460	Rodentia	Muridae	Rhipidomys scandens	VU	3.700	2.9338
1461	Primates	Cercopithecidae	Cercocebus torquatus	NT	8.384	2.9321
1462	Rodentia	Muridae	Bunomys fratrorum	NT	8.375	2.9312
1462	Rodentia	Muridae	Bunomys heinrichi	NT	8.375	2.9312
1464	Rodentia	Muridae	Hydromys habbema	NT	8.369	2.9306
1464	Rodentia	Muridae	Hydromys hussoni	NT	8.369	2.9306
1464	Rodentia	Muridae	Hydromys shawmayeri	NT	8.369	2.9306
1467	Carnivora	Canidae	Cuon alpinus	VU	3.683	2.9302
1467	Carnivora	Canidae	Speothos venaticus	VU	3.683	2.9302
1469	Chiroptera	Molossidae	Mormopterus beccarii	LC	17.731	2.9302
1469	Chiroptera	Molossidae	Mormopterus norfolkensis	LC	17.731	2.9302
1469	Chiroptera	Molossidae	Mormopterus planiceps	LC	17.731	2.9302
1472	Chiroptera	Vespertilionidae	Pipistrellus bodenheimeri	NT	8.361	2.9297
1473	Chiroptera	Vespertilionidae	Nyctalus azoreum	VU	3.671	2.9277
1474	Cetacea	Delphinidae	Stenella coeruleoalba	CD	8.338	2.9272
1475	Carnivora	Mustelidae	Poecilogale albinucha	LC	17.642	2.9254
1476	Primates	Lemuridae	Hapalemur griseus	LC	17.638	2.9252
1477	Chiroptera	Emballonuridae	Coleura afra	LC	17.634	2.9250
1477	Chiroptera	Emballonuridae	Emballonura alecto	LC	17.634	2.9250
1477	Chiroptera	Emballonuridae	Emballonura monticola	LC	17.634	2.9250
1480	Didelphimorphia	Didelphidae	Marmosa mexicana	LC	17.618	2.9241
1480	Didelphimorphia	Didelphidae	Marmosa murina	LC	17.618	2.9241
1480	Didelphimorphia	Didelphidae	Marmosa robinsoni	LC	17.618	2.9241
1480	Didelphimorphia	Didelphidae	Marmosa rubra	LC	17.618	2.9241
1484	Rodentia	Muridae	Lophuromys melanonyx	VU	3.643	2.9216
1485	Carnivora	Felidae	Leopardus tigrinus	NT	8.279	2.9209
1485	Carnivora	Felidae	Oncifelis geoffroyi	NT	8.279	2.9209
1487	Carnivora	Mustelidae	Taxidea taxus	LC	17.554	2.9207
1488	Diprotodontia	Macropodidae	Macropus parma	NT	8.277	2.9207
1489	Rodentia	Muridae	Taeromys punicans	NT	8.263	2.9192
1490	Rodentia	Muridae	Uromys anak	NT	8.232	2.9158
1490	Rodentia	Muridae	Uromys hadrourus	NT	8.232	2.9158
1492	Chiroptera	Phyllostomidae	Glossophaga morenoi	NT	8.216	2.9141
1493	Rodentia	Muridae	Reithrodontomys paradoxus	NT	8.182	2.9103
1494	Insectivora	Chrysochloridae	Chlorotalpa arendsi	LC	17.363	2.9103
1494	Insectivora	Chrysochloridae	Chlorotalpa sclateri	LC	17.363	2.9103
1496	Chiroptera	Vespertilionidae	Pipistrellus mordax	NT	8.169	2.9090
1497	Chiroptera	Vespertilionidae	Myotis macrotarsus	NT	8.147	2.9066
1498	Primates	Cebidae	Cacajao calvus	NT	8.142	2.9060
1499	Rodentia	Muridae	Mastomys verheyeni	NT	8.103	2.9017
1500	Chiroptera	Phyllostomidae	Phylloderma stenops	LC	17.147	2.8985

Rank	Order	Family	Species	IUCN	ED'	EDGE
1501	Artiodactyla	Bovidae	Raphicerus melanotis	CD	8.070	2.8981
1502	Lagomorpha	Ochotonidae	Ochotona erythrotis	LC	17.133	2.8977
1503	Insectivora	Erinaceidae	Hemiechinus hypomelas	LC	17.130	2.8976
1504	Rodentia	Muridae	Mus mayori	NT	8.064	2.8975
1504	Rodentia	Muridae	Mus vulcani	NT	8.064	2.8975
1506	Macroscelidea	Macroscelididae	Elephantulus brachyrhynchus	LC	17.122	2.8971
1507	Rodentia	Muridae	Volemys clarkei	NT	8.054	2.8964
1508	Rodentia	Sciuridae	Spermophilus suslicus	VU	3.525	2.8960
1509	Artiodactyla	Bovidae	Antidorcas marsupialis	CD	8.032	2.8940
1509	Artiodactyla	Bovidae	Litocranius walleri	CD	8.032	2.8940
1511	Rodentia	Muridae	Malacomys lukolelae	NT	8.016	2.8921
1511	Rodentia	Muridae	Malacomys verschureni	NT	8.016	2.8921
1513	Chiroptera	Pteropodidae	Melonycteris melanops	LC	17.015	2.8912
1514	Artiodactyla	Bovidae	Bison bison	CD	8.000	2.8904
1515	Insectivora	Erinaceidae	Mesechinus dauuricus	LC	16.985	2.8895
1516	Chiroptera	Vespertilionidae	Barbastella leucomelas	LC	16.951	2.8876
1517	Rodentia	Abrocomidae	Abrocoma bennettii	LC	16.945	2.8873
1517	Rodentia	Abrocomidae	Abrocoma cinerea	LC	16.945	2.8873
1517	Rodentia	Hydrochaeridae	Hydrochaeris hydrochaeris	LC	16.945	2.8873
1517	Rodentia	Petromuridae	Petromus typicus	LC	16.945	2.8873
1521	Rodentia	Muridae	Reithrodontomys microdon	NT	7.966	2.8866
1522	Lagomorpha	Ochotonidae	Ochotona himalayana	LC	16.908	2.8853
1523	Rodentia	Sciuridae	Sundasciurus brookei	NT	7.929	2.8824
1523	Rodentia	Sciuridae	Sundasciurus moellendorffi	NT	7.929	2.8824
1523	Rodentia	Sciuridae	Sundasciurus steerii	NT	7.929	2.8824
1526	Primates	Cercopithecidae	Cercocebus galeritus	NT	7.919	2.8813
1527	Diprotodontia	Pseudocheiridae	Petauroides volans	LC	16.815	2.8800
1528	Rodentia	Muridae	Gerbillus bilensis	CR	0.111	2.8783
1528	Rodentia	Muridae	Gerbillus cosensi	CR	0.111	2.8783
1528	Rodentia	Muridae	Gerbillus quadrimaculatus	CR	0.111	2.8783
1531	Chiroptera	Vespertilionidae	Pipistrellus kitcheneri	NT	7.860	2.8747
1532	Carnivora	Felidae	Felis margarita	NT	7.852	2.8738
1533	Primates	Cercopithecidae	Macaca mulatta	NT	7.835	2.8719
1534	Rodentia	Dipodidae	Pygeretmus platyurus	LC	16.654	2.8710
1534	Rodentia	Dipodidae	Pygeretmus pumilio	LC	16.654	2.8710
1536	Rodentia	Muridae	Grammomys aridulus	NT	7.819	2.8700
1536	Rodentia	Muridae	Grammomys dryas	NT	7.819	2.8700
1538	Rodentia	Muridae	Thamnomys venustus	NT	7.815	2.8696
1539	Rodentia	Muridae	Chiropodomys muroides	NT	7.813	2.8694
1540	Lagomorpha	Leporidae	Poelagus marjorita	LC	16.587	2.8671
1541	Rodentia	Muridae	Ototylomys phyllotis	LC	16.531	2.8640
1542	Rodentia	Muridae	Ichthyomys hydrobates	NT	7.765	2.8639
1542	Rodentia	Muridae	Rheomys mexicanus	NT	7.765	2.8639
1544	Diprotodontia	Petauridae	Dactylopsila palpator	LC	16.502	2.8623
1544	Diprotodontia	Petauridae	Dactylopsila trivirgata	LC	16.502	2.8623
1546	Carnivora	Felidae	Oncifelis colocolo	NT	7.750	2.8622
1547	Rodentia	Muridae	Lasiopodomys fuscus	NT	7.749	2.8620
1548	Insectivora	Erinaceidae	Atelerix algirus	LC	16.497	2.8620
1549	Rodentia	Geomyidae	Geomys personatus	NT	7.748	2.8620
1550	Carnivora	Felidae	Lynx lynx	NT	7.729	2.8598
1551	Rodentia	Muridae	Eolagurus luteus	CD	7.726	2.8594
1552	Rodentia	Muridae	Arvicola sapidus	NT	7.708	2.8574
1552	Rodentia	Muridae	Blanfordimys bucharicus	NT	7.708	2.8574
1554	Chiroptera	Rhinolophidae	Hipposideros dinops	NT	7.672	2.8532
1554	Chiroptera	Rhinolophidae	Hipposideros lekaguli	NT	7.672	2.8532

Rank	Order	Family	Species	IUCN	ED'	EDGE
1556	Rodentia	Muridae	Baiomys musculus	LC	16.338	2.8529
1556	Rodentia	Muridae	Baiomys taylori	LC	16.338	2.8529
1558	Rodentia	Heteromyidae	Chaetodipus arenarius	LC	16.304	2.8509
1559	Lagomorpha	Leporidae	Sylvilagus audubonii	LC	16.291	2.8502
1559	Lagomorpha	Leporidae	Sylvilagus nuttallii	LC	16.291	2.8502
1561	Rodentia	Bathyergidae	Georychus capensis	LC	16.226	2.8464
1562	Dasyuromorphia	Dasyuridae	Sminthopsis granulipes	LC	16.205	2.8452
1563	Chiroptera	Pteropodidae	Dobsonia emersa	VU	3.301	2.8450
1563	Chiroptera	Pteropodidae	Dobsonia peroni	VU	3.301	2.8450
1565	Artiodactyla	Suidae	Potamochoerus larvatus	LC	16.153	2.8422
1565	Artiodactyla	Suidae	Potamochoerus porcus	LC	16.153	2.8422
1567	Chiroptera	Phyllostomidae	Vampyressa bidens	NT	7.568	2.8412
1567	Chiroptera	Phyllostomidae	Vampyressa brocki	NT	7.568	2.8412
1569	Rodentia	Sciuridae	Nannosciurus melanotis	LC	16.132	2.8409
1570	Chiroptera	Rhinolophidae	Hipposideros lylei	NT	7.565	2.8408
1570	Chiroptera	Rhinolophidae	Hipposideros pratti	NT	7.565	2.8408
1572	Rodentia	Muridae	Eliurus webbi	NT	7.541	2.8380
1573	Rodentia	Sciuridae	Callosciurus albescens	LC	16.078	2.8378
1573	Rodentia	Sciuridae	Callosciurus nigrovittatus	LC	16.078	2.8378
1575	Dasyuromorphia	Dasyuridae	Sminthopsis longicaudata	LC	16.062	2.8368
1576	Chiroptera	Phyllostomidae	Macrotus waterhousii	LC	16.058	2.8366
1577	Chiroptera	Rhinolophidae	Rhinolophus pusillus	LC	16.053	2.8363
1578	Carnivora	Felidae	Herpailurus yaguarondi	LC	16.044	2.8358
1579	Rodentia	Muridae	Macrotarsomys bastardi	LC	16.031	2.8350
1580	Chiroptera	Rhinolophidae	Hipposideros semoni	NT	7.513	2.8347
1580	Chiroptera	Rhinolophidae	Hipposideros stenotis	NT	7.513	2.8347
1580	Chiroptera	Rhinolophidae	Hipposideros wollastoni	NT	7.513	2.8347
1583	Rodentia	Muridae	Neotoma cinerea	LC	16.007	2.8336
1583	Rodentia	Muridae	Neotoma fuscipes	LC	16.007	2.8336
1585	Carnivora	Ursidae	Ursus americanus	LC	16.002	2.8333
1586	Primates	Cercopithecidae	Miopithecus talapoin	LC	15.992	2.8328
1587	Rodentia	Muridae	Pelomys campanae	LC	15.989	2.8326
1587	Rodentia	Muridae	Pelomys fallax	LC	15.989	2.8326
1587	Rodentia	Muridae	Pelomys minor	LC	15.989	2.8326
1590	Chiroptera	Pteropodidae	Pteropus howensis	VU	3.242	2.8314
1590	Chiroptera	Pteropodidae	Pteropus ornatus	VU	3.242	2.8314
1590	Chiroptera	Pteropodidae	Pteropus pumilus	VU	3.242	2.8314
1590	Chiroptera	Pteropodidae	Pteropus sanctacrucis	VU	3.242	2.8314
1590	Chiroptera	Pteropodidae	Pteropus speciosus	VU	3.242	2.8314
1595	Chiroptera	Pteropodidae	Scotonycteris zenkeri	NT	7.483	2.8312
1596	Rodentia	Muridae	Praomys delectorum	NT	7.473	2.8301
1596	Rodentia	Muridae	Praomys mutoni	NT	7.473	2.8301
1598	Rodentia	Muridae	Arvicanthis blicki	NT	7.446	2.8268
1599	Rodentia	Sciuridae	Belomys pearsonii	NT	7.425	2.8243
1600	Rodentia	Muridae	Niviventer coxingi	NT	7.422	2.8240
1600	Rodentia	Muridae	Niviventer culturatus	NT	7.422	2.8240
1600	Rodentia	Muridae	Niviventer hinpoon	NT	7.422	2.8240
1603	Carnivora	Herpestidae	Suricata suricatta	LC	15.843	2.8239
1604	Chiroptera	Phyllostomidae	Hylonycteris underwoodi	NT	7.405	2.8220
1605	Artiodactyla	Bovidae	Boselaphus tragocamelus	LC	15.767	2.8194
1606	Dasyuromorphia	Dasyuridae	Dasyurus hallucatus	NT	7.367	2.8175
1607	Rodentia	Muridae	Apodemus semotus	NT	7.363	2.8170
1608	Chiroptera	Vespertilionidae	Myotis sicarius	VU	3.180	2.8166
1609	Rodentia	Muridae	Brachiones przewalskii	LC	15.693	2.8150
1609	Rodentia	Muridae	Sekeetamys calurus	LC	15.693	2.8150

Rank	Order	Family	Species	IUCN	ED'	EDGE
1611	Didelphimorphia	Didelphidae	Philander andersoni	LC	15.678	2.8141
1611	Didelphimorphia	Didelphidae	Philander opossum	LC	15.678	2.8141
1613	Rodentia	Hystricidae	Trichys fasciculata	LC	15.649	2.8124
1614	Lagomorpha	Ochotonidae	Ochotona curzoniae	LC	15.631	2.8113
1614	Lagomorpha	Ochotonidae	Ochotona dauurica	LC	15.631	2.8113
1616	Insectivora	Talpidae	Talpa altaica	LC	15.629	2.8111
1616	Insectivora	Talpidae	Talpa caeca	LC	15.629	2.8111
1616	Insectivora	Talpidae	Talpa caucasica	LC	15.629	2.8111
1616	Insectivora	Talpidae	Talpa europaea	LC	15.629	2.8111
1616	Insectivora	Talpidae	Talpa levantis	LC	15.629	2.8111
1616	Insectivora	Talpidae	Talpa occidentalis	LC	15.629	2.8111
1616	Insectivora	Talpidae	Talpa romana	LC	15.629	2.8111
1616	Insectivora	Talpidae	Talpa stankovici	LC	15.629	2.8111
1624	Diprotodontia	Phalangeridae	Spilocuscus maculatus	LC	15.589	2.8087
1625	Lagomorpha	Ochotonidae	Ochotona nubrica	LC	15.578	2.8081
1626	Dasyuromorphia	Dasyuridae	Ningaui timealeyi	LC	15.556	2.8067
1627	Chiroptera	Nycteridae	Nycteris tragata	LC	15.554	2.8067
1628	Chiroptera	Pteropodidae	Eonycteris major	LC	15.540	2.8058
1628	Chiroptera	Pteropodidae	Eonycteris spelaea	LC	15.540	2.8058
1630	Rodentia	Muridae	Reithrodontomys zacatecae	NT	7.270	2.8058
1631	Rodentia	Echimyidae	Diplomys caniceps	NT	7.260	2.8045
1632	Chiroptera	Pteropodidae	Harpyionycteris whiteheadi	LC	15.511	2.8041
1633	Chiroptera	Pteropodidae	Nyctimene aello	NT	7.253	2.8037
1633	Chiroptera	Pteropodidae	Nyctimene certans	NT	7.253	2.8037
1633	Chiroptera	Pteropodidae	Nyctimene cyclotis	NT	7.253	2.8037
1636	Chiroptera	Rhinolophidae	Rhinolophus keyensis	EN	1.062	2.8033
1636	Chiroptera	Rhinolophidae	Rhinolophus maclaudi	EN	1.062	2.8033
1638	Chiroptera	Vespertilionidae	Kerivoula agnella	VU	3.120	2.8022
1638	Chiroptera	Vespertilionidae	Kerivoula muscina	VU	3.120	2.8022
1638	Chiroptera	Vespertilionidae	Kerivoula myrella	VU	3.120	2.8022
1641	Chiroptera	Pteropodidae	Sphaerias blanfordi	LC	15.477	2.8020
1642	Insectivora	Erinaceidae	Hemiechinus auritus	LC	15.456	2.8007
1642	Insectivora	Erinaceidae	Hemiechinus collaris	LC	15.456	2.8007
1644	Diprotodontia	Phalangeridae	Phalanger pelengensis	LC	15.439	2.7997
1645	Macroscelidea	Macroscelididae	Elephantulus intufi	LC	15.434	2.7993
1645	Macroscelidea	Macroscelididae	Elephantulus rufescens	LC	15.434	2.7993
1645	Macroscelidea	Macroscelididae	Elephantulus rupestris	LC	15.434	2.7993
1648	Xenarthra	Dasypodidae	Euphractus sexcinctus	LC	15.413	2.7980
1649	Artiodactyla	Bovidae	Ovis canadensis	CD	7.199	2.7971
1650	Chiroptera	Vespertilionidae	Myotis aelleni	VU	3.083	2.7930
1650	Chiroptera	Vespertilionidae	Myotis dasycneme	VU	3.083	2.7930
1650	Chiroptera	Vespertilionidae	Myotis peninsularis	VU	3.083	2.7930
1653	Carnivora	Viverridae	Civettictis civetta	LC	15.322	2.7925
1654	Cetacea	Delphinidae	Orcinus orca	CD	7.143	2.7903
1654	Cetacea	Delphinidae	Stenella longirostris	CD	7.143	2.7903
1656	Rodentia	Muridae	Myospalax epsilanus	NT	7.133	2.7891
1656	Rodentia	Muridae	Myospalax rothschildi	NT	7.133	2.7891
1656	Rodentia	Muridae	Myospalax smithii	NT	7.133	2.7891
1659	Rodentia	Muridae	Tatera boehmi	LC	15.259	2.7887
1659	Rodentia	Muridae	Tatera indica	LC	15.259	2.7887
1661	Rodentia	Muridae	Calomyscus mystax	NT	7.101	2.7851
1661	Rodentia	Muridae	Calomyscus tsolovi	NT	7.101	2.7851
1661	Rodentia	Muridae	Calomyscus urartensis	NT	7.101	2.7851
1664	Chiroptera	Molossidae	Tadarida fulminans	LC	15.189	2.7843
1664	Chiroptera	Molossidae	Tadarida teniotis	LC	15.189	2.7843
1666	Rodentia	Muridae	Neofiber alleni	NT	7.087	2.7835

Rank	Order	Family	Species	IUCN	ED'	EDGE
1666	Rodentia	Muridae	Proedromys bedfordi	NT	7.087	2.7835
1668	Chiroptera	Molossidae	Myopterus whitleyi	LC	15.144	2.7815
1669	Dasyuromorphia	Dasyuridae	Planigale maculata	LC	15.113	2.7797
1670	Insectivora	Soricidae	Soriculus nigrescens	LC	15.107	2.7792
1670	Insectivora	Soricidae	Soriculus parca	LC	15.107	2.7792
1672	Rodentia	Muridae	Maxomys hylomyoides	NT	7.048	2.7786
1672	Rodentia	Muridae	Maxomys pagensis	NT	7.048	2.7786
1672	Rodentia	Muridae	Maxomys panglima	NT	7.048	2.7786
1675	Chiroptera	Phyllostomidae	Lonchorhina aurita	LC	15.063	2.7765
1676	Rodentia	Dasyproctidae	Myoprocta acouchy	LC	15.054	2.7759
1677	Rodentia	Sciuridae	Tamias panamintinus	LC	15.028	2.7743
1678	Chiroptera	Phyllostomidae	Artibeus hartii	LC	15.017	2.7736
1679	Carnivora	Phocidae	Cystophora cristata	LC	15.002	2.7727
1680	Didelphimorphia	Didelphidae	Micoureus demerarae	LC	14.999	2.7725
1680	Didelphimorphia	Didelphidae	Micoureus regina	LC	14.999	2.7725
1682	Rodentia	Heteromyidae	Heteromys oresterus	NT	6.987	2.7710
1683	Chiroptera	Pteropodidae	Macroglossus minimus	LC	14.932	2.7683
1683	Chiroptera	Pteropodidae	Macroglossus sobrinus	LC	14.932	2.7683
1685	Chiroptera	Rhinolophidae	Hipposideros coronatus	NT	6.951	2.7664
1686	Rodentia	Sciuridae	Hylopetes baberi	NT	6.932	2.7641
1686	Rodentia	Sciuridae	Hylopetes nigripes	NT	6.932	2.7641
1688	Rodentia	Heteromyidae	Chaetodipus californicus	LC	14.838	2.7624
1689	Rodentia	Sciuridae	Xerus erythropus	LC	14.822	2.7614
1689	Rodentia	Sciuridae	Xerus inauris	LC	14.822	2.7614
1689	Rodentia	Sciuridae	Xerus princeps	LC	14.822	2.7614
1689	Rodentia	Sciuridae	Xerus rutilus	LC	14.822	2.7614
1693	Chiroptera	Vespertilionidae	Pipistrellus paterculus	NT	6.905	2.7606
1694	Chiroptera	Pteropodidae	Melonycteris woodfordi	LC	14.800	2.7600
1695	Rodentia	Sciuridae	Tamiops swinhoei	LC	14.781	2.7588
1696	Artiodactyla	Cervidae	Rangifer tarandus	LC	14.780	2.7587
1697	Rodentia	Muridae	Cricetus cricetus	LC	14.776	2.7585
1698	Rodentia	Heteromyidae	Chaetodipus fallax	LC	14.738	2.7561
1698	Rodentia	Heteromyidae	Chaetodipus spinatus	LC	14.738	2.7561
1700	Insectivora	Soricidae	Neomys anomalus	LC	14.735	2.7559
1700	Insectivora	Soricidae	Neomys fodiens	LC	14.735	2.7559
1700	Insectivora	Soricidae	Neomys schelkovnikovi	LC	14.735	2.7559
1703	Insectivora	Erinaceidae	Hemiechinus aethiopicus	LC	14.730	2.7556
1703	Insectivora	Erinaceidae	Hemiechinus micropus	LC	14.730	2.7556
1705	Carnivora	Herpestidae	Ichneumia albicauda	LC	14.678	2.7523
1706	Lagomorpha	Ochotonidae	Ochotona cansus	LC	14.622	2.7487
1707	Insectivora	Soricidae	Sorex alpinus	LC	14.609	2.7478
1708	Chiroptera	Vespertilionidae	Eptesicus douglasorum	NT	6.798	2.7470
1709	Insectivora	Erinaceidae	Erinaceus concolor	LC	14.595	2.7469
1710	Rodentia	Echimyidae	Olallamys albicauda	NT	6.793	2.7464
1710	Rodentia	Echimyidae	Olallamys edax	NT	6.793	2.7464
1712	Rodentia	Muridae	Oxymycterus hiska	VU	2.894	2.7459
1712	Rodentia	Muridae	Oxymycterus hucucha	VU	2.894	2.7459
1714	Diprotodontia	Pseudocheiridae	Pseudocheirus mayeri	LC	14.575	2.7457
1715	Artiodactyla	Bovidae	Oryx gazella	CD	6.773	2.7438
1716	Carnivora	Mustelidae	Galictis cuja	LC	14.534	2.7430
1716	Carnivora	Mustelidae	Galictis vittata	LC	14.534	2.7430
1718	Chiroptera	Phyllostomidae	Artibeus amplus	NT	6.752	2.7411
1719	Artiodactyla	Bovidae	Ovis nivicola	CD	6.742	2.7398
1720	Xenarthra	Dasypodidae	Chaetophractus vellerosus	LC	14.479	2.7395
1720	Xenarthra	Dasypodidae	Chaetophractus villosus	LC	14.479	2.7395
1722	Carnivora	Viverridae	Hemigalus derbyanus	LC	14.473	2.7391

Rank	Order	Family	Species	IUCN	ED'	EDGE
1723	Rodentia	Sciuridae	Exilisciurus exilis	LC	14.460	2.7383
1723	Rodentia	Sciuridae	Exilisciurus whiteheadi	LC	14.460	2.7383
1725	Lagomorpha	Leporidae	Sylvilagus bachmani	LC	14.458	2.7381
1726	Rodentia	Heteromyidae	Chaetodipus pernix	LC	14.419	2.7356
1727	Diprotodontia	Phalangeridae	Phalanger ornatus	LC	14.416	2.7354
1728	Diprotodontia	Macropodidae	Wallabia bicolor	LC	14.414	2.7352
1729	Carnivora	Viverridae	Poiana richardsonii	LC	14.413	2.7352
1730	Dasyuromorphia	Dasyuridae	Sminthopsis crassicaudata	LC	14.407	2.7348
1731	Rodentia	Muridae	Parotomys brantsii	LC	14.404	2.7346
1731	Rodentia	Muridae	Parotomys littledalei	LC	14.404	2.7346
1733	Chiroptera	Vespertilionidae	Pipistrellus musciculus	NT	6.697	2.7339
1734	Rodentia	Muridae	Acomys minous	VU	2.843	2.7326
1735	Chiroptera	Mormoopidae	Pteronotus davyi	LC	14.361	2.7318
1735	Chiroptera	Mormoopidae	Pteronotus gymnonotus	LC	14.361	2.7318
1737	Chiroptera	Phyllostomidae	Brachyphylla cavernarum	LC	14.323	2.7294
1738	Chiroptera	Phyllostomidae	Rhinophylla pumilio	LC	14.323	2.7294
1739	Chiroptera	Vespertilionidae	Miniopterus fraterculus	LC	14.295	2.7275
1739	Chiroptera	Vespertilionidae	Miniopterus fuscus	LC	14.295	2.7275
1739	Chiroptera	Vespertilionidae	Miniopterus inflatus	LC	14.295	2.7275
1739	Chiroptera	Vespertilionidae	Miniopterus magnater	LC	14.295	2.7275
1739	Chiroptera	Vespertilionidae	Miniopterus tristis	LC	14.295	2.7275
1744	Rodentia	Thryonomyidae	Thryonomys gregorianus	LC	14.275	2.7262
1744	Rodentia	Thryonomyidae	Thryonomys swinderianus	LC	14.275	2.7262
1746	Diprotodontia	Pseudocheiridae	Pseudocheirus forbesi	LC	14.267	2.7257
1747	Chiroptera	Vespertilionidae	Rhogeessa gracilis	NT	6.624	2.7244
1748	Rodentia	Heteromyidae	Microdipodops megacephalus	LC	14.241	2.7240
1748	Rodentia	Heteromyidae	Microdipodops pallidus	LC	14.241	2.7240
1750	Artiodactyla	Cervidae	Ozotoceros bezoarticus	NT	6.611	2.7228
1751	Primates	Cercopithecidae	Colobus polykomos	NT	6.598	2.7211
1752	Primates	Cercopithecidae	Macaca thibetana	CD	6.580	2.7186
1753	Chiroptera	Pteropodidae	Rousettus spinalatus	VU	2.773	2.7141
1754	Primates	Cercopithecidae	Macaca tonkeana	NT	6.534	2.7126
1755	Rodentia	Dasyproctidae	Dasyprocta mexicana	NT	6.529	2.7119
1756	Artiodactyla	Bovidae	Gazella subgutturosa	VU	2.764	2.7118
1757	Chiroptera	Vespertilionidae	Miniopterus australis	LC	14.045	2.7110
1757	Chiroptera	Vespertilionidae	Miniopterus pusillus	LC	14.045	2.7110
1759	Dasyuromorphia	Dasyuridae	Sminthopsis griseoventer	LC	14.037	2.7105
1760	Artiodactyla	Bovidae	Raphicerus sharpei	CD	6.515	2.7100
1761	Chiroptera	Molossidae	Molossops temminckii	LC	14.019	2.7093
1762	Insectivora	Talpidae	Euroscaptor grandis	LC	14.019	2.7093
1762	Insectivora	Talpidae	Euroscaptor klossi	LC	14.019	2.7093
1762	Insectivora	Talpidae	Euroscaptor longirostris	LC	14.019	2.7093
1762	Insectivora	Talpidae	Euroscaptor micrura	LC	14.019	2.7093
1766	Insectivora	Erinaceidae	Atelerix albiventris	LC	14.005	2.7084
1766	Insectivora	Erinaceidae	Atelerix sclateri	LC	14.005	2.7084
1768	Primates	Galagonidae	Otolemur crassicaudatus	LC	13.980	2.7067
1768	Primates	Galagonidae	Otolemur garnettii	LC	13.980	2.7067
1770	Chiroptera	Phyllostomidae	Phyllops falcatus	NT	6.474	2.7045
1771	Rodentia	Sciuridae	Petinomys lugens	NT	6.471	2.7042
1772	Artiodactyla	Bovidae	Tragelaphus scriptus	LC	13.942	2.7041
1773	Carnivora	Phocidae	Phoca sibirica	NT	6.456	2.7022
1774	Diprotodontia	Macropodidae	Macropus agilis	LC	13.855	2.6983
1775	Diprotodontia	Phalangeridae	Phalanger lullulae	LC	13.830	2.6967
1776	Chiroptera	Nycteridae	Nycteris arge	LC	13.826	2.6964
1776	Chiroptera	Nycteridae	Nycteris nana	LC	13.826	2.6964
1778	Chiroptera	Phyllostomidae	Choeroniscus godmani	NT	6.411	2.6962

Rank	Order	Family	Species	IUCN	ED'	EDGE
1778	Chiroptera	Phyllostomidae	Choeroniscus intermedius	NT	6.411	2.6962
1780	Artiodactyla	Bovidae	Oreamnos americanus	LC	13.818	2.6959
1781	Primates	Cercopithecidae	Lophocebus albigena	LC	13.818	2.6958
1782	Chiroptera	Vespertilionidae	Myotis lesueuri	VU	2.702	2.6951
1782	Chiroptera	Vespertilionidae	Myotis seabrai	VU	2.702	2.6951
1784	Cetacea	Delphinidae	Peponocephala electra	LC	13.803	2.6948
1785	Rodentia	Muridae	Platacanthomys lasiurus	LC	13.797	2.6944
1786	Artiodactyla	Bovidae	Cephalophus dorsalis	NT	6.389	2.6932
1787	Rodentia	Sciuridae	Tamias speciosus	LC	13.774	2.6929
1788	Rodentia	Geomyidae	Pappogeomys fumosus	NT	6.383	2.6923
1788	Rodentia	Geomyidae	Pappogeomys tylorhinus	NT	6.383	2.6923
1788	Rodentia	Geomyidae	Pappogeomys zinseri	NT	6.383	2.6923
1791	Lagomorpha	Ochotonidae	Ochotona thibetana	LC	13.753	2.6915
1792	Rodentia	Sciuridae	Petaurista leucogenys	LC	13.737	2.6904
1793	Insectivora	Talpidae	Mogera insularis	LC	13.732	2.6900
1793	Insectivora	Talpidae	Mogera kobeae	LC	13.732	2.6900
1793	Insectivora	Talpidae	Mogera minor	LC	13.732	2.6900
1793	Insectivora	Talpidae	Mogera robusta	LC	13.732	2.6900
1793	Insectivora	Talpidae	Mogera wogura	LC	13.732	2.6900
1798	Dasyuromorphia	Dasyuridae	Sminthopsis hirtipes	LC	13.732	2.6900
1799	Rodentia	Muridae	Nelsonia goldmani	LC	13.701	2.6879
1799	Rodentia	Muridae	Nelsonia neotomodon	LC	13.701	2.6879
1801	Xenarthra	Dasypodidae	Dasypus kappleri	LC	13.700	2.6879
1801	Xenarthra	Dasypodidae	Dasypus novemcinctus	LC	13.700	2.6879
1801	Xenarthra	Dasypodidae	Dasypus sabanicola	LC	13.700	2.6879
1801	Xenarthra	Dasypodidae	Dasypus septemcinctus	LC	13.700	2.6879
1805	Rodentia	Dipodidae	Salpingotus michaelis	LC	13.662	2.6852
1805	Rodentia	Dipodidae	Salpingotus pallidus	LC	13.662	2.6852
1807	Chiroptera	Pteropodidae	Acerodon celebensis	NT	6.329	2.6849
1808	Diprotodontia	Macropodidae	Macropus parryi	LC	13.652	2.6846
1808	Diprotodontia	Macropodidae	Macropus rufogriseus	LC	13.652	2.6846
1810	Insectivora	Soricidae	Chimarrogale himalayica	LC	13.637	2.6836
1810	Insectivora	Soricidae	Chimarrogale platycephala	LC	13.637	2.6836
1810	Insectivora	Soricidae	Chimarrogale styani	LC	13.637	2.6836
1813	Chiroptera	Phyllostomidae	Pygoderma bilabiatum	NT	6.294	2.6801
1814	Rodentia	Muridae	Nannospalax ehrenbergi	LC	13.585	2.6800
1814	Rodentia	Muridae	Nannospalax nehringi	LC	13.585	2.6800
1816	Carnivora	Mustelidae	Lutra lutra	NT	6.283	2.6786
1817	Diprotodontia	Phalangeridae	Trichosurus caninus	LC	13.538	2.6767
1818	Artiodactyla	Bovidae	Redunca fulvorufula	LC	13.516	2.6752
1819	Rodentia	Muridae	Neotoma phenax	LC	13.515	2.6752
1820	Chiroptera	Phyllostomidae	Lionycteris spurrelli	LC	13.505	2.6745
1821	Rodentia	Sciuridae	Tamias amoenus	LC	13.485	2.6731
1822	Peramelemorphia	Peramelidae	Perameles nasuta	LC	13.425	2.6690
1823	Rodentia	Sciuridae	Tamias quadrimaculatus	LC	13.419	2.6685
1824	Chiroptera	Vespertilionidae	Myotis bombinus	NT	6.201	2.6674
1825	Rodentia	Geomyidae	Thomomys bulbivorus	LC	13.392	2.6667
1826	Rodentia	Muridae	Prometheomys schaposchnikowi	LC	13.392	2.6667
1827	Insectivora	Erinaceidae	Erinaceus amurensis	LC	13.383	2.6661
1827	Insectivora	Erinaceidae	Erinaceus europaeus	LC	13.383	2.6661
1829	Chiroptera	Phyllostomidae	Erophylla sezekorni	LC	13.374	2.6654
1830	Insectivora	Soricidae	Soriculus caudatus	LC	13.373	2.6654
1830	Insectivora	Soricidae	Soriculus fumidus	LC	13.373	2.6654
1830	Insectivora	Soricidae	Soriculus hypsibius	LC	13.373	2.6654
1830	Insectivora	Soricidae	Soriculus lamula	LC	13.373	2.6654
1830	Insectivora	Soricidae	Soriculus leucops	LC	13.373	2.6654

Rank	Order	Family	Species	IUCN	ED'	EDGE
1830	Insectivora	Soricidae	Soriculus macrurus	LC	13.373	2.6654
1830	Insectivora	Soricidae	Soriculus smithii	LC	13.373	2.6654
1837	Primates	Cebidae	Alouatta fusca	NT	6.186	2.6652
1838	Dasyuromorphia	Dasyuridae	Ningaui ridei	LC	13.368	2.6650
1838	Dasyuromorphia	Dasyuridae	Ningaui yvonnae	LC	13.368	2.6650
1840	Rodentia	Muridae	Desmodillus auricularis	LC	13.347	2.6636
1841	Carnivora	Herpestidae	Cynictis penicillata	LC	13.345	2.6634
1841	Carnivora	Herpestidae	Paracynictis selousi	LC	13.345	2.6634
1843	Rodentia	Heteromyidae	Chaetodipus baileyi	LC	13.308	2.6608
1843	Rodentia	Heteromyidae	Chaetodipus hispidus	LC	13.308	2.6608
1845	Chiroptera	Molossidae	Molossops greenhalli	LC	13.301	2.6603
1845	Chiroptera	Molossidae	Molossops planirostris	LC	13.301	2.6603
1847	Carnivora	Mustelidae	Amblonyx cinereus	NT	6.147	2.6598
1848	Rodentia	Sciuridae	Prosciurillus leucomus	LC	13.259	2.6574
1848	Rodentia	Sciuridae	Prosciurillus murinus	LC	13.259	2.6574
1850	Primates	Hylobatidae	Hylobates agilis	NT	6.104	2.6538
1850	Primates	Hylobatidae	Hylobates lar	NT	6.104	2.6538
1850	Primates	Hylobatidae	Hylobates muelleri	NT	6.104	2.6538
1853	Scandentia	Tupaiidae	Tupaia belangeri	LC	13.202	2.6534
1853	Scandentia	Tupaiidae	Tupaia dorsalis	LC	13.202	2.6534
1853	Scandentia	Tupaiidae	Tupaia glis	LC	13.202	2.6534
1853	Scandentia	Tupaiidae	Tupaia gracilis	LC	13.202	2.6534
1853	Scandentia	Tupaiidae	Tupaia javanica	LC	13.202	2.6534
1853	Scandentia	Tupaiidae	Tupaia minor	LC	13.202	2.6534
1853	Scandentia	Tupaiidae	Tupaia montana	LC	13.202	2.6534
1853	Scandentia	Tupaiidae	Tupaia picta	LC	13.202	2.6534
1853	Scandentia	Tupaiidae	Tupaia splendidula	LC	13.202	2.6534
1853	Scandentia	Tupaiidae	Tupaia tana	LC	13.202	2.6534
1863	Carnivora	Ursidae	Ursus arctos	LC	13.199	2.6532
1864	Rodentia	Heteromyidae	Heteromys goldmani	NT	6.090	2.6519
1865	Rodentia	Muridae	Isthmomys flavidus	LC	13.167	2.6509
1866	Chiroptera	Phyllostomidae	Micronycteris hirsuta	LC	13.162	2.6506
1866	Chiroptera	Phyllostomidae	Micronycteris megalotis	LC	13.162	2.6506
1868	Carnivora	Viverridae	Viverricula indica	LC	13.150	2.6497
1869	Dasyuromorphia	Dasyuridae	Planigale tenuirostris	LC	13.127	2.6481
1870	Rodentia	Sciuridae	Pteromys momonga	LC	13.124	2.6479
1871	Chiroptera	Pteropodidae	Pteropus personatus	LC	13.120	2.6476
1872	Rodentia	Muridae	Rattus foramineus	NT	6.060	2.6476
1873	Rodentia	Muridae	Desmodilliscus braueri	LC	13.063	2.6435
1873	Rodentia	Muridae	Pachyuromys duprasi	LC	13.063	2.6435
1875	Carnivora	Mustelidae	Mustela vison	LC	13.061	2.6434
1876	Lagomorpha	Leporidae	Lepus insularis	NT	6.026	2.6428
1877	Carnivora	Herpestidae	Galerella flavescens	LC	13.044	2.6422
1877	Carnivora	Herpestidae	Galerella pulverulenta	LC	13.044	2.6422
1877	Carnivora	Herpestidae	Galerella sanguinea	LC	13.044	2.6422
1877	Carnivora	Herpestidae	Galerella swalius	LC	13.044	2.6422
1881	Chiroptera	Pteropodidae	Syconycteris australis	LC	13.034	2.6415
1882	Rodentia	Sciuridae	Syntheosciurus brochus	NT	5.993	2.6381
1883	Rodentia	Sciuridae	Funambulus pennantii	LC	12.972	2.6370
1884	Rodentia	Dipodidae	Allactaga balikunica	LC	12.964	2.6365
1884	Rodentia	Dipodidae	Allactaga elater	LC	12.964	2.6365
1884	Rodentia	Dipodidae	Allactaga hotsoni	LC	12.964	2.6365
1884	Rodentia	Dipodidae	Allactaga major	LC	12.964	2.6365
1884	Rodentia	Dipodidae	Allactaga severtzovi	LC	12.964	2.6365
1884	Rodentia	Dipodidae	Allactaga sibirica	LC	12.964	2.6365
1884	Rodentia	Dipodidae	Allactaga vinogradovi	LC	12.964	2.6365

Rank	Order	Family	Species	IUCN	ED'	EDGE
1891	Rodentia	Geomyidae	Geomys pinetis	LC	12.956	2.6359
1892	Carnivora	Viverridae	Paradoxurus hermaphroditus	LC	12.943	2.6350
1893	Lagomorpha	Ochotonidae	Ochotona pallasi	LC	12.923	2.6335
1894	Carnivora	Mustelidae	Martes pennanti	LC	12.906	2.6323
1895	Diprotodontia	Macropodidae	Petrogale burbidgei	NT	5.950	2.6319
1895	Diprotodontia	Macropodidae	Petrogale concinna	NT	5.950	2.6319
1897	Carnivora	Phocidae	Hydrurga leptonyx	LC	12.891	2.6312
1897	Carnivora	Phocidae	Lobodon carcinophagus	LC	12.891	2.6312
1899	Primates	Cercopithecidae	Papio hamadryas	LC	12.881	2.6305
1900	Rodentia	Sciuridae	Petaurista xanthotis	LC	12.858	2.6288
1901	Rodentia	Muridae	Peromyscus bullatus	EN	0.732	2.6288
1901	Rodentia	Muridae	Peromyscus dickeyi	EN	0.732	2.6288
1901	Rodentia	Muridae	Peromyscus interparietalis	EN	0.732	2.6288
1901	Rodentia	Muridae	Peromyscus mayensis	EN	0.732	2.6288
1901	Rodentia	Muridae	Peromyscus stephani	EN	0.732	2.6288
1906	Chiroptera	Vespertilionidae	Antrozous pallidus	LC	12.823	2.6263
1907	Chiroptera	Nycteridae	Nycteris gambiensis	LC	12.799	2.6246
1907	Chiroptera	Nycteridae	Nycteris grandis	LC	12.799	2.6246
1907	Chiroptera	Nycteridae	Nycteris hispida	LC	12.799	2.6246
1907	Chiroptera	Nycteridae	Nycteris macrotis	LC	12.799	2.6246
1907	Chiroptera	Nycteridae	Nycteris thebaica	LC	12.799	2.6246
1912	Dasyuromorphia	Dasyuridae	Sminthopsis ooldea	LC	12.783	2.6234
1912	Dasyuromorphia	Dasyuridae	Sminthopsis youngsoni	LC	12.783	2.6234
1914	Rodentia	Muridae	Deomys ferrugineus	LC	12.768	2.6224
1914	Rodentia	Muridae	Lophiomys imhausi	LC	12.768	2.6224
1914	Rodentia	Muridae	Malacothrix typica	LC	12.768	2.6224
1917	Chiroptera	Molossidae	Chaerephon ansorgei	LC	12.765	2.6222
1917	Chiroptera	Molossidae	Chaerephon bemmeleni	LC	12.765	2.6222
1917	Chiroptera	Molossidae	Chaerephon bivittata	LC	12.765	2.6222
1920	Carnivora	Phocidae	Leptonychotes weddellii	LC	12.761	2.6219
1920	Carnivora	Phocidae	Ommatophoca rossii	LC	12.761	2.6219
1922	Rodentia	Muridae	Allocricetulus curtatus	LC	12.761	2.6218
1922	Rodentia	Muridae	Allocricetulus eversmanni	LC	12.761	2.6218
1924	Rodentia	Heteromyidae	Liomys adspersus	NT	5.879	2.6217
1925	Carnivora	Procyonidae	Bassariscus astutus	LC	12.732	2.6197
1926	Rodentia	Muridae	Acomys louisae	LC	12.726	2.6193
1927	Rodentia	Agoutidae	Agouti paca	LC	12.721	2.6189
1928	Rodentia	Muridae	Scapteromys tumidus	LC	12.716	2.6185
1929	Lagomorpha	Leporidae	Lepus callotis	NT	5.845	2.6167
1930	Primates	Lemuridae	Eulemur fulvus	LC	12.682	2.6161
1931	Chiroptera	Phyllostomidae	Mimon bennettii	LC	12.670	2.6152
1931	Chiroptera	Phyllostomidae	Mimon crenulatum	LC	12.670	2.6152
1933	Rodentia	Sciuridae	Protoxerus aubinnii	LC	12.643	2.6132
1933	Rodentia	Sciuridae	Protoxerus stangeri	LC	12.643	2.6132
1935	Chiroptera	Phyllostomidae	Monophyllus redmani	LC	12.606	2.6105
1936	Didelphimorphia	Didelphidae	Marmosops noctivagus	LC	12.585	2.6090
1937	Chiroptera	Phyllostomidae	Mesophylla macconnelli	LC	12.563	2.6073
1938	Peramelemorphia	Peramelidae	Isoodon macrourus	LC	12.561	2.6072
1939	Carnivora	Mustelidae	Mydaus javanensis	LC	12.522	2.6043
1940	Rodentia	Chinchillidae	Lagidium peruanum	LC	12.499	2.6026
1941	Artiodactyla	Cervidae	Pudu mephistophiles	NT	5.746	2.6022
1942	Rodentia	Dipodidae	Napaeozapus insignis	LC	12.477	2.6010
1943	Artiodactyla	Bovidae	Cephalophus monticola	LC	12.461	2.5998
1944	Chiroptera	Phyllostomidae	Choeronycteris mexicana	NT	5.707	2.5962

Rank	Order	Family	Species	IUCN	ED'	EDGE
1945	Didelphimorphia	Didelphidae	Monodelphis adusta	LC	12.337	2.5905
1945	Didelphimorphia	Didelphidae	Monodelphis brevicaudata	LC	12.337	2.5905
1945	Didelphimorphia	Didelphidae	Monodelphis domestica	LC	12.337	2.5905
1948	Rodentia	Geomyidae	Thomomys umbrinus	LC	12.336	2.5905
1949	Rodentia	Muridae	Mesocricetus brandti	LC	12.281	2.5864
1949	Rodentia	Muridae	Mesocricetus raddei	LC	12.281	2.5864
1951	Dasyuromorphia	Dasyuridae	Dasyurus viverrinus	NT	5.640	2.5863
1952	Rodentia	Muridae	Cannomys badius	LC	12.273	2.5857
1953	Rodentia	Muridae	Neotomodon alstoni	LC	12.266	2.5852
1954	Rodentia	Muridae	Otomys denti	NT	5.625	2.5840
1955	Carnivora	Felidae	Prionailurus bengalensis	LC	12.222	2.5819
1956	Rodentia	Heteromyidae	Liomys spectabilis	NT	5.598	2.5799
1957	Insectivora	Soricidae	Cryptotis goodwini	LC	12.191	2.5795
1957	Insectivora	Soricidae	Cryptotis magna	LC	12.191	2.5795
1957	Insectivora	Soricidae	Cryptotis meridensis	LC	12.191	2.5795
1957	Insectivora	Soricidae	Cryptotis nigrescens	LC	12.191	2.5795
1957	Insectivora	Soricidae	Cryptotis parva	LC	12.191	2.5795
1957	Insectivora	Soricidae	Cryptotis thomasi	LC	12.191	2.5795
1963	Insectivora	Soricidae	Paracrocidura maxima	NT	5.593	2.5792
1964	Dasyuromorphia	Dasyuridae	Sminthopsis macroura	LC	12.174	2.5782
1965	Diprotodontia	Macropodidae	Petrogale assimilis	LC	12.164	2.5775
1965	Diprotodontia	Macropodidae	Petrogale godmani	LC	12.164	2.5775
1967	Chiroptera	Phyllostomidae	Sturnira magna	NT	5.570	2.5756
1967	Chiroptera	Phyllostomidae	Sturnira mordax	NT	5.570	2.5756
1969	Rodentia	Sciuridae	Petaurista elegans	LC	12.138	2.5755
1970	Carnivora	Mustelidae	Vormela peregusna	LC	12.038	2.5679
1971	Rodentia	Muridae	Apodemus mystacinus	LC	12.027	2.5670
1972	Chiroptera	Molossidae	Mops brachypterus	LC	11.998	2.5648
1972	Chiroptera	Molossidae	Mops nanulus	LC	11.998	2.5648
1972	Chiroptera	Molossidae	Mops spurrelli	LC	11.998	2.5648
1972	Chiroptera	Molossidae	Mops thersites	LC	11.998	2.5648
1976	Rodentia	Muridae	Nesoryzomys fernandinae	VU	2.249	2.5645
1976	Rodentia	Muridae	Nesoryzomys swarthi	VU	2.249	2.5645
1978	Chiroptera	Phyllostomidae	Phyllostomus discolor	LC	11.974	2.5629
1979	Carnivora	Procyonidae	Bassaricyon alleni	LC	11.965	2.5622
1980	Rodentia	Muridae	Psammomys obesus	LC	11.961	2.5619
1981	Rodentia	Sciuridae	Tamias ochrogenys	LC	11.959	2.5618
1981	Rodentia	Sciuridae	Tamias ruficaudus	LC	11.959	2.5618
1983	Rodentia	Muridae	Cricetulus alticola	LC	11.955	2.5615
1983	Rodentia	Muridae	Cricetulus barabensis	LC	11.955	2.5615
1983	Rodentia	Muridae	Cricetulus kamensis	LC	11.955	2.5615
1983	Rodentia	Muridae	Cricetulus longicaudatus	LC	11.955	2.5615
1983	Rodentia	Muridae	Cricetulus sokolovi	LC	11.955	2.5615
1988	Chiroptera	Pteropodidae	Balionycteris maculata	LC	11.949	2.5610
1988	Chiroptera	Pteropodidae	Chironax melanocephalus	LC	11.949	2.5610
1990	Rodentia	Muridae	Pseudomys shortridgei	CD	5.469	2.5601
1990	Rodentia	Muridae	Pseudomys delicatulus	NT	5.469	2.5601
1990	Rodentia	Muridae	Pseudomys desertor	NT	5.469	2.5601
1990	Rodentia	Muridae	Pseudomys johnsoni	NT	5.469	2.5601
1990	Rodentia	Muridae	Pseudomys nanus	NT	5.469	2.5601
1995	Diprotodontia	Phalangeridae	Trichosurus vulpecula	LC	11.937	2.5601
1996	Rodentia	Muridae	Microtus breweri	NT	5.447	2.5567
1996	Rodentia	Muridae	Microtus townsendii	NT	5.447	2.5567
1998	Carnivora	Felidae	Leptailurus serval	LC	11.892	2.5566
1999	Rodentia	Heteromyidae	Heteromys gaumeri	LC	11.867	2.5546

Rank	Order	Family	Species	IUCN	ED'	EDGE
2000	Carnivora	Mustelidae	Arctonyx collaris	LC	11.865	2.5545
2000	Carnivora	Mustelidae	Meles meles	LC	11.865	2.5545
2002	Artiodactyla	Bovidae	Cephalophus silvicultor	NT	5.432	2.5545
2003	Chiroptera	Vespertilionidae	Nycticeius schlieffeni	LC	11.858	2.5539
2003	Chiroptera	Vespertilionidae	Pipistrellus tasmaniensis	LC	11.858	2.5539
2005	Chiroptera	Vespertilionidae	Plecotus auritus	LC	11.846	2.5530
2005	Chiroptera	Vespertilionidae	Plecotus austriacus	LC	11.846	2.5530
2007	Rodentia	Muridae	Onychomys arenicola	LC	11.823	2.5512
2007	Rodentia	Muridae	Onychomys leucogaster	LC	11.823	2.5512
2007	Rodentia	Muridae	Onychomys torridus	LC	11.823	2.5512
2010	Rodentia	Ctenomyidae	Ctenomys magellanicus	VU	2.199	2.5491
2011	Rodentia	Muridae	Spalax zemni	LC	11.776	2.5476
2012	Rodentia	Dipodidae	Sicista caucasica	LC	11.769	2.5470
2012	Rodentia	Dipodidae	Sicista concolor	LC	11.769	2.5470
2012	Rodentia	Dipodidae	Sicista napaea	LC	11.769	2.5470
2012	Rodentia	Dipodidae	Sicista severtzovi	LC	11.769	2.5470
2012	Rodentia	Dipodidae	Sicista strandi	LC	11.769	2.5470
2012	Rodentia	Dipodidae	Sicista tianshanica	LC	11.769	2.5470
2018	Rodentia	Muridae	Typhlomys cinereus	LC	11.764	2.5466
2019	Rodentia	Bathyergidae	Bathyergus janetta	LC	11.761	2.5464
2019	Rodentia	Bathyergidae	Bathyergus suillus	LC	11.761	2.5464
2021	Cetacea	Delphinidae	Pseudorca crassidens	LC	11.754	2.5458
2022	Carnivora	Otariidae	Arctocephalus pusillus	LC	11.753	2.5458
2023	Rodentia	Muridae	Echiothrix leucura	LC	11.726	2.5437
2024	Rodentia	Sciuridae	Spermophilus madrensis	NT	5.343	2.5405
2025	Rodentia	Geomyidae	Thomomys bottae	LC	11.685	2.5405
2025	Rodentia	Geomyidae	Thomomys townsendii	LC	11.685	2.5405
2027	Rodentia	Muridae	Scotinomys teguina	LC	11.658	2.5383
2027	Rodentia	Muridae	Scotinomys xerampelinus	LC	11.658	2.5383
2029	Rodentia	Muridae	Nesokia bunnii	NT	5.317	2.5364
2029	Rodentia	Muridae	Phloeomys pallidus	NT	5.317	2.5364
2029	Rodentia	Muridae	Pithecheir melanurus	NT	5.317	2.5364
2032	Rodentia	Sciuridae	Tamias durangae	LC	11.589	2.5328
2033	Carnivora	Phocidae	Mirounga angustirostris	LC	11.577	2.5319
2033	Carnivora	Phocidae	Mirounga leonina	LC	11.577	2.5319
2035	Artiodactyla	Bovidae	Cephalophus leucogaster	NT	5.286	2.5315
2035	Artiodactyla	Bovidae	Cephalophus niger	NT	5.286	2.5315
2037	Carnivora	Viverridae	Arctictis binturong	LC	11.568	2.5312
2037	Carnivora	Viverridae	Paguma larvata	LC	11.568	2.5312
2039	Chiroptera	Vespertilionidae	Myotis elegans	NT	5.264	2.5280
2039	Chiroptera	Vespertilionidae	Myotis martiniquensis	NT	5.264	2.5280
2039	Chiroptera	Vespertilionidae	Myotis nesopolus	NT	5.264	2.5280
2042	Chiroptera	Vespertilionidae	Pipistrellus nathusii	LC	11.522	2.5275
2042	Chiroptera	Vespertilionidae	Pipistrellus pipistrellus	LC	11.522	2.5275
2044	Rodentia	Sciuridae	Ratufa affinis	LC	11.521	2.5274
2044	Rodentia	Sciuridae	Ratufa bicolor	LC	11.521	2.5274
2046	Rodentia	Muridae	Apomys sacobianus	VU	2.130	2.5274
2047	Rodentia	Echimyidae	Makalata armata	LC	11.516	2.5270
2048	Diprotodontia	Phalangeridae	Phalanger sericeus	LC	11.505	2.5262
2049	Chiroptera	Molossidae	Mops condylurus	LC	11.496	2.5254
2049	Chiroptera	Molossidae	Mops midas	LC	11.496	2.5254
2049	Chiroptera	Molossidae	Mops mops	LC	11.496	2.5254
2052	Rodentia	Muridae	Rattus hainaldi	NT	5.242	2.5244
2052	Rodentia	Muridae	Rattus sordidus	NT	5.242	2.5244
2052	Rodentia	Muridae	Rattus tunneyi	NT	5.242	2.5244
2055	Rodentia	Sciuridae	Sciurus vulgaris	NT	5.235	2.5233

Rank	Order	Family	Species	IUCN	ED'	EDGE
2056	Rodentia	Muridae	Petromyscus barbouri	LC	11.460	2.5225
2056	Rodentia	Muridae	Petromyscus collinus	LC	11.460	2.5225
2056	Rodentia	Muridae	Petromyscus monticularis	LC	11.460	2.5225
2056	Rodentia	Muridae	Petromyscus shortridgei	LC	11.460	2.5225
2060	Rodentia	Muridae	Microtus felteni	NT	5.228	2.5223
2061	Primates	Cebidae	Lagothrix lagotricha	LC	11.452	2.5219
2062	Carnivora	Herpestidae	Herpestes ichneumon	LC	11.432	2.5203
2062	Carnivora	Herpestidae	Herpestes naso	LC	11.432	2.5203
2062	Carnivora	Herpestidae	Herpestes semitorquatus	LC	11.432	2.5203
2062	Carnivora	Herpestidae	Herpestes smithii	LC	11.432	2.5203
2062	Carnivora	Herpestidae	Herpestes vitticollis	LC	11.432	2.5203
2067	Chiroptera	Phyllostomidae	Ardops nichollsi	NT	5.213	2.5197
2068	Rodentia	Heteromyidae	Dipodomys nitratoides	NT	5.210	2.5193
2069	Rodentia	Muridae	Neacomys pictus	NT	5.198	2.5174
2070	Rodentia	Sciuridae	Lariscus insignis	LC	11.393	2.5172
2071	Rodentia	Muridae	Steatomys caurinus	LC	11.388	2.5167
2071	Rodentia	Muridae	Steatomys cuppedius	LC	11.388	2.5167
2071	Rodentia	Muridae	Steatomys krebsii	LC	11.388	2.5167
2071	Rodentia	Muridae	Steatomys parvus	LC	11.388	2.5167
2071	Rodentia	Muridae	Steatomys pratensis	LC	11.388	2.5167
2076	Rodentia	Sciuridae	Tamiops macclellandi	LC	11.364	2.5148
2076	Rodentia	Sciuridae	Tamiops maritimus	LC	11.364	2.5148
2076	Rodentia	Sciuridae	Tamiops rodolphei	LC	11.364	2.5148
2079	Chiroptera	Phyllostomidae	Platyrrhinus aurarius	NT	5.181	2.5146
2079	Chiroptera	Phyllostomidae	Platyrrhinus infuscus	NT	5.181	2.5146
2079	Chiroptera	Phyllostomidae	Platyrrhinus umbratus	NT	5.181	2.5146
2082	Insectivora	Soricidae	Cryptotis avia	LC	11.355	2.5140
2082	Insectivora	Soricidae	Cryptotis goldmani	LC	11.355	2.5140
2082	Insectivora	Soricidae	Cryptotis mexicana	LC	11.355	2.5140
2082	Insectivora	Soricidae	Cryptotis montivaga	LC	11.355	2.5140
2082	Insectivora	Soricidae	Cryptotis squamipes	LC	11.355	2.5140
2087	Lagomorpha	Ochotonidae	Ochotona collaris	LC	11.319	2.5111
2087	Lagomorpha	Ochotonidae	Ochotona princeps	LC	11.319	2.5111
2089	Primates	Cebidae	Ateles paniscus	LC	11.293	2.5090
2090	Carnivora	Viverridae	Genetta servalina	LC	11.273	2.5074
2090	Carnivora	Viverridae	Genetta victoriae	LC	11.273	2.5074
2092	Rodentia	Sciuridae	Spermophilus major	NT	5.136	2.5074
2093	Chiroptera	Vespertilionidae	Harpiocephalus harpia	LC	11.269	2.5071
2094	Artiodactyla	Bovidae	Capra pyrenaica	NT	5.129	2.5062
2095	Carnivora	Herpestidae	Helogale hirtula	LC	11.249	2.5054
2095	Carnivora	Herpestidae	Helogale parvula	LC	11.249	2.5054
2097	Rodentia	Sciuridae	Sciurus anomalus	NT	5.110	2.5030
2098	Rodentia	Muridae	Hyperacrius fertilis	LC	11.209	2.5022
2098	Rodentia	Muridae	Hyperacrius wynnei	LC	11.209	2.5022
2100	Artiodactyla	Bovidae	Rupicapra rupicapra	LC	11.186	2.5003
2101	Rodentia	Muridae	Meriones hurrianae	LC	11.180	2.4998
2101	Rodentia	Muridae	Meriones tamariscinus	LC	11.180	2.4998
2103	Diprotodontia	Phalangeridae	Phalanger orientalis	LC	11.138	2.4963
2104	Rodentia	Sciuridae	Dremomys everetti	LC	11.121	2.4949
2104	Rodentia	Sciuridae	Dremomys lokriah	LC	11.121	2.4949
2104	Rodentia	Sciuridae	Dremomys pernyi	LC	11.121	2.4949
2104	Rodentia	Sciuridae	Dremomys pyrrhomerus	LC	11.121	2.4949
2104	Rodentia	Sciuridae	Dremomys rufigenis	LC	11.121	2.4949
2109	Carnivora	Viverridae	Paradoxurus zeylonensis	LC	11.115	2.4944
2110	Rodentia	Echimyidae	Echimys blainvillei	NT	5.053	2.4937

Rank	Order	Family	Species	IUCN	ED'	EDGE
2111	Lagomorpha	Ochotonidae	Ochotona gloveri	LC	11.101	2.4933
2111	Lagomorpha	Ochotonidae	Ochotona ladacensis	LC	11.101	2.4933
2111	Lagomorpha	Ochotonidae	Ochotona rutila	LC	11.101	2.4933
2114	Rodentia	Dipodidae	Zapus hudsonius	LC	11.098	2.4930
2114	Rodentia	Dipodidae	Zapus princeps	LC	11.098	2.4930
2116	Carnivora	Felidae	Caracal caracal	LC	11.096	2.4929
2117	Rodentia	Geomyidae	Thomomys monticola	LC	11.090	2.4924
2117	Rodentia	Geomyidae	Thomomys talpoides	LC	11.090	2.4924
2119	Rodentia	Muridae	Anisomys imitator	LC	11.078	2.4913
2119	Rodentia	Muridae	Crossomys moncktoni	LC	11.078	2.4913
2119	Rodentia	Muridae	Parahydromys asper	LC	11.078	2.4913
2122	Rodentia	Sciuridae	Tamias siskiyou	LC	11.056	2.4895
2123	Insectivora	Tenrecidae	Microgale brevicaudata	LC	11.052	2.4892
2123	Insectivora	Tenrecidae	Microgale cowani	LC	11.052	2.4892
2123	Insectivora	Tenrecidae	Microgale dobsoni	LC	11.052	2.4892
2123	Insectivora	Tenrecidae	Microgale gracilis	LC	11.052	2.4892
2123	Insectivora	Tenrecidae	Microgale longicaudata	LC	11.052	2.4892
2123	Insectivora	Tenrecidae	Microgale parvula	LC	11.052	2.4892
2123	Insectivora	Tenrecidae	Microgale principula	LC	11.052	2.4892
2123	Insectivora	Tenrecidae	Microgale pusilla	LC	11.052	2.4892
2123	Insectivora	Tenrecidae	Microgale talazaci	LC	11.052	2.4892
2123	Insectivora	Tenrecidae	Microgale thomasi	LC	11.052	2.4892
2133	Rodentia	Muridae	Lorentzimys nouhuysi	LC	11.051	2.4892
2134	Lagomorpha	Ochotonidae	Ochotona macrotis	LC	11.050	2.4890
2134	Lagomorpha	Ochotonidae	Ochotona roylei	LC	11.050	2.4890
2136	Rodentia	Heteromyidae	Dipodomys phillipsii	NT	4.986	2.4825
2137	Artiodactyla	Bovidae	Cephalophus callipygus	NT	4.983	2.4820
2137	Artiodactyla	Bovidae	Cephalophus ogilbyi	NT	4.983	2.4820
2137	Artiodactyla	Bovidae	Cephalophus weynsi	NT	4.983	2.4820
2140	Chiroptera	Pteropodidae	Pteropus samoensis	VU	1.990	2.4817
2141	Lagomorpha	Leporidae	Lepus comus	LC	10.941	2.4800
2141	Lagomorpha	Leporidae	Lepus oiostolus	LC	10.941	2.4800
2143	Rodentia	Muridae	Chrotomys whiteheadi	VU	1.974	2.4761
2144	Chiroptera	Phyllostomidae	Phyllostomus elongatus	LC	10.894	2.4760
2144	Chiroptera	Phyllostomidae	Phyllostomus hastatus	LC	10.894	2.4760
2146	Carnivora	Herpestidae	Herpestes brachyurus	LC	10.891	2.4758
2146	Carnivora	Herpestidae	Herpestes urva	LC	10.891	2.4758
2148	Peramelemorphia	Peroryctidae	Microperoryctes longicauda	LC	10.887	2.4754
2149	Lagomorpha	Leporidae	Lepus americanus	LC	10.873	2.4742
2150	Chiroptera	Vespertilionidae	Nyctophilus walkeri	NT	4.925	2.4724
2151	Peramelemorphia	Peroryctidae	Peroryctes raffrayana	LC	10.850	2.4723
2152	Rodentia	Geomyidae	Orthogeomys cherriei	NT	4.917	2.4709
2152	Rodentia	Geomyidae	Orthogeomys heterodus	NT	4.917	2.4709
2154	Carnivora	Otariidae	Zalophus californianus	LC	10.793	2.4675
2155	Carnivora	Procyonidae	Nasua narica	LC	10.786	2.4669
2155	Carnivora	Procyonidae	Nasua nasua	LC	10.786	2.4669
2157	Lagomorpha	Ochotonidae	Ochotona alpina	LC	10.779	2.4663
2157	Lagomorpha	Ochotonidae	Ochotona hyperborea	LC	10.779	2.4663
2159	Rodentia	Sciuridae	Iomys horsfieldii	LC	10.760	2.4647
2160	Primates	Cebidae	Callicebus torquatus	LC	10.755	2.4643
2161	Rodentia	Capromyidae	Capromys pilorides	LC	10.754	2.4642
2162	Chiroptera	Molossidae	Mops niveiventer	LC	10.742	2.4632
2163	Primates	Cercopithecidae	Cercopithecus mitis	LC	10.727	2.4619
2163	Primates	Cercopithecidae	Cercopithecus nictitans	LC	10.727	2.4619
2165	Chiroptera	Pteropodidae	Pteropus vetulus	NT	4.863	2.4619
2166	Primates	Cercopithecidae	Presbytis melalophos	NT	4.859	2.4612

Rank	Order	Family	Species	IUCN	ED'	EDGE
2167	Rodentia	Muridae	Melomys aerosus	NT	4.846	2.4589
2167	Rodentia	Muridae	Melomys bougainville	NT	4.846	2.4589
2167	Rodentia	Muridae	Melomys fraterculus	NT	4.846	2.4589
2167	Rodentia	Muridae	Melomys obiensis	NT	4.846	2.4589
2171	Rodentia	Dipodidae	Dipus sagitta	LC	10.679	2.4578
2171	Rodentia	Dipodidae	Eremodipus lichtensteini	LC	10.679	2.4578
2173	Rodentia	Muridae	Dendromus insignis	LC	10.676	2.4575
2173	Rodentia	Muridae	Dendromus kivu	LC	10.676	2.4575
2173	Rodentia	Muridae	Dendromus melanotis	LC	10.676	2.4575
2173	Rodentia	Muridae	Dendromus mesomelas	LC	10.676	2.4575
2173	Rodentia	Muridae	Dendromus messorius	LC	10.676	2.4575
2173	Rodentia	Muridae	Dendromus mystacalis	LC	10.676	2.4575
2173	Rodentia	Muridae	Dendromus nyikae	LC	10.676	2.4575
2180	Dasyuromorphia	Dasyuridae	Antechinus minimus	LC	10.674	2.4574
2180	Dasyuromorphia	Dasyuridae	Antechinus swainsonii	LC	10.674	2.4574
2182	Carnivora	Mustelidae	Melogale moschata	LC	10.668	2.4569
2182	Carnivora	Mustelidae	Melogale personata	LC	10.668	2.4569
2184	Carnivora	Mustelidae	Mustela kathiah	LC	10.658	2.4560
2185	Rodentia	Echimyidae	Chaetomys subspinosus	VU	1.912	2.4553
2186	Peramelemorphia	Peroryctidae	Echymipera kalubu	LC	10.645	2.4549
2186	Peramelemorphia	Peroryctidae	Echymipera rufescens	LC	10.645	2.4549
2188	Carnivora	Mustelidae	Martes foina	LC	10.631	2.4537
2189	Rodentia	Muridae	Reithrodontomys fulvescens	LC	10.621	2.4528
2190	Rodentia	Sciuridae	Glaucomys sabrinus	LC	10.613	2.4522
2190	Rodentia	Sciuridae	Glaucomys volans	LC	10.613	2.4522
2192	Chiroptera	Rhinolophidae	Rhinolophus euryale	VU	1.901	2.4513
2192	Chiroptera	Rhinolophidae	Rhinolophus guineensis	VU	1.901	2.4513
2192	Chiroptera	Rhinolophidae	Rhinolophus mehelyi	VU	1.901	2.4513
2195	Carnivora	Felidae	Lynx rufus	LC	10.600	2.4510
2196	Chiroptera	Molossidae	Chaerephon jobensis	LC	10.569	2.4484
2196	Chiroptera	Molossidae	Chaerephon major	LC	10.569	2.4484
2196	Chiroptera	Molossidae	Chaerephon nigeriae	LC	10.569	2.4484
2196	Chiroptera	Molossidae	Chaerephon plicata	LC	10.569	2.4484
2196	Chiroptera	Molossidae	Chaerephon pumila	LC	10.569	2.4484
2201	Carnivora	Mustelidae	Ictonyx libyca	LC	10.569	2.4483
2201	Carnivora	Mustelidae	Ictonyx striatus	LC	10.569	2.4483
2203	Lagomorpha	Leporidae	Lepus nigricollis	LC	10.566	2.4481
2203	Lagomorpha	Leporidae	Lepus peguensis	LC	10.566	2.4481
2205	Carnivora	Mustelidae	Martes martes	LC	10.527	2.4447
2206	Chiroptera	Vespertilionidae	Murina aenea	NT	4.752	2.4427
2206	Chiroptera	Vespertilionidae	Murina huttoni	NT	4.752	2.4427
2206	Chiroptera	Vespertilionidae	Murina rozendaali	NT	4.752	2.4427
2209	Insectivora	Soricidae	Sorex merriami	LC	10.493	2.4417
2209	Insectivora	Soricidae	Sorex trowbridgii	LC	10.493	2.4417
2211	Chiroptera	Pteropodidae	Penthetor lucasi	LC	10.478	2.4405
2212	Dasyuromorphia	Dasyuridae	Planigale gilesi	LC	10.474	2.4400
2212	Dasyuromorphia	Dasyuridae	Planigale ingrami	LC	10.474	2.4400
2214	Artiodactyla	Cervidae	Capreolus capreolus	LC	10.469	2.4397
2214	Artiodactyla	Cervidae	Capreolus pygargus	LC	10.469	2.4397
2216	Rodentia	Muridae	Rattus adustus	VU	1.865	2.4387
2216	Rodentia	Muridae	Rattus burrus	VU	1.865	2.4387
2216	Rodentia	Muridae	Rattus mindorensis	VU	1.865	2.4387
2216	Rodentia	Muridae	Rattus mollicomulus	VU	1.865	2.4387
2216	Rodentia	Muridae	Rattus palmarum	VU	1.865	2.4387
2216	Rodentia	Muridae	Rattus sikkimensis	VU	1.865	2.4387
2216	Rodentia	Muridae	Rattus tawitawiensis	VU	1.865	2.4387

Rank	Order	Family	Species	IUCN	ED'	EDGE
2223	Carnivora	Mustelidae	Martes flavigula	LC	10.457	2.4386
2224	Dasyuromorphia	Dasyuridae	Myoictis melas	LC	10.454	2.4384
2224	Dasyuromorphia	Dasyuridae	Pseudantechinus ningbing	LC	10.454	2.4384
2226	Lagomorpha	Leporidae	Lepus brachyurus	LC	10.447	2.4378
2226	Lagomorpha	Leporidae	Lepus mandshuricus	LC	10.447	2.4378
2228	Artiodactyla	Bovidae	Naemorhedus crispus	CD	4.685	2.4310
2228	Artiodactyla	Bovidae	Naemorhedus goral	NT	4.685	2.4310
2230	Carnivora	Herpestidae	Herpestes edwardsii	LC	10.351	2.4293
2230	Carnivora	Herpestidae	Herpestes javanicus	LC	10.351	2.4293
2232	Rodentia	Muridae	Akodon lindberghi	VU	1.838	2.4292
2233	Primates	Cercopithecidae	Cercopithecus lhoesti	NT	4.657	2.4260
2234	Rodentia	Muridae	Tylomys mirae	LC	10.311	2.4258
2234	Rodentia	Muridae	Tylomys nudicaudus	LC	10.311	2.4258
2234	Rodentia	Muridae	Tylomys watsoni	LC	10.311	2.4258
2237	Rodentia	Sciuridae	Tamias merriami	LC	10.296	2.4244
2237	Rodentia	Sciuridae	Tamias obscurus	LC	10.296	2.4244
2239	Rodentia	Muridae	Uranomys ruddi	LC	10.293	2.4242
2240	Dasyuromorphia	Dasyuridae	Antechinus stuartii	LC	10.292	2.4241
2241	Chiroptera	Vespertilionidae	Vespertilio murinus	LC	10.264	2.4216
2241	Chiroptera	Vespertilionidae	Vespertilio superans	LC	10.264	2.4216
2243	Rodentia	Caviidae	Microcavia australis	LC	10.256	2.4209
2243	Rodentia	Caviidae	Microcavia niata	LC	10.256	2.4209
2243	Rodentia	Caviidae	Microcavia shiptoni	LC	10.256	2.4209
2246	Chiroptera	Phyllostomidae	Vampyrodes caraccioli	LC	10.249	2.4203
2247	Rodentia	Sciuridae	Cynomys parvidens	CD	4.623	2.4201
2248	Primates	Cercopithecidae	Presbytis thomasi	NT	4.618	2.4190
2249	Rodentia	Muridae	Dephomys defua	LC	10.207	2.4166
2249	Rodentia	Muridae	Dephomys eburnea	LC	10.207	2.4166
2251	Rodentia	Muridae	Conilurus penicillatus	LC	10.196	2.4155
2252	Rodentia	Heteromyidae	Dipodomys stephensi	CD	4.582	2.4127
2253	Carnivora	Mustelidae	Mephitis macroura	LC	10.158	2.4121
2253	Carnivora	Mustelidae	Mephitis mephitis	LC	10.158	2.4121
2253	Carnivora	Mustelidae	Spilogale putorius	LC	10.158	2.4121
2253	Carnivora	Mustelidae	Spilogale pygmaea	LC	10.158	2.4121
2257	Chiroptera	Pteropodidae	Megaloglossus woermanni	LC	10.138	2.4104
2257	Chiroptera	Pteropodidae	Rousettus angolensis	LC	10.138	2.4104
2259	Rodentia	Muridae	Chroeomys andinus	LC	10.135	2.4100
2259	Rodentia	Muridae	Chroeomys jelskii	LC	10.135	2.4100
2261	Carnivora	Viverridae	Prionodon linsang	LC	10.112	2.4080
2261	Carnivora	Viverridae	Prionodon pardicolor	LC	10.112	2.4080
2263	Rodentia	Sciuridae	Sciurus sanborni	NT	4.552	2.4073
2264	Carnivora	Felidae	Leopardus pardalis	LC	10.056	2.4030
2264	Carnivora	Felidae	Leopardus wiedii	LC	10.056	2.4030
2266	Carnivora	Mustelidae	Conepatus humboldtii	LC	10.047	2.4022
2266	Carnivora	Mustelidae	Conepatus semistriatus	LC	10.047	2.4022
2268	Rodentia	Sciuridae	Tamias alpinus	LC	10.040	2.4016
2268	Rodentia	Sciuridae	Tamias minimus	LC	10.040	2.4016
2270	Rodentia	Dipodidae	Stylodipus andrewsi	LC	10.036	2.4012
2270	Rodentia	Dipodidae	Stylodipus sungorus	LC	10.036	2.4012
2270	Rodentia	Dipodidae	Stylodipus telum	LC	10.036	2.4012
2273	Rodentia	Muridae	Notomys alexis	LC	10.035	2.4010
2273	Rodentia	Muridae	Notomys mitchellii	LC	10.035	2.4010
2275	Diprotodontia	Phalangeridae	Phalanger carmelitae	LC	10.034	2.4009
2276	Peramelemorphia	Peramelidae	Isoodon obesulus	LC	10.011	2.3989
2277	Dasyuromorphia	Dasyuridae	Sminthopsis virginiae	LC	10.009	2.3987

Rank	Order	Family	Species	IUCN	ED'	EDGE
2278	Rodentia	Dipodidae	Jaculus blanfordi	LC	10.008	2.3986
2278	Rodentia	Dipodidae	Jaculus jaculus	LC	10.008	2.3986
2278	Rodentia	Dipodidae	Jaculus orientalis	LC	10.008	2.3986
2278	Rodentia	Dipodidae	Jaculus turcmenicus	LC	10.008	2.3986
2282	Chiroptera	Vespertilionidae	Nycticeius balstoni	LC	9.991	2.3971
2283	Dasyuromorphia	Dasyuridae	Dasykaluta rosamondae	LC	9.987	2.3967
2284	Dasyuromorphia	Dasyuridae	Antechinus melanurus	LC	9.979	2.3959
2285	Rodentia	Muridae	Dasymys nudipes	NT	4.487	2.3955
2286	Dasyuromorphia	Dasyuridae	Parantechinus bilarni	LC	9.971	2.3953
2287	Carnivora	Mustelidae	Martes zibellina	LC	9.961	2.3944
2288	Chiroptera	Phyllostomidae	Sturnira lilium	LC	9.961	2.3943
2289	Chiroptera	Vespertilionidae	Pipistrellus ceylonicus	LC	9.952	2.3935
2290	Chiroptera	Vespertilionidae	Myotis goudoti	NT	4.475	2.3933
2291	Rodentia	Muridae	Megadontomys nelsoni	LC	9.942	2.3926
2291	Rodentia	Muridae	Megadontomys thomasi	LC	9.942	2.3926
2293	Rodentia	Bathyergidae	Cryptomys damarensis	LC	9.942	2.3926
2293	Rodentia	Bathyergidae	Cryptomys hottentotus	LC	9.942	2.3926
2293	Rodentia	Bathyergidae	Cryptomys mechowi	LC	9.942	2.3926
2293	Rodentia	Bathyergidae	Cryptomys zechi	LC	9.942	2.3926
2297	Rodentia	Muridae	Microtus thomasi	NT	4.466	2.3916
2298	Diprotodontia	Macropodidae	Petrogale inornata	LC	9.928	2.3913
2298	Diprotodontia	Macropodidae	Petrogale penicillata	LC	9.928	2.3913
2300	Diprotodontia	Macropodidae	Macropus robustus	LC	9.914	2.3900
2301	Chiroptera	Pteropodidae	Pteropus chrysoproctus	NT	4.445	2.3878
2302	Carnivora	Herpestidae	Mungos mungo	LC	9.840	2.3833
2303	Carnivora	Herpestidae	Bdeogale crassicauda	LC	9.839	2.3832
2303	Carnivora	Herpestidae	Bdeogale nigripes	LC	9.839	2.3832
2305	Primates	Cebidae	Ateles geoffroyi	LC	9.838	2.3831
2306	Carnivora	Phocidae	Phoca fasciata	LC	9.825	2.3818
2306	Carnivora	Phocidae	Phoca groenlandica	LC	9.825	2.3818
2308	Chiroptera	Phyllostomidae	Artibeus obscurus	NT	4.404	2.3803
2309	Rodentia	Muridae	Rattus giluwensis	NT	4.402	2.3800
2309	Rodentia	Muridae	Rattus jobiensis	NT	4.402	2.3800
2311	Primates	Cebidae	Pithecia aequatorialis	LC	9.773	2.3770
2311	Primates	Cebidae	Pithecia pithecia	LC	9.773	2.3770
2313	Diprotodontia	Petauridae	Petaurus breviceps	LC	9.767	2.3765
2314	Lagomorpha	Leporidae	Sylvilagus floridanus	LC	9.754	2.3753
2315	Rodentia	Muridae	Microtus sachalinensis	NT	4.371	2.3741
2316	Rodentia	Sciuridae	Funambulus layardi	LC	9.739	2.3738
2316	Rodentia	Sciuridae	Funambulus palmarum	LC	9.739	2.3738
2316	Rodentia	Sciuridae	Funambulus sublineatus	LC	9.739	2.3738
2319	Artiodactyla	Bovidae	Connochaetes gnou	LC	9.737	2.3737
2320	Carnivora	Mustelidae	Mustela altaica	LC	9.735	2.3735
2320	Carnivora	Mustelidae	Mustela erminea	LC	9.735	2.3735
2322	Lagomorpha	Leporidae	Lepus saxatilis	LC	9.728	2.3728
2322	Lagomorpha	Leporidae	Lepus victoriae	LC	9.728	2.3728
2324	Carnivora	Mustelidae	Martes americana	LC	9.719	2.3720
2324	Carnivora	Mustelidae	Martes melampus	LC	9.719	2.3720
2326	Primates	Cebidae	Cebus apella	LC	9.713	2.3714
2327	Rodentia	Muridae	Abditomys latidens	LC	9.706	2.3708
2327	Rodentia	Muridae	Limnomys sibuanus	LC	9.706	2.3708
2329	Chiroptera	Vespertilionidae	Rhogeessa minutilla	NT	4.349	2.3701
2329	Chiroptera	Vespertilionidae	Rhogeessa parvula	NT	4.349	2.3701
2331	Rodentia	Muridae	Thallomys loringi	LC	9.694	2.3697
2331	Rodentia	Muridae	Thallomys nigricauda	LC	9.694	2.3697
2331	Rodentia	Muridae	Thallomys paedulcus	LC	9.694	2.3697

Rank	Order	Family	Species	IUCN	ED'	EDGE
2334	Rodentia	Muridae	Habromys chinanteco	LC	9.684	2.3687
2334	Rodentia	Muridae	Habromys lophurus	LC	9.684	2.3687
2336	Rodentia	Muridae	Aethomys bocagei	LC	9.675	2.3679
2336	Rodentia	Muridae	Aethomys chrysophilus	LC	9.675	2.3679
2336	Rodentia	Muridae	Aethomys granti	LC	9.675	2.3679
2336	Rodentia	Muridae	Aethomys hindei	LC	9.675	2.3679
2336	Rodentia	Muridae	Aethomys kaiseri	LC	9.675	2.3679
2336	Rodentia	Muridae	Aethomys namaquensis	LC	9.675	2.3679
2336	Rodentia	Muridae	Aethomys nyikae	LC	9.675	2.3679
2336	Rodentia	Muridae	Aethomys thomasi	LC	9.675	2.3679
2344	Carnivora	Viverridae	Genetta genetta	LC	9.673	2.3677
2344	Carnivora	Viverridae	Genetta tigrina	LC	9.673	2.3677
2346	Cetacea	Delphinidae	Globicephala melas	LC	9.665	2.3670
2347	Rodentia	Caviidae	Cavia aperea	LC	9.660	2.3665
2347	Rodentia	Caviidae	Cavia fulgida	LC	9.660	2.3665
2347	Rodentia	Caviidae	Cavia magna	LC	9.660	2.3665
2347	Rodentia	Caviidae	Cavia porcellus	LC	9.660	2.3665
2347	Rodentia	Caviidae	Cavia tschudii	LC	9.660	2.3665
2352	Chiroptera	Vespertilionidae	Pipistrellus imbricatus	LC	9.658	2.3663
2353	Rodentia	Muridae	Haeromys minahassae	LC	9.636	2.3642
2354	Rodentia	Sciuridae	Spermophilus perotensis	NT	4.307	2.3623
2355	Rodentia	Muridae	Myomys daltoni	LC	9.606	2.3614
2355	Rodentia	Muridae	Myomys derooi	LC	9.606	2.3614
2355	Rodentia	Muridae	Myomys fumatus	LC	9.606	2.3614
2355	Rodentia	Muridae	Myomys verreauxii	LC	9.606	2.3614
2355	Rodentia	Muridae	Myomys yemeni	LC	9.606	2.3614
2360	Rodentia	Muridae	Bibimys torresi	NT	4.302	2.3612
2361	Rodentia	Muridae	Microtus nasarovi	NT	4.290	2.3589
2361	Rodentia	Muridae	Microtus tatricus	NT	4.290	2.3589
2363	Artiodactyla	Bovidae	Pseudois nayaur	LC	9.570	2.3580
2364	Insectivora	Soricidae	Crocidura ansellorum	EN	0.320	2.3570
2364	Insectivora	Soricidae	Crocidura beccarii	EN	0.320	2.3570
2364	Insectivora	Soricidae	Crocidura bottegoides	EN	0.320	2.3570
2364	Insectivora	Soricidae	Crocidura desperata	EN	0.320	2.3570
2364	Insectivora	Soricidae	Crocidura grandis	EN	0.320	2.3570
2364	Insectivora	Soricidae	Crocidura hispida	EN	0.320	2.3570
2364	Insectivora	Soricidae	Crocidura malayana	EN	0.320	2.3570
2364	Insectivora	Soricidae	Crocidura mindorus	EN	0.320	2.3570
2364	Insectivora	Soricidae	Crocidura miya	EN	0.320	2.3570
2364	Insectivora	Soricidae	Crocidura orii	EN	0.320	2.3570
2364	Insectivora	Soricidae	Crocidura paradoxura	EN	0.320	2.3570
2364	Insectivora	Soricidae	Crocidura picea	EN	0.320	2.3570
2364	Insectivora	Soricidae	Crocidura susiana	EN	0.320	2.3570
2364	Insectivora	Soricidae	Crocidura telfordi	EN	0.320	2.3570
2364	Insectivora	Soricidae	Crocidura usambarae	EN	0.320	2.3570
2379	Rodentia	Erethizontidae	Echinoprocta rufescens	LC	9.547	2.3558
2379	Rodentia	Erethizontidae	Erethizon dorsatum	LC	9.547	2.3558
2381	Rodentia	Sciuridae	Sciurus richmondi	NT	4.262	2.3536
2382	Chiroptera	Rhinolophidae	Asellia tridens	LC	9.523	2.3535
2383	Dasyuromorphia	Dasyuridae	Murexia longicaudata	LC	9.505	2.3519
2384	Rodentia	Heteromyidae	Perognathus fasciatus	LC	9.494	2.3508
2384	Rodentia	Heteromyidae	Perognathus flavescens	LC	9.494	2.3508
2384	Rodentia	Heteromyidae	Perognathus flavus	LC	9.494	2.3508
2384	Rodentia	Heteromyidae	Perognathus inornatus	LC	9.494	2.3508
2384	Rodentia	Heteromyidae	Perognathus longimembris	LC	9.494	2.3508
2384	Rodentia	Heteromyidae	Perognathus merriami	LC	9.494	2.3508

Rank	Order	Family	Species	IUCN	ED'	EDGE
2384	Rodentia	Heteromyidae	Perognathus parvus	LC	9.494	2.3508
2384	Rodentia	Heteromyidae	Perognathus xanthanotus	LC	9.494	2.3508
2392	Chiroptera	Vespertilionidae	Scotophilus nigrita	NT	4.242	2.3499
2393	Rodentia	Muridae	Taterillus arenarius	LC	9.475	2.3490
2393	Rodentia	Muridae	Taterillus congicus	LC	9.475	2.3490
2393	Rodentia	Muridae	Taterillus emini	LC	9.475	2.3490
2393	Rodentia	Muridae	Taterillus gracilis	LC	9.475	2.3490
2393	Rodentia	Muridae	Taterillus harringtoni	LC	9.475	2.3490
2393	Rodentia	Muridae	Taterillus lacustris	LC	9.475	2.3490
2393	Rodentia	Muridae	Taterillus petteri	LC	9.475	2.3490
2393	Rodentia	Muridae	Taterillus pygargus	LC	9.475	2.3490
2401	Chiroptera	Pteropodidae	Pteropus scapulatus	LC	9.468	2.3483
2401	Chiroptera	Pteropodidae	Pteropus woodfordi	LC	9.468	2.3483
2403	Rodentia	Muridae	Coccymys ruemmleri	LC	9.465	2.3480
2403	Rodentia	Muridae	Hyomys dammermani	LC	9.465	2.3480
2403	Rodentia	Muridae	Hyomys goliath	LC	9.465	2.3480
2403	Rodentia	Muridae	Microhydromys musseri	LC	9.465	2.3480
2407	Diprotodontia	Macropodidae	Dorcopsulus vanheurni	LC	9.465	2.3480
2408	Chiroptera	Vespertilionidae	Plecotus mexicanus	LC	9.456	2.3472
2409	Rodentia	Muridae	Lagurus lagurus	LC	9.424	2.3441
2410	Rodentia	Heteromyidae	Chaetodipus penicillatus	LC	9.422	2.3439
2411	Rodentia	Sciuridae	Tamias bulleri	LC	9.377	2.3395
2412	Rodentia	Sciuridae	Cynomys ludovicianus	NT	4.177	2.3374
2413	Insectivora	Soricidae	Sorex samniticus	LC	9.354	2.3374
2414	Chiroptera	Phyllostomidae	Tonatia bidens	LC	9.348	2.3368
2414	Chiroptera	Phyllostomidae	Tonatia brasiliense	LC	9.348	2.3368
2414	Chiroptera	Phyllostomidae	Tonatia silvicola	LC	9.348	2.3368
2417	Chiroptera	Phyllostomidae	Micronycteris minuta	LC	9.345	2.3365
2417	Chiroptera	Phyllostomidae	Micronycteris schmidtorum	LC	9.345	2.3365
2419	Lagomorpha	Leporidae	Lepus starcki	LC	9.330	2.3351
2420	Chiroptera	Phyllostomidae	Carollia brevicauda	LC	9.305	2.3326
2420	Chiroptera	Phyllostomidae	Carollia castanea	LC	9.305	2.3326
2420	Chiroptera	Phyllostomidae	Carollia perspicillata	LC	9.305	2.3326
2420	Chiroptera	Phyllostomidae	Carollia subrufa	LC	9.305	2.3326
2424	Rodentia	Sciuridae	Tamiasciurus mearnsi	LC	9.285	2.3307
2425	Carnivora	Mustelidae	Conepatus chinga	LC	9.281	2.3303
2425	Carnivora	Mustelidae	Conepatus leuconotus	LC	9.281	2.3303
2425	Carnivora	Mustelidae	Conepatus mesoleucus	LC	9.281	2.3303
2428	Artiodactyla	Bovidae	Gazella granti	CD	4.126	2.3275
2429	Rodentia	Heteromyidae	Heteromys anomalus	LC	9.233	2.3257
2430	Artiodactyla	Bovidae	Madoqua guentheri	LC	9.232	2.3256
2430	Artiodactyla	Bovidae	Madoqua kirkii	LC	9.232	2.3256
2430	Artiodactyla	Bovidae	Madoqua saltiana	LC	9.232	2.3256
2433	Artiodactyla	Cervidae	Dama dama	LC	9.217	2.3240
2434	Chiroptera	Vespertilionidae	Chalinolobus gouldii	LC	9.216	2.3240
2434	Chiroptera	Vespertilionidae	Chalinolobus morio	LC	9.216	2.3240
2434	Chiroptera	Vespertilionidae	Chalinolobus nigrogriseus	LC	9.216	2.3240
2437	Diprotodontia	Macropodidae	Macropus fuliginosus	LC	9.204	2.3228
2437	Diprotodontia	Macropodidae	Macropus giganteus	LC	9.204	2.3228
2439	Chiroptera	Rhinolophidae	Hipposideros abae	NT	4.100	2.3224
2439	Chiroptera	Rhinolophidae	Hipposideros fuliginosus	NT	4.100	2.3224
2439	Chiroptera	Rhinolophidae	Hipposideros halophyllus	NT	4.100	2.3224
2439	Chiroptera	Rhinolophidae	Hipposideros jonesi	NT	4.100	2.3224
2439	Chiroptera	Rhinolophidae	Hipposideros macrobullatus	NT	4.100	2.3224
2439	Chiroptera	Rhinolophidae	Hipposideros megalotis	NT	4.100	2.3224
2439	Chiroptera	Rhinolophidae	Hipposideros obscurus	NT	4.100	2.3224

Rank	Order	Family	Species	IUCN	ED'	EDGE
2439	Chiroptera	Rhinolophidae	Hipposideros pygmaeus	NT	4.100	2.3224
2447	Primates	Cebidae	Callicebus dubius	LC	9.187	2.3211
2448	Rodentia	Muridae	Juscelinomys candango	NT	4.084	2.3193
2449	Rodentia	Sciuridae	Heliosciurus gambianus	LC	9.134	2.3159
2449	Rodentia	Sciuridae	Heliosciurus mutabilis	LC	9.134	2.3159
2449	Rodentia	Sciuridae	Heliosciurus rufobrachium	LC	9.134	2.3159
2449	Rodentia	Sciuridae	Heliosciurus ruwenzorii	LC	9.134	2.3159
2453	Rodentia	Heteromyidae	Chaetodipus intermedius	LC	9.115	2.3140
2453	Rodentia	Heteromyidae	Chaetodipus nelsoni	LC	9.115	2.3140
2455	Chiroptera	Vespertilionidae	Scotophilus robustus	NT	4.045	2.3116
2456	Chiroptera	Phyllostomidae	Anoura caudifera	LC	9.070	2.3096
2456	Chiroptera	Phyllostomidae	Anoura cultrata	LC	9.070	2.3096
2456	Chiroptera	Phyllostomidae	Anoura geoffroyi	LC	9.070	2.3096
2459	Rodentia	Muridae	Zyzomys argurus	LC	9.061	2.3087
2459	Rodentia	Muridae	Zyzomys maini	LC	9.061	2.3087
2459	Rodentia	Muridae	Zyzomys woodwardi	LC	9.061	2.3087
2462	Rodentia	Geomyidae	Geomys bursarius	LC	9.058	2.3084
2463	Chiroptera	Pteropodidae	Myonycteris torquata	LC	9.043	2.3069
2464	Rodentia	Sciuridae	Petaurista petaurista	LC	9.026	2.3052
2464	Rodentia	Sciuridae	Petaurista philippensis	LC	9.026	2.3052
2466	Rodentia	Muridae	Synaptomys borealis	LC	9.019	2.3045
2466	Rodentia	Muridae	Synaptomys cooperi	LC	9.019	2.3045
2468	Artiodactyla	Bovidae	Cephalophus harveyi	CD	3.995	2.3016
2468	Artiodactyla	Bovidae	Cephalophus natalensis	CD	3.995	2.3016
2470	Cetacea	Delphinidae	Lagenorhynchus cruciger	LC	8.966	2.2992
2471	Insectivora	Soricidae	Sorex roboratus	LC	8.965	2.2991
2472	Rodentia	Muridae	Microtus agrestis	LC	8.962	2.2988
2473	Chiroptera	Phyllostomidae	Glossophaga commissarisi	LC	8.952	2.2978
2473	Chiroptera	Phyllostomidae	Glossophaga soricina	LC	8.952	2.2978
2475	Carnivora	Otariidae	Otaria byronia	LC	8.942	2.2968
2476	Primates	Cebidae	Aotus azarai	LC	8.925	2.2951
2476	Primates	Cebidae	Aotus infulatus	LC	8.925	2.2951
2476	Primates	Cebidae	Aotus nancymaae	LC	8.925	2.2951
2476	Primates	Cebidae	Aotus nigriceps	LC	8.925	2.2951
2476	Primates	Cebidae	Aotus trivirgatus	LC	8.925	2.2951
2476	Primates	Cebidae	Aotus vociferans	LC	8.925	2.2951
2482	Dasyuromorphia	Dasyuridae	Sminthopsis dolichura	LC	8.912	2.2938
2482	Dasyuromorphia	Dasyuridae	Sminthopsis gilberti	LC	8.912	2.2938
2482	Dasyuromorphia	Dasyuridae	Sminthopsis murina	LC	8.912	2.2938
2485	Primates	Cercopithecidae	Macaca radiata	LC	8.910	2.2935
2486	Diprotodontia	Macropodidae	Thylogale billardierii	LC	8.908	2.2933
2487	Rodentia	Sciuridae	Petaurista alborufus	LC	8.887	2.2912
2488	Insectivora	Soricidae	Sorex gracillimus	LC	8.850	2.2874
2489	Carnivora	Canidae	Otocyon megalotis	LC	8.822	2.2846
2490	Rodentia	Muridae	Aepeomys fuscatus	LC	8.796	2.2820
2490	Rodentia	Muridae	Aepeomys lugens	LC	8.796	2.2820
2492	Rodentia	Muridae	Chiruromys forbesi	LC	8.794	2.2817
2492	Rodentia	Muridae	Chiruromys lamia	LC	8.794	2.2817
2492	Rodentia	Muridae	Chiruromys vates	LC	8.794	2.2817
2492	Rodentia	Muridae	Pogonomelomys mayeri	LC	8.794	2.2817
2492	Rodentia	Muridae	Pogonomelomys sevia	LC	8.794	2.2817
2497	Primates	Callitrichidae	Saguinus inustus	LC	8.785	2.2808
2498	Rodentia	Muridae	Mallomys aroaensis	LC	8.778	2.2801
2498	Rodentia	Muridae	Mallomys rothschildi	LC	8.778	2.2801
2500	Chiroptera	Vespertilionidae	Myotis brandti	LC	8.773	2.2796
2500	Chiroptera	Vespertilionidae	Myotis mystacinus	LC	8.773	2.2796

Rank	Order	Family	Species	IUCN	ED'	EDGE
2502	Rodentia	Sciuridae	Funisciurus carruthersi	LC	8.769	2.2792
2502	Rodentia	Sciuridae	Funisciurus congicus	LC	8.769	2.2792
2502	Rodentia	Sciuridae	Funisciurus isabella	LC	8.769	2.2792
2502	Rodentia	Sciuridae	Funisciurus pyrropus	LC	8.769	2.2792
2506	Insectivora	Soricidae	Sylvisorex granti	LC	8.763	2.2786
2506	Insectivora	Soricidae	Sylvisorex johnstoni	LC	8.763	2.2786
2506	Insectivora	Soricidae	Sylvisorex lunaris	LC	8.763	2.2786
2506	Insectivora	Soricidae	Sylvisorex megalura	LC	8.763	2.2786
2506	Insectivora	Soricidae	Sylvisorex ollula	LC	8.763	2.2786
2506	Insectivora	Soricidae	Sylvisorex vulcanorum	LC	8.763	2.2786
2512	Rodentia	Sciuridae	Spermophilus elegans	LC	8.762	2.2785
2513	Chiroptera	Vespertilionidae	Eptesicus capensis	LC	8.759	2.2782
2513	Chiroptera	Vespertilionidae	Eptesicus melckorum	LC	8.759	2.2782
2513	Chiroptera	Vespertilionidae	Eptesicus somalicus	LC	8.759	2.2782
2516	Rodentia	Erethizontidae	Sphiggurus insidiosus	LC	8.757	2.2780
2516	Rodentia	Erethizontidae	Sphiggurus mexicanus	LC	8.757	2.2780
2516	Rodentia	Erethizontidae	Sphiggurus spinosus	LC	8.757	2.2780
2516	Rodentia	Erethizontidae	Sphiggurus villosus	LC	8.757	2.2780
2520	Rodentia	Muridae	Gerbillurus paeba	LC	8.736	2.2758
2520	Rodentia	Muridae	Gerbillurus setzeri	LC	8.736	2.2758
2520	Rodentia	Muridae	Gerbillurus tytonis	LC	8.736	2.2758
2520	Rodentia	Muridae	Gerbillurus vallinus	LC	8.736	2.2758
2524	Carnivora	Viverridae	Viverra tangalunga	LC	8.733	2.2755
2524	Carnivora	Viverridae	Viverra zibetha	LC	8.733	2.2755
2526	Rodentia	Capromyidae	Mysateles prehensilis	LC	8.726	2.2748
2527	Rodentia	Muridae	Paraleptomys rufilatus	LC	8.702	2.2723
2528	Chiroptera	Pteropodidae	Nyctimene major	LC	8.689	2.2710
2528	Chiroptera	Pteropodidae	Nyctimene robinsoni	LC	8.689	2.2710
2530	Primates	Cercopithecidae	Cercopithecus neglectus	LC	8.677	2.2698
2531	Rodentia	Sciuridae	Spermophilus mexicanus	LC	8.665	2.2685
2532	Rodentia	Myoxidae	Graphiurus lorraineus	LC	8.655	2.2674
2532	Rodentia	Myoxidae	Graphiurus microtis	LC	8.655	2.2674
2532	Rodentia	Myoxidae	Graphiurus murinus	LC	8.655	2.2674
2532	Rodentia	Myoxidae	Graphiurus ocularis	LC	8.655	2.2674
2532	Rodentia	Myoxidae	Graphiurus parvus	LC	8.655	2.2674
2532	Rodentia	Myoxidae	Graphiurus platyops	LC	8.655	2.2674
2532	Rodentia	Myoxidae	Graphiurus rupicola	LC	8.655	2.2674
2539	Rodentia	Muridae	Clethrionomys sikotanensis	NT	3.825	2.2670
2540	Chiroptera	Vespertilionidae	Myotis annectans	NT	3.824	2.2667
2540	Chiroptera	Vespertilionidae	Myotis ridleyi	NT	3.824	2.2667
2540	Chiroptera	Vespertilionidae	Myotis rosseti	NT	3.824	2.2667
2543	Artiodactyla	Suidae	Sus barbatus	LC	8.642	2.2662
2543	Artiodactyla	Suidae	Sus celebensis	LC	8.642	2.2662
2543	Artiodactyla	Suidae	Sus scrofa	LC	8.642	2.2662
2546	Carnivora	Viverridae	Viverra megaspila	LC	8.624	2.2642
2547	Rodentia	Sciuridae	Marmota bobak	CD	3.801	2.2619
2548	Diprotodontia	Macropodidae	Macropus antilopinus	LC	8.598	2.2615
2548	Diprotodontia	Macropodidae	Macropus rufus	LC	8.598	2.2615
2550	Rodentia	Sciuridae	Hylopetes lepidus	LC	8.597	2.2615
2551	Diprotodontia	Macropodidae	Dorcopsis hageni	LC	8.594	2.2612
2551	Diprotodontia	Macropodidae	Dorcopsis luctuosa	LC	8.594	2.2612
2551	Diprotodontia	Macropodidae	Dorcopsis muelleri	LC	8.594	2.2612
2554	Chiroptera	Rhinolophidae	Hipposideros cyclops	LC	8.590	2.2608
2555	Chiroptera	Vespertilionidae	Eptesicus rendalli	LC	8.578	2.2595
2555	Chiroptera	Vespertilionidae	Eptesicus tenuipinnis	LC	8.578	2.2595

Rank	Order	Family	Species	IUCN	ED'	EDGE
2557	Chiroptera	Vespertilionidae	Hesperoptenus blanfordi	LC	8.567	2.2583
2557	Chiroptera	Vespertilionidae	Hesperoptenus tickelli	LC	8.567	2.2583
2557	Chiroptera	Vespertilionidae	Hesperoptenus tomesi	LC	8.567	2.2583
2557	Chiroptera	Vespertilionidae	Histiotus montanus	LC	8.567	2.2583
2557	Chiroptera	Vespertilionidae	Histiotus velatus	LC	8.567	2.2583
2557	Chiroptera	Vespertilionidae	Laephotis botswanae	LC	8.567	2.2583
2557	Chiroptera	Vespertilionidae	Laephotis namibensis	LC	8.567	2.2583
2557	Chiroptera	Vespertilionidae	Laephotis wintoni	LC	8.567	2.2583
2565	Primates	Cercopithecidae	Erythrocebus patas	LC	8.549	2.2565
2566	Rodentia	Sciuridae	Aeromys tephromelas	LC	8.545	2.2560
2566	Rodentia	Sciuridae	Aeromys thomasi	LC	8.545	2.2560
2568	Rodentia	Echimyidae	Kannabateomys amblyonyx	LC	8.543	2.2558
2569	Rodentia	Muridae	Lemmus amurensis	LC	8.543	2.2558
2569	Rodentia	Muridae	Lemmus lemmus	LC	8.543	2.2558
2569	Rodentia	Muridae	Lemmus sibiricus	LC	8.543	2.2558
2572	Diprotodontia	Macropodidae	Petrogale lateralis	LC	8.535	2.2549
2572	Diprotodontia	Macropodidae	Petrogale rothschildi	LC	8.535	2.2549
2574	Rodentia	Muridae	Microtus chrotorrhinus	LC	8.526	2.2540
2575	Carnivora	Mustelidae	Mustela frenata	LC	8.523	2.2537
2575	Carnivora	Mustelidae	Mustela nivalis	LC	8.523	2.2537
2577	Rodentia	Hystricidae	Hystrix africaeaustralis	LC	8.515	2.2528
2577	Rodentia	Hystricidae	Hystrix cristata	LC	8.515	2.2528
2577	Rodentia	Hystricidae	Hystrix indica	LC	8.515	2.2528
2577	Rodentia	Hystricidae	Hystrix javanica	LC	8.515	2.2528
2577	Rodentia	Hystricidae	Hystrix pumila	LC	8.515	2.2528
2577	Rodentia	Hystricidae	Hystrix sumatrae	LC	8.515	2.2528
2583	Artiodactyla	Bovidae	Gazella thomsonii	CD	3.757	2.2528
2584	Chiroptera	Pteropodidae	Eidolon dupreanum	LC	8.513	2.2526
2584	Chiroptera	Pteropodidae	Eidolon helvum	LC	8.513	2.2526
2586	Rodentia	Sciuridae	Spermophilus saturatus	LC	8.505	2.2518
2587	Rodentia	Muridae	Alticola albicauda	NT	3.752	2.2518
2587	Rodentia	Muridae	Alticola roylei	NT	3.752	2.2518
2589	Rodentia	Muridae	Cremnomys blanfordi	LC	8.504	2.2517
2589	Rodentia	Muridae	Cremnomys cutchicus	LC	8.504	2.2517
2591	Chiroptera	Vespertilionidae	Pipistrellus aegyptius	LC	8.496	2.2509
2591	Chiroptera	Vespertilionidae	Pipistrellus kuhlii	LC	8.496	2.2509
2591	Chiroptera	Vespertilionidae	Pipistrellus rusticus	LC	8.496	2.2509
2594	Chiroptera	Vespertilionidae	Eptesicus pachyotis	NT	3.744	2.2501
2595	Cetacea	Delphinidae	Delphinus delphis	LC	8.478	2.2490
2596	Carnivora	Herpestidae	Crossarchus alexandri	LC	8.458	2.2468
2596	Carnivora	Herpestidae	Crossarchus ansorgei	LC	8.458	2.2468
2596	Carnivora	Herpestidae	Crossarchus obscurus	LC	8.458	2.2468
2599	Rodentia	Heteromyidae	Heteromys australis	LC	8.456	2.2467
2600	Rodentia	Muridae	Neotoma palatina	NT	3.717	2.2443
2601	Chiroptera	Phyllostomidae	Vampyressa pusilla	LC	8.427	2.2435
2602	Chiroptera	Vespertilionidae	Chalinolobus beatrix	NT	3.711	2.2431
2602	Chiroptera	Vespertilionidae	Chalinolobus egeria	NT	3.711	2.2431
2602	Chiroptera	Vespertilionidae	Chalinolobus gleni	NT	3.711	2.2431
2602	Chiroptera	Vespertilionidae	Chalinolobus poensis	NT	3.711	2.2431
2606	Rodentia	Sciuridae	Marmota caudata	NT	3.703	2.2413
2607	Rodentia	Muridae	Rhipidomys caucensis	NT	3.700	2.2407
2607	Rodentia	Muridae	Rhipidomys ochrogaster	NT	3.700	2.2407
2609	Rodentia	Muridae	Ellobius fuscocapillus	LC	8.390	2.2396
2609	Rodentia	Muridae	Ellobius lutescens	LC	8.390	2.2396
2609	Rodentia	Muridae	Ellobius talpinus	LC	8.390	2.2396
2609	Rodentia	Muridae	Ellobius tancrei	LC	8.390	2.2396

Rank	Order	Family	Species	IUCN	ED'	EDGE
2613	Carnivora	Felidae	Panthera pardus	LC	8.381	2.2387
2614	Rodentia	Muridae	Bunomys andrewsi	LC	8.375	2.2380
2614	Rodentia	Muridae	Bunomys chrysocomus	LC	8.375	2.2380
2614	Rodentia	Muridae	Bunomys penitus	LC	8.375	2.2380
2617	Rodentia	Muridae	Hydromys chrysogaster	LC	8.369	2.2374
2618	Carnivora	Canidae	Chrysocyon brachyurus	NT	3.683	2.2371
2619	Artiodactyla	Bovidae	Cephalophus rufilatus	CD	3.682	2.2369
2619	Artiodactyla	Bovidae	Cephalophus nigrifrons	NT	3.682	2.2369
2621	Carnivora	Viverridae	Genetta angolensis	LC	8.362	2.2367
2621	Carnivora	Viverridae	Genetta maculata	LC	8.362	2.2367
2623	Chiroptera	Vespertilionidae	Pipistrellus savii	LC	8.361	2.2365
2624	Chiroptera	Vespertilionidae	Nyctalus aviator	NT	3.671	2.2346
2624	Chiroptera	Vespertilionidae	Nyctalus lasiopterus	NT	3.671	2.2346
2624	Chiroptera	Vespertilionidae	Nyctalus leisleri	NT	3.671	2.2346
2624	Chiroptera	Vespertilionidae	Nyctalus montanus	NT	3.671	2.2346
2628	Artiodactyla	Bovidae	Sylvicapra grimmia	LC	8.326	2.2328
2629	Rodentia	Muridae	Lophuromys medicaudatus	NT	3.643	2.2284
2629	Rodentia	Muridae	Lophuromys rahmi	NT	3.643	2.2284
2631	Diprotodontia	Macropodidae	Macropus dorsalis	LC	8.277	2.2275
2632	Lagomorpha	Leporidae	Lepus capensis	LC	8.265	2.2262
2633	Rodentia	Muridae	Taeromys callitrichus	LC	8.263	2.2260
2633	Rodentia	Muridae	Taeromys celebensis	LC	8.263	2.2260
2633	Rodentia	Muridae	Taeromys taerae	LC	8.263	2.2260
2636	Rodentia	Muridae	Batomys granti	LC	8.255	2.2252
2636	Rodentia	Muridae	Batomys salomonseni	LC	8.255	2.2252
2638	Rodentia	Muridae	Millardia gleadowi	LC	8.254	2.2250
2638	Rodentia	Muridae	Millardia kathleenae	LC	8.254	2.2250
2638	Rodentia	Muridae	Millardia meltada	LC	8.254	2.2250
2641	Rodentia	Muridae	Uromys caudimaculatus	LC	8.232	2.2227
2641	Rodentia	Muridae	Uromys neobritanicus	LC	8.232	2.2227
2643	Artiodactyla	Cervidae	Axis axis	LC	8.228	2.2223
2644	Chiroptera	Vespertilionidae	Nycticeius greyii	LC	8.227	2.2221
2644	Chiroptera	Vespertilionidae	Nycticeius sanborni	LC	8.227	2.2221
2646	Chiroptera	Pteropodidae	Cynopterus brachyotis	LC	8.217	2.2210
2646	Chiroptera	Pteropodidae	Cynopterus sphinx	LC	8.217	2.2210
2648	Chiroptera	Phyllostomidae	Glossophaga leachii	LC	8.216	2.2210
2648	Chiroptera	Phyllostomidae	Glossophaga longirostris	LC	8.216	2.2210
2650	Rodentia	Muridae	Microtus oaxacensis	NT	3.594	2.2179
2651	Rodentia	Muridae	Rhizomys pruinosus	LC	8.186	2.2176
2651	Rodentia	Muridae	Rhizomys sinensis	LC	8.186	2.2176
2651	Rodentia	Muridae	Rhizomys sumatrensis	LC	8.186	2.2176
2654	Rodentia	Muridae	Reithrodontomys brevirostris	LC	8.182	2.2172
2654	Rodentia	Muridae	Reithrodontomys darienensis	LC	8.182	2.2172
2654	Rodentia	Muridae	Reithrodontomys gracilis	LC	8.182	2.2172
2654	Rodentia	Muridae	Reithrodontomys mexicanus	LC	8.182	2.2172
2658	Chiroptera	Vespertilionidae	Pipistrellus affinis	LC	8.169	2.2158
2658	Chiroptera	Vespertilionidae	Pipistrellus petersi	LC	8.169	2.2158
2660	Rodentia	Sciuridae	Tamias rufus	LC	8.161	2.2150
2661	Rodentia	Muridae	Bullimus bagobus	LC	8.158	2.2147
2661	Rodentia	Muridae	Bullimus luzonicus	LC	8.158	2.2147
2661	Rodentia	Muridae	Tarsomys apoensis	LC	8.158	2.2147
2664	Primates	Cebidae	Cacajao melanocephalus	LC	8.142	2.2129
2664	Primates	Cebidae	Chiropotes albinasus	LC	8.142	2.2129
2664	Primates	Cebidae	Chiropotes satanas	LC	8.142	2.2129
2667	Lagomorpha	Leporidae	Lepus europaeus	LC	8.142	2.2129
2668	Carnivora	Felidae	Felis silvestris	LC	8.140	2.2127

Rank	Order	Family	Species	IUCN	ED'	EDGE
2669	Rodentia	Sciuridae	Tamias townsendii	LC	8.124	2.2110
2670	Rodentia	Sciuridae	Spermophilus annulatus	LC	8.120	2.2105
2671	Carnivora	Phocidae	Halichoerus grypus	LC	8.110	2.2093
2672	Rodentia	Muridae	Mastomys angolensis	LC	8.103	2.2086
2672	Rodentia	Muridae	Mastomys coucha	LC	8.103	2.2086
2672	Rodentia	Muridae	Mastomys erythroleucus	LC	8.103	2.2086
2672	Rodentia	Muridae	Mastomys hildebrandtii	LC	8.103	2.2086
2672	Rodentia	Muridae	Mastomys natalensis	LC	8.103	2.2086
2672	Rodentia	Muridae	Mastomys shortridgei	LC	8.103	2.2086
2678	Primates	Cercopithecidae	Colobus angolensis	LC	8.099	2.2082
2679	Chiroptera	Phyllostomidae	Uroderma bilobatum	LC	8.094	2.2076
2679	Chiroptera	Phyllostomidae	Uroderma magnirostrum	LC	8.094	2.2076
2681	Insectivora	Soricidae	Sorex mirabilis	LC	8.089	2.2070
2681	Insectivora	Soricidae	Sorex raddei	LC	8.089	2.2070
2683	Dasyuromorphia	Dasyuridae	Antechinus bellus	LC	8.078	2.2059
2683	Dasyuromorphia	Dasyuridae	Antechinus flavipes	LC	8.078	2.2059
2685	Rodentia	Muridae	Mus crociduroides	LC	8.064	2.2043
2685	Rodentia	Muridae	Mus pahari	LC	8.064	2.2043
2687	Rodentia	Sciuridae	Tamiasciurus douglasii	LC	8.055	2.2033
2687	Rodentia	Sciuridae	Tamiasciurus hudsonicus	LC	8.055	2.2033
2689	Rodentia	Muridae	Volemys millicens	LC	8.054	2.2032
2689	Rodentia	Muridae	Volemys musseri	LC	8.054	2.2032
2691	Rodentia	Muridae	Oryzomys galapagoensis	VU	1.263	2.2028
2692	Rodentia	Muridae	Hybomys planifrons	LC	8.046	2.2023
2692	Rodentia	Muridae	Hybomys trivirgatus	LC	8.046	2.2023
2692	Rodentia	Muridae	Hybomys univittatus	LC	8.046	2.2023
2695	Primates	Cebidae	Callicebus caligatus	LC	8.043	2.2020
2695	Primates	Cebidae	Callicebus cupreus	LC	8.043	2.2020
2697	Rodentia	Muridae	Meriones persicus	LC	8.016	2.1990
2697	Rodentia	Muridae	Meriones rex	LC	8.016	2.1990
2699	Rodentia	Muridae	Malacomys cansdalei	LC	8.016	2.1990
2699	Rodentia	Muridae	Malacomys edwardsi	LC	8.016	2.1990
2699	Rodentia	Muridae	Malacomys longipes	LC	8.016	2.1990
2699	Rodentia	Muridae	Stenomys ceramicus	LC	8.016	2.1990
2699	Rodentia	Muridae	Stenomys niobe	LC	8.016	2.1990
2699	Rodentia	Muridae	Stenomys richardsoni	LC	8.016	2.1990
2699	Rodentia	Muridae	Stenomys verecundus	LC	8.016	2.1990
2706	Chiroptera	Vespertilionidae	Lasiurus cinereus	LC	8.011	2.1985
2707	Rodentia	Muridae	Margaretamys beccarii	LC	8.002	2.1974
2708	Lagomorpha	Leporidae	Lepus coreanus	LC	7.996	2.1968
2708	Lagomorpha	Leporidae	Lepus sinensis	LC	7.996	2.1968
2710	Carnivora	Viverridae	Genetta thierryi	LC	7.995	2.1967
2711	Rodentia	Muridae	Reithrodontomys creper	LC	7.966	2.1934
2711	Rodentia	Muridae	Reithrodontomys tenuirostris	LC	7.966	2.1934
2713	Rodentia	Muridae	Celaenomys silaceus	NT	3.482	2.1933
2714	Rodentia	Muridae	Leptomys ernstmayri	LC	7.958	2.1925
2715	Insectivora	Soricidae	Diplomesodon pulchellum	LC	7.957	2.1924
2715	Insectivora	Soricidae	Scutisorex somereni	LC	7.957	2.1924
2717	Primates	Cebidae	Ateles chamek	LC	7.948	2.1914
2718	Primates	Cebidae	Pithecia albicans	LC	7.943	2.1909
2718	Primates	Cebidae	Pithecia irrorata	LC	7.943	2.1909
2718	Primates	Cebidae	Pithecia monachus	LC	7.943	2.1909
2721	Primates	Callitrichidae	Saguinus nigricollis	LC	7.938	2.1903
2722	Chiroptera	Vespertilionidae	Lasiurus ega	LC	7.934	2.1899
2722	Chiroptera	Vespertilionidae	Lasiurus intermedius	LC	7.934	2.1899

Rank	Order	Family	Species	IUCN	ED'	EDGE
2724	Rodentia	Sciuridae	Sundasciurus davensis	LC	7.929	2.1893
2724	Rodentia	Sciuridae	Sundasciurus fraterculus	LC	7.929	2.1893
2724	Rodentia	Sciuridae	Sundasciurus hippurus	LC	7.929	2.1893
2724	Rodentia	Sciuridae	Sundasciurus hoogstraali	LC	7.929	2.1893
2724	Rodentia	Sciuridae	Sundasciurus lowii	LC	7.929	2.1893
2724	Rodentia	Sciuridae	Sundasciurus mindanensis	LC	7.929	2.1893
2724	Rodentia	Sciuridae	Sundasciurus philippinensis	LC	7.929	2.1893
2724	Rodentia	Sciuridae	Sundasciurus tenuis	LC	7.929	2.1893
2732	Chiroptera	Phyllostomidae	Artibeus cinereus	LC	7.914	2.1876
2733	Rodentia	Muridae	Microtus richardsoni	LC	7.910	2.1872
2733	Rodentia	Muridae	Microtus xanthognathus	LC	7.910	2.1872
2735	Rodentia	Sciuridae	Spermophilus lateralis	LC	7.901	2.1862
2735	Rodentia	Sciuridae	Spermophilus variegatus	LC	7.901	2.1862
2737	Chiroptera	Vespertilionidae	Pipistrellus coromandra	LC	7.890	2.1849
2737	Chiroptera	Vespertilionidae	Pipistrellus tenuis	LC	7.890	2.1849
2739	Insectivora	Soricidae	Myosorex cafer	LC	7.879	2.1837
2739	Insectivora	Soricidae	Myosorex varius	LC	7.879	2.1837
2741	Rodentia	Muridae	Tatera afra	LC	7.867	2.1823
2741	Rodentia	Muridae	Tatera brantsii	LC	7.867	2.1823
2741	Rodentia	Muridae	Tatera guineae	LC	7.867	2.1823
2741	Rodentia	Muridae	Tatera inclusa	LC	7.867	2.1823
2741	Rodentia	Muridae	Tatera kempi	LC	7.867	2.1823
2741	Rodentia	Muridae	Tatera leucogaster	LC	7.867	2.1823
2741	Rodentia	Muridae	Tatera nigricauda	LC	7.867	2.1823
2741	Rodentia	Muridae	Tatera phillipsi	LC	7.867	2.1823
2741	Rodentia	Muridae	Tatera robusta	LC	7.867	2.1823
2741	Rodentia	Muridae	Tatera valida	LC	7.867	2.1823
2751	Diprotodontia	Macropodidae	Onychogalea unguifera	LC	7.866	2.1823
2752	Chiroptera	Vespertilionidae	Pipistrellus nanus	LC	7.860	2.1815
2753	Cetacea	Delphinidae	Lissodelphis borealis	LC	7.855	2.1810
2754	Rodentia	Muridae	Grammomys cometes	LC	7.819	2.1769
2754	Rodentia	Muridae	Grammomys dolichurus	LC	7.819	2.1769
2754	Rodentia	Muridae	Grammomys ibeanus	LC	7.819	2.1769
2754	Rodentia	Muridae	Grammomys macmillani	LC	7.819	2.1769
2754	Rodentia	Muridae	Grammomys rutilans	LC	7.819	2.1769
2759	Rodentia	Muridae	Oenomys hypoxanthus	LC	7.815	2.1765
2760	Rodentia	Muridae	Chiropodomys calamianensis	LC	7.813	2.1762
2760	Rodentia	Muridae	Chiropodomys gliroides	LC	7.813	2.1762
2760	Rodentia	Muridae	Chiropodomys major	LC	7.813	2.1762
2760	Rodentia	Muridae	Chiropodomys pusillus	LC	7.813	2.1762
2764	Rodentia	Muridae	Apodemus agrarius	LC	7.804	2.1752
2764	Rodentia	Muridae	Apodemus chevrieri	LC	7.804	2.1752
2766	Rodentia	Muridae	Phyllotis bonaeriensis	NT	3.399	2.1746
2767	Insectivora	Soricidae	Sorex minutissimus	LC	7.790	2.1736
2768	Primates	Cebidae	Cebus olivaceus	LC	7.788	2.1734
2769	Insectivora	Soricidae	Sorex isodon	LC	7.782	2.1727
2769	Insectivora	Soricidae	Sorex unguiculatus	LC	7.782	2.1727
2771	Rodentia	Muridae	Ichthyomys stolzmanni	LC	7.765	2.1707
2771	Rodentia	Muridae	Ichthyomys tweedii	LC	7.765	2.1707
2771	Rodentia	Muridae	Rheomys raptor	LC	7.765	2.1707
2771	Rodentia	Muridae	Rheomys thomasi	LC	7.765	2.1707
2771	Rodentia	Muridae	Rheomys underwoodi	LC	7.765	2.1707
2776	Chiroptera	Pteropodidae	Dobsonia praedatrix	NT	3.381	2.1704
2777	Carnivora	Canidae	Nyctereutes procyonoides	LC	7.759	2.1701
2778	Rodentia	Muridae	Lasiopodomys brandtii	LC	7.749	2.1689
2778	Rodentia	Muridae	Lasiopodomys mandarinus	LC	7.749	2.1689

Rank	Order	Family	Species	IUCN	ED'	EDGE
2780	Artiodactyla	Bovidae	Procapra gutturosa	LC	7.747	2.1688
2780	Artiodactyla	Bovidae	Procapra picticaudata	LC	7.747	2.1688
2782	Chiroptera	Vespertilionidae	Pipistrellus circumdatus	LC	7.744	2.1683
2782	Chiroptera	Vespertilionidae	Pipistrellus crassulus	LC	7.744	2.1683
2782	Chiroptera	Vespertilionidae	Pipistrellus nanulus	LC	7.744	2.1683
2782	Chiroptera	Vespertilionidae	Pipistrellus rueppelli	LC	7.744	2.1683
2782	Chiroptera	Vespertilionidae	Pipistrellus stenopterus	LC	7.744	2.1683
2787	Carnivora	Otariidae	Neophoca cinerea	LC	7.744	2.1683
2788	Rodentia	Sciuridae	Sciurus arizonensis	NT	3.371	2.1682
2789	Carnivora	Felidae	Lynx canadensis	LC	7.729	2.1667
2790	Rodentia	Muridae	Eolagurus przewalskii	LC	7.726	2.1663
2791	Cetacea	Delphinidae	Lagenorhynchus obliquidens	LC	7.723	2.1659
2792	Rodentia	Muridae	Neusticomys monticolus	LC	7.721	2.1658
2793	Rodentia	Muridae	Arvicola terrestris	LC	7.708	2.1643
2793	Rodentia	Muridae	Blanfordimys afghanus	LC	7.708	2.1643
2795	Primates	Cercopithecidae	Chlorocebus aethiops	LC	7.693	2.1625
2796	Primates	Cebidae	Cebus albifrons	LC	7.684	2.1615
2796	Primates	Cebidae	Cebus capucinus	LC	7.684	2.1615
2798	Chiroptera	Rhinolophidae	Hipposideros armiger	LC	7.672	2.1601
2798	Chiroptera	Rhinolophidae	Hipposideros commersoni	LC	7.672	2.1601
2798	Chiroptera	Rhinolophidae	Hipposideros diadema	LC	7.672	2.1601
2798	Chiroptera	Rhinolophidae	Hipposideros lankadiva	LC	7.672	2.1601
2798	Chiroptera	Rhinolophidae	Hipposideros larvatus	LC	7.672	2.1601
2803	Chiroptera	Pteropodidae	Ptenochirus jagori	LC	7.654	2.1581
2803	Chiroptera	Pteropodidae	Ptenochirus minor	LC	7.654	2.1581
2805	Chiroptera	Pteropodidae	Megaerops ecaudatus	LC	7.648	2.1573
2805	Chiroptera	Pteropodidae	Megaerops niphanae	LC	7.648	2.1573
2805	Chiroptera	Pteropodidae	Megaerops wetmorei	LC	7.648	2.1573
2808	Rodentia	Muridae	Delomys dorsalis	LC	7.636	2.1559
2808	Rodentia	Muridae	Delomys sublineatus	LC	7.636	2.1559
2810	Primates	Cebidae	Alouatta sara	LC	7.633	2.1556
2810	Primates	Cebidae	Alouatta seniculus	LC	7.633	2.1556
2812	Chiroptera	Pteropodidae	Cynopterus horsfieldi	LC	7.608	2.1526
2812	Chiroptera	Pteropodidae	Cynopterus titthaecheilus	LC	7.608	2.1526
2814	Rodentia	Heteromyidae	Dipodomys compactus	LC	7.603	2.1521
2815	Chiroptera	Pteropodidae	Dobsonia exoleta	NT	3.301	2.1519
2815	Chiroptera	Pteropodidae	Dobsonia minor	NT	3.301	2.1519
2817	Dasyuromorphia	Dasyuridae	Sarcophilus laniarius	LC	7.600	2.1518
2818	Rodentia	Muridae	Microtus guentheri	NT	3.300	2.1517
2819	Primates	Cebidae	Callicebus donacophilus	LC	7.578	2.1492
2820	Chiroptera	Phyllostomidae	Vampyressa nymphaea	LC	7.568	2.1481
2821	Chiroptera	Phyllostomidae	Artibeus anderseni	LC	7.564	2.1475
2821	Chiroptera	Phyllostomidae	Artibeus glaucus	LC	7.564	2.1475
2823	Lagomorpha	Leporidae	Sylvilagus brasiliensis	LC	7.557	2.1468
2824	Chiroptera	Vespertilionidae	Kerivoula atrox	LC	7.543	2.1451
2824	Chiroptera	Vespertilionidae	Kerivoula jagorii	LC	7.543	2.1451
2824	Chiroptera	Vespertilionidae	Kerivoula papuensis	LC	7.543	2.1451
2827	Rodentia	Muridae	Eliurus minor	LC	7.541	2.1449
2827	Rodentia	Muridae	Eliurus myoxinus	LC	7.541	2.1449
2827	Rodentia	Muridae	Eliurus tanala	LC	7.541	2.1449
2830	Rodentia	Muridae	Berylmys berdmorei	LC	7.537	2.1444
2830	Rodentia	Muridae	Berylmys bowersi	LC	7.537	2.1444
2830	Rodentia	Muridae	Berylmys mackenziei	LC	7.537	2.1444
2830	Rodentia	Muridae	Berylmys manipulus	LC	7.537	2.1444
2830	Rodentia	Muridae	Leopoldamys edwardsi	LC	7.537	2.1444
2830	Rodentia	Muridae	Leopoldamys sabanus	LC	7.537	2.1444

Rank	Order	Family	Species	IUCN	ED'	EDGE
2830	Rodentia	Muridae	Pogonomys loriae	LC	7.537	2.1444
2830	Rodentia	Muridae	Pogonomys macrourus	LC	7.537	2.1444
2830	Rodentia	Muridae	Pogonomys sylvestris	LC	7.537	2.1444
2839	Chiroptera	Phyllostomidae	Lonchophylla mordax	LC	7.509	2.1411
2839	Chiroptera	Phyllostomidae	Lonchophylla robusta	LC	7.509	2.1411
2839	Chiroptera	Phyllostomidae	Lonchophylla thomasi	LC	7.509	2.1411
2842	Rodentia	Muridae	Phaulomys smithii	LC	7.477	2.1373
2843	Rodentia	Muridae	Praomys jacksoni	LC	7.473	2.1369
2843	Rodentia	Muridae	Praomys misonnei	LC	7.473	2.1369
2843	Rodentia	Muridae	Praomys rostratus	LC	7.473	2.1369
2843	Rodentia	Muridae	Praomys tullbergi	LC	7.473	2.1369
2847	Rodentia	Muridae	Diplothrix legatus	EN	0.058	2.1360
2847	Rodentia	Muridae	Muriculus imberbis	EN	0.058	2.1360
2847	Rodentia	Muridae	Palawanomys furvus	EN	0.058	2.1360
2850	Rodentia	Muridae	Arvicanthis abyssinicus	LC	7.446	2.1337
2850	Rodentia	Muridae	Arvicanthis nairobae	LC	7.446	2.1337
2850	Rodentia	Muridae	Arvicanthis niloticus	LC	7.446	2.1337
2850	Rodentia	Muridae	Arvicanthis somalicus	LC	7.446	2.1337
2854	Lagomorpha	Leporidae	Sylvilagus aquaticus	LC	7.443	2.1334
2854	Lagomorpha	Leporidae	Sylvilagus palustris	LC	7.443	2.1334
2856	Rodentia	Muridae	Niviventer andersoni	LC	7.422	2.1309
2856	Rodentia	Muridae	Niviventer brahma	LC	7.422	2.1309
2856	Rodentia	Muridae	Niviventer confucianus	LC	7.422	2.1309
2856	Rodentia	Muridae	Niviventer cremoriventer	LC	7.422	2.1309
2856	Rodentia	Muridae	Niviventer eha	LC	7.422	2.1309
2856	Rodentia	Muridae	Niviventer excelsior	LC	7.422	2.1309
2856	Rodentia	Muridae	Niviventer fulvescens	LC	7.422	2.1309
2856	Rodentia	Muridae	Niviventer langbianis	LC	7.422	2.1309
2856	Rodentia	Muridae	Niviventer lepturus	LC	7.422	2.1309
2856	Rodentia	Muridae	Niviventer niviventer	LC	7.422	2.1309
2856	Rodentia	Muridae	Niviventer rapit	LC	7.422	2.1309
2856	Rodentia	Muridae	Niviventer tenaster	LC	7.422	2.1309
2868	Chiroptera	Phyllostomidae	Lichonycteris obscura	LC	7.405	2.1288
2869	Rodentia	Muridae	Apodemus argenteus	LC	7.363	2.1238
2869	Rodentia	Muridae	Apodemus draco	LC	7.363	2.1238
2869	Rodentia	Muridae	Apodemus gurkha	LC	7.363	2.1238
2869	Rodentia	Muridae	Apodemus latronum	LC	7.363	2.1238
2869	Rodentia	Muridae	Apodemus peninsulae	LC	7.363	2.1238
2869	Rodentia	Muridae	Apodemus speciosus	LC	7.363	2.1238
2875	Chiroptera	Vespertilionidae	Myotis myotis	NT	3.180	2.1235
2876	Lagomorpha	Leporidae	Lepus townsendii	LC	7.344	2.1215
2877	Rodentia	Heteromyidae	Dipodomys deserti	LC	7.335	2.1204
2878	Rodentia	Caviidae	Galea flavidens	LC	7.313	2.1178
2878	Rodentia	Caviidae	Galea musteloides	LC	7.313	2.1178
2878	Rodentia	Caviidae	Galea spixii	LC	7.313	2.1178
2881	Insectivora	Soricidae	Sorex caecutiens	LC	7.286	2.1145
2881	Insectivora	Soricidae	Sorex shinto	LC	7.286	2.1145
2883	Rodentia	Sciuridae	Tamias senex	LC	7.274	2.1131
2883	Rodentia	Sciuridae	Tamias sonomae	LC	7.274	2.1131
2885	Rodentia	Muridae	Reithrodontomys burti	LC	7.270	2.1126
2885	Rodentia	Muridae	Reithrodontomys chrysopsis	LC	7.270	2.1126
2885	Rodentia	Muridae	Reithrodontomys humulis	LC	7.270	2.1126
2885	Rodentia	Muridae	Reithrodontomys megalotis	LC	7.270	2.1126
2885	Rodentia	Muridae	Reithrodontomys montanus	LC	7.270	2.1126
2885	Rodentia	Muridae	Reithrodontomys sumichrasti	LC	7.270	2.1126
2891	Rodentia	Echimyidae	Diplomys labilis	LC	7.260	2.1114

Rank	Order	Family	Species	IUCN	ED'	EDGE
2892	Chiroptera	Rhinolophidae	Rhinolophus mitratus	VU	1.062	2.1102
2892	Chiroptera	Rhinolophidae	Rhinolophus paradoxolophus	VU	1.062	2.1102
2892	Chiroptera	Rhinolophidae	Rhinolophus rex	VU	1.062	2.1102
2892	Chiroptera	Rhinolophidae	Rhinolophus silvestris	VU	1.062	2.1102
2892	Chiroptera	Rhinolophidae	Rhinolophus subrufus	VU	1.062	2.1102
2897	Chiroptera	Vespertilionidae	Kerivoula cuprosa	NT	3.120	2.1091
2897	Chiroptera	Vespertilionidae	Kerivoula intermedia	NT	3.120	2.1091
2897	Chiroptera	Vespertilionidae	Kerivoula minuta	NT	3.120	2.1091
2897	Chiroptera	Vespertilionidae	Kerivoula phalaena	NT	3.120	2.1091
2897	Chiroptera	Vespertilionidae	Kerivoula smithii	NT	3.120	2.1091
2902	Insectivora	Soricidae	Sorex buchariensis	LC	7.231	2.1079
2902	Insectivora	Soricidae	Sorex minutus	LC	7.231	2.1079
2902	Insectivora	Soricidae	Sorex volnuchini	LC	7.231	2.1079
2905	Insectivora	Soricidae	Sorex arcticus	LC	7.212	2.1056
2906	Chiroptera	Pteropodidae	Epomophorus angolensis	NT	3.105	2.1054
2907	Primates	Cebidae	Saimiri boliviensis	LC	7.204	2.1047
2908	Rodentia	Muridae	Alticola lemminus	LC	7.186	2.1024
2908	Rodentia	Muridae	Alticola strelzowi	LC	7.186	2.1024
2910	Rodentia	Muridae	Tachyoryctes ankoliae	LC	7.184	2.1022
2910	Rodentia	Muridae	Tachyoryctes audax	LC	7.184	2.1022
2910	Rodentia	Muridae	Tachyoryctes daemon	LC	7.184	2.1022
2910	Rodentia	Muridae	Tachyoryctes naivashae	LC	7.184	2.1022
2910	Rodentia	Muridae	Tachyoryctes rex	LC	7.184	2.1022
2910	Rodentia	Muridae	Tachyoryctes ruandae	LC	7.184	2.1022
2910	Rodentia	Muridae	Tachyoryctes ruddi	LC	7.184	2.1022
2910	Rodentia	Muridae	Tachyoryctes spalacinus	LC	7.184	2.1022
2910	Rodentia	Muridae	Tachyoryctes splendens	LC	7.184	2.1022
2919	Rodentia	Sciuridae	Paraxerus alexandri	LC	7.180	2.1017
2919	Rodentia	Sciuridae	Paraxerus boehmi	LC	7.180	2.1017
2919	Rodentia	Sciuridae	Paraxerus cepapi	LC	7.180	2.1017
2919	Rodentia	Sciuridae	Paraxerus ochraceus	LC	7.180	2.1017
2919	Rodentia	Sciuridae	Paraxerus palliatus	LC	7.180	2.1017
2919	Rodentia	Sciuridae	Paraxerus poensis	LC	7.180	2.1017
2925	Diprotodontia	Macropodidae	Thylogale stigmatica	LC	7.178	2.1014
2925	Diprotodontia	Macropodidae	Thylogale thetis	LC	7.178	2.1014
2927	Dasyuromorphia	Dasyuridae	Neophascogale lorentzi	LC	7.178	2.1014
2928	Chiroptera	Vespertilionidae	Myotis chiloensis	NT	3.083	2.0999
2928	Chiroptera	Vespertilionidae	Myotis fortidens	NT	3.083	2.0999
2928	Chiroptera	Vespertilionidae	Myotis montivagus	NT	3.083	2.0999
2928	Chiroptera	Vespertilionidae	Myotis ricketti	NT	3.083	2.0999
2932	Cetacea	Delphinidae	Lagenorhynchus acutus	LC	7.143	2.0972
2932	Cetacea	Delphinidae	Lagenorhynchus albirostris	LC	7.143	2.0972
2934	Rodentia	Muridae	Myospalax aspalax	LC	7.133	2.0960
2934	Rodentia	Muridae	Myospalax myospalax	LC	7.133	2.0960
2934	Rodentia	Muridae	Myospalax psilurus	LC	7.133	2.0960
2937	Primates	Callitrichidae	Saguinus imperator	LC	7.123	2.0946
2938	Chiroptera	Vespertilionidae	Myotis siligorensis	LC	7.110	2.0931
2939	Chiroptera	Vespertilionidae	Myotis formosus	LC	7.107	2.0927
2939	Chiroptera	Vespertilionidae	Myotis welwitschii	LC	7.107	2.0927
2941	Rodentia	Muridae	Calomyscus bailwardi	LC	7.101	2.0920
2941	Rodentia	Muridae	Calomyscus baluchi	LC	7.101	2.0920
2943	Rodentia	Muridae	Lemmiscus curtatus	LC	7.087	2.0903
2943	Rodentia	Muridae	Ondatra zibethicus	LC	7.087	2.0903
2945	Carnivora	Otariidae	Arctocephalus gazella	LC	7.077	2.0890
2945	Carnivora	Otariidae	Arctocephalus tropicalis	LC	7.077	2.0890

Rank	Order	Family	Species	IUCN	ED'	EDGE
2947	Rodentia	Muridae	Microtus gregalis	LC	7.059	2.0868
2947	Rodentia	Muridae	Microtus miurus	LC	7.059	2.0868
2949	Rodentia	Sciuridae	Rheithrosciurus macrotis	LC	7.048	2.0855
2950	Rodentia	Muridae	Maxomys bartelsii	LC	7.048	2.0854
2950	Rodentia	Muridae	Maxomys hellwaldii	LC	7.048	2.0854
2950	Rodentia	Muridae	Maxomys inas	LC	7.048	2.0854
2950	Rodentia	Muridae	Maxomys inflatus	LC	7.048	2.0854
2950	Rodentia	Muridae	Maxomys moi	LC	7.048	2.0854
2950	Rodentia	Muridae	Maxomys musschenbroekii	LC	7.048	2.0854
2950	Rodentia	Muridae	Maxomys ochraceiventer	LC	7.048	2.0854
2950	Rodentia	Muridae	Maxomys rajah	LC	7.048	2.0854
2950	Rodentia	Muridae	Maxomys surifer	LC	7.048	2.0854
2950	Rodentia	Muridae	Maxomys whiteheadi	LC	7.048	2.0854
2960	Chiroptera	Rhinolophidae	Aselliscus stoliczkanus	LC	6.951	2.0733
2960	Chiroptera	Rhinolophidae	Hipposideros calcaratus	LC	6.951	2.0733
2960	Chiroptera	Rhinolophidae	Hipposideros cervinus	LC	6.951	2.0733
2960	Chiroptera	Rhinolophidae	Hipposideros ruber	LC	6.951	2.0733
2960	Chiroptera	Rhinolophidae	Hipposideros sabanus	LC	6.951	2.0733
2965	Rodentia	Sciuridae	Hylopetes spadiceus	LC	6.932	2.0709
2966	Carnivora	Felidae	Felis chaus	LC	6.906	2.0676
2967	Chiroptera	Vespertilionidae	Pipistrellus javanicus	LC	6.905	2.0675
2968	Primates	Callitrichidae	Saguinus bicolor	LC	6.867	2.0627
2968	Primates	Callitrichidae	Saguinus midas	LC	6.867	2.0627
2970	Rodentia	Muridae	Microtus canicaudus	LC	6.855	2.0612
2970	Rodentia	Muridae	Microtus montanus	LC	6.855	2.0612
2972	Dasyuromorphia	Dasyuridae	Pseudantechinus macdonnellensis	LC	6.841	2.0593
2972	Dasyuromorphia	Dasyuridae	Pseudantechinus woolleyae	LC	6.841	2.0593
2974	Rodentia	Sciuridae	Spermophilus canus	LC	6.830	2.0580
2975	Rodentia	Heteromyidae	Chaetodipus artus	LC	6.803	2.0546
2975	Rodentia	Heteromyidae	Chaetodipus goldmani	LC	6.803	2.0546
2977	Chiroptera	Vespertilionidae	Eptesicus baverstocki	LC	6.798	2.0539
2977	Chiroptera	Vespertilionidae	Eptesicus pumilus	LC	6.798	2.0539
2977	Chiroptera	Vespertilionidae	Eptesicus regulus	LC	6.798	2.0539
2977	Chiroptera	Vespertilionidae	Eptesicus sagittula	LC	6.798	2.0539
2977	Chiroptera	Vespertilionidae	Eptesicus vulturnus	LC	6.798	2.0539
2982	Carnivora	Canidae	Urocyon cinereoargenteus	LC	6.779	2.0514
2983	Rodentia	Muridae	Mus spicilegus	NT	2.883	2.0498
2984	Artiodactyla	Bovidae	Ovis dalli	LC	6.742	2.0466
2985	Chiroptera	Pteropodidae	Nyctimene albiventer	LC	6.729	2.0450
2986	Primates	Callitrichidae	Saguinus fuscicollis	LC	6.725	2.0444
2986	Primates	Callitrichidae	Saguinus tripartitus	LC	6.725	2.0444
2988	Chiroptera	Phyllostomidae	Artibeus aztecus	LC	6.723	2.0442
2988	Chiroptera	Phyllostomidae	Artibeus phaeotis	LC	6.723	2.0442
2988	Chiroptera	Phyllostomidae	Artibeus toltecus	LC	6.723	2.0442
2991	Rodentia	Sciuridae	Sciurus deppei	LC	6.708	2.0423
2992	Chiroptera	Phyllostomidae	Chiroderma salvini	LC	6.698	2.0410
2993	Chiroptera	Vespertilionidae	Glischropus tylopus	LC	6.697	2.0408
2993	Chiroptera	Vespertilionidae	Pipistrellus eisentrauti	LC	6.697	2.0408
2993	Chiroptera	Vespertilionidae	Pipistrellus hesperus	LC	6.697	2.0408
2993	Chiroptera	Vespertilionidae	Tylonycteris pachypus	LC	6.697	2.0408
2993	Chiroptera	Vespertilionidae	Tylonycteris robustula	LC	6.697	2.0408
2998	Rodentia	Sciuridae	Petaurillus emiliae	LC	6.694	2.0404
2998	Rodentia	Sciuridae	Petaurillus hosei	LC	6.694	2.0404
2998	Rodentia	Sciuridae	Petaurillus kinlochii	LC	6.694	2.0404

Rank	Order	Family	Species	IUCN	ED'	EDGE
3001	Artiodactyla	Cervidae	Muntiacus atherodes	LC	6.646	2.0341
3001	Artiodactyla	Cervidae	Muntiacus muntjak	LC	6.646	2.0341
3001	Artiodactyla	Cervidae	Muntiacus reevesi	LC	6.646	2.0341
3004	Rodentia	Muridae	Bandicota bengalensis	LC	6.639	2.0333
3004	Rodentia	Muridae	Bandicota indica	LC	6.639	2.0333
3004	Rodentia	Muridae	Bandicota savilei	LC	6.639	2.0333
3004	Rodentia	Muridae	Sundamys infraluteus	LC	6.639	2.0333
3004	Rodentia	Muridae	Sundamys muelleri	LC	6.639	2.0333
3009	Rodentia	Sciuridae	Spermophilus tereticaudus	LC	6.637	2.0330
3010	Rodentia	Muridae	Lemniscomys barbarus	LC	6.631	2.0323
3010	Rodentia	Muridae	Lemniscomys bellieri	LC	6.631	2.0323
3010	Rodentia	Muridae	Lemniscomys griselda	LC	6.631	2.0323
3010	Rodentia	Muridae	Lemniscomys macculus	LC	6.631	2.0323
3010	Rodentia	Muridae	Lemniscomys rosalia	LC	6.631	2.0323
3010	Rodentia	Muridae	Lemniscomys striatus	LC	6.631	2.0323
3016	Chiroptera	Phyllostomidae	Ametrida centurio	LC	6.599	2.0281
3016	Chiroptera	Phyllostomidae	Sphaeronycteris toxophyllum	LC	6.599	2.0281
3018	Primates	Cercopithecidae	Colobus guereza	LC	6.598	2.0280
3019	Rodentia	Sciuridae	Sciurus nayaritensis	LC	6.581	2.0257
3020	Chiroptera	Pteropodidae	Rousettus madagascariensis	NT	2.773	2.0209
3020	Chiroptera	Pteropodidae	Rousettus obliviosus	NT	2.773	2.0209
3022	Rodentia	Dasyproctidae	Dasyprocta fuliginosa	LC	6.529	2.0187
3022	Rodentia	Dasyproctidae	Dasyprocta guamara	LC	6.529	2.0187
3022	Rodentia	Dasyproctidae	Dasyprocta leporina	LC	6.529	2.0187
3022	Rodentia	Dasyproctidae	Dasyprocta prymnolopha	LC	6.529	2.0187
3022	Rodentia	Dasyproctidae	Dasyprocta punctata	LC	6.529	2.0187
3027	Artiodactyla	Bovidae	Raphicerus campestris	LC	6.515	2.0169
3028	Rodentia	Sciuridae	Petinomys crinitus	LC	6.471	2.0110
3028	Rodentia	Sciuridae	Petinomys genibarbis	LC	6.471	2.0110
3028	Rodentia	Sciuridae	Petinomys hageni	LC	6.471	2.0110
3028	Rodentia	Sciuridae	Petinomys sagitta	LC	6.471	2.0110
3028	Rodentia	Sciuridae	Petinomys vordermanni	LC	6.471	2.0110
3033	Carnivora	Phocidae	Phoca hispida	LC	6.456	2.0091
3034	Rodentia	Muridae	Phodopus campbelli	LC	6.434	2.0060
3034	Rodentia	Muridae	Phodopus roborovskii	LC	6.434	2.0060
3034	Rodentia	Muridae	Phodopus sungorus	LC	6.434	2.0060
3037	Chiroptera	Phyllostomidae	Choeroniscus minor	LC	6.411	2.0030
3038	Carnivora	Phocidae	Phoca largha	LC	6.407	2.0025
3038	Carnivora	Phocidae	Phoca vitulina	LC	6.407	2.0025
3040	Rodentia	Heteromyidae	Dipodomys ordii	LC	6.385	1.9995
3041	Rodentia	Geomyidae	Pappogeomys bulleri	LC	6.383	1.9991
3041	Rodentia	Geomyidae	Pappogeomys castanops	LC	6.383	1.9991
3041	Rodentia	Geomyidae	Pappogeomys gymnurus	LC	6.383	1.9991
3041	Rodentia	Geomyidae	Pappogeomys merriami	LC	6.383	1.9991
3045	Primates	Cebidae	Callicebus brunneus	LC	6.336	1.9928
3045	Primates	Cebidae	Callicebus cinerascens	LC	6.336	1.9928
3045	Primates	Cebidae	Callicebus hoffmannsi	LC	6.336	1.9928
3045	Primates	Cebidae	Callicebus moloch	LC	6.336	1.9928
3049	Chiroptera	Pteropodidae	Acerodon mackloti	LC	6.329	1.9918
3050	Carnivora	Mustelidae	Mustela putorius	LC	6.297	1.9875
3051	Rodentia	Sciuridae	Hylopetes phayrei	LC	6.294	1.9871
3052	Chiroptera	Vespertilionidae	Myotis adversus	LC	6.294	1.9871
3052	Chiroptera	Vespertilionidae	Myotis bocagei	LC	6.294	1.9871
3052	Chiroptera	Vespertilionidae	Myotis hasseltii	LC	6.294	1.9871
3052	Chiroptera	Vespertilionidae	Myotis horsfieldii	LC	6.294	1.9871
3056	Chiroptera	Phyllostomidae	Centurio senex	LC	6.294	1.9870

Rank	Order	Family	Species	IUCN	ED'	EDGE
3057	Lagomorpha	Leporidae	Lepus arcticus	LC	6.265	1.9831
3058	Rodentia	Heteromyidae	Liomys irroratus	LC	6.253	1.9814
3059	Primates	Callitrichidae	Saguinus labiatus	LC	6.223	1.9773
3059	Primates	Callitrichidae	Saguinus mystax	LC	6.223	1.9773
3061	Carnivora	Mustelidae	Lutra maculicollis	LC	6.220	1.9769
3062	Chiroptera	Vespertilionidae	Myotis riparius	LC	6.214	1.9760
3063	Chiroptera	Vespertilionidae	Myotis nattereri	LC	6.201	1.9742
3063	Chiroptera	Vespertilionidae	Myotis thysanodes	LC	6.201	1.9742
3065	Rodentia	Muridae	Graomys domorum	LC	6.195	1.9734
3065	Rodentia	Muridae	Graomys edithae	LC	6.195	1.9734
3065	Rodentia	Muridae	Graomys griseoflavus	LC	6.195	1.9734
3068	Primates	Cebidae	Alouatta belzebul	LC	6.186	1.9721
3068	Primates	Cebidae	Alouatta caraya	LC	6.186	1.9721
3070	Primates	Cercopithecidae	Cercopithecus campbelli	LC	6.185	1.9719
3070	Primates	Cercopithecidae	Cercopithecus mona	LC	6.185	1.9719
3072	Lagomorpha	Leporidae	Lepus alleni	LC	6.168	1.9696
3073	Lagomorpha	Leporidae	Lepus othus	LC	6.165	1.9692
3073	Lagomorpha	Leporidae	Lepus timidus	LC	6.165	1.9692
3075	Rodentia	Muridae	Auliscomys boliviensis	LC	6.159	1.9684
3075	Rodentia	Muridae	Auliscomys micropus	LC	6.159	1.9684
3075	Rodentia	Muridae	Auliscomys pictus	LC	6.159	1.9684
3075	Rodentia	Muridae	Auliscomys sublimis	LC	6.159	1.9684
3079	Rodentia	Sciuridae	Sciurus pucheranii	LC	6.156	1.9680
3080	Rodentia	Heteromyidae	Dipodomys nelsoni	LC	6.141	1.9659
3080	Rodentia	Heteromyidae	Dipodomys spectabilis	LC	6.141	1.9659
3082	Rodentia	Sciuridae	Petinomys fuscocapillus	LC	6.118	1.9626
3082	Rodentia	Sciuridae	Petinomys setosus	LC	6.118	1.9626
3084	Chiroptera	Vespertilionidae	Lasiurus borealis	LC	6.102	1.9604
3084	Chiroptera	Vespertilionidae	Lasiurus seminolus	LC	6.102	1.9604
3086	Rodentia	Sciuridae	Tamias cinereicollis	LC	6.101	1.9603
3087	Rodentia	Heteromyidae	Heteromys desmarestianus	LC	6.090	1.9587
3088	Chiroptera	Vespertilionidae	Myotis altarium	LC	6.087	1.9582
3089	Artiodactyla	Cervidae	Odocoileus hemionus	LC	6.085	1.9580
3089	Artiodactyla	Cervidae	Odocoileus virginianus	LC	6.085	1.9580
3091	Primates	Callitrichidae	Callithrix pygmaea	LC	6.074	1.9565
3092	Diprotodontia	Macropodidae	Petrogale brachyotis	LC	6.065	1.9552
3093	Chiroptera	Pteropodidae	Nyctimene cephalotes	LC	6.064	1.9551
3093	Chiroptera	Pteropodidae	Nyctimene vizcaccia	LC	6.064	1.9551
3095	Dasyuromorphia	Dasyuridae	Phascolosorex dorsalis	LC	6.064	1.9550
3096	Rodentia	Muridae	Rattus marmosurus	LC	6.060	1.9545
3096	Rodentia	Muridae	Rattus xanthurus	LC	6.060	1.9545
3098	Chiroptera	Vespertilionidae	Eptesicus bobrinskoi	LC	6.029	1.9501
3098	Chiroptera	Vespertilionidae	Eptesicus nilssoni	LC	6.029	1.9501
3100	Lagomorpha	Leporidae	Lepus californicus	LC	6.026	1.9496
3101	Rodentia	Muridae	Microtus juldaschi	LC	6.013	1.9478
3101	Rodentia	Muridae	Microtus leucurus	LC	6.013	1.9478
3103	Primates	Cebidae	Saimiri sciureus	LC	5.950	1.9387
3103	Primates	Cebidae	Saimiri ustus	LC	5.950	1.9387
3105	Rodentia	Sciuridae	Tamias dorsalis	LC	5.939	1.9372
3105	Rodentia	Sciuridae	Tamias quadrivittatus	LC	5.939	1.9372
3107	Rodentia	Muridae	Peromyscus caniceps	VU	0.732	1.9356
3107	Rodentia	Muridae	Peromyscus madrensis	VU	0.732	1.9356
3107	Rodentia	Muridae	Peromyscus mekisturus	VU	0.732	1.9356
3107	Rodentia	Muridae	Peromyscus melanurus	VU	0.732	1.9356
3107	Rodentia	Muridae	Peromyscus polius	VU	0.732	1.9356
3107	Rodentia	Muridae	Peromyscus zarhynchus	VU	0.732	1.9356

Rank	Order	Family	Species	IUCN	ED'	EDGE
3113	Carnivora	Mustelidae	Aonyx capensis	LC	5.921	1.9346
3114	Rodentia	Heteromyidae	Liomys salvini	LC	5.879	1.9285
3115	Rodentia	Sciuridae	Tamias umbrinus	LC	5.868	1.9269
3116	Primates	Cercopithecidae	Cercopithecus ascanius	LC	5.853	1.9247
3116	Primates	Cercopithecidae	Cercopithecus cephus	LC	5.853	1.9247
3116	Primates	Cercopithecidae	Cercopithecus petaurista	LC	5.853	1.9247
3119	Rodentia	Muridae	Andalgalomys pearsoni	LC	5.824	1.9204
3120	Rodentia	Muridae	Phenacomys intermedius	LC	5.818	1.9195
3120	Rodentia	Muridae	Phenacomys ungava	LC	5.818	1.9195
3122	Carnivora	Canidae	Vulpes bengalensis	LC	5.800	1.9170
3122	Carnivora	Canidae	Vulpes chama	LC	5.800	1.9170
3124	Rodentia	Sciuridae	Sciurus gilvigularis	LC	5.766	1.9119
3125	Rodentia	Heteromyidae	Dipodomys microps	LC	5.715	1.9043
3126	Primates	Cercopithecidae	Cercopithecus pogonias	LC	5.712	1.9038
3127	Rodentia	Muridae	Microtus californicus	LC	5.688	1.9003
3127	Rodentia	Muridae	Microtus oregoni	LC	5.688	1.9003
3129	Rodentia	Sciuridae	Spermophilus alashanicus	LC	5.678	1.8988
3130	Rodentia	Muridae	Meriones crassus	LC	5.644	1.8937
3130	Rodentia	Muridae	Meriones libycus	LC	5.644	1.8937
3130	Rodentia	Muridae	Meriones meridianus	LC	5.644	1.8937
3130	Rodentia	Muridae	Meriones shawi	LC	5.644	1.8937
3130	Rodentia	Muridae	Meriones tristrami	LC	5.644	1.8937
3130	Rodentia	Muridae	Meriones unguiculatus	LC	5.644	1.8937
3130	Rodentia	Muridae	Meriones vinogradovi	LC	5.644	1.8937
3137	Rodentia	Sciuridae	Sciurus yucatanensis	LC	5.631	1.8918
3138	Chiroptera	Pteropodidae	Rousettus lanosus	LC	5.630	1.8917
3139	Rodentia	Muridae	Otomys anchietae	LC	5.625	1.8908
3139	Rodentia	Muridae	Otomys angoniensis	LC	5.625	1.8908
3139	Rodentia	Muridae	Otomys irroratus	LC	5.625	1.8908
3139	Rodentia	Muridae	Otomys laminatus	LC	5.625	1.8908
3139	Rodentia	Muridae	Otomys maximus	LC	5.625	1.8908
3139	Rodentia	Muridae	Otomys saundersiae	LC	5.625	1.8908
3139	Rodentia	Muridae	Otomys sloggetti	LC	5.625	1.8908
3139	Rodentia	Muridae	Otomys tropicalis	LC	5.625	1.8908
3139	Rodentia	Muridae	Otomys typus	LC	5.625	1.8908
3139	Rodentia	Muridae	Otomys unisulcatus	LC	5.625	1.8908
3149	Rodentia	Sciuridae	Microsciurus alfari	LC	5.624	1.8907
3150	Rodentia	Heteromyidae	Liomys pictus	LC	5.598	1.8868
3151	Insectivora	Soricidae	Paracrocidura schoutedeni	LC	5.593	1.8860
3152	Chiroptera	Vespertilionidae	Murina aurata	NT	2.286	1.8827
3152	Chiroptera	Vespertilionidae	Murina silvatica	NT	2.286	1.8827
3154	Chiroptera	Phyllostomidae	Sturnira bogotensis	LC	5.570	1.8825
3154	Chiroptera	Phyllostomidae	Sturnira erythromos	LC	5.570	1.8825
3154	Chiroptera	Phyllostomidae	Sturnira ludovici	LC	5.570	1.8825
3154	Chiroptera	Phyllostomidae	Sturnira luisi	LC	5.570	1.8825
3154	Chiroptera	Phyllostomidae	Sturnira tildae	LC	5.570	1.8825
3159	Rodentia	Muridae	Microtus limnophilus	LC	5.564	1.8816
3159	Rodentia	Muridae	Microtus montebelli	LC	5.564	1.8816
3159	Rodentia	Muridae	Microtus oeconomus	LC	5.564	1.8816
3162	Chiroptera	Vespertilionidae	Eudiscopus denticulus	NT	2.280	1.8810
3162	Chiroptera	Vespertilionidae	Ia io	NT	2.280	1.8810
3162	Chiroptera	Vespertilionidae	Nycticeius rueppellii	NT	2.280	1.8810
3165	Rodentia	Muridae	Microtus pinetorum	LC	5.544	1.8785
3165	Rodentia	Muridae	Microtus quasiater	LC	5.544	1.8785
3167	Primates	Cercopithecidae	Presbytis rubicunda	LC	5.540	1.8779
3168	Rodentia	Sciuridae	Sciurus ignitus	LC	5.537	1.8775

Rank	Order	Family	Species	IUCN	ED'	EDGE
3169	Chiroptera	Pteropodidae	Hypsignathus monstrosus	LC	5.534	1.8770
3170	Rodentia	Sciuridae	Cynomys gunnisoni	LC	5.508	1.8731
3171	Rodentia	Muridae	Nesoryzomys indefessus	NT	2.249	1.8714
3172	Rodentia	Muridae	Pseudomys albocinereus	LC	5.469	1.8670
3172	Rodentia	Muridae	Pseudomys apodemoides	LC	5.469	1.8670
3172	Rodentia	Muridae	Pseudomys bolami	LC	5.469	1.8670
3172	Rodentia	Muridae	Pseudomys chapmani	LC	5.469	1.8670
3172	Rodentia	Muridae	Pseudomys fuscus	LC	5.469	1.8670
3172	Rodentia	Muridae	Pseudomys gracilicaudatus	LC	5.469	1.8670
3172	Rodentia	Muridae	Pseudomys hermannsburgensis	LC	5.469	1.8670
3172	Rodentia	Muridae	Pseudomys higginsi	LC	5.469	1.8670
3172	Rodentia	Muridae	Pseudomys laborifex	LC	5.469	1.8670
3172	Rodentia	Muridae	Pseudomys novaehollandiae	LC	5.469	1.8670
3182	Carnivora	Procyonidae	Procyon cancrivorus	LC	5.466	1.8666
3182	Carnivora	Procyonidae	Procyon lotor	LC	5.466	1.8666
3184	Artiodactyla	Cervidae	Mazama rufina	NT	2.224	1.8638
3185	Rodentia	Muridae	Microtus pennsylvanicus	LC	5.447	1.8636
3186	Rodentia	Sciuridae	Spermophilus fulvus	LC	5.436	1.8620
3187	Chiroptera	Vespertilionidae	Myotis auriculus	LC	5.407	1.8573
3187	Chiroptera	Vespertilionidae	Myotis evotis	LC	5.407	1.8573
3187	Chiroptera	Vespertilionidae	Myotis keenii	LC	5.407	1.8573
3190	Rodentia	Sciuridae	Spermophilus xanthoprymnus	LC	5.401	1.8565
3191	Rodentia	Ctenomyidae	Ctenomys latro	NT	2.199	1.8559
3191	Rodentia	Ctenomyidae	Ctenomys nattereri	NT	2.199	1.8559
3191	Rodentia	Ctenomyidae	Ctenomys sociabilis	NT	2.199	1.8559
3194	Carnivora	Mustelidae	Mustela eversmannii	LC	5.388	1.8544
3195	Carnivora	Otariidae	Arctocephalus australis	LC	5.329	1.8452
3195	Carnivora	Otariidae	Arctocephalus forsteri	LC	5.329	1.8452
3197	Rodentia	Muridae	Lenomys meyeri	LC	5.317	1.8433
3197	Rodentia	Muridae	Nesokia indica	LC	5.317	1.8433
3197	Rodentia	Muridae	Paruromys dominator	LC	5.317	1.8433
3197	Rodentia	Muridae	Pithecheir parvus	LC	5.317	1.8433
3197	Rodentia	Muridae	Vandeleuria oleracea	LC	5.317	1.8433
3197	Rodentia	Muridae	Zelotomys hildegardeae	LC	5.317	1.8433
3197	Rodentia	Muridae	Zelotomys woosnami	LC	5.317	1.8433
3204	Chiroptera	Vespertilionidae	Myotis simus	LC	5.316	1.8431
3205	Rodentia	Muridae	Microtus fortis	LC	5.272	1.8361
3205	Rodentia	Muridae	Microtus hyperboreus	LC	5.272	1.8361
3207	Chiroptera	Vespertilionidae	Scotophilus heathi	LC	5.269	1.8356
3207	Chiroptera	Vespertilionidae	Scotophilus viridis	LC	5.269	1.8356
3209	Carnivora	Mustelidae	Lontra canadensis	LC	5.268	1.8355
3210	Chiroptera	Vespertilionidae	Myotis keaysi	LC	5.264	1.8349
3210	Chiroptera	Vespertilionidae	Myotis nigricans	LC	5.264	1.8349
3212	Artiodactyla	Cervidae	Cervus elaphus	LC	5.259	1.8341
3212	Artiodactyla	Cervidae	Cervus nippon	LC	5.259	1.8341
3212	Artiodactyla	Cervidae	Cervus timorensis	LC	5.259	1.8341
3212	Artiodactyla	Cervidae	Cervus unicolor	LC	5.259	1.8341
3216	Rodentia	Muridae	Oligoryzomys vegetus	NT	2.123	1.8321
3217	Rodentia	Muridae	Rattus colletti	LC	5.242	1.8312
3217	Rodentia	Muridae	Rattus fuscipes	LC	5.242	1.8312
3217	Rodentia	Muridae	Rattus lutreolus	LC	5.242	1.8312
3217	Rodentia	Muridae	Rattus villosissimus	LC	5.242	1.8312
3221	Rodentia	Sciuridae	Sciurus stramineus	LC	5.233	1.8298
3222	Rodentia	Muridae	Microtus gerbei	LC	5.228	1.8291
3222	Rodentia	Muridae	Microtus savii	LC	5.228	1.8291

Rank	Order	Family	Species	IUCN	ED'	EDGE
3224	Chiroptera	Vespertilionidae	Myotis daubentoni	LC	5.220	1.8277
3224	Chiroptera	Vespertilionidae	Myotis macrodactylus	LC	5.220	1.8277
3226	Rodentia	Heteromyidae	Dipodomys merriami	LC	5.210	1.8261
3227	Rodentia	Sciuridae	Ammospermophilus insularis	LC	5.210	1.8261
3228	Rodentia	Sciuridae	Spermophilus beecheyi	LC	5.210	1.8261
3229	Rodentia	Heteromyidae	Dipodomys panamintinus	LC	5.203	1.8250
3230	Chiroptera	Phyllostomidae	Chiroderma trinitatum	LC	5.203	1.8250
3230	Chiroptera	Phyllostomidae	Chiroderma villosum	LC	5.203	1.8250
3232	Rodentia	Muridae	Euneomys chinchilloides	LC	5.198	1.8243
3232	Rodentia	Muridae	Euneomys mordax	LC	5.198	1.8243
3232	Rodentia	Muridae	Euneomys petersoni	LC	5.198	1.8243
3232	Rodentia	Muridae	Neacomys guianae	LC	5.198	1.8243
3232	Rodentia	Muridae	Neacomys spinosus	LC	5.198	1.8243
3232	Rodentia	Muridae	Neacomys tenuipes	LC	5.198	1.8243
3238	Chiroptera	Phyllostomidae	Platyrrhinus brachycephalus	LC	5.181	1.8215
3238	Chiroptera	Phyllostomidae	Platyrrhinus dorsalis	LC	5.181	1.8215
3238	Chiroptera	Phyllostomidae	Platyrrhinus helleri	LC	5.181	1.8215
3238	Chiroptera	Phyllostomidae	Platyrrhinus lineatus	LC	5.181	1.8215
3238	Chiroptera	Phyllostomidae	Platyrrhinus vittatus	LC	5.181	1.8215
3243	Rodentia	Sciuridae	Sciurus aestuans	LC	5.144	1.8154
3244	Artiodactyla	Bovidae	Capra ibex	LC	5.129	1.8130
3244	Artiodactyla	Bovidae	Capra sibirica	LC	5.129	1.8130
3246	Primates	Cebidae	Alouatta palliata	LC	5.123	1.8120
3247	Primates	Callitrichidae	Saguinus geoffroyi	LC	5.122	1.8119
3248	Rodentia	Echimyidae	Echimys braziliensis	LC	5.053	1.8005
3248	Rodentia	Echimyidae	Echimys dasythrix	LC	5.053	1.8005
3248	Rodentia	Echimyidae	Echimys grandis	LC	5.053	1.8005
3248	Rodentia	Echimyidae	Echimys lamarum	LC	5.053	1.8005
3248	Rodentia	Echimyidae	Echimys macrurus	LC	5.053	1.8005
3248	Rodentia	Echimyidae	Echimys nigrispinus	LC	5.053	1.8005
3248	Rodentia	Echimyidae	Echimys saturnus	LC	5.053	1.8005
3248	Rodentia	Echimyidae	Echimys semivillosus	LC	5.053	1.8005
3248	Rodentia	Echimyidae	Echimys unicolor	LC	5.053	1.8005
3257	Insectivora	Soricidae	Sorex asper	LC	5.040	1.7985
3257	Insectivora	Soricidae	Sorex tundrensis	LC	5.040	1.7985
3259	Rodentia	Heteromyidae	Dipodomys agilis	LC	5.034	1.7974
3260	Rodentia	Sciuridae	Sciurus pyrrhinus	LC	5.025	1.7959
3261	Rodentia	Muridae	Microtus irene	LC	5.022	1.7953
3261	Rodentia	Muridae	Microtus sikimensis	LC	5.022	1.7953
3263	Rodentia	Muridae	Thomasomys monochromos	NT	2.005	1.7934
3263	Rodentia	Muridae	Thomasomys notatus	NT	2.005	1.7934
3265	Rodentia	Sciuridae	Spermophilus dauricus	LC	5.010	1.7934
3265	Rodentia	Sciuridae	Spermophilus spilosoma	LC	5.010	1.7934
3267	Rodentia	Heteromyidae	Dipodomys californicus	LC	4.986	1.7894
3268	Chiroptera	Pteropodidae	Nanonycteris veldkampi	LC	4.943	1.7822
3269	Rodentia	Sciuridae	Sciurus aureogaster	LC	4.935	1.7809
3270	Rodentia	Muridae	Pseudoryzomys simplex	LC	4.928	1.7797
3271	Chiroptera	Vespertilionidae	Nyctophilus arnhemensis	LC	4.925	1.7792
3271	Chiroptera	Vespertilionidae	Nyctophilus geoffroyi	LC	4.925	1.7792
3271	Chiroptera	Vespertilionidae	Nyctophilus gouldi	LC	4.925	1.7792
3271	Chiroptera	Vespertilionidae	Nyctophilus microtis	LC	4.925	1.7792
3275	Rodentia	Geomyidae	Orthogeomys cavator	LC	4.917	1.7778
3275	Rodentia	Geomyidae	Orthogeomys dariensis	LC	4.917	1.7778
3275	Rodentia	Geomyidae	Orthogeomys grandis	LC	4.917	1.7778
3275	Rodentia	Geomyidae	Orthogeomys hispidus	LC	4.917	1.7778
3275	Rodentia	Geomyidae	Orthogeomys lanius	LC	4.917	1.7778

Rank	Order	Family	Species	IUCN	ED'	EDGE
3275	Rodentia	Geomyidae	Orthogeomys matagalpae	LC	4.917	1.7778
3275	Rodentia	Geomyidae	Orthogeomys thaeleri	LC	4.917	1.7778
3275	Rodentia	Geomyidae	Orthogeomys underwoodi	LC	4.917	1.7778
3283	Rodentia	Sciuridae	Sciurus variegatoides	LC	4.911	1.7769
3284	Rodentia	Echimyidae	Clyomys laticeps	LC	4.904	1.7756
3285	Rodentia	Muridae	Calomys boliviae	LC	4.898	1.7746
3285	Rodentia	Muridae	Calomys callidus	LC	4.898	1.7746
3285	Rodentia	Muridae	Calomys callosus	LC	4.898	1.7746
3285	Rodentia	Muridae	Calomys hummelincki	LC	4.898	1.7746
3285	Rodentia	Muridae	Calomys laucha	LC	4.898	1.7746
3285	Rodentia	Muridae	Calomys lepidus	LC	4.898	1.7746
3285	Rodentia	Muridae	Calomys musculinus	LC	4.898	1.7746
3285	Rodentia	Muridae	Calomys sorellus	LC	4.898	1.7746
3285	Rodentia	Muridae	Calomys tener	LC	4.898	1.7746
3294	Primates	Cercopithecidae	Presbytis femoralis	LC	4.859	1.7680
3295	Rodentia	Sciuridae	Sciurus lis	LC	4.846	1.7657
3296	Rodentia	Muridae	Melomys burtoni	LC	4.846	1.7657
3296	Rodentia	Muridae	Melomys capensis	LC	4.846	1.7657
3296	Rodentia	Muridae	Melomys cervinipes	LC	4.846	1.7657
3296	Rodentia	Muridae	Melomys gracilis	LC	4.846	1.7657
3296	Rodentia	Muridae	Melomys lanosus	LC	4.846	1.7657
3296	Rodentia	Muridae	Melomys leucogaster	LC	4.846	1.7657
3296	Rodentia	Muridae	Melomys levipes	LC	4.846	1.7657
3296	Rodentia	Muridae	Melomys lorentzii	LC	4.846	1.7657
3296	Rodentia	Muridae	Melomys mollis	LC	4.846	1.7657
3296	Rodentia	Muridae	Melomys moncktoni	LC	4.846	1.7657
3296	Rodentia	Muridae	Melomys platyops	LC	4.846	1.7657
3296	Rodentia	Muridae	Melomys rattoides	LC	4.846	1.7657
3296	Rodentia	Muridae	Melomys rubex	LC	4.846	1.7657
3296	Rodentia	Muridae	Melomys rufescens	LC	4.846	1.7657
3310	Rodentia	Sciuridae	Marmota monax	LC	4.841	1.7650
3311	Rodentia	Echimyidae	Dactylomys boliviensis	LC	4.836	1.7640
3311	Rodentia	Echimyidae	Dactylomys dactylinus	LC	4.836	1.7640
3313	Rodentia	Echimyidae	Carterodon sulcidens	NT	1.912	1.7622
3313	Rodentia	Echimyidae	Proechimys albispinus	NT	1.912	1.7622
3313	Rodentia	Echimyidae	Proechimys gorgonae	NT	1.912	1.7622
3316	Primates	Callitrichidae	Callithrix argentata	LC	4.822	1.7616
3316	Primates	Callitrichidae	Callithrix humeralifera	LC	4.822	1.7616
3318	Chiroptera	Pteropodidae	Pteropus seychellensis	LC	4.813	1.7601
3319	Carnivora	Mustelidae	Mustela nudipes	LC	4.769	1.7525
3319	Carnivora	Mustelidae	Mustela sibirica	LC	4.769	1.7525
3321	Rodentia	Muridae	Melanomys caliginosus	LC	4.752	1.7496
3321	Rodentia	Muridae	Melanomys robustulus	LC	4.752	1.7496
3321	Rodentia	Muridae	Melanomys zunigae	LC	4.752	1.7496
3321	Rodentia	Muridae	Nectomys palmipes	LC	4.752	1.7496
3321	Rodentia	Muridae	Nectomys squamipes	LC	4.752	1.7496
3326	Chiroptera	Vespertilionidae	Murina cyclotis	LC	4.752	1.7495
3327	Insectivora	Soricidae	Suncus etruscus	LC	4.733	1.7463
3327	Insectivora	Soricidae	Suncus infinitesimus	LC	4.733	1.7463
3327	Insectivora	Soricidae	Suncus lixus	LC	4.733	1.7463
3327	Insectivora	Soricidae	Suncus madagascariensis	LC	4.733	1.7463
3327	Insectivora	Soricidae	Suncus murinus	LC	4.733	1.7463
3327	Insectivora	Soricidae	Suncus remyi	LC	4.733	1.7463
3327	Insectivora	Soricidae	Suncus stoliczkanus	LC	4.733	1.7463
3327	Insectivora	Soricidae	Suncus varilla	LC	4.733	1.7463
3335	Rodentia	Muridae	Rattus koopmani	NT	1.865	1.7456

Rank	Order	Family	Species	IUCN	ED'	EDGE
3336	Rodentia	Sciuridae	Microsciurus santanderensis	LC	4.709	1.7421
3337	Chiroptera	Vespertilionidae	Scotophilus leucogaster	LC	4.694	1.7395
3338	Artiodactyla	Bovidae	Ovibos moschatus	LC	4.685	1.7379
3339	Rodentia	Muridae	Akodon sanctipaulensis	NT	1.838	1.7361
3340	Rodentia	Sciuridae	Cynomys leucurus	LC	4.623	1.7269
3341	Rodentia	Sciuridae	Sciurus flammifer	LC	4.591	1.7211
3342	Rodentia	Heteromyidae	Dipodomys venustus	LC	4.588	1.7207
3343	Rodentia	Heteromyidae	Dipodomys heermanni	LC	4.582	1.7196
3344	Insectivora	Soricidae	Sorex bendirii	LC	4.553	1.7143
3344	Insectivora	Soricidae	Sorex palustris	LC	4.553	1.7143
3346	Rodentia	Muridae	Akodon aerosus	LC	4.546	1.7130
3346	Rodentia	Muridae	Akodon hershkovitzi	LC	4.546	1.7130
3346	Rodentia	Muridae	Akodon illuteus	LC	4.546	1.7130
3346	Rodentia	Muridae	Akodon kempi	LC	4.546	1.7130
3346	Rodentia	Muridae	Akodon nigrita	LC	4.546	1.7130
3346	Rodentia	Muridae	Akodon xanthorhinus	LC	4.546	1.7130
3352	Rodentia	Muridae	Sigmodon alleni	LC	4.546	1.7130
3352	Rodentia	Muridae	Sigmodon alstoni	LC	4.546	1.7130
3352	Rodentia	Muridae	Sigmodon arizonae	LC	4.546	1.7130
3352	Rodentia	Muridae	Sigmodon fulviventer	LC	4.546	1.7130
3352	Rodentia	Muridae	Sigmodon hispidus	LC	4.546	1.7130
3352	Rodentia	Muridae	Sigmodon inopinatus	LC	4.546	1.7130
3352	Rodentia	Muridae	Sigmodon leucotis	LC	4.546	1.7130
3352	Rodentia	Muridae	Sigmodon mascotensis	LC	4.546	1.7130
3352	Rodentia	Muridae	Sigmodon ochrognathus	LC	4.546	1.7130
3352	Rodentia	Muridae	Sigmodon peruanus	LC	4.546	1.7130
3362	Insectivora	Soricidae	Sorex monticolus	LC	4.521	1.7086
3362	Insectivora	Soricidae	Sorex pacificus	LC	4.521	1.7086
3362	Insectivora	Soricidae	Sorex vagrans	LC	4.521	1.7086
3365	Insectivora	Soricidae	Sorex coronatus	LC	4.521	1.7085
3366	Rodentia	Muridae	Dasymys incomtus	LC	4.487	1.7024
3366	Rodentia	Muridae	Dasymys rufulus	LC	4.487	1.7024
3368	Chiroptera	Vespertilionidae	Myotis tricolor	LC	4.475	1.7001
3369	Chiroptera	Vespertilionidae	Eptesicus diminutus	LC	4.472	1.6997
3370	Rodentia	Muridae	Microtus duodecimcostatus	LC	4.466	1.6985
3370	Rodentia	Muridae	Microtus lusitanicus	LC	4.466	1.6985
3372	Chiroptera	Pteropodidae	Pteropus giganteus	LC	4.445	1.6947
3372	Chiroptera	Pteropodidae	Pteropus lombocensis	LC	4.445	1.6947
3372	Chiroptera	Pteropodidae	Pteropus lylei	LC	4.445	1.6947
3372	Chiroptera	Pteropodidae	Pteropus macrotis	LC	4.445	1.6947
3372	Chiroptera	Pteropodidae	Pteropus poliocephalus	LC	4.445	1.6947
3372	Chiroptera	Pteropodidae	Pteropus rayneri	LC	4.445	1.6947
3372	Chiroptera	Pteropodidae	Pteropus vampyrus	LC	4.445	1.6947
3379	Chiroptera	Phyllostomidae	Artibeus jamaicensis	LC	4.404	1.6871
3379	Chiroptera	Phyllostomidae	Artibeus lituratus	LC	4.404	1.6871
3379	Chiroptera	Phyllostomidae	Artibeus planirostris	LC	4.404	1.6871
3382	Rodentia	Muridae	Rattus leucopus	LC	4.402	1.6868
3382	Rodentia	Muridae	Rattus mordax	LC	4.402	1.6868
3382	Rodentia	Muridae	Rattus morotaiensis	LC	4.402	1.6868
3382	Rodentia	Muridae	Rattus novaeguineae	LC	4.402	1.6868
3382	Rodentia	Muridae	Rattus praetor	LC	4.402	1.6868
3382	Rodentia	Muridae	Rattus steini	LC	4.402	1.6868
3388	Rodentia	Sciuridae	Sciurus colliaei	LC	4.394	1.6854
3389	Rodentia	Muridae	Microtus maximowiczii	LC	4.371	1.6809
3389	Rodentia	Muridae	Microtus middendorffi	LC	4.371	1.6809
3389	Rodentia	Muridae	Microtus mongolicus	LC	4.371	1.6809

Rank	Order	Family	Species	IUCN	ED'	EDGE
3392	Rodentia	Muridae	Akodon lanosus	LC	4.350	1.6772
3392	Rodentia	Muridae	Akodon longipilis	LC	4.350	1.6772
3392	Rodentia	Muridae	Akodon mansoensis	LC	4.350	1.6772
3392	Rodentia	Muridae	Akodon sanborni	LC	4.350	1.6772
3396	Chiroptera	Vespertilionidae	Rhogeessa tumida	LC	4.349	1.6769
3397	Insectivora	Soricidae	Sorex daphaenodon	LC	4.336	1.6745
3397	Insectivora	Soricidae	Sorex satunini	LC	4.336	1.6745
3399	Rodentia	Muridae	Akodon budini	LC	4.325	1.6723
3400	Rodentia	Sciuridae	Spermophilus tridecemlineatus	LC	4.307	1.6691
3401	Rodentia	Muridae	Bibimys chacoensis	LC	4.302	1.6681
3401	Rodentia	Muridae	Bibimys labiosus	LC	4.302	1.6681
3403	Rodentia	Sciuridae	Sciurus granatensis	LC	4.295	1.6667
3404	Rodentia	Muridae	Microtus daghestanicus	LC	4.290	1.6657
3404	Rodentia	Muridae	Microtus subterraneus	LC	4.290	1.6657
3406	Rodentia	Muridae	Microtus multiplex	LC	4.282	1.6643
3406	Rodentia	Muridae	Microtus schelkovnikovi	LC	4.282	1.6643
3408	Insectivora	Soricidae	Crocidura beatus	VU	0.320	1.6639
3408	Insectivora	Soricidae	Crocidura canariensis	VU	0.320	1.6639
3408	Insectivora	Soricidae	Crocidura eisentrauti	VU	0.320	1.6639
3408	Insectivora	Soricidae	Crocidura glassi	VU	0.320	1.6639
3408	Insectivora	Soricidae	Crocidura grayi	VU	0.320	1.6639
3408	Insectivora	Soricidae	Crocidura kivuana	VU	0.320	1.6639
3408	Insectivora	Soricidae	Crocidura lucina	VU	0.320	1.6639
3408	Insectivora	Soricidae	Crocidura macmillani	VU	0.320	1.6639
3408	Insectivora	Soricidae	Crocidura manengubae	VU	0.320	1.6639
3408	Insectivora	Soricidae	Crocidura nimbae	VU	0.320	1.6639
3408	Insectivora	Soricidae	Crocidura palawanensis	VU	0.320	1.6639
3408	Insectivora	Soricidae	Crocidura pergrisea	VU	0.320	1.6639
3408	Insectivora	Soricidae	Crocidura phaeura	VU	0.320	1.6639
3408	Insectivora	Soricidae	Crocidura stenocephala	VU	0.320	1.6639
3408	Insectivora	Soricidae	Crocidura tansaniana	VU	0.320	1.6639
3408	Insectivora	Soricidae	Crocidura tarella	VU	0.320	1.6639
3408	Insectivora	Soricidae	Crocidura tenuis	VU	0.320	1.6639
3408	Insectivora	Soricidae	Crocidura zimmermanni	VU	0.320	1.6639
3426	Rodentia	Muridae	Arborimus longicaudus	LC	4.258	1.6598
3427	Chiroptera	Pteropodidae	Pteropus caniceps	LC	4.254	1.6590
3427	Chiroptera	Pteropodidae	Pteropus conspicillatus	LC	4.254	1.6590
3427	Chiroptera	Pteropodidae	Pteropus melanopogon	LC	4.254	1.6590
3427	Chiroptera	Pteropodidae	Pteropus tonganus	LC	4.254	1.6590
3431	Chiroptera	Vespertilionidae	Scotophilus kuhlii	LC	4.242	1.6568
3432	Rodentia	Muridae	Eligmodontia moreni	LC	4.202	1.6490
3432	Rodentia	Muridae	Eligmodontia morgani	LC	4.202	1.6490
3432	Rodentia	Muridae	Eligmodontia puerulus	LC	4.202	1.6490
3432	Rodentia	Muridae	Eligmodontia typus	LC	4.202	1.6490
3436	Chiroptera	Rhinolophidae	Rhinolophus canuti	NT	1.600	1.6487
3436	Chiroptera	Rhinolophidae	Rhinolophus creaghi	NT	1.600	1.6487
3436	Chiroptera	Rhinolophidae	Rhinolophus nereis	NT	1.600	1.6487
3439	Rodentia	Sciuridae	Marmota broweri	LC	4.181	1.6449
3439	Rodentia	Sciuridae	Marmota marmota	LC	4.181	1.6449
3441	Rodentia	Erethizontidae	Coendou bicolor	LC	4.163	1.6415
3441	Rodentia	Erethizontidae	Coendou koopmani	LC	4.163	1.6415
3441	Rodentia	Erethizontidae	Coendou prehensilis	LC	4.163	1.6415
3441	Rodentia	Erethizontidae	Coendou rothschildi	LC	4.163	1.6415
3445	Carnivora	Canidae	Alopex lagopus	LC	4.154	1.6397
3445	Carnivora	Canidae	Vulpes velox	LC	4.154	1.6397

Rank	Order	Family	Species	IUCN	ED'	EDGE
3447	Rodentia	Muridae	Akodon bogotensis	LC	4.131	1.6352
3447	Rodentia	Muridae	Akodon latebricola	LC	4.131	1.6352
3447	Rodentia	Muridae	Akodon mimus	LC	4.131	1.6352
3450	Insectivora	Soricidae	Sorex bairdii	LC	4.113	1.6318
3450	Insectivora	Soricidae	Sorex cinereus	LC	4.113	1.6318
3450	Insectivora	Soricidae	Sorex dispar	LC	4.113	1.6318
3450	Insectivora	Soricidae	Sorex fumeus	LC	4.113	1.6318
3450	Insectivora	Soricidae	Sorex haydeni	LC	4.113	1.6318
3450	Insectivora	Soricidae	Sorex hoyi	LC	4.113	1.6318
3450	Insectivora	Soricidae	Sorex longirostris	LC	4.113	1.6318
3450	Insectivora	Soricidae	Sorex nanus	LC	4.113	1.6318
3450	Insectivora	Soricidae	Sorex ornatus	LC	4.113	1.6318
3450	Insectivora	Soricidae	Sorex portenkoi	LC	4.113	1.6318
3450	Insectivora	Soricidae	Sorex preblei	LC	4.113	1.6318
3450	Insectivora	Soricidae	Sorex tenellus	LC	4.113	1.6318
3450	Insectivora	Soricidae	Sorex ugyunak	LC	4.113	1.6318
3463	Chiroptera	Rhinolophidae	Hipposideros ater	LC	4.100	1.6292
3463	Chiroptera	Rhinolophidae	Hipposideros beatus	LC	4.100	1.6292
3463	Chiroptera	Rhinolophidae	Hipposideros bicolor	LC	4.100	1.6292
3463	Chiroptera	Rhinolophidae	Hipposideros caffer	LC	4.100	1.6292
3463	Chiroptera	Rhinolophidae	Hipposideros cineraceus	LC	4.100	1.6292
3463	Chiroptera	Rhinolophidae	Hipposideros dyacorum	LC	4.100	1.6292
3463	Chiroptera	Rhinolophidae	Hipposideros fulvus	LC	4.100	1.6292
3463	Chiroptera	Rhinolophidae	Hipposideros galeritus	LC	4.100	1.6292
3463	Chiroptera	Rhinolophidae	Hipposideros maggietaylorae	LC	4.100	1.6292
3463	Chiroptera	Rhinolophidae	Hipposideros pomona	LC	4.100	1.6292
3463	Chiroptera	Rhinolophidae	Hipposideros speoris	LC	4.100	1.6292
3474	Rodentia	Sciuridae	Sciurus alleni	LC	4.084	1.6262
3475	Rodentia	Muridae	Chelemys macronyx	LC	4.084	1.6261
3475	Rodentia	Muridae	Chelemys megalonyx	LC	4.084	1.6261
3475	Rodentia	Muridae	Microryzomys altissimus	LC	4.084	1.6261
3475	Rodentia	Muridae	Microryzomys minutus	LC	4.084	1.6261
3475	Rodentia	Muridae	Sigmodontomys alfari	LC	4.084	1.6261
3475	Rodentia	Muridae	Thalpomys cerradensis	LC	4.084	1.6261
3475	Rodentia	Muridae	Thalpomys lasiotis	LC	4.084	1.6261
3475	Rodentia	Muridae	Wilfredomys oenax	LC	4.084	1.6261
3475	Rodentia	Muridae	Wilfredomys pictipes	LC	4.084	1.6261
3475	Rodentia	Muridae	Zygodontomys brevicauda	LC	4.084	1.6261
3475	Rodentia	Muridae	Zygodontomys brunneus	LC	4.084	1.6261
3486	Insectivora	Soricidae	Sorex araneus	LC	4.074	1.6242
3486	Insectivora	Soricidae	Sorex granarius	LC	4.074	1.6242
3488	Carnivora	Canidae	Vulpes corsac	LC	4.053	1.6201
3488	Carnivora	Canidae	Vulpes ferrilata	LC	4.053	1.6201
3488	Carnivora	Canidae	Vulpes vulpes	LC	4.053	1.6201
3491	Chiroptera	Pteropodidae	Epomops buettikoferi	LC	4.050	1.6194
3491	Chiroptera	Pteropodidae	Epomops dobsoni	LC	4.050	1.6194
3491	Chiroptera	Pteropodidae	Epomops franqueti	LC	4.050	1.6194
3494	Rodentia	Sciuridae	Ammospermophilus harrisii	LC	4.050	1.6193
3494	Rodentia	Sciuridae	Ammospermophilus interpres	LC	4.050	1.6193
3494	Rodentia	Sciuridae	Ammospermophilus leucurus	LC	4.050	1.6193
3497	Chiroptera	Vespertilionidae	Scotophilus dinganii	LC	4.045	1.6184
3497	Chiroptera	Vespertilionidae	Scotophilus nux	LC	4.045	1.6184
3499	Chiroptera	Pteropodidae	Epomophorus wahlbergi	LC	4.041	1.6175
3500	Rodentia	Muridae	Rattus annandalei	LC	4.036	1.6167
3500	Rodentia	Muridae	Rattus exulans	LC	4.036	1.6167
3500	Rodentia	Muridae	Rattus korinchi	LC	4.036	1.6167

Rank	Order	Family	Species	IUCN	ED'	EDGE
3500	Rodentia	Muridae	Rattus norvegicus	LC	4.036	1.6167
3504	Rodentia	Sciuridae	Marmota flaviventris	LC	4.020	1.6135
3505	Rodentia	Muridae	Rhynchomys soricoides	LC	3.999	1.6093
3506	Rodentia	Sciuridae	Spermophilus erythrogenys	LC	3.947	1.5989
3506	Rodentia	Sciuridae	Spermophilus pygmaeus	LC	3.947	1.5989
3506	Rodentia	Sciuridae	Spermophilus relictus	LC	3.947	1.5989
3509	Rodentia	Muridae	Blarinomys breviceps	NT	1.435	1.5833
3509	Rodentia	Muridae	Lenoxus apicalis	NT	1.435	1.5833
3509	Rodentia	Muridae	Podoxymys roraimae	NT	1.435	1.5833
3512	Rodentia	Sciuridae	Microsciurus flaviventer	LC	3.868	1.5828
3512	Rodentia	Sciuridae	Microsciurus mimulus	LC	3.868	1.5828
3514	Rodentia	Sciuridae	Sciurus griseus	LC	3.867	1.5825
3515	Primates	Callitrichidae	Callithrix jacchus	LC	3.853	1.5795
3515	Primates	Callitrichidae	Callithrix kuhlii	LC	3.853	1.5795
3515	Primates	Callitrichidae	Callithrix penicillata	LC	3.853	1.5795
3518	Rodentia	Muridae	Clethrionomys californicus	LC	3.825	1.5738
3518	Rodentia	Muridae	Clethrionomys centralis	LC	3.825	1.5738
3518	Rodentia	Muridae	Clethrionomys gapperi	LC	3.825	1.5738
3518	Rodentia	Muridae	Clethrionomys glareolus	LC	3.825	1.5738
3518	Rodentia	Muridae	Clethrionomys rufocanus	LC	3.825	1.5738
3518	Rodentia	Muridae	Clethrionomys rutilus	LC	3.825	1.5738
3524	Chiroptera	Vespertilionidae	Myotis ikonnikovi	LC	3.824	1.5736
3524	Chiroptera	Vespertilionidae	Myotis muricola	LC	3.824	1.5736
3526	Rodentia	Echimyidae	Mesomys hispidus	LC	3.805	1.5696
3526	Rodentia	Echimyidae	Mesomys leniceps	LC	3.805	1.5696
3526	Rodentia	Echimyidae	Mesomys stimulax	LC	3.805	1.5696
3529	Rodentia	Sciuridae	Sciurus carolinensis	LC	3.804	1.5695
3529	Rodentia	Sciuridae	Sciurus niger	LC	3.804	1.5695
3531	Rodentia	Sciuridae	Marmota baibacina	LC	3.801	1.5688
3532	Rodentia	Muridae	Alticola argentatus	LC	3.752	1.5586
3532	Rodentia	Muridae	Alticola barakshin	LC	3.752	1.5586
3532	Rodentia	Muridae	Alticola macrotis	LC	3.752	1.5586
3532	Rodentia	Muridae	Alticola semicanus	LC	3.752	1.5586
3532	Rodentia	Muridae	Alticola stoliczkanus	LC	3.752	1.5586
3532	Rodentia	Muridae	Alticola stracheyi	LC	3.752	1.5586
3532	Rodentia	Muridae	Alticola tuvinicus	LC	3.752	1.5586
3539	Chiroptera	Vespertilionidae	Myotis californicus	LC	3.751	1.5583
3539	Chiroptera	Vespertilionidae	Myotis leibii	LC	3.751	1.5583
3541	Chiroptera	Vespertilionidae	Eptesicus bottae	LC	3.744	1.5570
3541	Chiroptera	Vespertilionidae	Eptesicus brasiliensis	LC	3.744	1.5570
3541	Chiroptera	Vespertilionidae	Eptesicus furinalis	LC	3.744	1.5570
3541	Chiroptera	Vespertilionidae	Eptesicus fuscus	LC	3.744	1.5570
3541	Chiroptera	Vespertilionidae	Eptesicus hottentotus	LC	3.744	1.5570
3541	Chiroptera	Vespertilionidae	Eptesicus serotinus	LC	3.744	1.5570
3547	Primates	Cercopithecidae	Trachypithecus obscurus	LC	3.726	1.5531
3548	Rodentia	Muridae	Neotoma albigula	LC	3.717	1.5512
3548	Rodentia	Muridae	Neotoma angustapalata	LC	3.717	1.5512
3548	Rodentia	Muridae	Neotoma chrysomelas	LC	3.717	1.5512
3548	Rodentia	Muridae	Neotoma devia	LC	3.717	1.5512
3548	Rodentia	Muridae	Neotoma floridana	LC	3.717	1.5512
3548	Rodentia	Muridae	Neotoma goldmani	LC	3.717	1.5512
3548	Rodentia	Muridae	Neotoma lepida	LC	3.717	1.5512
3548	Rodentia	Muridae	Neotoma mexicana	LC	3.717	1.5512
3548	Rodentia	Muridae	Neotoma micropus	LC	3.717	1.5512
3548	Rodentia	Muridae	Neotoma stephensi	LC	3.717	1.5512
3558	Chiroptera	Vespertilionidae	Chalinolobus argentatus	LC	3.711	1.5500

Rank	Order	Family	Species	IUCN	ED'	EDGE
3558	Chiroptera	Vespertilionidae	Chalinolobus variegatus	LC	3.711	1.5500
3560	Rodentia	Muridae	Rhipidomys austrinus	LC	3.700	1.5475
3560	Rodentia	Muridae	Rhipidomys couesi	LC	3.700	1.5475
3560	Rodentia	Muridae	Rhipidomys fulviventer	LC	3.700	1.5475
3560	Rodentia	Muridae	Rhipidomys latimanus	LC	3.700	1.5475
3560	Rodentia	Muridae	Rhipidomys leucodactylus	LC	3.700	1.5475
3560	Rodentia	Muridae	Rhipidomys macconnelli	LC	3.700	1.5475
3560	Rodentia	Muridae	Rhipidomys mastacalis	LC	3.700	1.5475
3560	Rodentia	Muridae	Rhipidomys nitela	LC	3.700	1.5475
3560	Rodentia	Muridae	Rhipidomys venezuelae	LC	3.700	1.5475
3560	Rodentia	Muridae	Rhipidomys venustus	LC	3.700	1.5475
3560	Rodentia	Muridae	Rhipidomys wetzeli	LC	3.700	1.5475
3571	Carnivora	Canidae	Cerdocyon thous	LC	3.683	1.5439
3572	Chiroptera	Vespertilionidae	Nyctalus noctula	LC	3.671	1.5414
3573	Carnivora	Canidae	Canis mesomelas	LC	3.665	1.5402
3574	Rodentia	Sciuridae	Sciurus oculatus	LC	3.661	1.5393
3575	Rodentia	Muridae	Lophuromys flavopunctatus	LC	3.643	1.5353
3575	Rodentia	Muridae	Lophuromys luteogaster	LC	3.643	1.5353
3575	Rodentia	Muridae	Lophuromys nudicaudus	LC	3.643	1.5353
3575	Rodentia	Muridae	Lophuromys sikapusi	LC	3.643	1.5353
3575	Rodentia	Muridae	Lophuromys woosnami	LC	3.643	1.5353
3580	Rodentia	Sciuridae	Marmota sibirica	LC	3.623	1.5311
3581	Rodentia	Muridae	Microtus guatemalensis	LC	3.594	1.5247
3581	Rodentia	Muridae	Microtus longicaudus	LC	3.594	1.5247
3581	Rodentia	Muridae	Microtus mexicanus	LC	3.594	1.5247
3581	Rodentia	Muridae	Microtus ochrogaster	LC	3.594	1.5247
3581	Rodentia	Muridae	Microtus umbrosus	LC	3.594	1.5247
3586	Rodentia	Muridae	Akodon boliviensis	LC	3.572	1.5200
3586	Rodentia	Muridae	Akodon juninensis	LC	3.572	1.5200
3588	Carnivora	Canidae	Canis adustus	LC	3.538	1.5124
3588	Carnivora	Canidae	Canis aureus	LC	3.538	1.5124
3590	Rodentia	Sciuridae	Spermophilus atricapillus	LC	3.536	1.5121
3591	Rodentia	Sciuridae	Spermophilus adocetus	LC	3.525	1.5097
3591	Rodentia	Sciuridae	Spermophilus beldingi	LC	3.525	1.5097
3591	Rodentia	Sciuridae	Spermophilus mollis	LC	3.525	1.5097
3591	Rodentia	Sciuridae	Spermophilus musicus	LC	3.525	1.5097
3595	Rodentia	Muridae	Oryzomys dimidiatus	NT	1.263	1.5097
3595	Rodentia	Muridae	Oryzomys levipes	NT	1.263	1.5097
3595	Rodentia	Muridae	Oryzomys oniscus	NT	1.263	1.5097
3598	Rodentia	Sciuridae	Marmota camtschatica	LC	3.523	1.5091
3598	Rodentia	Sciuridae	Marmota himalayana	LC	3.523	1.5091
3600	Chiroptera	Pteropodidae	Micropteropus pusillus	LC	3.506	1.5055
3601	Artiodactyla	Bovidae	Gazella bennettii	LC	3.451	1.4931
3602	Rodentia	Muridae	Gerbillus allenbyi	VU	0.111	1.4920
3602	Rodentia	Muridae	Gerbillus bonhotei	VU	0.111	1.4920
3602	Rodentia	Muridae	Gerbillus hesperinus	VU	0.111	1.4920
3605	Rodentia	Sciuridae	Spermophilus armatus	LC	3.436	1.4898
3605	Rodentia	Sciuridae	Spermophilus richardsonii	LC	3.436	1.4898
3607	Rodentia	Muridae	Phyllotis amicus	LC	3.399	1.4814
3607	Rodentia	Muridae	Phyllotis andium	LC	3.399	1.4814
3607	Rodentia	Muridae	Phyllotis caprinus	LC	3.399	1.4814
3607	Rodentia	Muridae	Phyllotis darwini	LC	3.399	1.4814
3607	Rodentia	Muridae	Phyllotis definitus	LC	3.399	1.4814
3607	Rodentia	Muridae	Phyllotis gerbillus	LC	3.399	1.4814
3607	Rodentia	Muridae	Phyllotis haggardi	LC	3.399	1.4814
3607	Rodentia	Muridae	Phyllotis magister	LC	3.399	1.4814

Rank	Order	Family	Species	IUCN	ED'	EDGE
3607	Rodentia	Muridae	Phyllotis osgoodi	LC	3.399	1.4814
3607	Rodentia	Muridae	Phyllotis osilae	LC	3.399	1.4814
3607	Rodentia	Muridae	Phyllotis wolffsohni	LC	3.399	1.4814
3607	Rodentia	Muridae	Phyllotis xanthopygus	LC	3.399	1.4814
3619	Chiroptera	Pteropodidae	Dobsonia inermis	LC	3.381	1.4772
3619	Chiroptera	Pteropodidae	Dobsonia viridis	LC	3.381	1.4772
3621	Chiroptera	Pteropodidae	Dobsonia moluccensis	LC	3.301	1.4587
3621	Chiroptera	Pteropodidae	Dobsonia pannietensis	LC	3.301	1.4587
3623	Rodentia	Muridae	Microtus irani	LC	3.300	1.4585
3623	Rodentia	Muridae	Microtus socialis	LC	3.300	1.4585
3625	Rodentia	Muridae	Apodemus arianus	LC	3.249	1.4466
3625	Rodentia	Muridae	Apodemus flavicollis	LC	3.249	1.4466
3625	Rodentia	Muridae	Apodemus fulvipectus	LC	3.249	1.4466
3625	Rodentia	Muridae	Apodemus ponticus	LC	3.249	1.4466
3625	Rodentia	Muridae	Apodemus rusiges	LC	3.249	1.4466
3625	Rodentia	Muridae	Apodemus sylvaticus	LC	3.249	1.4466
3625	Rodentia	Muridae	Apodemus uralensis	LC	3.249	1.4466
3625	Rodentia	Muridae	Apodemus wardi	LC	3.249	1.4466
3633	Rodentia	Muridae	Microtus arvalis	LC	3.244	1.4456
3633	Rodentia	Muridae	Microtus kirgisorum	LC	3.244	1.4456
3633	Rodentia	Muridae	Microtus obscurus	LC	3.244	1.4456
3633	Rodentia	Muridae	Microtus rossiaemeridionalis	LC	3.244	1.4456
3633	Rodentia	Muridae	Microtus transcaspicus	LC	3.244	1.4456
3638	Chiroptera	Pteropodidae	Pteropus admiralitatum	LC	3.242	1.4451
3638	Chiroptera	Pteropodidae	Pteropus alecto	LC	3.242	1.4451
3638	Chiroptera	Pteropodidae	Pteropus griseus	LC	3.242	1.4451
3638	Chiroptera	Pteropodidae	Pteropus hypomelanus	LC	3.242	1.4451
3638	Chiroptera	Pteropodidae	Pteropus melanotus	LC	3.242	1.4451
3638	Chiroptera	Pteropodidae	Pteropus neohibernicus	LC	3.242	1.4451
3644	Rodentia	Muridae	Anonymomys mindorensis	VU	0.058	1.4428
3644	Rodentia	Muridae	Vernaya fulva	VU	0.058	1.4428
3646	Carnivora	Canidae	Canis lupus	LC	3.194	1.4337
3647	Carnivora	Canidae	Canis latrans	LC	3.188	1.4323
3648	Chiroptera	Vespertilionidae	Myotis blythii	LC	3.180	1.4304
3648	Chiroptera	Vespertilionidae	Myotis chinensis	LC	3.180	1.4304
3650	Rodentia	Sciuridae	Spermophilus parryii	LC	3.131	1.4186
3651	Chiroptera	Rhinolophidae	Rhinolophus blasii	NT	1.062	1.4170
3651	Chiroptera	Rhinolophidae	Rhinolophus capensis	NT	1.062	1.4170
3651	Chiroptera	Rhinolophidae	Rhinolophus celebensis	NT	1.062	1.4170
3651	Chiroptera	Rhinolophidae	Rhinolophus ferrumequinum	NT	1.062	1.4170
3651	Chiroptera	Rhinolophidae	Rhinolophus marshalli	NT	1.062	1.4170
3651	Chiroptera	Rhinolophidae	Rhinolophus philippinensis	NT	1.062	1.4170
3651	Chiroptera	Rhinolophidae	Rhinolophus rufus	NT	1.062	1.4170
3651	Chiroptera	Rhinolophidae	Rhinolophus shameli	NT	1.062	1.4170
3651	Chiroptera	Rhinolophidae	Rhinolophus swinnyi	NT	1.062	1.4170
3651	Chiroptera	Rhinolophidae	Rhinolophus thomasi	NT	1.062	1.4170
3651	Chiroptera	Rhinolophidae	Rhinolophus virgo	NT	1.062	1.4170
3651	Chiroptera	Rhinolophidae	Rhinolophus yunanensis	NT	1.062	1.4170
3663	Chiroptera	Vespertilionidae	Kerivoula argentata	LC	3.120	1.4159
3663	Chiroptera	Vespertilionidae	Kerivoula flora	LC	3.120	1.4159
3663	Chiroptera	Vespertilionidae	Kerivoula hardwickei	LC	3.120	1.4159
3663	Chiroptera	Vespertilionidae	Kerivoula lanosa	LC	3.120	1.4159
3663	Chiroptera	Vespertilionidae	Kerivoula papillosa	LC	3.120	1.4159
3663	Chiroptera	Vespertilionidae	Kerivoula pellucida	LC	3.120	1.4159
3663	Chiroptera	Vespertilionidae	Kerivoula picta	LC	3.120	1.4159
3663	Chiroptera	Vespertilionidae	Kerivoula whiteheadi	LC	3.120	1.4159

Rank	Order	Family	Species	IUCN	ED'	EDGE
3671	Chiroptera	Pteropodidae	Epomophorus gambianus	LC	3.105	1.4123
3671	Chiroptera	Pteropodidae	Epomophorus labiatus	LC	3.105	1.4123
3671	Chiroptera	Pteropodidae	Epomophorus minimus	LC	3.105	1.4123
3674	Chiroptera	Vespertilionidae	Myotis albescens	LC	3.083	1.4068
3674	Chiroptera	Vespertilionidae	Myotis austroriparius	LC	3.083	1.4068
3674	Chiroptera	Vespertilionidae	Myotis levis	LC	3.083	1.4068
3674	Chiroptera	Vespertilionidae	Myotis lucifugus	LC	3.083	1.4068
3674	Chiroptera	Vespertilionidae	Myotis oxyotus	LC	3.083	1.4068
3674	Chiroptera	Vespertilionidae	Myotis velifer	LC	3.083	1.4068
3674	Chiroptera	Vespertilionidae	Myotis volans	LC	3.083	1.4068
3674	Chiroptera	Vespertilionidae	Myotis yumanensis	LC	3.083	1.4068
3682	Rodentia	Muridae	Eothenomys chinensis	LC	3.044	1.3972
3682	Rodentia	Muridae	Eothenomys custos	LC	3.044	1.3972
3682	Rodentia	Muridae	Eothenomys eva	LC	3.044	1.3972
3682	Rodentia	Muridae	Eothenomys inez	LC	3.044	1.3972
3682	Rodentia	Muridae	Eothenomys melanogaster	LC	3.044	1.3972
3682	Rodentia	Muridae	Eothenomys olitor	LC	3.044	1.3972
3682	Rodentia	Muridae	Eothenomys proditor	LC	3.044	1.3972
3682	Rodentia	Muridae	Eothenomys regulus	LC	3.044	1.3972
3682	Rodentia	Muridae	Eothenomys shanseius	LC	3.044	1.3972
3691	Rodentia	Muridae	Hylomyscus aeta	LC	3.034	1.3947
3691	Rodentia	Muridae	Hylomyscus alleni	LC	3.034	1.3947
3691	Rodentia	Muridae	Hylomyscus carillus	LC	3.034	1.3947
3691	Rodentia	Muridae	Hylomyscus denniae	LC	3.034	1.3947
3691	Rodentia	Muridae	Hylomyscus parvus	LC	3.034	1.3947
3691	Rodentia	Muridae	Hylomyscus stella	LC	3.034	1.3947
3697	Rodentia	Muridae	Dicrostonyx groenlandicus	LC	2.991	1.3841
3697	Rodentia	Muridae	Dicrostonyx hudsonius	LC	2.991	1.3841
3697	Rodentia	Muridae	Dicrostonyx kilangmiutak	LC	2.991	1.3841
3697	Rodentia	Muridae	Dicrostonyx nelsoni	LC	2.991	1.3841
3697	Rodentia	Muridae	Dicrostonyx richardsoni	LC	2.991	1.3841
3697	Rodentia	Muridae	Dicrostonyx rubricatus	LC	2.991	1.3841
3697	Rodentia	Muridae	Dicrostonyx torquatus	LC	2.991	1.3841
3704	Rodentia	Sciuridae	Sciurus igniventris	LC	2.984	1.3823
3704	Rodentia	Sciuridae	Sciurus spadiceus	LC	2.984	1.3823
3706	Rodentia	Sciuridae	Spermophilus columbianus	LC	2.956	1.3753
3706	Rodentia	Sciuridae	Spermophilus undulatus	LC	2.956	1.3753
3708	Chiroptera	Vespertilionidae	Murina leucogaster	LC	2.942	1.3717
3709	Rodentia	Muridae	Oxymycterus akodontius	LC	2.894	1.3596
3709	Rodentia	Muridae	Oxymycterus angularis	LC	2.894	1.3596
3709	Rodentia	Muridae	Oxymycterus delator	LC	2.894	1.3596
3709	Rodentia	Muridae	Oxymycterus hispidus	LC	2.894	1.3596
3709	Rodentia	Muridae	Oxymycterus iheringi	LC	2.894	1.3596
3709	Rodentia	Muridae	Oxymycterus inca	LC	2.894	1.3596
3709	Rodentia	Muridae	Oxymycterus nasutus	LC	2.894	1.3596
3709	Rodentia	Muridae	Oxymycterus paramensis	LC	2.894	1.3596
3709	Rodentia	Muridae	Oxymycterus roberti	LC	2.894	1.3596
3709	Rodentia	Muridae	Oxymycterus rufus	LC	2.894	1.3596
3719	Rodentia	Muridae	Mus booduga	LC	2.883	1.3566
3719	Rodentia	Muridae	Mus caroli	LC	2.883	1.3566
3719	Rodentia	Muridae	Mus cervicolor	LC	2.883	1.3566
3719	Rodentia	Muridae	Mus cookii	LC	2.883	1.3566
3719	Rodentia	Muridae	Mus macedonicus	LC	2.883	1.3566
3719	Rodentia	Muridae	Mus musculus	LC	2.883	1.3566
3719	Rodentia	Muridae	Mus spretus	LC	2.883	1.3566
3719	Rodentia	Muridae	Mus terricolor	LC	2.883	1.3566

Rank	Order	Family	Species	IUCN	ED'	EDGE
3727	Rodentia	Muridae	Acomys cahirinus	LC	2.843	1.3463
3727	Rodentia	Muridae	Acomys cinerasceus	LC	2.843	1.3463
3727	Rodentia	Muridae	Acomys ignitus	LC	2.843	1.3463
3727	Rodentia	Muridae	Acomys kempi	LC	2.843	1.3463
3727	Rodentia	Muridae	Acomys mullah	LC	2.843	1.3463
3727	Rodentia	Muridae	Acomys percivali	LC	2.843	1.3463
3727	Rodentia	Muridae	Acomys russatus	LC	2.843	1.3463
3727	Rodentia	Muridae	Acomys spinosissimus	LC	2.843	1.3463
3727	Rodentia	Muridae	Acomys subspinosus	LC	2.843	1.3463
3727	Rodentia	Muridae	Acomys wilsoni	LC	2.843	1.3463
3737	Chiroptera	Pteropodidae	Rousettus amplexicaudatus	LC	2.773	1.3278
3737	Chiroptera	Pteropodidae	Rousettus celebensis	LC	2.773	1.3278
3737	Chiroptera	Pteropodidae	Rousettus egyptiacus	LC	2.773	1.3278
3737	Chiroptera	Pteropodidae	Rousettus leschenaulti	LC	2.773	1.3278
3741	Rodentia	Sciuridae	Marmota caligata	LC	2.734	1.3174
3741	Rodentia	Sciuridae	Marmota olympus	LC	2.734	1.3174
3743	Carnivora	Canidae	Pseudalopex culpaeus	LC	2.667	1.2994
3743	Carnivora	Canidae	Pseudalopex griseus	LC	2.667	1.2994
3743	Carnivora	Canidae	Pseudalopex gymnocercus	LC	2.667	1.2994
3746	Rodentia	Muridae	Mus baoulei	LC	2.648	1.2941
3746	Rodentia	Muridae	Mus bufo	LC	2.648	1.2941
3746	Rodentia	Muridae	Mus haussa	LC	2.648	1.2941
3746	Rodentia	Muridae	Mus indutus	LC	2.648	1.2941
3746	Rodentia	Muridae	Mus mahomet	LC	2.648	1.2941
3746	Rodentia	Muridae	Mus mattheyi	LC	2.648	1.2941
3746	Rodentia	Muridae	Mus minutoides	LC	2.648	1.2941
3746	Rodentia	Muridae	Mus musculoides	LC	2.648	1.2941
3746	Rodentia	Muridae	Mus orangiae	LC	2.648	1.2941
3746	Rodentia	Muridae	Mus setulosus	LC	2.648	1.2941
3746	Rodentia	Muridae	Mus setzeri	LC	2.648	1.2941
3746	Rodentia	Muridae	Mus sorella	LC	2.648	1.2941
3746	Rodentia	Muridae	Mus tenellus	LC	2.648	1.2941
3746	Rodentia	Muridae	Mus triton	LC	2.648	1.2941
3760	Rodentia	Muridae	Crunomys melanius	LC	2.566	1.2715
3761	Rodentia	Muridae	Peromyscus grandis	NT	0.732	1.2425
3761	Rodentia	Muridae	Peromyscus guardia	NT	0.732	1.2425
3761	Rodentia	Muridae	Peromyscus simulus	NT	0.732	1.2425
3761	Rodentia	Muridae	Peromyscus winkelmanni	NT	0.732	1.2425
3765	Rodentia	Muridae	Mus fernandoni	LC	2.305	1.1954
3765	Rodentia	Muridae	Mus phillipsi	LC	2.305	1.1954
3765	Rodentia	Muridae	Mus platythrix	LC	2.305	1.1954
3765	Rodentia	Muridae	Mus saxicola	LC	2.305	1.1954
3765	Rodentia	Muridae	Mus shortridgei	LC	2.305	1.1954
3770	Chiroptera	Vespertilionidae	Murina florium	LC	2.286	1.1896
3770	Chiroptera	Vespertilionidae	Murina suilla	LC	2.286	1.1896
3770	Chiroptera	Vespertilionidae	Murina tubinaris	LC	2.286	1.1896
3773	Chiroptera	Vespertilionidae	Lasionycteris noctivagans	LC	2.280	1.1879
3773	Chiroptera	Vespertilionidae	Mimetillus moloneyi	LC	2.280	1.1879
3773	Chiroptera	Vespertilionidae	Nycticeius humeralis	LC	2.280	1.1879
3773	Chiroptera	Vespertilionidae	Otonycteris hemprichii	LC	2.280	1.1879
3773	Chiroptera	Vespertilionidae	Philetor brachypterus	LC	2.280	1.1879
3773	Chiroptera	Vespertilionidae	Pipistrellus dormeri	LC	2.280	1.1879
3773	Chiroptera	Vespertilionidae	Pipistrellus subflavus	LC	2.280	1.1879
3780	Rodentia	Muridae	Bolomys amoenus	LC	2.243	1.1765
3780	Rodentia	Muridae	Bolomys lactens	LC	2.243	1.1765
3780	Rodentia	Muridae	Bolomys lasiurus	LC	2.243	1.1765

Rank	Order	Family	Species	IUCN	ED'	EDGE
3780	Rodentia	Muridae	Bolomys obscurus	LC	2.243	1.1765
3780	Rodentia	Muridae	Bolomys punctulatus	LC	2.243	1.1765
3780	Rodentia	Muridae	Bolomys temchuki	LC	2.243	1.1765
3786	Rodentia	Ctenomyidae	Ctenomys argentinus	LC	2.199	1.1628
3786	Rodentia	Ctenomyidae	Ctenomys australis	LC	2.199	1.1628
3786	Rodentia	Ctenomyidae	Ctenomys azarae	LC	2.199	1.1628
3786	Rodentia	Ctenomyidae	Ctenomys boliviensis	LC	2.199	1.1628
3786	Rodentia	Ctenomyidae	Ctenomys bonettoi	LC	2.199	1.1628
3786	Rodentia	Ctenomyidae	Ctenomys brasiliensis	LC	2.199	1.1628
3786	Rodentia	Ctenomyidae	Ctenomys colburni	LC	2.199	1.1628
3786	Rodentia	Ctenomyidae	Ctenomys conoveri	LC	2.199	1.1628
3786	Rodentia	Ctenomyidae	Ctenomys dorsalis	LC	2.199	1.1628
3786	Rodentia	Ctenomyidae	Ctenomys emilianus	LC	2.199	1.1628
3786	Rodentia	Ctenomyidae	Ctenomys frater	LC	2.199	1.1628
3786	Rodentia	Ctenomyidae	Ctenomys fulvus	LC	2.199	1.1628
3786	Rodentia	Ctenomyidae	Ctenomys haigi	LC	2.199	1.1628
3786	Rodentia	Ctenomyidae	Ctenomys knighti	LC	2.199	1.1628
3786	Rodentia	Ctenomyidae	Ctenomys leucodon	LC	2.199	1.1628
3786	Rodentia	Ctenomyidae	Ctenomys lewisi	LC	2.199	1.1628
3786	Rodentia	Ctenomyidae	Ctenomys maulinus	LC	2.199	1.1628
3786	Rodentia	Ctenomyidae	Ctenomys mendocinus	LC	2.199	1.1628
3786	Rodentia	Ctenomyidae	Ctenomys minutus	LC	2.199	1.1628
3786	Rodentia	Ctenomyidae	Ctenomys occultus	LC	2.199	1.1628
3786	Rodentia	Ctenomyidae	Ctenomys opimus	LC	2.199	1.1628
3786	Rodentia	Ctenomyidae	Ctenomys pearsoni	LC	2.199	1.1628
3786	Rodentia	Ctenomyidae	Ctenomys perrensis	LC	2.199	1.1628
3786	Rodentia	Ctenomyidae	Ctenomys peruanus	LC	2.199	1.1628
3786	Rodentia	Ctenomyidae	Ctenomys pontifex	LC	2.199	1.1628
3786	Rodentia	Ctenomyidae	Ctenomys porteousi	LC	2.199	1.1628
3786	Rodentia	Ctenomyidae	Ctenomys saltarius	LC	2.199	1.1628
3786	Rodentia	Ctenomyidae	Ctenomys sericeus	LC	2.199	1.1628
3786	Rodentia	Ctenomyidae	Ctenomys steinbachi	LC	2.199	1.1628
3786	Rodentia	Ctenomyidae	Ctenomys talarum	LC	2.199	1.1628
3786	Rodentia	Ctenomyidae	Ctenomys torquatus	LC	2.199	1.1628
3786	Rodentia	Ctenomyidae	Ctenomys tuconax	LC	2.199	1.1628
3786	Rodentia	Ctenomyidae	Ctenomys tucumanus	LC	2.199	1.1628
3786	Rodentia	Ctenomyidae	Ctenomys validus	LC	2.199	1.1628
3820	Rodentia	Muridae	Apomys abrae	LC	2.130	1.1411
3820	Rodentia	Muridae	Apomys datae	LC	2.130	1.1411
3820	Rodentia	Muridae	Apomys hylocoetes	LC	2.130	1.1411
3820	Rodentia	Muridae	Apomys insignis	LC	2.130	1.1411
3820	Rodentia	Muridae	Apomys littoralis	LC	2.130	1.1411
3820	Rodentia	Muridae	Apomys microdon	LC	2.130	1.1411
3820	Rodentia	Muridae	Apomys musculus	LC	2.130	1.1411
3827	Rodentia	Muridae	Oligoryzomys andinus	LC	2.123	1.1389
3827	Rodentia	Muridae	Oligoryzomys arenalis	LC	2.123	1.1389
3827	Rodentia	Muridae	Oligoryzomys chacoensis	LC	2.123	1.1389
3827	Rodentia	Muridae	Oligoryzomys delticola	LC	2.123	1.1389
3827	Rodentia	Muridae	Oligoryzomys eliurus	LC	2.123	1.1389
3827	Rodentia	Muridae	Oligoryzomys flavescens	LC	2.123	1.1389
3827	Rodentia	Muridae	Oligoryzomys fulvescens	LC	2.123	1.1389
3827	Rodentia	Muridae	Oligoryzomys griseolus	LC	2.123	1.1389
3827	Rodentia	Muridae	Oligoryzomys longicaudatus	LC	2.123	1.1389
3827	Rodentia	Muridae	Oligoryzomys magellanicus	LC	2.123	1.1389
3827	Rodentia	Muridae	Oligoryzomys microtis	LC	2.123	1.1389
3827	Rodentia	Muridae	Oligoryzomys nigripes	LC	2.123	1.1389

Rank	Order	Family	Species	IUCN	ED'	EDGE
3839	Rodentia	Muridae	Oecomys bicolor	LC	2.086	1.1268
3839	Rodentia	Muridae	Oecomys concolor	LC	2.086	1.1268
3839	Rodentia	Muridae	Oecomys flavicans	LC	2.086	1.1268
3839	Rodentia	Muridae	Oecomys mamorae	LC	2.086	1.1268
3839	Rodentia	Muridae	Oecomys paricola	LC	2.086	1.1268
3839	Rodentia	Muridae	Oecomys phaeotis	LC	2.086	1.1268
3839	Rodentia	Muridae	Oecomys rex	LC	2.086	1.1268
3839	Rodentia	Muridae	Oecomys roberti	LC	2.086	1.1268
3839	Rodentia	Muridae	Oecomys rutilus	LC	2.086	1.1268
3839	Rodentia	Muridae	Oecomys speciosus	LC	2.086	1.1268
3839	Rodentia	Muridae	Oecomys superans	LC	2.086	1.1268
3839	Rodentia	Muridae	Oecomys trinitatis	LC	2.086	1.1268
3851	Rodentia	Muridae	Thomasomys aureus	LC	2.005	1.1002
3851	Rodentia	Muridae	Thomasomys baeops	LC	2.005	1.1002
3851	Rodentia	Muridae	Thomasomys bombycinus	LC	2.005	1.1002
3851	Rodentia	Muridae	Thomasomys cinereiventer	LC	2.005	1.1002
3851	Rodentia	Muridae	Thomasomys cinereus	LC	2.005	1.1002
3851	Rodentia	Muridae	Thomasomys daphne	LC	2.005	1.1002
3851	Rodentia	Muridae	Thomasomys eleusis	LC	2.005	1.1002
3851	Rodentia	Muridae	Thomasomys gracilis	LC	2.005	1.1002
3851	Rodentia	Muridae	Thomasomys hylophilus	LC	2.005	1.1002
3851	Rodentia	Muridae	Thomasomys incanus	LC	2.005	1.1002
3851	Rodentia	Muridae	Thomasomys ischyurus	LC	2.005	1.1002
3851	Rodentia	Muridae	Thomasomys kalinowskii	LC	2.005	1.1002
3851	Rodentia	Muridae	Thomasomys ladewi	LC	2.005	1.1002
3851	Rodentia	Muridae	Thomasomys laniger	LC	2.005	1.1002
3851	Rodentia	Muridae	Thomasomys niveipes	LC	2.005	1.1002
3851	Rodentia	Muridae	Thomasomys oreas	LC	2.005	1.1002
3851	Rodentia	Muridae	Thomasomys paramorum	LC	2.005	1.1002
3851	Rodentia	Muridae	Thomasomys pyrrhonotus	LC	2.005	1.1002
3851	Rodentia	Muridae	Thomasomys rhoadsi	LC	2.005	1.1002
3851	Rodentia	Muridae	Thomasomys rosalinda	LC	2.005	1.1002
3851	Rodentia	Muridae	Thomasomys silvestris	LC	2.005	1.1002
3851	Rodentia	Muridae	Thomasomys taczanowskii	LC	2.005	1.1002
3851	Rodentia	Muridae	Thomasomys vestitus	LC	2.005	1.1002
3874	Chiroptera	Pteropodidae	Pteropus anetianus	LC	1.990	1.0954
3875	Rodentia	Muridae	Chrotomys mindorensis	LC	1.974	1.0898
3876	Rodentia	Echimyidae	Euryzygomatomys spinosus	LC	1.912	1.0690
3876	Rodentia	Echimyidae	Hoplomys gymnurus	LC	1.912	1.0690
3876	Rodentia	Echimyidae	Lonchothrix emiliae	LC	1.912	1.0690
3876	Rodentia	Echimyidae	Proechimys amphichoricus	LC	1.912	1.0690
3876	Rodentia	Echimyidae	Proechimys bolivianus	LC	1.912	1.0690
3876	Rodentia	Echimyidae	Proechimys brevicauda	LC	1.912	1.0690
3876	Rodentia	Echimyidae	Proechimys canicollis	LC	1.912	1.0690
3876	Rodentia	Echimyidae	Proechimys cayennensis	LC	1.912	1.0690
3876	Rodentia	Echimyidae	Proechimys chrysaeolus	LC	1.912	1.0690
3876	Rodentia	Echimyidae	Proechimys cuvieri	LC	1.912	1.0690
3876	Rodentia	Echimyidae	Proechimys decumanus	LC	1.912	1.0690
3876	Rodentia	Echimyidae	Proechimys dimidiatus	LC	1.912	1.0690
3876	Rodentia	Echimyidae	Proechimys goeldii	LC	1.912	1.0690
3876	Rodentia	Echimyidae	Proechimys guairae	LC	1.912	1.0690
3876	Rodentia	Echimyidae	Proechimys gularis	LC	1.912	1.0690
3876	Rodentia	Echimyidae	Proechimys hendeei	LC	1.912	1.0690
3876	Rodentia	Echimyidae	Proechimys hoplomyoides	LC	1.912	1.0690
3876	Rodentia	Echimyidae	Proechimys iheringi	LC	1.912	1.0690
3876	Rodentia	Echimyidae	Proechimys longicaudatus	LC	1.912	1.0690

Rank	Order	Family	Species	IUCN	ED'	EDGE
3876	Rodentia	Echimyidae	Proechimys magdalenae	LC	1.912	1.0690
3876	Rodentia	Echimyidae	Proechimys mincae	LC	1.912	1.0690
3876	Rodentia	Echimyidae	Proechimys myosuros	LC	1.912	1.0690
3876	Rodentia	Echimyidae	Proechimys oconnelli	LC	1.912	1.0690
3876	Rodentia	Echimyidae	Proechimys oris	LC	1.912	1.0690
3876	Rodentia	Echimyidae	Proechimys poliopus	LC	1.912	1.0690
3876	Rodentia	Echimyidae	Proechimys quadruplicatus	LC	1.912	1.0690
3876	Rodentia	Echimyidae	Proechimys semispinosus	LC	1.912	1.0690
3876	Rodentia	Echimyidae	Proechimys setosus	LC	1.912	1.0690
3876	Rodentia	Echimyidae	Proechimys simonsi	LC	1.912	1.0690
3876	Rodentia	Echimyidae	Proechimys steerei	LC	1.912	1.0690
3876	Rodentia	Echimyidae	Proechimys trinitatis	LC	1.912	1.0690
3876	Rodentia	Echimyidae	Proechimys urichi	LC	1.912	1.0690
3876	Rodentia	Echimyidae	Proechimys warreni	LC	1.912	1.0690
3876	Rodentia	Echimyidae	Thrichomys apereoides	LC	1.912	1.0690
3910	Chiroptera	Rhinolophidae	Rhinolophus landeri	LC	1.901	1.0650
3911	Rodentia	Muridae	Rattus argentiventer	LC	1.865	1.0524
3911	Rodentia	Muridae	Rattus everetti	LC	1.865	1.0524
3911	Rodentia	Muridae	Rattus hoffmanni	LC	1.865	1.0524
3911	Rodentia	Muridae	Rattus losea	LC	1.865	1.0524
3911	Rodentia	Muridae	Rattus lugens	LC	1.865	1.0524
3911	Rodentia	Muridae	Rattus nitidus	LC	1.865	1.0524
3911	Rodentia	Muridae	Rattus osgoodi	LC	1.865	1.0524
3911	Rodentia	Muridae	Rattus rattus	LC	1.865	1.0524
3911	Rodentia	Muridae	Rattus simalurensis	LC	1.865	1.0524
3911	Rodentia	Muridae	Rattus tanezumi	LC	1.865	1.0524
3911	Rodentia	Muridae	Rattus tiomanicus	LC	1.865	1.0524
3911	Rodentia	Muridae	Rattus turkestanicus	LC	1.865	1.0524
3923	Rodentia	Muridae	Akodon affinis	LC	1.838	1.0429
3923	Rodentia	Muridae	Akodon albiventer	LC	1.838	1.0429
3923	Rodentia	Muridae	Akodon azarae	LC	1.838	1.0429
3923	Rodentia	Muridae	Akodon cursor	LC	1.838	1.0429
3923	Rodentia	Muridae	Akodon fumeus	LC	1.838	1.0429
3923	Rodentia	Muridae	Akodon iniscatus	LC	1.838	1.0429
3923	Rodentia	Muridae	Akodon kofordi	LC	1.838	1.0429
3923	Rodentia	Muridae	Akodon markhami	LC	1.838	1.0429
3923	Rodentia	Muridae	Akodon mollis	LC	1.838	1.0429
3923	Rodentia	Muridae	Akodon olivaceus	LC	1.838	1.0429
3923	Rodentia	Muridae	Akodon orophilus	LC	1.838	1.0429
3923	Rodentia	Muridae	Akodon puer	LC	1.838	1.0429
3923	Rodentia	Muridae	Akodon serrensis	LC	1.838	1.0429
3923	Rodentia	Muridae	Akodon spegazzinii	LC	1.838	1.0429
3923	Rodentia	Muridae	Akodon subfuscus	LC	1.838	1.0429
3923	Rodentia	Muridae	Akodon surdus	LC	1.838	1.0429
3923	Rodentia	Muridae	Akodon sylvanus	LC	1.838	1.0429
3923	Rodentia	Muridae	Akodon torques	LC	1.838	1.0429
3923	Rodentia	Muridae	Akodon urichi	LC	1.838	1.0429
3942	Insectivora	Soricidae	Crocidura baileyi	NT	0.320	0.9707
3942	Insectivora	Soricidae	Crocidura grandiceps	NT	0.320	0.9707
3944	Rodentia	Muridae	Holochilus brasiliensis	LC	1.625	0.9652
3944	Rodentia	Muridae	Holochilus chacarius	LC	1.625	0.9652
3944	Rodentia	Muridae	Holochilus magnus	LC	1.625	0.9652
3944	Rodentia	Muridae	Holochilus sciureus	LC	1.625	0.9652
3948	Chiroptera	Rhinolophidae	Rhinolophus borneensis	LC	1.600	0.9555
3949	Rodentia	Muridae	Andinomys edax	LC	1.435	0.8901
3949	Rodentia	Muridae	Chilomys instans	LC	1.435	0.8901

Rank	Order	Family	Species	IUCN	ED'	EDGE
3949	Rodentia	Muridae	Chinchillula sahamae	LC	1.435	0.8901
3949	Rodentia	Muridae	Galenomys garleppi	LC	1.435	0.8901
3949	Rodentia	Muridae	Geoxus valdivianus	LC	1.435	0.8901
3949	Rodentia	Muridae	Irenomys tarsalis	LC	1.435	0.8901
3949	Rodentia	Muridae	Neotomys ebriosus	LC	1.435	0.8901
3949	Rodentia	Muridae	Notiomys edwardsii	LC	1.435	0.8901
3949	Rodentia	Muridae	Punomys lemminus	LC	1.435	0.8901
3949	Rodentia	Muridae	Reithrodon auritus	LC	1.435	0.8901
3949	Rodentia	Muridae	Wiedomys pyrrhorhinos	LC	1.435	0.8901
3960	Rodentia	Muridae	Akodon dayi	LC	1.416	0.8821
3960	Rodentia	Muridae	Akodon dolores	LC	1.416	0.8821
3960	Rodentia	Muridae	Akodon molinae	LC	1.416	0.8821
3960	Rodentia	Muridae	Akodon neocenus	LC	1.416	0.8821
3960	Rodentia	Muridae	Akodon simulator	LC	1.416	0.8821
3960	Rodentia	Muridae	Akodon toba	LC	1.416	0.8821
3960	Rodentia	Muridae	Akodon varius	LC	1.416	0.8821
3967	Rodentia	Muridae	Oryzomys albigularis	LC	1.263	0.8165
3967	Rodentia	Muridae	Oryzomys alfaroi	LC	1.263	0.8165
3967	Rodentia	Muridae	Oryzomys auriventer	LC	1.263	0.8165
3967	Rodentia	Muridae	Oryzomys balneator	LC	1.263	0.8165
3967	Rodentia	Muridae	Oryzomys bolivaris	LC	1.263	0.8165
3967	Rodentia	Muridae	Oryzomys buccinatus	LC	1.263	0.8165
3967	Rodentia	Muridae	Oryzomys capito	LC	1.263	0.8165
3967	Rodentia	Muridae	Oryzomys chapmani	LC	1.263	0.8165
3967	Rodentia	Muridae	Oryzomys couesi	LC	1.263	0.8165
3967	Rodentia	Muridae	Oryzomys devius	LC	1.263	0.8165
3967	Rodentia	Muridae	Oryzomys hammondi	LC	1.263	0.8165
3967	Rodentia	Muridae	Oryzomys intectus	LC	1.263	0.8165
3967	Rodentia	Muridae	Oryzomys intermedius	LC	1.263	0.8165
3967	Rodentia	Muridae	Oryzomys keaysi	LC	1.263	0.8165
3967	Rodentia	Muridae	Oryzomys kelloggi	LC	1.263	0.8165
3967	Rodentia	Muridae	Oryzomys lamia	LC	1.263	0.8165
3967	Rodentia	Muridae	Oryzomys legatus	LC	1.263	0.8165
3967	Rodentia	Muridae	Oryzomys macconnelli	LC	1.263	0.8165
3967	Rodentia	Muridae	Oryzomys nitidus	LC	1.263	0.8165
3967	Rodentia	Muridae	Oryzomys palustris	LC	1.263	0.8165
3967	Rodentia	Muridae	Oryzomys polius	LC	1.263	0.8165
3967	Rodentia	Muridae	Oryzomys ratticeps	LC	1.263	0.8165
3967	Rodentia	Muridae	Oryzomys rhabdops	LC	1.263	0.8165
3967	Rodentia	Muridae	Oryzomys rostratus	LC	1.263	0.8165
3967	Rodentia	Muridae	Oryzomys saturatior	LC	1.263	0.8165
3967	Rodentia	Muridae	Oryzomys subflavus	LC	1.263	0.8165
3967	Rodentia	Muridae	Oryzomys talamancae	LC	1.263	0.8165
3967	Rodentia	Muridae	Oryzomys xantheolus	LC	1.263	0.8165
3967	Rodentia	Muridae	Oryzomys yunganus	LC	1.263	0.8165
3996	Rodentia	Muridae	Gerbillus hoogstraali	NT	0.111	0.7988
3996	Rodentia	Muridae	Gerbillus poecilops	NT	0.111	0.7988
3996	Rodentia	Muridae	Gerbillus riggenbachi	NT	0.111	0.7988
3999	Rodentia	Muridae	Diomys crumpi	NT	0.058	0.7497
3999	Rodentia	Muridae	Kadarsanomys sodyi	NT	0.058	0.7497
3999	Rodentia	Muridae	Micromys minutus	NT	0.058	0.7497
3999	Rodentia	Muridae	Srilankamys ohiensis	NT	0.058	0.7497
4003	Chiroptera	Rhinolophidae	Rhinolophus acuminatus	LC	1.062	0.7239
4003	Chiroptera	Rhinolophidae	Rhinolophus affinis	LC	1.062	0.7239
4003	Chiroptera	Rhinolophidae	Rhinolophus alcyone	LC	1.062	0.7239
4003	Chiroptera	Rhinolophidae	Rhinolophus arcuatus	LC	1.062	0.7239

Rank	Order	Family	Species	IUCN	ED'	EDGE
4003	Chiroptera	Rhinolophidae	Rhinolophus clivosus	LC	1.062	0.7239
4003	Chiroptera	Rhinolophidae	Rhinolophus coelophyllus	LC	1.062	0.7239
4003	Chiroptera	Rhinolophidae	Rhinolophus darlingi	LC	1.062	0.7239
4003	Chiroptera	Rhinolophidae	Rhinolophus euryotis	LC	1.062	0.7239
4003	Chiroptera	Rhinolophidae	Rhinolophus fumigatus	LC	1.062	0.7239
4003	Chiroptera	Rhinolophidae	Rhinolophus hildebrandti	LC	1.062	0.7239
4003	Chiroptera	Rhinolophidae	Rhinolophus hipposideros	LC	1.062	0.7239
4003	Chiroptera	Rhinolophidae	Rhinolophus luctus	LC	1.062	0.7239
4003	Chiroptera	Rhinolophidae	Rhinolophus macrotis	LC	1.062	0.7239
4003	Chiroptera	Rhinolophidae	Rhinolophus malayanus	LC	1.062	0.7239
4003	Chiroptera	Rhinolophidae	Rhinolophus megaphyllus	LC	1.062	0.7239
4003	Chiroptera	Rhinolophidae	Rhinolophus pearsonii	LC	1.062	0.7239
4003	Chiroptera	Rhinolophidae	Rhinolophus rouxii	LC	1.062	0.7239
4003	Chiroptera	Rhinolophidae	Rhinolophus sedulus	LC	1.062	0.7239
4003	Chiroptera	Rhinolophidae	Rhinolophus simulator	LC	1.062	0.7239
4003	Chiroptera	Rhinolophidae	Rhinolophus stheno	LC	1.062	0.7239
4003	Chiroptera	Rhinolophidae	Rhinolophus trifoliatus	LC	1.062	0.7239
4024	Rodentia	Muridae	Peromyscus attwateri	LC	0.732	0.5493
4024	Rodentia	Muridae	Peromyscus aztecus	LC	0.732	0.5493
4024	Rodentia	Muridae	Peromyscus boylii	LC	0.732	0.5493
4024	Rodentia	Muridae	Peromyscus californicus	LC	0.732	0.5493
4024	Rodentia	Muridae	Peromyscus crinitus	LC	0.732	0.5493
4024	Rodentia	Muridae	Peromyscus difficilis	LC	0.732	0.5493
4024	Rodentia	Muridae	Peromyscus eremicus	LC	0.732	0.5493
4024	Rodentia	Muridae	Peromyscus eva	LC	0.732	0.5493
4024	Rodentia	Muridae	Peromyscus furvus	LC	0.732	0.5493
4024	Rodentia	Muridae	Peromyscus gossypinus	LC	0.732	0.5493
4024	Rodentia	Muridae	Peromyscus gratus	LC	0.732	0.5493
4024	Rodentia	Muridae	Peromyscus guatemalensis	LC	0.732	0.5493
4024	Rodentia	Muridae	Peromyscus gymnotis	LC	0.732	0.5493
4024	Rodentia	Muridae	Peromyscus hooperi	LC	0.732	0.5493
4024	Rodentia	Muridae	Peromyscus leucopus	LC	0.732	0.5493
4024	Rodentia	Muridae	Peromyscus levipes	LC	0.732	0.5493
4024	Rodentia	Muridae	Peromyscus maniculatus	LC	0.732	0.5493
4024	Rodentia	Muridae	Peromyscus megalops	LC	0.732	0.5493
4024	Rodentia	Muridae	Peromyscus melanocarpus	LC	0.732	0.5493
4024	Rodentia	Muridae	Peromyscus melanophrys	LC	0.732	0.5493
4024	Rodentia	Muridae	Peromyscus melanotis	LC	0.732	0.5493
4024	Rodentia	Muridae	Peromyscus merriami	LC	0.732	0.5493
4024	Rodentia	Muridae	Peromyscus mexicanus	LC	0.732	0.5493
4024	Rodentia	Muridae	Peromyscus nasutus	LC	0.732	0.5493
4024	Rodentia	Muridae	Peromyscus ochraventer	LC	0.732	0.5493
4024	Rodentia	Muridae	Peromyscus oreas	LC	0.732	0.5493
4024	Rodentia	Muridae	Peromyscus pectoralis	LC	0.732	0.5493
4024	Rodentia	Muridae	Peromyscus perfulvus	LC	0.732	0.5493
4024	Rodentia	Muridae	Peromyscus polionotus	LC	0.732	0.5493
4024	Rodentia	Muridae	Peromyscus sejugis	LC	0.732	0.5493
4024	Rodentia	Muridae	Peromyscus sitkensis	LC	0.732	0.5493
4024	Rodentia	Muridae	Peromyscus spicilegus	LC	0.732	0.5493
4024	Rodentia	Muridae	Peromyscus stirtoni	LC	0.732	0.5493
4024	Rodentia	Muridae	Peromyscus truei	LC	0.732	0.5493
4024	Rodentia	Muridae	Peromyscus yucatanicus	LC	0.732	0.5493
4059	Insectivora	Soricidae	Crocidura aleksandrisi	LC	0.320	0.2776
4059	Insectivora	Soricidae	Crocidura allex	LC	0.320	0.2776
4059	Insectivora	Soricidae	Crocidura arabica	LC	0.320	0.2776
4059	Insectivora	Soricidae	Crocidura attenuata	LC	0.320	0.2776

Rank	Order	Family	Species	IUCN	ED'	EDGE
4059	Insectivora	Soricidae	Crocidura attila	LC	0.320	0.2776
4059	Insectivora	Soricidae	Crocidura batesi	LC	0.320	0.2776
4059	Insectivora	Soricidae	Crocidura buettikoferi	LC	0.320	0.2776
4059	Insectivora	Soricidae	Crocidura caliginea	LC	0.320	0.2776
4059	Insectivora	Soricidae	Crocidura cinderella	LC	0.320	0.2776
4059	Insectivora	Soricidae	Crocidura congobelgica	LC	0.320	0.2776
4059	Insectivora	Soricidae	Crocidura cossyrensis	LC	0.320	0.2776
4059	Insectivora	Soricidae	Crocidura crenata	LC	0.320	0.2776
4059	Insectivora	Soricidae	Crocidura crossei	LC	0.320	0.2776
4059	Insectivora	Soricidae	Crocidura cyanea	LC	0.320	0.2776
4059	Insectivora	Soricidae	Crocidura denti	LC	0.320	0.2776
4059	Insectivora	Soricidae	Crocidura dolichura	LC	0.320	0.2776
4059	Insectivora	Soricidae	Crocidura dsinezumi	LC	0.320	0.2776
4059	Insectivora	Soricidae	Crocidura elgonius	LC	0.320	0.2776
4059	Insectivora	Soricidae	Crocidura elongata	LC	0.320	0.2776
4059	Insectivora	Soricidae	Crocidura flavescens	LC	0.320	0.2776
4059	Insectivora	Soricidae	Crocidura foxi	LC	0.320	0.2776
4059	Insectivora	Soricidae	Crocidura fuliginosa	LC	0.320	0.2776
4059	Insectivora	Soricidae	Crocidura fulvastra	LC	0.320	0.2776
4059	Insectivora	Soricidae	Crocidura fumosa	LC	0.320	0.2776
4059	Insectivora	Soricidae	Crocidura fuscomurina	LC	0.320	0.2776
4059	Insectivora	Soricidae	Crocidura goliath	LC	0.320	0.2776
4059	Insectivora	Soricidae	Crocidura grassei	LC	0.320	0.2776
4059	Insectivora	Soricidae	Crocidura greenwoodi	LC	0.320	0.2776
4059	Insectivora	Soricidae	Crocidura gueldenstaedtii	LC	0.320	0.2776
4059	Insectivora	Soricidae	Crocidura hildegardeae	LC	0.320	0.2776
4059	Insectivora	Soricidae	Crocidura hirta	LC	0.320	0.2776
4059	Insectivora	Soricidae	Crocidura horsfieldii	LC	0.320	0.2776
4059	Insectivora	Soricidae	Crocidura jacksoni	LC	0.320	0.2776
4059	Insectivora	Soricidae	Crocidura lamottei	LC	0.320	0.2776
4059	Insectivora	Soricidae	Crocidura lanosa	LC	0.320	0.2776
4059	Insectivora	Soricidae	Crocidura lasiura	LC	0.320	0.2776
4059	Insectivora	Soricidae	Crocidura latona	LC	0.320	0.2776
4059	Insectivora	Soricidae	Crocidura lea	LC	0.320	0.2776
4059	Insectivora	Soricidae	Crocidura leucodon	LC	0.320	0.2776
4059	Insectivora	Soricidae	Crocidura levicula	LC	0.320	0.2776
4059	Insectivora	Soricidae	Crocidura littoralis	LC	0.320	0.2776
4059	Insectivora	Soricidae	Crocidura ludia	LC	0.320	0.2776
4059	Insectivora	Soricidae	Crocidura luna	LC	0.320	0.2776
4059	Insectivora	Soricidae	Crocidura lusitania	LC	0.320	0.2776
4059	Insectivora	Soricidae	Crocidura macarthuri	LC	0.320	0.2776
4059	Insectivora	Soricidae	Crocidura maquassiensis	LC	0.320	0.2776
4059	Insectivora	Soricidae	Crocidura mariquensis	LC	0.320	0.2776
4059	Insectivora	Soricidae	Crocidura maxi	LC	0.320	0.2776
4059	Insectivora	Soricidae	Crocidura monticola	LC	0.320	0.2776
4059	Insectivora	Soricidae	Crocidura montis	LC	0.320	0.2776
4059	Insectivora	Soricidae	Crocidura muricauda	LC	0.320	0.2776
4059	Insectivora	Soricidae	Crocidura nanilla	LC	0.320	0.2776
4059	Insectivora	Soricidae	Crocidura neglecta	LC	0.320	0.2776
4059	Insectivora	Soricidae	Crocidura nigeriae	LC	0.320	0.2776
4059	Insectivora	Soricidae	Crocidura nigricans	LC	0.320	0.2776
4059	Insectivora	Soricidae	Crocidura nigripes	LC	0.320	0.2776
4059	Insectivora	Soricidae	Crocidura nigrofusca	LC	0.320	0.2776
4059	Insectivora	Soricidae	Crocidura niobe	LC	0.320	0.2776
4059	Insectivora	Soricidae	Crocidura obscurior	LC	0.320	0.2776
4059	Insectivora	Soricidae	Crocidura olivieri	LC	0.320	0.2776

Rank	Order	Family	Species	IUCN	ED'	EDGE
4059	Insectivora	Soricidae	Crocidura parvipes	LC	0.320	0.2776
4059	Insectivora	Soricidae	Crocidura pasha	LC	0.320	0.2776
4059	Insectivora	Soricidae	Crocidura poensis	LC	0.320	0.2776
4059	Insectivora	Soricidae	Crocidura pullata	LC	0.320	0.2776
4059	Insectivora	Soricidae	Crocidura rhoditis	LC	0.320	0.2776
4059	Insectivora	Soricidae	Crocidura roosevelti	LC	0.320	0.2776
4059	Insectivora	Soricidae	Crocidura russula	LC	0.320	0.2776
4059	Insectivora	Soricidae	Crocidura selina	LC	0.320	0.2776
4059	Insectivora	Soricidae	Crocidura serezkyensis	LC	0.320	0.2776
4059	Insectivora	Soricidae	Crocidura sibirica	LC	0.320	0.2776
4059	Insectivora	Soricidae	Crocidura sicula	LC	0.320	0.2776
4059	Insectivora	Soricidae	Crocidura silacea	LC	0.320	0.2776
4059	Insectivora	Soricidae	Crocidura smithii	LC	0.320	0.2776
4059	Insectivora	Soricidae	Crocidura somalica	LC	0.320	0.2776
4059	Insectivora	Soricidae	Crocidura suaveolens	LC	0.320	0.2776
4059	Insectivora	Soricidae	Crocidura thalia	LC	0.320	0.2776
4059	Insectivora	Soricidae	Crocidura theresae	LC	0.320	0.2776
4059	Insectivora	Soricidae	Crocidura turba	LC	0.320	0.2776
4059	Insectivora	Soricidae	Crocidura viaria	LC	0.320	0.2776
4059	Insectivora	Soricidae	Crocidura voi	LC	0.320	0.2776
4059	Insectivora	Soricidae	Crocidura whitakeri	LC	0.320	0.2776
4059	Insectivora	Soricidae	Crocidura xantippe	LC	0.320	0.2776
4059	Insectivora	Soricidae	Crocidura yankariensis	LC	0.320	0.2776
4059	Insectivora	Soricidae	Crocidura zarudnyi	LC	0.320	0.2776
4143	Rodentia	Muridae	Gerbillus andersoni	LC	0.111	0.1057
4143	Rodentia	Muridae	Gerbillus aquilus	LC	0.111	0.1057
4143	Rodentia	Muridae	Gerbillus campestris	LC	0.111	0.1057
4143	Rodentia	Muridae	Gerbillus cheesmani	LC	0.111	0.1057
4143	Rodentia	Muridae	Gerbillus dasyurus	LC	0.111	0.1057
4143	Rodentia	Muridae	Gerbillus diminutus	LC	0.111	0.1057
4143	Rodentia	Muridae	Gerbillus famulus	LC	0.111	0.1057
4143	Rodentia	Muridae	Gerbillus floweri	LC	0.111	0.1057
4143	Rodentia	Muridae	Gerbillus garamantis	LC	0.111	0.1057
4143	Rodentia	Muridae	Gerbillus gerbillus	LC	0.111	0.1057
4143	Rodentia	Muridae	Gerbillus gleadowi	LC	0.111	0.1057
4143	Rodentia	Muridae	Gerbillus harwoodi	LC	0.111	0.1057
4143	Rodentia	Muridae	Gerbillus henleyi	LC	0.111	0.1057
4143	Rodentia	Muridae	Gerbillus juliani	LC	0.111	0.1057
4143	Rodentia	Muridae	Gerbillus mackillingini	LC	0.111	0.1057
4143	Rodentia	Muridae	Gerbillus maghrebi	LC	0.111	0.1057
4143	Rodentia	Muridae	Gerbillus mesopotamiae	LC	0.111	0.1057
4143	Rodentia	Muridae	Gerbillus muriculus	LC	0.111	0.1057
4143	Rodentia	Muridae	Gerbillus nanus	LC	0.111	0.1057
4143	Rodentia	Muridae	Gerbillus nigeriae	LC	0.111	0.1057
4143	Rodentia	Muridae	Gerbillus percivali	LC	0.111	0.1057
4143	Rodentia	Muridae	Gerbillus perpallidus	LC	0.111	0.1057
4143	Rodentia	Muridae	Gerbillus pulvinatus	LC	0.111	0.1057
4143	Rodentia	Muridae	Gerbillus pusillus	LC	0.111	0.1057
4143	Rodentia	Muridae	Gerbillus pyramidum	LC	0.111	0.1057
4143	Rodentia	Muridae	Gerbillus ruberrimus	LC	0.111	0.1057
4143	Rodentia	Muridae	Gerbillus simoni	LC	0.111	0.1057
4143	Rodentia	Muridae	Gerbillus tarabuli	LC	0.111	0.1057
4143	Rodentia	Muridae	Gerbillus vivax	LC	0.111	0.1057
4143	Rodentia	Muridae	Gerbillus watersi	LC	0.111	0.1057
4173	Rodentia	Muridae	Chiromyscus chiropus	LC	0.058	0.0565
4173	Rodentia	Muridae	Colomys goslingi	LC	0.058	0.0565

Rank	Order	Family	Species	IUCN	ED'	EDGE
4173	Rodentia	Muridae	Dacnomys millardi	LC	0.058	0.0565
4173	Rodentia	Muridae	Desmomys harringtoni	LC	0.058	0.0565
4173	Rodentia	Muridae	Golunda ellioti	LC	0.058	0.0565
4173	Rodentia	Muridae	Hadromys humei	LC	0.058	0.0565
4173	Rodentia	Muridae	Heimyscus fumosus	LC	0.058	0.0565
4173	Rodentia	Muridae	Lenothrix canus	LC	0.058	0.0565
4173	Rodentia	Muridae	Rhabdomys pumilio	LC	0.058	0.0565
4173	Rodentia	Muridae	Stochomys longicaudatus	LC	0.058	0.0565
	Cetacea	Platanistidae	Pontoporia blainvillei	DD	36.295	
	Rodentia	Myoxidae	Graphiurus crassicaudatus	DD	31.043	
	Rodentia	Ctenodactylidae	Felovia vae	DD	30.328	
	Rodentia	Anomaluridae	Zenkerella insignis	DD	30.017	
	Insectivora	Tenrecidae	Oryzorictes tetradactylus	DD	28.524	
	Rodentia	Anomaluridae	Idiurus zenkeri	DD	26.977	
	Rodentia	Sciuridae	Myosciurus pumilio	DD	26.436	
	Diprotodontia	Phalangeridae	Ailurops ursinus	DD	26.373	
	Artiodactyla	Tragulidae	Hyemoschus aquaticus	DD	22.942	
	Primates	Tarsiidae	Tarsius pumilus	DD	21.234	
	Primates	Tarsiidae	Tarsius syrichta	DD	21.234	
	Cetacea	Monodontidae	Monodon monoceros	DD	21.041	
	Rodentia	Muridae	Leimacomys buettneri	DD	19.581	
	Rodentia	Ctenodactylidae	Pectinator spekei	DD	19.431	
	Cetacea	Balaenopteridae	Balaenoptera edeni	DD	18.918	
	Xenarthra	Dasypodidae	Cabassous centralis	DD	18.467	
	Rodentia	Ctenodactylidae	Ctenodactylus vali	DD	18.301	
	Dasyuromorphia	Dasyuridae	Sminthopsis laniger	DD	18.235	
	Insectivora	Chrysochloridae	Chrysochloris visagiei	DD	17.992	
	Carnivora	Ursidae	Helarctos malayanus	DD	17.834	
	Didelphimorphia	Didelphidae	Marmosa canescens	DD	17.618	
	Didelphimorphia	Didelphidae	Marmosa tyleriana	DD	17.618	
	Artiodactyla	Cervidae	Elaphodus cephalophus	DD	17.590	
	Insectivora	Chrysochloridae	Chlorotalpa leucorhina	DD	17.363	
	Insectivora	Chrysochloridae	Chlorotalpa tytonis	DD	17.363	
	Chiroptera	Pteropodidae	Plerotes anchietai	DD	17.330	
	Chiroptera	Rhinolophidae	Coelops hirsutus	DD	17.151	
	Chiroptera	Rhinolophidae	Rhinolophus osgoodi	DD	15.665	
	Carnivora	Viverridae	Osbornictis piscivora	DD	15.530	
	Macroscelidea	Macroscelididae	Elephantulus revoili	DD	15.434	
	Macroscelidea	Macroscelididae	Elephantulus fuscipes	DD	15.204	
	Macroscelidea	Macroscelididae	Elephantulus fuscus	DD	15.204	
	Chiroptera	Molossidae	Tadarida lobata	DD	15.189	
	Rodentia	Dasyproctidae	Myoprocta exilis	DD	15.054	
	Diprotodontia	Pseudocheiridae	Pseudocheirus canescens	DD	14.575	
	Rodentia	Dipodidae	Salpingotus thomasi	DD	13.662	
	Chiroptera	Vespertilionidae	Scotomanes emarginatus	DD	13.306	
	Dasyuromorphia	Dasyuridae	Sminthopsis archeri	DD	13.200	
	Rodentia	Muridae	Ammodillus imbellis	DD	13.063	
	Rodentia	Dipodidae	Allactaga tetradactyla	DD	12.964	
	Diprotodontia	Phalangeridae	Strigocuscus celebensis	DD	12.910	
	Diprotodontia	Phalangeridae	Strigocuscus gymnotis	DD	12.910	
	Rodentia	Muridae	Dendroprionomys rousseloti	DD	12.768	
	Rodentia	Muridae	Megadendromus nikolausi	DD	12.768	
	Rodentia	Muridae	Prionomys batesi	DD	12.768	
	Rodentia	Sciuridae	Epixerus ebii	DD	12.643	
	Rodentia	Sciuridae	Epixerus wilsoni	DD	12.643	
	Rodentia	Chinchillidae	Lagidium viscacia	DD	12.499	

Rank	Order	Family	Species	IUCN	ED'	EDGE
	Carnivora	Procyonidae	Nasuella olivacea	DD	12.368	
	Diprotodontia	Pseudocheiridae	Pseudocheirus caroli	DD	12.354	
	Rodentia	Muridae	Psammomys vexillaris	DD	11.961	
	Chiroptera	Vespertilionidae	Plecotus teneriffae	DD	11.846	
	Rodentia	Dipodidae	Sicista kazbegica	DD	11.769	
	Rodentia	Dipodidae	Sicista kluchorica	DD	11.769	
	Rodentia	Dipodidae	Sicista pseudonapaea	DD	11.769	
	Cetacea	Delphinidae	Feresa attenuata	DD	11.754	
	Cetacea	Phocoenidae	Phocoena spinipinnis	DD	11.641	
	Chiroptera	Vespertilionidae	Pipistrellus permixtus	DD	11.522	
	Chiroptera	Molossidae	Mops niangarae	DD	11.496	
	Diprotodontia	Macropodidae	Dendrolagus inustus	DD	11.313	
	Lagomorpha	Ochotonidae	Ochotona gaoligongensis	DD	11.255	
	Diprotodontia	Macropodidae	Dendrolagus ursinus	DD	11.192	
	Lagomorpha	Ochotonidae	Ochotona muliensis	DD	11.101	
	Lagomorpha	Leporidae	Lepus fagani	DD	11.001	
	Peramelemorphia	Peroryctidae	Microperoryctes murina	DD	10.887	
	Peramelemorphia	Peroryctidae	Microperoryctes papuensis	DD	10.887	
	Peramelemorphia	Peroryctidae	Rhynchomeles prattorum	DD	10.887	
	Peramelemorphia	Peroryctidae	Peroryctes broadbenti	DD	10.850	
	Cetacea	Delphinidae	Cephalorhynchus heavisidii	DD	10.732	
	Rodentia	Muridae	Dendromus vernayi	DD	10.676	
	Peramelemorphia	Peroryctidae	Echymipera clara	DD	10.645	
	Peramelemorphia	Peroryctidae	Echymipera davidi	DD	10.645	
	Peramelemorphia	Peroryctidae	Echymipera echinista	DD	10.645	
	Cetacea	Ziphiidae	Indopacetus pacificus	DD	10.572	
	Cetacea	Ziphiidae	Mesoplodon bidens	DD	10.572	
	Cetacea	Ziphiidae	Mesoplodon bowdoini	DD	10.572	
	Cetacea	Ziphiidae	Mesoplodon carlhubbsi	DD	10.572	
	Cetacea	Ziphiidae	Mesoplodon densirostris	DD	10.572	
	Cetacea	Ziphiidae	Mesoplodon europaeus	DD	10.572	
	Cetacea	Ziphiidae	Mesoplodon ginkgodens	DD	10.572	
	Cetacea	Ziphiidae	Mesoplodon grayi	DD	10.572	
	Cetacea	Ziphiidae	Mesoplodon hectori	DD	10.572	
	Cetacea	Ziphiidae	Mesoplodon layardii	DD	10.572	
	Cetacea	Ziphiidae	Mesoplodon mirus	DD	10.572	
	Cetacea	Ziphiidae	Mesoplodon peruvianus	DD	10.572	
	Cetacea	Ziphiidae	Mesoplodon stejnegeri	DD	10.572	
	Cetacea	Ziphiidae	Tasmacetus shepherdi	DD	10.572	
	Cetacea	Ziphiidae	Ziphius cavirostris	DD	10.572	
	Chiroptera	Molossidae	Chaerephon chapini	DD	10.569	
	Cetacea	Phocoenidae	Australophocaena dioptrica	DD	10.167	
	Cetacea	Phocoenidae	Neophocaena phocaenoides	DD	10.167	
	Chiroptera	Vespertilionidae	Pipistrellus minahassae	DD	9.952	
	Rodentia	Bathyergidae	Cryptomys bocagei	DD	9.942	
	Rodentia	Bathyergidae	Cryptomys foxi	DD	9.942	
	Rodentia	Bathyergidae	Cryptomys ochraceocinereus	DD	9.942	
	Carnivora	Viverridae	Genetta johnstoni	DD	9.896	
	Carnivora	Herpestidae	Mungos gambianus	DD	9.840	
	Chiroptera	Rhinolophidae	Rhinolophus subbadius	DD	9.833	
	Rodentia	Muridae	Thallomys shortridgei	DD	9.694	
	Rodentia	Muridae	Myomys ruppi	DD	9.606	
	Dasyuromorphia	Dasyuridae	Antechinus naso	DD	9.505	
	Rodentia	Muridae	Carpomys melanurus	DD	9.218	
	Rodentia	Muridae	Carpomys phaeurus	DD	9.218	
	Rodentia	Sciuridae	Heliosciurus punctatus	DD	9.134	

Rank	Order	Family	Species	IUCN	ED'	EDGE
	Rodentia	Sciuridae	Heliosciurus undulatus	DD	9.134	
	Cetacea	Delphinidae	Lagenorhynchus australis	DD	8.966	
	Dasyuromorphia	Dasyuridae	Sminthopsis leucopus	DD	8.912	
	Chiroptera	Vespertilionidae	Myotis insularum	DD	8.773	
	Rodentia	Sciuridae	Funisciurus anerythrus	DD	8.769	
	Rodentia	Sciuridae	Funisciurus bayonii	DD	8.769	
	Rodentia	Sciuridae	Funisciurus lemniscatus	DD	8.769	
	Rodentia	Sciuridae	Funisciurus leucogenys	DD	8.769	
	Rodentia	Sciuridae	Funisciurus substriatus	DD	8.769	
	Insectivora	Soricidae	Sylvisorex oriundus	DD	8.763	
	Dasyuromorphia	Dasyuridae	Antechinus wilhelmina	DD	8.759	
	Dasyuromorphia	Dasyuridae	Murexia rothschildi	DD	8.759	
	Rodentia	Myoxidae	Graphiurus christyi	DD	8.655	
	Rodentia	Myoxidae	Graphiurus kelleni	DD	8.655	
	Rodentia	Myoxidae	Graphiurus monardi	DD	8.655	
	Rodentia	Myoxidae	Graphiurus olga	DD	8.655	
	Rodentia	Myoxidae	Graphiurus surdus	DD	8.655	
	Artiodactyla	Suidae	Sus bucculentus	DD	8.642	
	Chiroptera	Rhinolophidae	Hipposideros camerunensis	DD	8.590	
	Chiroptera	Vespertilionidae	Eptesicus flavescens	DD	8.578	
	Chiroptera	Vespertilionidae	Pipistrellus inexspectatus	DD	8.496	
	Cetacea	Delphinidae	Stenella frontalis	DD	8.478	
	Cetacea	Delphinidae	Tursiops truncatus	DD	8.478	
	Cetacea	Delphinidae	Sousa chinensis	DD	8.446	
	Cetacea	Delphinidae	Sousa teuszii	DD	8.446	
	Primates	Cercopithecidae	Macaca fuscata	DD	8.379	
	Chiroptera	Vespertilionidae	Pipistrellus ariel	DD	8.361	
	Cetacea	Delphinidae	Stenella clymene	DD	8.338	
	Diprotodontia	Macropodidae	Dendrolagus spadix	DD	8.294	
	Rodentia	Muridae	Batomys dentatus	DD	8.255	
	Primates	Hylobatidae	Hylobates leucogenys	DD	8.253	
	Rodentia	Muridae	Mastomys pernanus	DD	8.103	
	Insectivora	Soricidae	Sorex excelsus	DD	8.089	
	Carnivora	Viverridae	Genetta abyssinica	DD	7.995	
	Insectivora	Soricidae	Congosorex polli	DD	7.957	
	Insectivora	Soricidae	Myosorex geata	DD	7.879	
	Insectivora	Soricidae	Myosorex schalleri	DD	7.879	
	Insectivora	Soricidae	Myosorex tenuis	DD	7.879	
	Chiroptera	Vespertilionidae	Pipistrellus lophurus	DD	7.860	
	Chiroptera	Vespertilionidae	Scotoecus albofuscus	DD	7.860	
	Chiroptera	Vespertilionidae	Scotoecus hirundo	DD	7.860	
	Cetacea	Delphinidae	Lissodelphis peronii	DD	7.855	
	Rodentia	Muridae	Grammomys buntingi	DD	7.819	
	Rodentia	Muridae	Grammomys caniceps	DD	7.819	
	Rodentia	Muridae	Oenomys ornatus	DD	7.815	
	Chiroptera	Vespertilionidae	Pipistrellus societatis	DD	7.744	
	Cetacea	Delphinidae	Lagenorhynchus obscurus	DD	7.723	
	Chiroptera	Rhinolophidae	Hipposideros schistaceus	DD	7.672	
	Chiroptera	Vespertilionidae	Kerivoula aerosa	DD	7.543	
	Rodentia	Muridae	Praomys minor	DD	7.473	
	Carnivora	Mustelidae	Mustela africana	DD	7.419	
	Primates	Cercopithecidae	Macaca ochreata	DD	7.366	
	Cetacea	Delphinidae	Cephalorhynchus commersonii	DD	7.252	
	Cetacea	Delphinidae	Cephalorhynchus eutropia	DD	7.252	
	Rodentia	Muridae	Tachyoryctes annectens	DD	7.184	
	Rodentia	Sciuridae	Paraxerus cooperi	DD	7.180	

Rank	Order	Family	Species	IUCN	ED'	EDGE
	Rodentia	Sciuridae	Paraxerus flavovittis	DD	7.180	
	Rodentia	Sciuridae	Paraxerus lucifer	DD	7.180	
	Cetacea	Delphinidae	Grampus griseus	DD	7.143	
	Cetacea	Delphinidae	Lagenodelphis hosei	DD	7.143	
	Cetacea	Delphinidae	Sotalia fluviatilis	DD	7.143	
	Cetacea	Delphinidae	Steno bredanensis	DD	7.143	
	Rodentia	Muridae	Microtus abbreviatus	DD	7.059	
	Chiroptera	Rhinolophidae	Hipposideros crumeniferus	DD	6.951	
	Chiroptera	Rhinolophidae	Hipposideros doriae	DD	6.951	
	Chiroptera	Vespertilionidae	Pipistrellus peguensis	DD	6.905	
	Primates	Cercopithecidae	Cercopithecus dryas	DD	6.783	
	Artiodactyla	Cervidae	Muntiacus feae	DD	6.646	
	Artiodactyla	Cervidae	Muntiacus gongshanensis	DD	6.646	
	Rodentia	Muridae	Lemniscomys hoogstraali	DD	6.631	
	Rodentia	Muridae	Lemniscomys linulus	DD	6.631	
	Rodentia	Muridae	Lemniscomys mittendorfi	DD	6.631	
	Rodentia	Muridae	Lemniscomys roseveari	DD	6.631	
	Rodentia	Dasyproctidae	Dasyprocta cristata	DD	6.529	
	Rodentia	Dasyproctidae	Dasyprocta kalinowskii	DD	6.529	
	Carnivora	Mustelidae	Lutra sumatrana	DD	6.283	
	Chiroptera	Vespertilionidae	Myotis oreias	DD	6.087	
	Dasyuromorphia	Dasyuridae	Phascolosorex doriae	DD	6.064	
	Carnivora	Mustelidae	Aonyx congicus	DD	5.921	
	Carnivora	Canidae	Vulpes pallida	DD	5.800	
	Artiodactyla	Cervidae	Hippocamelus antisensis	DD	5.679	
	Insectivora	Soricidae	Paracrocidura graueri	DD	5.593	
	Primates	Cercopithecidae	Presbytis frontata	DD	5.540	
	Chiroptera	Vespertilionidae	Scotophilus celebensis	DD	5.269	
	Artiodactyla	Cervidae	Cervus mariannus	DD	5.259	
	Rodentia	Muridae	Rattus timorensis	DD	5.242	
	Rodentia	Sciuridae	Spermophilus townsendii	DD	5.222	
	Carnivora	Canidae	Vulpes zerda	DD	5.120	
	Rodentia	Echimyidae	Echimys pictus	DD	5.053	
	Rodentia	Echimyidae	Echimys rhipidurus	DD	5.053	
	Rodentia	Echimyidae	Dactylomys peruanus	DD	4.836	
	Insectivora	Soricidae	Suncus malayanus	DD	4.733	
	Primates	Cercopithecidae	Presbytis hosei	DD	4.618	
	Carnivora	Mustelidae	Lontra longicaudis	DD	4.480	
	Rodentia	Muridae	Microtus bavaricus	DD	4.290	
	Rodentia	Muridae	Arborimus albipes	DD	4.258	
	Rodentia	Muridae	Arborimus pomo	DD	4.258	
	Chiroptera	Pteropodidae	Pteropus argentatus	DD	4.254	
	Carnivora	Canidae	Vulpes rueppellii	DD	4.053	
	Chiroptera	Vespertilionidae	Myotis australis	DD	3.824	
	Rodentia	Echimyidae	Mesomys didelphoides	DD	3.805	
	Rodentia	Echimyidae	Mesomys obscurus	DD	3.805	
	Chiroptera	Vespertilionidae	Eptesicus kobayashii	DD	3.744	
	Chiroptera	Vespertilionidae	Eptesicus platyops	DD	3.744	
	Chiroptera	Vespertilionidae	Eptesicus tatei	DD	3.744	
	Chiroptera	Vespertilionidae	Chalinolobus kenyacola	DD	3.711	
	Carnivora	Canidae	Atelocynus microtis	DD	3.683	
	Rodentia	Muridae	Lophuromys cinereus	DD	3.643	
	Chiroptera	Pteropodidae	Micropteropus intermedius	DD	3.506	
	Rodentia	Muridae	Apodemus alpicola	DD	3.249	
	Rodentia	Muridae	Apodemus hyrcanicus	DD	3.249	
	Chiroptera	Pteropodidae	Pteropus mearnsi	DD	3.242	

Rank	Order	Family	Species	IUCN	ED'	EDGE
	Chiroptera	Vespertilionidae	Kerivoula eriophora	DD	3.120	
	Chiroptera	Pteropodidae	Epomophorus grandis	DD	3.105	
	Chiroptera	Vespertilionidae	Myotis abei	DD	3.083	
	Rodentia	Muridae	Dicrostonyx exsul	DD	2.991	
	Rodentia	Muridae	Dicrostonyx nunatakensis	DD	2.991	
	Rodentia	Muridae	Dicrostonyx unalascensis	DD	2.991	
	Carnivora	Canidae	Pseudalopex vetulus	DD	2.962	
	Chiroptera	Vespertilionidae	Murina fusca	DD	2.942	
	Rodentia	Muridae	Acomys nesiotes	DD	2.843	
	Carnivora	Canidae	Pseudalopex sechurae	DD	2.667	
	Rodentia	Muridae	Mus callewaerti	DD	2.648	
	Rodentia	Muridae	Mus goundae	DD	2.648	
	Rodentia	Muridae	Mus neavei	DD	2.648	
	Rodentia	Muridae	Mus oubanguii	DD	2.648	
	Artiodactyla	Cervidae	Mazama americana	DD	2.224	
	Artiodactyla	Cervidae	Mazama chunyi	DD	2.224	
	Artiodactyla	Cervidae	Mazama gouazoupira	DD	2.224	
	Artiodactyla	Cervidae	Mazama nana	DD	2.224	
	Rodentia	Muridae	Oligoryzomys destructor	DD	2.123	
	Rodentia	Muridae	Oryzomys melanotis	DD	1.263	
	Chiroptera	Rhinolophidae	Rhinolophus adami	DD	1.062	
	Chiroptera	Rhinolophidae	Rhinolophus anderseni	DD	1.062	
	Chiroptera	Rhinolophidae	Rhinolophus deckenii	DD	1.062	
	Chiroptera	Rhinolophidae	Rhinolophus denti	DD	1.062	
	Chiroptera	Rhinolophidae	Rhinolophus eloquens	DD	1.062	
	Chiroptera	Rhinolophidae	Rhinolophus inops	DD	1.062	
	Insectivora	Soricidae	Crocidura andamanensis	DD	0.320	
	Insectivora	Soricidae	Crocidura armenica	DD	0.320	
	Insectivora	Soricidae	Crocidura bottegi	DD	0.320	
	Insectivora	Soricidae	Crocidura douceti	DD	0.320	
	Insectivora	Soricidae	Crocidura erica	DD	0.320	
	Insectivora	Soricidae	Crocidura fischeri	DD	0.320	
	Insectivora	Soricidae	Crocidura floweri	DD	0.320	
	Insectivora	Soricidae	Crocidura gracilipes	DD	0.320	
	Insectivora	Soricidae	Crocidura jenkinsi	DD	0.320	
	Insectivora	Soricidae	Crocidura longipes	DD	0.320	
	Insectivora	Soricidae	Crocidura macowi	DD	0.320	
	Insectivora	Soricidae	Crocidura maurisca	DD	0.320	
	Insectivora	Soricidae	Crocidura minuta	DD	0.320	
	Insectivora	Soricidae	Crocidura monax	DD	0.320	
	Insectivora	Soricidae	Crocidura mutesae	DD	0.320	
	Insectivora	Soricidae	Crocidura nana	DD	0.320	
	Insectivora	Soricidae	Crocidura nicobarica	DD	0.320	
	Insectivora	Soricidae	Crocidura osorio	DD	0.320	
	Insectivora	Soricidae	Crocidura pitmani	DD	0.320	
	Insectivora	Soricidae	Crocidura planiceps	DD	0.320	
	Insectivora	Soricidae	Crocidura polia	DD	0.320	
	Insectivora	Soricidae	Crocidura raineyi	DD	0.320	
	Insectivora	Soricidae	Crocidura religiosa	DD	0.320	
	Insectivora	Soricidae	Crocidura tarfayensis	DD	0.320	
	Insectivora	Soricidae	Crocidura ultima	DD	0.320	
	Insectivora	Soricidae	Crocidura zaphiri	DD	0.320	
	Insectivora	Soricidae	Crocidura zimmeri	DD	0.320	
	Rodentia	Muridae	Gerbillus acticola	DD	0.111	
	Rodentia	Muridae	Gerbillus agag	DD	0.111	
	Rodentia	Muridae	Gerbillus amoenus	DD	0.111	

Rank	Order	Family	Species	IUCN	ED'	EDGE
	Rodentia	Muridae	Gerbillus bottai	DD	0.111	
	Rodentia	Muridae	Gerbillus brockmani	DD	0.111	
	Rodentia	Muridae	Gerbillus burtoni	DD	0.111	
	Rodentia	Muridae	Gerbillus dalloni	DD	0.111	
	Rodentia	Muridae	Gerbillus dongolanus	DD	0.111	
	Rodentia	Muridae	Gerbillus dunni	DD	0.111	
	Rodentia	Muridae	Gerbillus grobbeni	DD	0.111	
	Rodentia	Muridae	Gerbillus jamesi	DD	0.111	
	Rodentia	Muridae	Gerbillus latastei	DD	0.111	
	Rodentia	Muridae	Gerbillus lowei	DD	0.111	
	Rodentia	Muridae	Gerbillus mauritaniae	DD	0.111	
	Rodentia	Muridae	Gerbillus nancillus	DD	0.111	
	Rodentia	Muridae	Gerbillus occiduus	DD	0.111	
	Rodentia	Muridae	Gerbillus principulus	DD	0.111	
	Rodentia	Muridae	Gerbillus rosalinda	DD	0.111	
	Rodentia	Muridae	Gerbillus somalicus	DD	0.111	
	Rodentia	Muridae	Gerbillus stigmonyx	DD	0.111	
	Rodentia	Muridae	Gerbillus syrticus	DD	0.111	
	Perissodactyla	Equidae	Equus caballus	EW	31.372	
	Artiodactyla	Bovidae	Oryx dammah	EW	6.773	
	Carnivora	Mustelidae	Mustela nigripes	EW	5.388	
	Artiodactyla	Bovidae	Gazella saudiya	EW	3.451	
	Artiodactyla	Camelidae	Camelus dromedarius	NE	27.491	
	Chiroptera	Molossidae	Tadarida espiritosantensis	NE	25.128	
	Artiodactyla	Camelidae	Lama glama	NE	23.754	
	Artiodactyla	Camelidae	Lama pacos	NE	23.740	
	Chiroptera	Emballonuridae	Saccolaimus pluto	NE	18.455	
	Chiroptera	Emballonuridae	Taphozous philippinensis	NE	17.821	
	Chiroptera	Pteropodidae	Harpyionycteris celebensis	NE	15.511	
	Primates	Hominidae	Homo sapiens	NE	14.374	
	Diprotodontia	Phalangeridae	Trichosurus arnhemensis	NE	11.937	
	Rodentia	Heteromyidae	Chaetodipus lineatus	NE	9.115	
	Lagomorpha	Leporidae	Lepus granatensis	NE	8.964	
	Primates	Cebidae	Aotus hershkovitzi	NE	8.925	
	Rodentia	Muridae	Solomys spriggsarum	NE	8.716	
	Artiodactyla	Suidae	Sus heureni	NE	8.642	
	Artiodactyla	Suidae	Sus timoriensis	NE	8.642	
	Lagomorpha	Leporidae	Lepus tolai	NE	8.265	
	Artiodactyla	Cervidae	Axis porcinus	NE	8.228	
	Artiodactyla	Bovidae	Bos taurus	NE	8.120	
	Primates	Cercopithecidae	Cercocebus agilis	NE	7.919	
	Chiroptera	Vespertilionidae	Pipistrellus mimus	NE	7.890	
	Lagomorpha	Leporidae	Lepus corsicanus	NE	7.311	
	Chiroptera	Vespertilionidae	Pipistrellus babu	NE	6.905	
	Primates	Cercopithecidae	Cercopithecus wolfi	NE	5.712	
	Rodentia	Muridae	Euneomys fossor	NE	5.198	
	Rodentia	Muridae	Melomys spechti	NE	4.846	
	Rodentia	Heteromyidae	Dipodomys elephantinus	NE	4.588	
	Rodentia	Muridae	Rattus sanila	NE	4.402	
	Rodentia	Muridae	Microtus majori	NE	4.282	
	Primates	Cercopithecidae	Trachypithecus cristatus	NE	3.726	
	Primates	Cercopithecidae	Trachypithecus phayrei	NE	3.726	
	Rodentia	Muridae	Crunomys rabori	NE	2.566	
	Artiodactyla	Cervidae	Mazama bricenii	NE	2.224	
	Chiroptera	Rhinolophidae	Rhinolophus robinsoni	NE	1.062	
	Chiroptera	Rhinolophidae	Rhinolophus simplex	NE	1.062	
