# Supplementary material for: Mammals on the EDGE: Conservation Priorities Based on Threat and Phylogeny
Source: PLoS One. 2007 Mar 14;2(3):e296. doi: 10.1371/journal.pone.0000296 (PMC1808424; doi:10.1371/journal.pone.0000296)
Supplement: Table S2 — Evolutionary Distinctiveness for primates under two species concepts. This table lists ED scores for primates under the biological species concept i[.e. the taxonomy of ref 35], the number of phylogenetic species into which the biological species was split [36] and the estimated ED score of each phylogenetic species. See Materials and Methods for further information. ED scores are lower for phylogenetic species than biological species, even for taxa whose taxonomic status is the same under both concepts (i.e. the number of phylogenetic species is one). This occurs because the total number of species in the phylogeny is greater, so each receives a smaller share of the distinctiveness of ancestral branches. ED scores were calculated using just one set of branch lengths (the ‘best’ set), so differ from those in table S1. (0.05 MB PDF) [file pone.0000296.s002.pdf]

Family	Biological species name	ED (biological)	Phylogenetic species	ED (phylogenetic)
Cheirogaleidae	Allocebus trichotis	29.311	1	26.834
Cheirogaleidae	Microcebus coquereli	28.911	1	26.354
Cheirogaleidae	Microcebus murinus	22.461	2	11.829
Cheirogaleidae	Microcebus rufus	22.461	2	11.829
Cheirogaleidae	Cheirogaleus major	29.011	6	7.065
Cheirogaleidae	Cheirogaleus medius	29.011	1	20.982
Cheirogaleidae	Phaner furcifer	38.328	4	10.452
Indridae	Avahi laniger	30.426	2	15.454
Indridae	Propithecus diadema	16.940	3	7.364
Indridae	Propithecus tattersalli	16.940	1	11.039
Indridae	Propithecus verreauxi	16.940	3	7.364
Indridae	Indri indri	33.576	1	31.688
Daubentoniidae	Daubentonia madagascariensis	43.416	1	42.758
Lemuridae	Eulemur coronatus	13.064	1	10.596
Lemuridae	Eulemur fulvus	12.864	7	3.985
Lemuridae	Eulemur macaco	12.864	1	10.328
Lemuridae	Eulemur mongoz	12.864	1	10.366
Lemuridae	Eulemur rubriventer	12.864	1	10.366
Lemuridae	Hapalemur aureus	17.921	1	16.026
Lemuridae	Hapalemur griseus	17.921	3	7.257
Lemuridae	Hapalemur simus	17.921	1	16.026
Lemuridae	Lemur catta	20.051	1	18.838
Lemuridae	Varecia variegata	20.051	2	10.343
Megaladapidae	Lepilemur dorsalis	10.450	1	10.066
Megaladapidae	Lepilemur edwardsi	10.450	1	10.066
Megaladapidae	Lepilemur leucopus	10.450	1	10.066
Megaladapidae	Lepilemur microdon	10.450	1	10.066
Megaladapidae	Lepilemur mustelinus	10.450	1	10.066
Megaladapidae	Lepilemur ruficaudatus	10.450	1	10.066
Megaladapidae	Lepilemur septentrionalis	10.450	1	10.066
Loridae	Arctocebus aureus	24.631	1	22.086
Loridae	Arctocebus calabarensis	24.631	1	22.086
Loridae	Perodicticus potto	28.181	2	15.886
Loridae	Loris tardigradus	36.981	2	19.001
Loridae	Nycticebus coucang	28.231	2	16.084
Loridae	Nycticebus pygmaeus	28.231	1	23.684
Galagonidae	Euoticus elegantulus	15.365	1	13.562
Galagonidae	Euoticus pallidus	15.365	1	13.562
Galagonidae	Galago gallarum	15.910	1	14.107
Galagonidae	Galago matschiei	15.910	1	14.107
Galagonidae	Galago moholi	21.704	2	11.479
Galagonidae	Galago senegalensis	21.704	1	19.879
Galagonidae	Otolemur crassicaudatus	13.814	2	8.169
Galagonidae	Otolemur garnettii	13.814	1	10.719
Galagonidae	Galago alleni	27.701	3	10.266
Galagonidae	Galagoides demidoff	32.412	5	7.758
Galagonidae	Galagoides zanzibaricus	32.592	5	7.787
Tarsiidae	Tarsius bancanus	21.438	1	15.960
Tarsiidae	Tarsius dianae	21.438	1	15.960
Tarsiidae	Tarsius pumilus	21.438	2	12.801
Tarsiidae	Tarsius spectrum	21.438	3	11.748
Tarsiidae	Tarsius syrichta	21.438	1	15.960
Callitrichidae	Callimico goeldii	14.303	1	14.159
Callitrichidae	Callithrix argentata	4.992	9	1.758
Callitrichidae	Callithrix humeralifera	4.992	3	2.002
Callitrichidae	Callithrix pygmaea	6.392	1	5.302
Callitrichidae	Callithrix aurita	4.042	1	3.465
Callitrichidae	Callithrix flaviceps	4.042	1	3.465
Callitrichidae	Callithrix geoffroyi	3.892	1	3.315
Callitrichidae	Callithrix kuhlii	3.892	1	3.315

Family	Biological species name	ED (biological)	Phylogenetic species	ED (phylogenetic)
Callitrichidae	Callithrix jacchus	3.892	1	3.315
Callitrichidae	Callithrix penicillata	3.892	1	3.315
Callitrichidae	Leontopithecus caissara	5.436	1	5.392
Callitrichidae	Leontopithecus chrysopygus	5.436	1	5.392
Callitrichidae	Leontopithecus rosalia	5.536	1	5.492
Callitrichidae	Leontopithecus chrysomelas	5.603	1	5.559
Callitrichidae	Saguinus bicolor	6.866	2	3.939
Callitrichidae	Saguinus midas	6.866	2	3.939
Callitrichidae	Saguinus geoffroyi	5.116	1	4.884
Callitrichidae	Saguinus oedipus	5.116	1	4.884
Callitrichidae	Saguinus leucopus	6.566	1	6.334
Callitrichidae	Saguinus imperator	7.099	1	6.618
Callitrichidae	Saguinus labiatus	6.199	1	5.418
Callitrichidae	Saguinus mystax	6.199	2	4.018
Callitrichidae	Saguinus fuscicollis	6.722	2	4.132
Callitrichidae	Saguinus tripartitus	6.722	1	5.732
Callitrichidae	Saguinus nigricollis	7.922	2	4.532
Callitrichidae	Saguinus inustus	8.781	1	8.600
Cebidae	Alouatta belzebul	6.264	2	4.022
Cebidae	Alouatta caraya	6.264	1	5.890
Cebidae	Alouatta coibensis	4.746	1	4.371
Cebidae	Alouatta palliata	4.746	1	4.371
Cebidae	Alouatta fusca	6.264	1	5.890
Cebidae	Alouatta pigra	6.264	1	5.890
Cebidae	Alouatta sara	7.680	1	7.155
Cebidae	Alouatta seniculus	7.680	2	4.805
Cebidae	Ateles belzebuth	8.952	2	5.586
Cebidae	Ateles chamek	7.752	1	7.186
Cebidae	Ateles marginatus	7.752	1	7.186
Cebidae	Ateles fusciceps	10.369	1	9.986
Cebidae	Ateles geoffroyi	10.369	1	9.986
Cebidae	Ateles paniscus	10.499	1	10.119
Cebidae	Brachyteles arachnoides	15.449	2	8.098
Cebidae	Lagothrix flavicauda	11.799	1	8.908
Cebidae	Lagothrix lagotricha	11.799	4	4.408
Cebidae	Cacajao calvus	8.153	1	8.169
Cebidae	Cacajao melanocephalus	8.153	1	8.169
Cebidae	Chiropotes albinasus	8.153	1	8.169
Cebidae	Chiropotes satanas	8.153	1	8.169
Cebidae	Pithecia aequatorialis	9.826	1	9.842
Cebidae	Pithecia albicans	7.988	1	8.004
Cebidae	Pithecia irrorata	7.988	1	8.004
Cebidae	Pithecia monachus	7.988	1	8.004
Cebidae	Pithecia pithecia	9.826	1	9.842
Cebidae	Callicebus brunneus	6.331	1	5.780
Cebidae	Callicebus hoffmannsi	6.331	2	4.480
Cebidae	Callicebus cinerascens	6.331	1	5.814
Cebidae	Callicebus moloch	6.331	1	5.814
Cebidae	Callicebus caligatus	8.073	0	n/a
Cebidae	Callicebus cupreus	8.073	2	6.956
Cebidae	Callicebus dubius	9.223	0	n/a
Cebidae	Callicebus donacophilus	7.606	2	4.680
Cebidae	Callicebus modestus	7.606	1	7.310
Cebidae	Callicebus oenanthe	7.606	1	7.310
Cebidae	Callicebus olallae	7.606	1	7.310
Cebidae	Callicebus torquatus	10.796	2	6.170
Cebidae	Callicebus personatus	8.979	1	8.991
Cebidae	Aotus azarai	8.979	1	8.991
Cebidae	Aotus brumbacki	8.979	0	n/a
Cebidae	Aotus hershkovitzi	8.979	1	8.991

Family	Biological species name	ED (biological)	Phylogenetic species	ED (phylogenetic)
Cebidae	Aotus infulatus	8.979	0	n/a
Cebidae	Aotus lemurinus	8.979	1	8.991
Cebidae	Aotus miconax	8.979	1	8.991
Cebidae	Aotus nancymaae	8.979	1	8.991
Cebidae	Aotus nigriceps	8.979	1	8.991
Cebidae	Aotus trivirgatus	8.979	1	8.991
Cebidae	Aotus vociferans	8.979	1	8.991
Cebidae	Cebus albifrons	7.820	1	5.955
Cebidae	Cebus capucinus	7.820	1	5.955
Cebidae	Cebus olivaceus	7.870	2	4.555
Cebidae	Cebus apella	9.936	4	3.830
Cebidae	Saimiri boliviensis	7.281	1	7.262
Cebidae	Saimiri vanzolinii	7.281	1	7.262
Cebidae	Saimiri oerstedii	5.935	1	5.916
Cebidae	Saimiri sciureus	5.935	1	5.916
Cebidae	Saimiri ustus	5.935	1	5.916
Cercopithecidae	Allenopithecus nigroviridis	16.085	1	15.995
Cercopithecidae	Cercopithecus ascanius	6.474	1	6.241
Cercopithecidae	Cercopithecus cephus	6.474	1	6.241
Cercopithecidae	Cercopithecus erythrogaster	6.474	2	3.710
Cercopithecidae	Cercopithecus erythrotis	6.474	1	6.241
Cercopithecidae	Cercopithecus petaurista	6.474	1	6.241
Cercopithecidae	Cercopithecus sclateri	6.474	1	6.241
Cercopithecidae	Cercopithecus mitis	11.296	4	3.473
Cercopithecidae	Cercopithecus nictitans	11.296	1	11.048
Cercopithecidae	Cercopithecus campbelli	6.258	2	3.866
Cercopithecidae	Cercopithecus mona	6.258	1	5.316
Cercopithecidae	Cercopithecus pogonias	5.808	1	4.716
Cercopithecidae	Cercopithecus wolfi	5.808	2	3.716
Cercopithecidae	Cercopithecus diana	6.824	2	4.226
Cercopithecidae	Cercopithecus dryas	6.824	1	5.726
Cercopithecidae	Cercopithecus neglectus	8.729	1	8.555
Cercopithecidae	Cercopithecus hamlyni	12.038	1	11.869
Cercopithecidae	Cercopithecus lhoesti	4.532	1	3.548
Cercopithecidae	Cercopithecus preussi	4.532	1	3.548
Cercopithecidae	Cercopithecus solatus	4.582	1	3.598
Cercopithecidae	Chlorocebus aethiops	8.182	6	2.531
Cercopithecidae	Erythrocebus patas	8.332	1	7.375
Cercopithecidae	Miopithecus talapoin	15.894	2	8.251
Cercopithecidae	Cercocebus agilis	8.166	3	3.911
Cercopithecidae	Cercocebus galeritus	8.166	1	6.644
Cercopithecidae	Cercocebus torquatus	8.870	2	4.947
Cercopithecidae	Mandrillus leucophaeus	6.347	1	5.614
Cercopithecidae	Mandrillus sphinx	6.347	1	5.614
Cercopithecidae	Lophocebus albigena	14.097	3	5.112
Cercopithecidae	Papio hamadryas	13.197	5	3.506
Cercopithecidae	Theropithecus gelada	13.197	1	11.346
Cercopithecidae	Macaca arctoides	9.389	1	9.228
Cercopithecidae	Macaca assamensis	6.714	1	6.553
Cercopithecidae	Macaca thibetana	6.714	1	6.553
Cercopithecidae	Macaca radiata	9.264	1	9.103
Cercopithecidae	Macaca sinica	9.264	1	9.103
Cercopithecidae	Macaca cyclopis	8.212	1	8.051
Cercopithecidae	Macaca mulatta	8.212	1	8.051
Cercopithecidae	Macaca fuscata	8.312	1	8.151
Cercopithecidae	Macaca fascicularis	8.979	1	8.818
Cercopithecidae	Macaca maura	6.449	1	6.010
Cercopithecidae	Macaca nigra	7.516	2	4.352
Cercopithecidae	Macaca ochreata	7.516	1	7.052
Cercopithecidae	Macaca tonkeana	6.449	2	3.805

Family	Biological species name	ED (biological)	Phylogenetic species	ED (phylogenetic)
Cercopithecidae	Macaca nemestrina	6.538	3	3.416
Cercopithecidae	Macaca silenus	6.538	1	4.616
Cercopithecidae	Macaca sylvanus	8.300	1	8.139
Cercopithecidae	Colobus angolensis	8.101	1	7.399
Cercopithecidae	Colobus guereza	6.601	1	5.399
Cercopithecidae	Colobus polykomos	6.601	2	4.449
Cercopithecidae	Colobus satanas	9.901	1	9.424
Cercopithecidae	Procolobus badius	7.242	1	6.441
Cercopithecidae	Procolobus preussi	7.242	1	6.441
Cercopithecidae	Procolobus pennantii	8.035	6	2.376
Cercopithecidae	Procolobus rufomitratus	8.035	1	7.234
Cercopithecidae	Procolobus verus	11.031	1	10.494
Cercopithecidae	Nasalis concolor	7.882	1	7.695
Cercopithecidae	Nasalis larvatus	7.882	1	7.695
Cercopithecidae	Presbytis comata	4.708	1	4.436
Cercopithecidae	Presbytis hosei	4.708	1	4.436
Cercopithecidae	Presbytis thomasi	4.708	1	4.436
Cercopithecidae	Presbytis femoralis	4.955	4	2.094
Cercopithecidae	Presbytis melalophos	4.955	1	3.744
Cercopithecidae	Presbytis frontata	5.750	1	5.478
Cercopithecidae	Presbytis potenziani	5.750	1	5.478
Cercopithecidae	Presbytis rubicunda	5.750	1	5.478
Cercopithecidae	Semnopithecus entellus	6.263	7	1.601
Cercopithecidae	Trachypithecus auratus	3.840	2	2.214
Cercopithecidae	Trachypithecus cristatus	3.840	3	1.823
Cercopithecidae	Trachypithecus francoisi	3.840	5	1.509
Cercopithecidae	Trachypithecus geei	2.945	1	2.142
Cercopithecidae	Trachypithecus pileatus	2.945	2	1.942
Cercopithecidae	Trachypithecus obscurus	3.840	1	3.390
Cercopithecidae	Trachypithecus phayrei	3.840	1	3.390
Cercopithecidae	Trachypithecus johnii	6.263	1	6.011
Cercopithecidae	Trachypithecus vetulus	6.263	1	6.011
Cercopithecidae	Pygathrix avunculus	6.670	1	6.457
Cercopithecidae	Pygathrix bieti	5.642	1	5.429
Cercopithecidae	Pygathrix brelichi	5.642	1	5.429
Cercopithecidae	Pygathrix roxellana	5.642	1	5.429
Cercopithecidae	Pygathrix nemaeus	10.870	3	4.124
Hylobatidae	Hylobates agilis	6.237	2	4.240
Hylobatidae	Hylobates lar	6.237	1	5.706
Hylobatidae	Hylobates moloch	6.237	1	5.706
Hylobatidae	Hylobates muelleri	6.237	1	5.706
Hylobatidae	Hylobates pileatus	6.237	1	5.706
Hylobatidae	Hylobates klossii	7.966	1	7.441
Hylobatidae	Hylobates hoolock	11.216	1	10.784
Hylobatidae	Hylobates syndactylus	11.388	1	10.959
Hylobatidae	Hylobates concolor	8.279	2	5.426
Hylobatidae	Hylobates leucogenys	8.279	1	6.826
Hylobatidae	Hylobates gabriellae	10.579	2	6.193
Hominidae	Pongo pygmaeus	21.691	2	11.478
Hominidae	Gorilla gorilla	16.441	2	9.378
Hominidae	Pan paniscus	11.541	1	10.828
Hominidae	Pan troglodytes	11.541	1	10.828
Hominidae	Homo sapiens	14.441	1	13.728
